# Prognosis and personalized treatment prediction in lung adenocarcinoma: An *in silico* and *in vitro* strategy adopting cuproptosis related lncRNA towards precision oncology

**DOI:** 10.3389/fphar.2023.1113808

**Published:** 2023-02-15

**Authors:** Chao Ma, Feng Li, Zhanfeng He, Song Zhao, Yang Yang, Zhuoyu Gu

**Affiliations:** Department of Thoracic Surgery, The First Affiliated Hospital of Zhengzhou University, Zhengzhou, China

**Keywords:** lung adenocarcinoma, lncRNA, signature, cuproptosis, targets, therapeutic agent, prognosis

## Abstract

**Background:** There is a rapid increase in lung adenocarcinomas (LUAD), and studies suggest associations between cuproptosis and the occurrence of various types of tumors. However, it remains unclear whether cuproptosis plays a role in LUAD prognosis.

**Methods:** Dataset of the TCGA-LUAD was treated as training cohort, while validation cohort consisted of the merged datasets of the GSE29013, GSE30219, GSE31210, GSE37745, and GSE50081. Ten studied cuproptosis-related genes (CRG) were used to generated CRG clusters and CRG cluster-related differential expressed gene (CRG-DEG) clusters. The differently expressed lncRNA that with prognosis ability between the CRG-DEG clusters were put into a LASSO regression for cuproptosis-related lncRNA signature (CRLncSig). Kaplan–Meier estimator, Cox model, receiver operating characteristic (ROC), time-dependent AUC (tAUC), principal component analysis (PCA), and nomogram predictor were further deployed to confirm the model’s accuracy. We examined the model’s connections with other forms of regulated cell death, including apoptosis, necroptosis, pyroptosis, and ferroptosis. The immunotherapy ability of the signature was demonstrated by applying eight mainstream immunoinformatic algorithms, TMB, TIDE, and immune checkpoints. We evaluated the potential drugs for high risk CRLncSig LUADs. Real-time PCR in human LUAD tissues were performed to verify the CRLncSig expression pattern, and the signature’s pan-cancer’s ability was also assessed.

**Results:** A nine-lncRNA signature, CRLncSig, was built and demonstrated owning prognostic power by applied to the validation cohort. Each of the signature genes was confirmed differentially expressed in the real world by real-time PCR. The CRLncSig correlated with 2,469/3,681 (67.07%) apoptosis-related genes, 13/20 (65.00%) necroptosis-related genes, 35/50 (70.00%) pyroptosis-related genes, and 238/380 (62.63%) ferroptosis-related genes. Immunotherapy analysis suggested that CRLncSig correlated with immune status, and checkpoints, KIR2DL3, IL10, IL2, CD40LG, SELP, BTLA, and CD28, were linked closely to our signature and were potentially suitable for LUAD immunotherapy targets. For those high-risk patients, we found three agents, gemcitabine, daunorubicin, and nobiletin. Finally, we found some of the CRLncSig lncRNAs potentially play a vital role in some types of cancer and need more attention in further studies.

**Conclusion:** The results of this study suggest our cuproptosis-related CRLncSig can help to determine the outcome of LUAD and the effectiveness of immunotherapy, as well as help to better select targets and therapeutic agents.

## Introduction

In China and throughout the world, lung cancer is one of the most common malignant tumors (Chen et al., 2019; Sung et al., 2021). According to WHO statistics, in 2020, 2,206,771 new lung cancer patients were diagnosed globally, accounting for 11.4% of new cancer cases; deaths were ranked the first among all cancer types, accounting for 18% of all cancer deaths (Chen et al., 2019; Sung et al., 2021). Adenocarcinoma (LUAD) accounts for approximately 40% of lung cancer cases (Siegel et al., 2021). Current treatment methods, such as surgical, radiotherapy, and chemotherapy, can hardly meet advanced LUAD patients’ survival expectations (Siegel et al., 2021; Li et al., 2022a). Therefore, the discovery of more effective prognostic models is crucial for exploring the cellular and molecular mechanism of LUAD carcinogenesis and finding treatment strategies.

Cell suicide pathways, termed regulated cell death, play a critical role in organismal development, homeostasis, and pathogenesis (Strasser and Vaux, 2020). Regulated cell death has been observed in cancer for a long time, but the initial reports were mainly in cases where necrosis was observed in hypoxic areas of growing tumors (Strasser and Vaux, 2020). During further tumor progression, cancer cells often respond to their altered state through programmed regulated cell death and are highly dependent on certain survival signals from their environment (Strasser and Vaux, 2020). Regulated cell death has long been implicated in cancer treatment, with radiation and chemotherapy killing cancer cells while destroying normal, healthy cells (Strasser and Vaux, 2020). The biology and therapeutic response of LUAD are reported shaped by various forms of regulated cell death, such as apoptosis (Wu et al., 2019), necroptosis (Lu et al., 2022), pyroptosis (Lin et al., 2021), and ferroptosis (Ma et al., 2021; Zhang et al., 2021). Excitingly, Tsvetkov and colleagues published their latest study in the journal *Science*, confirming the existence of copper-induced regulated cell death, which is termed cuproptosis (Tsvetkov et al., 2022). In their study, it was shown that cuproptosis is distinct from apoptosis, necroptosis, pyroptosis, and ferroptosis (Tsvetkov et al., 2022). Recently, the teams of Zhang et al. (2022a); Zhang et al. (2022b) separately developed cuproptosis-related prognostic models for hepatocellular carcinoma. Li et al. (2022b) also conducted in-depth research on cuproptosis and constructed a prediction model for oral squamous cell carcinoma. Bian et al. (2022); Ji et al. (2022) have established cuproptosis models of clear cell renal cell carcinoma, respectively, both of which claim to be effective in predicting the prognosis of the disease.

Given that some forms of regulated cell death may be more immune-targeted than others, understanding how cuproptosis is initiated, propagated, and ultimately executed in LUAD may have important implications for possible combined diagnostic and therapeutic interventions. Regarding the prognostic model about cuproptosis, no LUAD research so far has been published. Most studies of prognostic signatures target entire tumor populations without tailoring high-risk patients, making them insufficient for risk stratification or treatment (Ma et al., 2018; Ma et al., 2020a; Ma et al., 2020b; Li et al., 2020; Zheng et al., 2020; Ma et al., 2021; Zhang et al., 2021; Ma et al., 2022a; Ma et al., 2022b). To cope with the above issues, we first developed a cuproptosis-related lncRNA prognostic signature of LUAD and potential therapeutic targets and drugs for high-risk patients. We gathered proved cuproptosis-related genes to construct a lncRNA signature having the power to predict LUAD outcomes, and further validated its prognostic ability in a large independent cohort. We also tested the signature genes’ expression profile in real world and the model’s connections with other forms of regulated cell death. More importantly, we revealed that KIR2DL3, IL10, IL2, CD40LG, SELP, BTLA, and CD28, were linked closely to our signature and were potentially suitable for LUAD immunotherapy targets. We found three agents, gemcitabine, daunorubicin, and nobiletin, for those high-risk patients. Finally, we tested the ability of our signature lncRNAs in pan-cancer.

## Materials and methods

### Data selection and preprocessing

We downloaded the RNA-seq data and clinical phenotype of patients in the TCGA-LUAD project from the Xena Hub (https://xenabrowser.net/). Use the keyword “lung adenocarcinoma” to search in Gene Expression Omnibus (Clough and Barrett, 2016) (GEO, https://www.ncbi.nlm.nih.gov/geo/), and filter out the datasets containing total RNA and the total number of lung cancer patients with survival time greater than 80 in the results as candidate validation cohorts. GSE29013, GSE30219, GSE31210, GSE37745, and GSE50081 were selected, and their data were downloaded. The TCGA-LUAD were gathered as training cohort. For the preprocessing of GSE29013, GSE30219, GSE31210, GSE37745, and GSE50081, we used the R package “inSilicoMerging” to merge them (Taminau et al., 2012), and then we adopted the method that developed by Johnson et al. (2007). To remove the batch effect and finally obtained the data matrix treated as a validation cohort.

### Identification of cuproptosis-regulated genes (CRG) subgroups using consensus clustering

In the present research, we retrospectively selected 10 CRG (FDX1, LIAS, LIPT1, DLD, DLAT, PDHA1, PDHB, MTF1, GLS and CDKN2A) from the work of Tsvetkov et al. (2022). We used the “limma” R package to determine the co-expression genes of the ten CRG with the parameters of correlation coefficient >0.4 or < −0.4 and *p* < 0.05. The “ConsensusClusterPlus” R package was used to cluster CRG co-expression genes in LUAD patients extracted from the training cohort into different subtypes. The counts of the subtypes were determined by the optimal k value. The Kaplan-Meier estimator (KM) curve was constructed using R packages “survival” and “survminer” to measure difference in overall survival of patients in the CRG clusters. The “scatterplot3d” R package was applied to perform principal component analysis (PCA) to see the clusters differences. Packages “reshape2,” “ggpubr,” “limma,” “GSEABase,” and “GSVA” were used to conduct the single-sample gene set enrichment analysis (ssGSEA) and visualization. We then screened the CRG cluster-related differential expressed genes (CRG-DEGs) between clusters using the R package “limma” with Log FC > 0.15 or < −0.15 and adjust *p* < 0.05. These CRG-DEGs were the put into an KEGG analysis for the potential pathways finding.

### Constructions of CRG-DEG clusters and a cuproptosis-regulated lncRNA signature (CRLncSig)

With the help of the “ConsensusClusterPlus” R package and the CRG-DEGs, we were able to group the patients in the training cohort into different CRG-DEG clusters. The survival difference of CRG-DEG clusters was measured using the KM curve calculated using the R packages “survival” and “survminer”. The “scatterplot3d” R package was applied to perform PCA to see the clusters differences. Packages “reshape2,” “ggpubr,” “limma,” “GSEABase,” and “GSVA” were used to conduct the ssGSEA analysis and visualization. We then conducted GSVA to screen the most important KEGG pathways by comparing CRG-DEG clusters by R packages “limma,” “GSEABase,” “GSVA,” and “pheatmap”. We investigated the differently expressed lncRNA (DEL) between the CRG-DEG clusters with Log FC > 0.15 or < −0.15 and adjust *p* < 0.05. Then, these DELs were subjected to univariate Cox and KM analyses for choosing the ones with potential prognostic power with *p* < 0.05. In order to prevent overfitting, we performed the least absolute shrinkage and selection operator (LASSO) using the R package “glmnet” on the prognostic DELs. A 10-fold cross-validation was performed in the training cohort at 1 SE above the minimum partial likelihood deviation to estimate the penalty parameter (Tibshirani, 1997; Sauerbrei et al., 2007; Friedman et al., 2010; Goeman, 2010). For each patient, a risk score was calculated according to the following formula:

$$Risk\;score=\overset{n}{\underset{i}{\sum }}Expi*\beta i$$

with βi denoting the coefficient, Expi denoting the relative expression level of each lncRNA normalized by z-score, and n denoting each lncRNA in the CRLncSig.

### Validation of the CRLncSig in an indenpendent cohort

High-risk and low-risk patients were divided using median risk scores. In both the training and validation cohorts, the CRLncSig’s predictive capability was evaluated using methods such as the KM curve, univariate and multivariable Cox analysis (Cao and Lopez-de-Ullibarri, 2019), receiver operating characteristic (ROC) curve, time-dependent AUC (tAUC) analysis, and PCA. ROC curve and tAUC analyzes were implemented with the help of the “timeROC” and “survival” R packages. The “scatterplot3D” R package was used to evaluate the distribution of patients with different risk scores by PCA. Additionally, we used the R packages “survival,” “survminer,” “rms,” and “regplot,” to construct nomograms that predicted 1-, 3-, and 5-year overall survival and calibrated the model to determine whether the model’s predictions matched up with the actual consistency.

### Correlations between the cuproptosis-related lncRNA signature and apoptosis, necroptosis, pyroptosis, and ferroptosis

For better knowing the interactions between our CRLncSig and other types of “cell death”, we adopted the Pearson analysis. Apoptosis, necroptosis, and pyroptosis-related genes were extracted from the GeneCard and Gene Set Enrichment Analysis (GSEA) online databases, respectively, by applying the following steps: 1) search the GeneCard using the corresponding keyword; 2) search the GSEA using the corresponding keyword: 3) merge the above results and take the unique genes. FerrDb is the first database dedicated to ferroptosis regulators and ferroptosis-related diseases (Zhou and Bao, 2020). Ferroptosis-related genes were obtained from FerrDb (http://www.datjar.com:40013/bt2104/).

### GSEA

We first downloaded the GSEA program from http://www.gsea-msigdb.org/gsea/downloads.jsp, the GSEA website. By dividing LUADs by their median risk scores, we classified them as low-risk and high-risk. From the Molecular Signatures Database (Liberzon et al., 2011) (http://www.gsea-msigdb.org/gsea/downloads.jsp), we downloaded “c2.cp.kegg.v7.4.symbols.gmt” to evaluate the assess of KEGG pathways between high-risk and low-risk groups using GSEA. GSEA parameter settings were set to five minimum genes, 5,000 maximum genes, and 1,000 resamplings. A statistically significant value was a *p*-value of 0.05 and a false discovery rate of 0.25.

### Identification of the immunological status of the CRLncSig

We calculated stromal, immune, and ESTIMATE scores for each patient based on gene expression in the training cohort using the R package “ESTIMATE” (Yoshihara et al., 2013). We calculated the association between CRLncSig and stromal, immune, and ESTIMATE scores using the Pearson coefficient and Wilcoxon rank sum. Multi-omics data can be leveraged with “IOBR” to facilitate immuno-oncology exploration, revealing tumor-immune interactions and accelerating precision immunotherapy. To calculate the immune-infiltrating cell scores for each sample of the training cohort, we used the R package “IOBR” and its methods of CIBERSORT, CIBERSORT-ABS, quantIseq, TIMER, IPS, MCPCounter, xCell, and EPIC. CRLncSig’s relationships with immune cell types of the eight algorithms were calculated using Pearson coefficients and Wilcoxon rank-sum, and lollipop plots and heatmap were applied for the visualization. Venn and cloud diagrams were used to summarize the results we obtained. As part of the “gsva” R package, the “ ssGSEA” function was used to assess 13 immune-related pathways of the CRLncSig.

### Identification of the immunotherapy role and immune checkpoint target of CRLncSig

Using the “maftools,” the top 20 mutated genes were identified, and the mutations and their frequencies were visualized across all training cohort samples. Based on the median risk score of LUADs, we divided them into two groups. To compare gene mutation frequencies between low- and high-risk LUAD populations, we used the chi-square test. Tumor mutational burden (TMB) is an emerging therapeutic measure of immunotherapy sensitivity. TMB is defined as the frequency of certain mutations within a tumor’s genes (Chalmers et al., 2017). The TMB rank score of each case with LUAD was obtained as previously described (Chalmers et al., 2017). We deployed the Pearson coefficient together with the Wilcoxon rank-sum to calculate the connections between the risk score and TMB. Tumor Immune Dysfunction and Exclusion (TIDE) is a computational framework that integrates T cell dysfunction expression signatures and T cell exclusion to model tumor immune evasion. Using TIDE, tumor immune evasion can be modeled in two ways and can be used to predict immunotherapy outcomes (Jiang et al., 2018; Fu et al., 2020; Chen et al., 2021a). More importantly, we tested if our signature correlated with the TIDE. A total of 60 immune checkpoints were selected from previous studies (Thorsson et al., 2018) (Supplementary Table S1), including 24 inhibitory and 36 stimulatory checkpoints. We measured the correlation between our signature and these 60 immune checkpoints based on Pearson and Wilcoxon rank-sum analysis. To further test whether our CRLncSig could guide immunotherapy, we deployed the Kaplan–Meier estimator and Cox regression to test the prognostic ability of the 60 immune checkpoints. Venn diagram was used to summarize the above results to find good checkpoints that may have the targeting ability of the CRLncSig. We collected data from multiple immune datasets to further evaluate the potential impact of these promising checkpoint genes on the immune system and immunotherapy. We presented them in a heatmap format with the help of the regulator prioritization module of the TIDE online tool (Fu et al., 2020).

### Identification of drugs for high risk score LUADs

High-risk and low-risk patients were divided using median risk scores. Data on expression profiles and somatic mutations in human cancer cell lines (CCLs) were acquired from the Broad Institute Cancer Cell Line Encyclopedia (CCLE) (https://portals.broadinstitute.org/ccle/) (Ghandi et al., 2019). The dependency map (DepMap, https://de.pmap.org/portal/) portal was used to collect the CERES scores of genome-scale CRISPR knockout screens on 18,333 genes in 739 cell lines. CERES scores indicate how dependent a gene is on certain CCLs. The lower the CERES score, the greater the likelihood that the gene plays an important role in the growth and survival of a given CCL. Based on PRISM Repurposing (https://depmap.org/portal/prism/) and the Cancer Therapeutics Response Portal (https://portals.broadinstitute.org/ctrp), drug sensitivity data of CCLs were gathered. In the CTRP, 481 compounds have been evaluated over 835 CCLs, while in the PRISM, 1,448 compounds have been evaluated over 482 CCLs. In both datasets, the area under the dose-response curve (area under the curve - AUC) represents drug sensitivity, and lower values indicate greater sensitivity. To identify drug candidates with higher drug sensitivity in patients with high risk scores, CTRP and PRISM derived drug response data were analyzed. To identify compounds with lower estimated AUC values in the high risk score group, a differential drug response analysis (log2FC > 0.9) was conducted between the groups of the top decile score and bottom decile score (Yang et al., 2021). As a next step, Spearman correlation analysis between AUC value and risk score was conducted to select compounds with a negative correlation coefficient (Spearman’s r < −0.09) (Yang et al., 2021).

### Validation of drugs using connectivity map (CMap) analysis

These above results were then put into multiple perspective analyses, including clinical trial data, published experiment evidence, and CMap for further confirming their effective in LUAD (Yang et al., 2021). CMap analysis was performed as a complement to investigate the therapeutic potential of candidate agents in LUAD. We first conducted differential expression analysis between tumor and normal samples. Next, 300 genes with the most significant fold changes (150 upregulated genes and 150 downregulated genes) were submitted to the CMap website (https://clue.io/query). Gene expression signature of perturbations in this website are derived from both CMap v2 and Library of Integrated Network-Based Cellular Signatures (LINCS) database, and a total of 2,429 compounds are available for CMap analysis. CMap calculates a connectivity score for each perturbation, which ranges from −100 to 100. Specific perturbations with negative scores have opposite gene expression patterns to a particular disease, suggesting they have therapeutic potential.

### Validation of the CRLncSig’s expression profile using the real-time PCR and human LUAD tissues

In order to verify the expression levels of each lncRNA of CRLncSig, nine pairs of LUAD tissues and adjacent normal tissues were examined using real-time PCR. The Ethics Review Committee of the First Affiliated Hospital of Zhengzhou University approved this study. Informed consent was obtained from all patients before surgery, no history of chemotherapy or radiotherapy was present before surgery. After extracting the tissues, we froze and stored them in liquid nitrogen. Total RNA was extracted from the sample tissues *via* Trizol lysate (Thermo Fisher Scientific). We reverse-transcribed total RNA from clinical samples into cDNA using the HiScript III RT SuperMix Kit (R32301, Vazyme, Nanjing, China). The real-time PCR was performed using the CFX96 system (BIO-RAD Laboratories, Inc., Hercules, CA, United States). Based on the 2^−ΔΔCT^ method, expression levels of target RNAs were normalized to GAPDH. The mean value was used as the final experimental result for replicated wells. The manufacturer’s instructions were followed during all procedures. Student’s t-test was applied to identify genes differentially expressed between normal and tumor samples. Differentially expressed genes were defined as adjusted *p*-value < 0.05.

### Pan-cancer ability determination of each lncRNA of the CRLncSig

First, we downloaded the TCGA TARGET GTEx dataset from the UCSC database (https://xenabrowser.net/) to determine whether CRLncSig lncRNAs are differentially expressed in tumors and normal tissues. In order to complete this step, we also excluded cancer types with fewer than three samples, giving us data on 34 cancer types. A Wilcoxon rank sum test was used in R software to determine the expression difference between normal and tumor samples in each tumor type.

For prognostic assessment, in addition to the TCGA TARGET GTEx dataset we got, we also obtained a high-quality TCGA prognostic dataset from the TCGA prognostic study previously published on Cell (Liu et al., 2018). A supplement of TARGET follow-up data was obtained from UCSC, and samples with a short than 30 days follow-up period were eliminated. Additionally, we eliminated cancer types with fewer than ten samples each and obtained data on 44 cancer types, including expression and overall survival. Based on the Cox proportional hazards regression model built with the R package “survival,” we analyzed the relationship between the lncRNAs and the prognosis of each cancer type.

In the final step, we evaluated the staging ability of each lncRNA. The TCGA TARGET GTEx dataset was obtained above, and the cancer types with fewer than three samples were eliminated, as were cancer types without tumor staging data. Finally, we obtained information on 30 cancer types. For each cancer type’s different clinical stages, we calculated expression distributions of CRLncSig lncRNAs using R software and utilized variance analysis to investigate the differences.

## Results

### Patient characteristics

**FIGURE 1:**
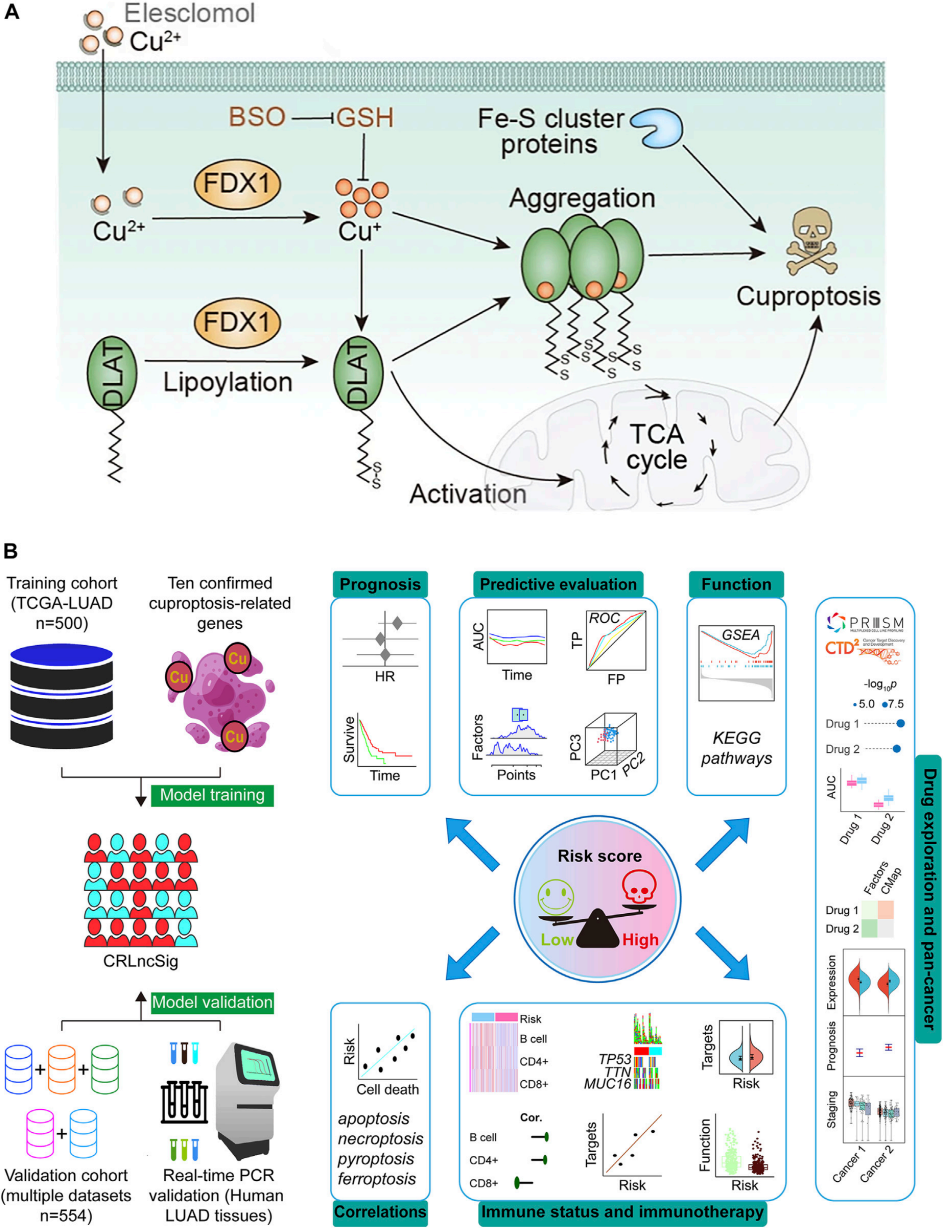
Schematic illustration indicates the mechanism of cuproptosis induction **(A)** (Hu et al., 2022; Tsvetkov et al., 2022) and research design **(B)**. DLAT, dihydrolipoamide S-acetyltransferase; FDX1, ferredoxin-1; Fe–S, iron–sulfur; TCA, tricarboxylic acid; GSH, Glutathione; BSO, buthionine sulfoximine; CRLncSig, cuproptosis-regulated lncRNA signature; TCGA, The Cancer Genome Atlas; LUAD, lung adenocarcinoma; HR, hazard ratio; ROC, receiver operating characteristic; TP, true positive rate; FP, false positive rate; AUC, area under the ROC curve; PC, principal component; KEGG, Kyoto Encyclopedia of Genes and Genomes; GSEA, Gene Set Enrichment Analysis.

**FIGURE 2:**
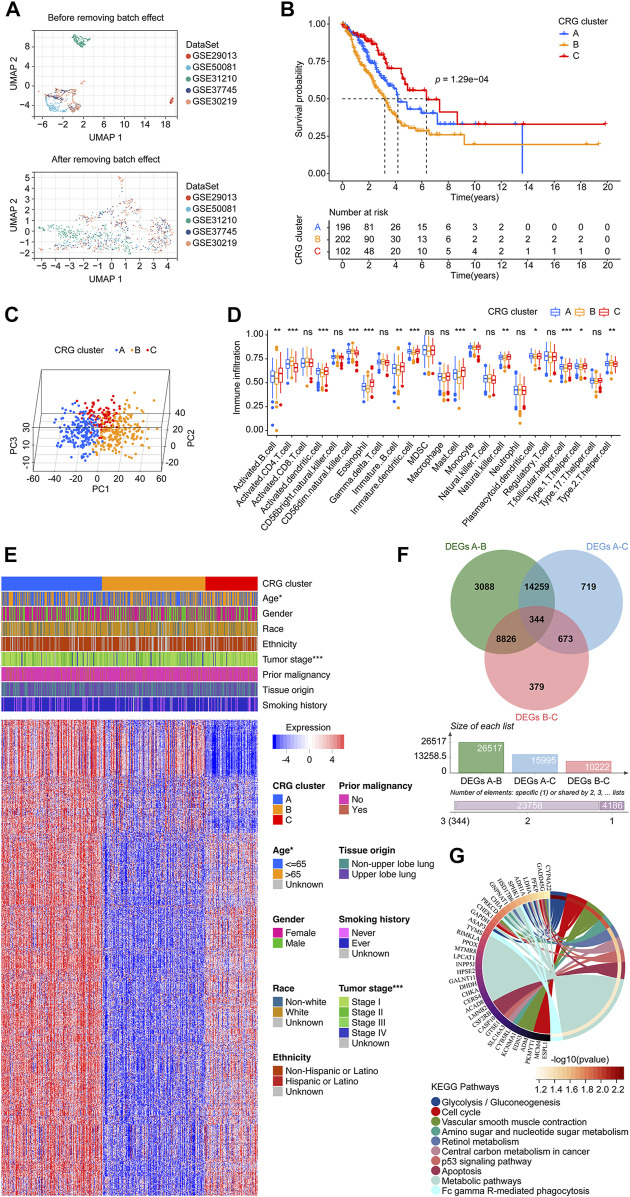
Data preprocessing and the CRG clusters construction. **(A)** UMAP plot shows validation cohort merged before batch effect removing, displaying that the samples of each dataset are separated from each other. UMAP plot shows validation cohort merged after batch effect removing, displaying that samples of each dataset are clustered and intertwined with each other. **(B)** The KM survival curve demonstrated that the overall survival of patients in the CRG clusters were significantly different. The cluster C had a more favor prognosis better than that of patients in cluster B and C, while the cluster B population had a promising outcome than that in the cluster C. **(C)** Principal component analysis scatter plot shows three clusters were obviously separated. **(D)** Infiltration distribution of immune cells in the three CRG clusters. **(E)** Heatmap shows the relationship between CRG clusters, clinical parameters, and 1175 CRG co-expressed genes. Each row represents a gene, and each column represents a sample. **(F)** Venn diagram shows the process of obtaining the 344 CRG-DEGs. **(G)** KEGG analysis shows the top 10 enriched pathways. CRG: cuproptosis-regulated gene; UMAP: Uniform Manifold Approximation and Projection; KM: Kaplan–Meier estimator; DEGs: differentially expressed genes; KEGG: Kyoto Encyclopedia of Genes and Genomes; *p-value* < 0.05 was considered statistically significant; *: *p-*value <0.05; ***: *p-value* < 0.001.

As Figure 1B demonstrates, 500 LUAD patients of project TCGA-LUAD were treated as a training cohort for model construction. 554 LUADs from the GSE29013, GSE30219, GSE31210, GSE37745, and GSE50081 datasets were taken and merged as a validation cohort for model certifying. The effect of data merging for the validation cohort is shown in Figure 2. According to the UMAP plot (Figure 2A), each dataset’s samples are separated before the batch effect is removed. A clustering and intertwining of each data set were observed after Johnson et al. (2007) method had been applied, indicating that the batch effect is well removed by this method. The clinical parameters of the enrolled patients in each cohort are shown in Table 1.

**TABLE 1:** Clinical characteristics of patients enrolled in the study.

Characteristics	Training cohort	Validation cohort
	(TCGA-LUAD, *n* = 500)	(GSE29013, GSE30219, GSE31210, GSE37745, and GSE50081, *n* = 554)
Age		
<65	219 (43.8%)	315 (56.86%)
≥65	271 (54.2%)	239 (43.14%)
Unknown	10 (2%)	0
Gender		
Female	270 (54%)	265 (47.83%)
Male	230 (46%)	289 (52.17%)
Race		
White	386 (77.2%)	NA
Non-White	60 (12%)	NA
Unknown	54 (10.8%)	NA
Ethnicity		
Hispanic or Latino	7 (1.4%)	NA
Non-Hispanic or Latino	381 (76.2%)	NA
Unknown	112 (22.4%)	NA
Tumor stage		
Stage I	268 (53.6%)	339 (61.19%)
Stage II	119 (23.8%)	108 (19.49%)
Stage III	80 (16%)	21 (3.79%)
Stage IV	25 (5%)	4 (0.72%)
Unknown	8 (1.6%)	82 (14.8%)
Prior malignancy		
Yes	79 (15.8%)	NA
No	421 (84.2%)	NA
Tissue origin		
Upper lobe lung	291 (58.2%)	NA
Non-upper lobe lung	209 (41.8%)	NA
Smoking history		
Ever	415 (83%)	216 (38.99%)
Never	71 (14.2%)	139 (25.09%)
Unknown	14 (2.8%)	199 (35.92%)
Vital status		
Alive	318 (63.6%)	348 (62.82%)
Dead	182 (36.4%)	206 (37.18%)

### Evaluation of the CRG clusters in LUADs using consensus clustering

We first explored the co-expressed genes of 10 CRGs according to our set screening criteria. After removing duplicate results, we finally identified 1,175 genes as CRG co-expressed genes (Supplementary Table S2). An algorithm of consensus clustering classified LUADs from the training cohort into three CRG clusters based on CRG co-expressed genes. A significant difference in the overall survival of patients in the CRG cluster was found with the KM survival curves. Cluster C had a better prognosis than patients in clusters A and B. Populations in cluster A had a better prognosis than those in cluster B (Figure 2B). The PCA revealed that the cluster A, B, and C were obviously separated from each other (Figure 2C). The infiltration level of the different immune cell populations in CRG clusters was determined by ssGSEA. As shown in Figure 2D, 14 immune cells, including Activated B cell, Activated CD4 T cell, Activated dendritic cell, CD56dim natural killer cell, Eosinophil, Immature B cell, Immature dendritic cell, Mast cell, Monocyte, Natural killer cell, Plasmacytoid dendritic cell, T follicular helper cell, Type 1 T helper cell, and Type 2 T helper cell were recognized significantly distributed in three CRG clusters. Furthermore, there was a statistically significant difference between the three clusters of LUAD patients in terms of age and tumor stage (Figure 2E). For exploring the mechanism of the cluster pattern, we first investigated the different expressed genes between each two clusters, finding there were 26,517 between cluster A and B, 15,995 between cluster A and C, and 10,222 between cluster B and C. We take the intersection of the above three parts and learned 344 CRG-DEGs (Figure 2F; Supplementary Table S3). Then, the KEGG method was used to assess the signaling mechanisms using the CRG-DEGs, which of the top 10 pathways were shown in Figure 2G, including Glycolysis/Gluconeogenesis, Cell cycle, Vascular smooth muscle contraction, Amino sugar and nucleotide sugar metabolism, Retinol metabolism, Central carbon metabolism in cancer, p53 signaling pathway, Apoptosis, Metabolic pathways, and Fc gamma R-mediated phagocytosis.

### Two CRG-DEG clusters identification and a CRLncSig generated

**FIGURE 3:**
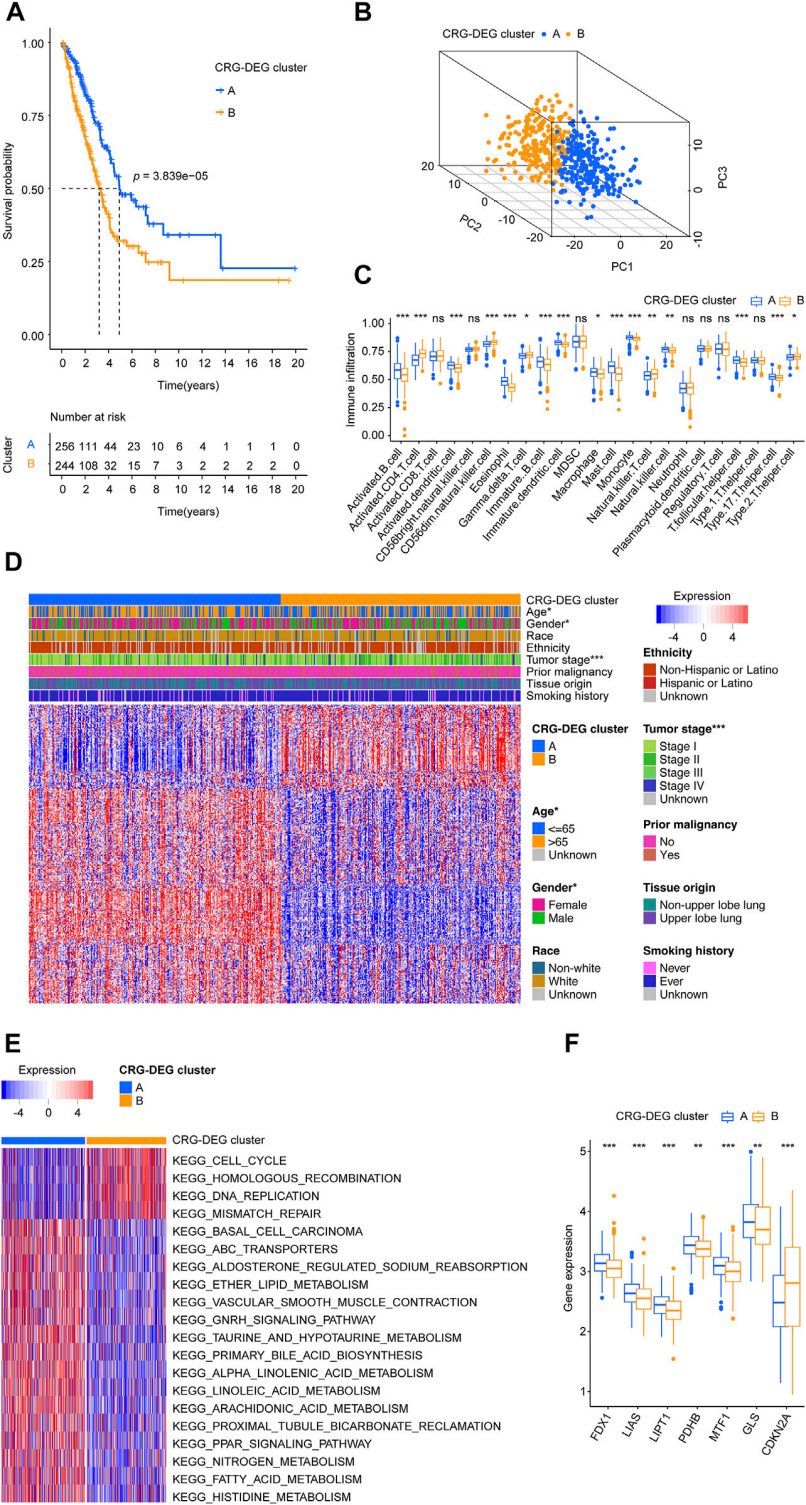
Two CRG-DEG clusters identified. **(A)** The KM survival curve demonstrated that the overall survival of patients in the CRG-DEG clusters were significantly different. **(B)** Principal component analysis scatter plot shows three clusters were obviously separated. **(C)** Infiltration distribution of immune cells in the two CRG-DEG clusters. **(D)** Heatmap shows the relationship between CRG-DEG clusters, clinical parameters, and 344 CRG-DEGs. Each row represents a gene, and each column represents a sample. **(E)** GSVA screens the most important KEGG pathways by comparing two CRG-DEG clusters. **(F)** Boxplots shows the distribution of ten CRGs (FDX1, LIAS, LIPT1, DLD, DLAT, PDHA1, PDHB, MTF1, GLS, and CDKN2A) across CRG-DEG clusters. CRG: cuproptosis-regulated gene; DEGs: differentially expressed genes; KM: Kaplan–Meier estimator; GSVA: gene set variation analysis; KEGG: Kyoto Encyclopedia of Genes and Genomes; *p-value* < 0.05 was considered statistically significant; ns: not significant; *: *p-*value < 0.05; **: *p-*value < 0.01; ***: *p-value* < 0.001.

Based on the CRG-DEGs, the LUADs from the training cohort were divided into two CRG-DEG clusters using consensus clustering methods. In the CRG-DEG clusters, the KM survival curve showed significant differences in overall survival. The cluster A had a more favor prognosis better than that of patients in cluster B (Figure 3A). The PCA revealed that the cluster A and B were obviously separated from each other (Figure 3B). Through the use of ssGSEA, we determined the degree of infiltration of the different immune cell populations in CRG-DEG clusters. As shown in Figure 3C, 16 immune cells, including Activated B cell, Activated CD4 T cell, Activated dendritic cell, CD56dim natural killer cell, Eosinophil, Gamma delta T cell, Immature B cell, Immature dendritic cell, Macrophage, Mast cell, Monocyte, Natural killer T cell, Natural killer cell, T follicular helper cell, Type 17 T helper cell, and Type 2 T helper cell were recognized significantly distributed in the CRG-DEG clusters. In addition, LUAD patients in the CRG-DEG clusters differed significantly in terms of age, gender, and tumor stage (Figure 3D). By comparing two CRG-DEG clusters, we carried out GSVA to identify KEGG pathways that are most important (Figure 3E; Supplementary Table S4). Surprisingly, KEGG_ALPHA_LINOLENIC_ACID_METABOLISM, KEGG_LINOLEIC_ACID_METABOLISM, KEGG_ARACHIDONIC_ACID_METABOLISM, KEGG_DNA_REPLICATION, KEGG_TAURINE_AND_HYPOTAURINE_METABOLISM, KEGG_CELL_CYCLE, KEGG_ABC_TRANSPORTERS, KEGG_PRIMARY_BILE_ACID_BIOSYNTHESIS, KEGG_FATTY_ACID_METABOLISM, and KEGG_ETHER_LIPID_METABOLISM ranked as the top 10 pathways. Notably, we looked at the distribution of ten CRGs (FDX1, LIAS, LIPT1, DLD, DLAT, PDHA1, PDHB, MTF1, GLS, and CDKN2A) across CRG-DEG clusters and found seven (FDX1, LIAS, LIPT1, PDHB, MTF1, GLS, and CDKN2A) were related to CRG-DEG clusters (Figure 3F). Interestingly, compared to CRG-DEG cluster A, only CDKN2A showed an upregulated situation, while the remaining six displayed downregulated.

**FIGURE 4:**
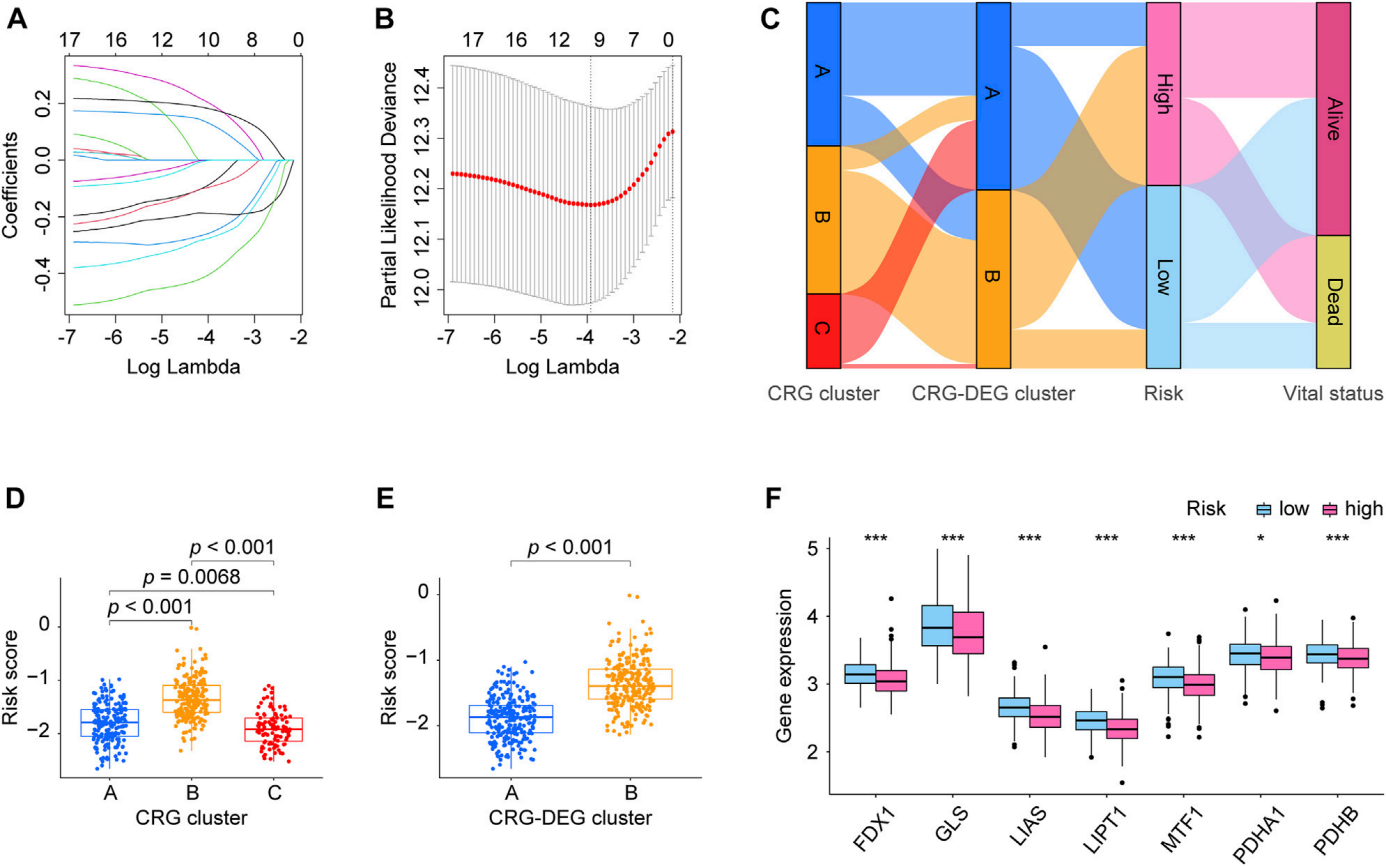
Risk model CRLncSig development. **(A)** LASSO coefficient profiles of prognostic lncRNAs enrolled. **(B)** LASSO regression with ten-fold cross-validation obtained nine prognostic genes using the minimum Lambda. **(C)** Sankey diagrams describes the relationship of CRG cluster, CRG-DEG cluster, risk, and vital status. **(D)** Boxplots shows the distribution of risk score within the CRG cluster was significantly different between each two clusters. **(E)** Boxplots displays the distribution of risk score was significantly different within the CRG-DEG cluster. **(F)** Boxplots exhibits expression pattern of the ten CRGs (FDX1, LIAS, LIPT1, DLD, DLAT, PDHA1, PDHB, MTF1, GLS, and CDKN2A) in the high- and low-risk groups. Seven genes (FDX1, LIAS, LIPT1, PDHA1, PDHB, MTF1, and GLS) were found having downregulated expression values in the high-risk group than that in the low-risk group, which statistics significantly. CRLncSig: cuproptosis-regulated lncRNA signature; LASSO: least absolute shrinkage and selection operator; FDR: false discovery rate. CRG: cuproptosis-regulated gene; DEGs: differentially expressed genes; KM: Kaplan–Meier estimator; *p-value* < 0.05 was considered statistically significant; *: *p-*value < 0.05; ***: *p-value* < 0.001.

For constructing the prognosis model of the LUAD, we first investigated the DEL between the two CRG-DEG clusters, finding there were 3,711 DELs between them. We take these DELs into KM and Cox analysis to further screen and found 17 DELs meet our criteria (Supplementary Table S5). Then the LASSO analysis was carried out using the 17 DELs for in-depth shrinkage and selection. Finally, 9 lncRNAs were identified (Figures 4A, B), and each gene’s coefficient was obtained (Table 2). In addition, we built Sankey diagrams to descript the relationship of CRG cluster, CRG-DEG cluster, risk, and vital status (Figure 4C). Using boxplots, we found that the distribution of risk scores within CRG cluster was significantly different. Consistently, the distribution of risk scores also differed significantly across the CRG-DEG clusters (Figures 4D, E). We next examined the expression pattern of the ten CRGs (FDX1, LIAS, LIPT1, DLD, DLAT, PDHA1, PDHB, MTF1, GLS, and CDKN2A) in the high- and low-risk groups. In the high-risk group, seven genes (FDX1, LIAS, LIPT1, PDHA1, PDHB, MTF1, and GLS) had downregulated expression values, which statistically significant (Figure 4F). In addition, we displayed the correlations between each of the nine lncRNAs and the ten CRGs, which are shown in Supplementary Figure S1A.

**TABLE 2:** Prognostic LncRNAs obtained from LASSO Cox regression model.

Gene symbol	Coefficient
AC009120.2	−0.080648545
AC093010.2	−0.254299619
AC107464.3	−0.27252566
COLCA1	−0.188492674
LINC00324	−0.397752717
LINC00862	0.124863725
LINC01711	0.195720949
LINC01833	0.1791624
PRKAG2-AS1	−0.093233879

### Independent cohort validation results confirmed the CRLncSig has stable prognostic power

**FIGURE 5:**
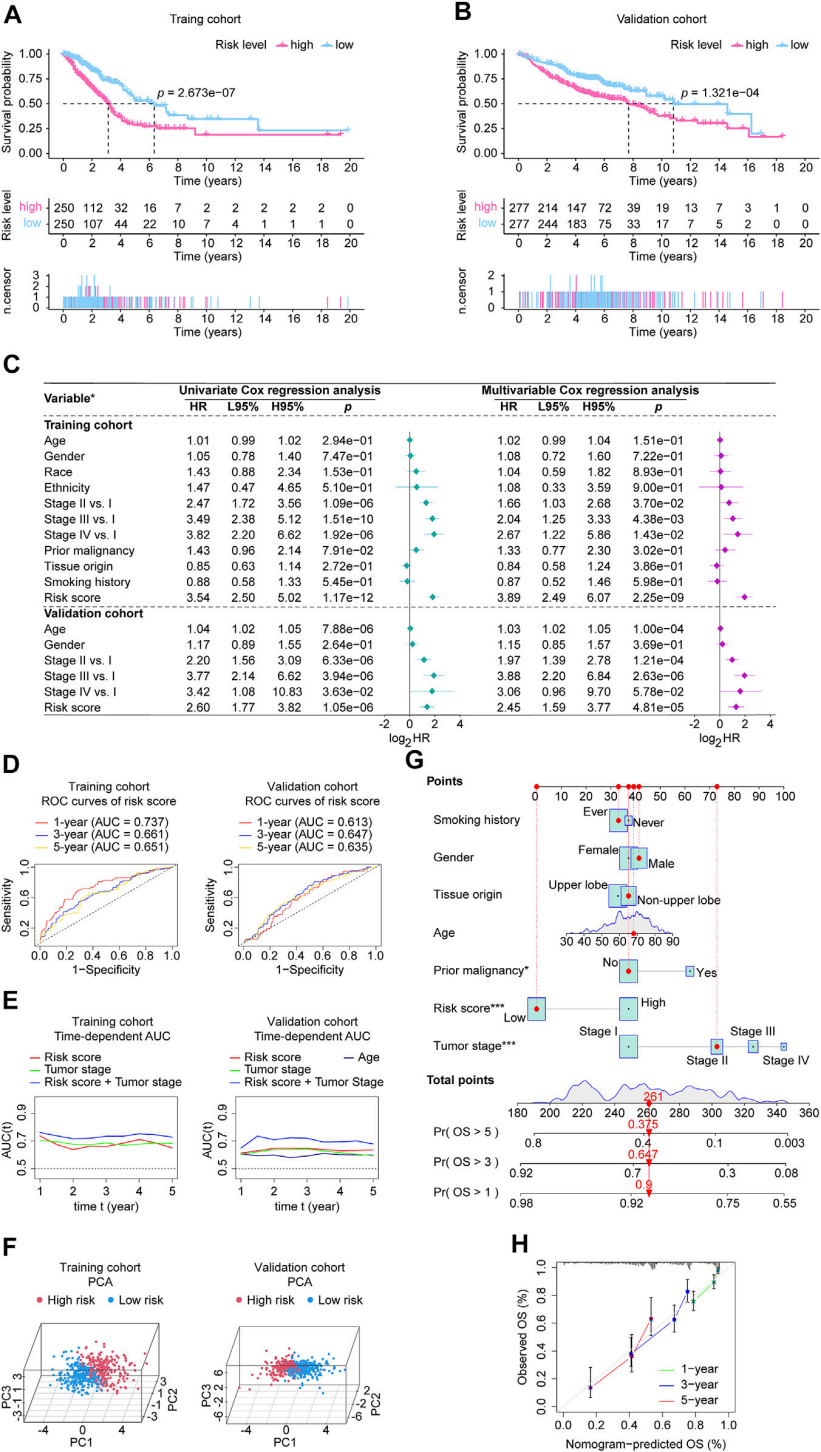
Validation of the CRLncSig in the involved studied cohorts. **(A, B)** Kaplan-Meier analysis was performed in the training and validation cohorts. Patients in each cohort and subtypes were divided into low-risk groups and high-risk groups based on their median risk score. The log-rank test with a *p-value* < 0.05 suggests the survival difference is significant. **(C)** Univariate and multivariable Cox proportional hazards models. *: the variables involved in the studied cohorts, explains as follows: Gender: male vs. female; Race: white vs. non-white; Ethnicity: Hispanic or Latino vs. non-Hispanic or Latino; Prior malignancy: yes vs. no; Tumor origin: upper lobe lung vs. non-upper lobe lung; Smoking history: ever vs. never. **(D)** ROC curves. The ROC curves value the accuracy for ​LUAD outcome prediction of our signature at 1-, 3-, and 5-year, respectively. **(E)** The tAUC analyses compare our signature’s prognostic ability with other available clinical characteristics. The larger the AUC, the stronger the model’s predictive ability. **(F)** Principal component analysis scatter plot. **(G)** The nomogram, a quantitative model for predicting clinical prognosis, to predict 1-year, 3-year, and 5-year OS in the LUAD patients of the TCGA-LUAD cohort using seven factors, including age, grade, tumor stage, risk score, smoking history, tissue origin, and prior malignancy. *: *p-*value < 0.05; ***: *p-value* < 0.001. **(H)** The calibration curves indicates that the nomogram accurately predicted the 1-, 3-, and 5-year OS of LUAD patients in the TCGA cohort. CRLncSig, cuproptosis-regulated lncRNA signature; HR, hazard ratio; L95%, 95% confidence interval lower; H95%, 95% confidence interval higher; HR, hazard ratio; ROC, receiver operating characteristic; AUC, area under the ROC curve; tAUC, time-dependent AUC; TCGA, The Cancer Genome Atlas; LUAD, lung adenocarcinoma; PCA, Principal components analysis; OS, overall survival; Exp, relative expression; *p-value* < 0.05 was considered statistically significant.

In Supplementary Figures S1B, C, the upper parts showed the patients sorted by increasing risk score, the scatter plot in the middle showed the survival status of the LUADs, and the heatmap in the lower part showed the relative expression levels of nine hub lncRNAs. Each LUAD was assigned a risk score based on the risk score calculator we developed. Patients were categorized into high-risk and low-risk groups according to their median scores. Figure 5A shows the Kaplan-Meier estimator suggested that LUADs at high risk had poorer survival prospects than LUADs at low risk. Additionally, the results of the validation cohort (Figure 5B) indicate that LUAD patients who fall into high-risk groups have a less favorable prognosis. In addition, in Supplementary Figure S2A, we displayed each nine lncRNA’s prognosis ability in the form of Kaplan-Meier curves using the two cohorts’ data, showing that the LINC0183, LINC01711, and LINC00862 performed stable unfavorable impact on LUAD patients, while AC107464.3, LINC00324, AC009120.2, COLCA1, PRKAG2-AS1, and AC093010.2 helped the prognosis improvement of LUADs. Next, we identified whether the risk score can be adopted to independently predict the outcomes of LUAD patients. The risk score and clinical parameters, including age, gender, race, ethnicity, tumor stage, tumor origin, etc. were included in univariate and multivariable analysis (Figure 5C). In the univariate Cox regression, the risk score showed significant prognostic ability. In the multivariate Cox analysis, it was recognized as an independent prognostic factor with a hazard ratio of 3.89 (95% CI: 2.49–6.07, *p* = 2.25e-09) in the training cohort, 2.45 (95% CI: 1.59–3.77, *p* = 4.81e-05) in the validation cohort. In addition, the age of validation cohort also showed independent prognostic value, however, its significance did not show a consistence in the two cohorts. Moreover, each gene’s univariate Cox regression was shown in the chart exhibited in Supplementary Figure S2B. We then tested our lncRNA signature’s prognostic ability using ROC analysis (Figure 5D) and time-dependent AUC (Figure 5E). The CRLncSig AUC in the training cohort was 0.737 at 1-year, 0.661 at 3-year, and 0.651 at 5-year, while in the validation cohort was 0.613 at 1-year, 0.647 at 3-year, and 0.635 at 5-year. According to the time-dependent AUC performed in the training cohort (Figure 5E), our risk score was close compared to tumor stage, which is regarded as the prognosis gold standard. Interestingly, when we combined the risk score and tumor stage, their predictive ability AUC could generally reach more than 0.7. Consistently, when we took the risk score and tumor stage combined in the validation cohort (Figure 5E), the predictive ability was stable beyond all factors at all time points, hinting that our risk score is an excellent addition to the tumor stage. As a result of PCA results (Figure 5F), significant heterogeneity was observed between high-risk and low-risk patients in training and validation cohorts, which demonstrated that the risk score model was superior at discriminating between these groups. Additionally, a nomogram using seven factors, including age, grade, tumor, etc., was constructed to predict 1-, 3-, and 5-year overall survival in the TCGA-LUAD cohort (Figure 5G). According to the calibration curves, the nomogram accurately predicted the 1-, 3-, and 5-year overall survival of LUAD patients in the TCGA cohort (Figure 5H).

### The CRLncSig’s relationships with apoptosis, necroptosis, pyroptosis, and ferroptosis

We got apoptosis, necroptosis, pyroptosis, and ferroptosis genes following our criteria, shown in Supplementary Table S6. The Pearson coefficient examined the relationships between our prognosis model and apoptosis, necroptosis, pyroptosis, and ferroptosis -related genes, respectively (Supplementary Table S6; Supplementary Figure S3). The analysis showed that ANLN, SELENBP1, TPX2, METTL7A, CDC20, CENPA, CAPN3, TUBA1C, CEP55, and CDC6 were the top ten correlated apoptosis-related genes, and overall, 2,469/3,681 (67.07%) genes significantly linked with the CRLncSig. CYLD, RIPK3, FADD, TLR3, TRPM7, ZBP1, IPMK, FAS, TNF, and FASLG were discovered as the top necroptosis-related that correlated with the lncRNA signature. As a whole, there were 13/20 (65.00%) necroptosis-related genes correlated with the signature pronouncedly. Moreover, the Pearson test found the top pyroptosis-related genes that correlated with our signature are NLRP1, CARD8, CYCS, CASP1, IRF2, NLRC4, NLRP3, GSDMB, NAIP, and GSDMD. In total, 35/50 (70.00%) pyroptosis-related genes correlated with our signature. The examination found RRM2, SLC2A1, AURKA, DUOX1, CDCA3, IL33, ACADSB, EPT1, SLC7A5, and HILPDA were top ferroptosis-related genes that correlated with our signature. To sum up, there were 238/380 (62.63%) ferroptosis-related genes correlating with our signature.

### CRLncSig’s mechanisms were identified by GSEA

**FIGURE 6:**
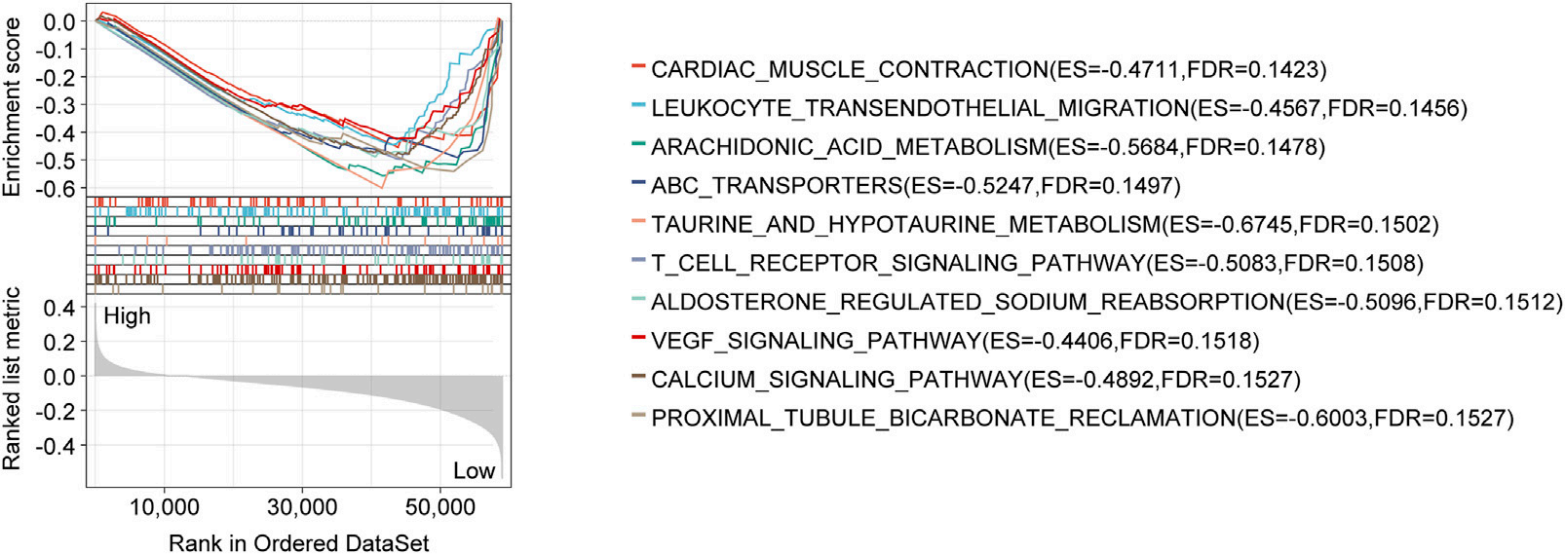
GSEA analysis with the KEGG gene set as the background identified relevant pathways of the CRLncSig. The significance threshold of this analysis was set as: *p-value* < 0.05, and *FDR* < 0.25. GSEA: Gene Set Enrichment Analysis; KEGG: Kyoto Encyclopedia of Genes and Genomes; FDR: false discovery rate.

As a result of the GSEA analysis based on risk scores, significantly enriched gene sets were identified in the CRLncSig, which the top ten KEGG items were primarily related to cardiac muscle contraction, leukocyte transendothelial migration, arachidonic acid metabolism, ATP-binding cassette transporter, taurine and hypotaurine metabolism, T cell receptor signaling, aldosterone-regulated sodium reabsorption, VEGF signaling pathway, calcium signaling, Proximal tubule bicarbonate reclamation (Figure 6).

### CRLncSig’s potential links to the LUADs immunological status

**FIGURE 7:**
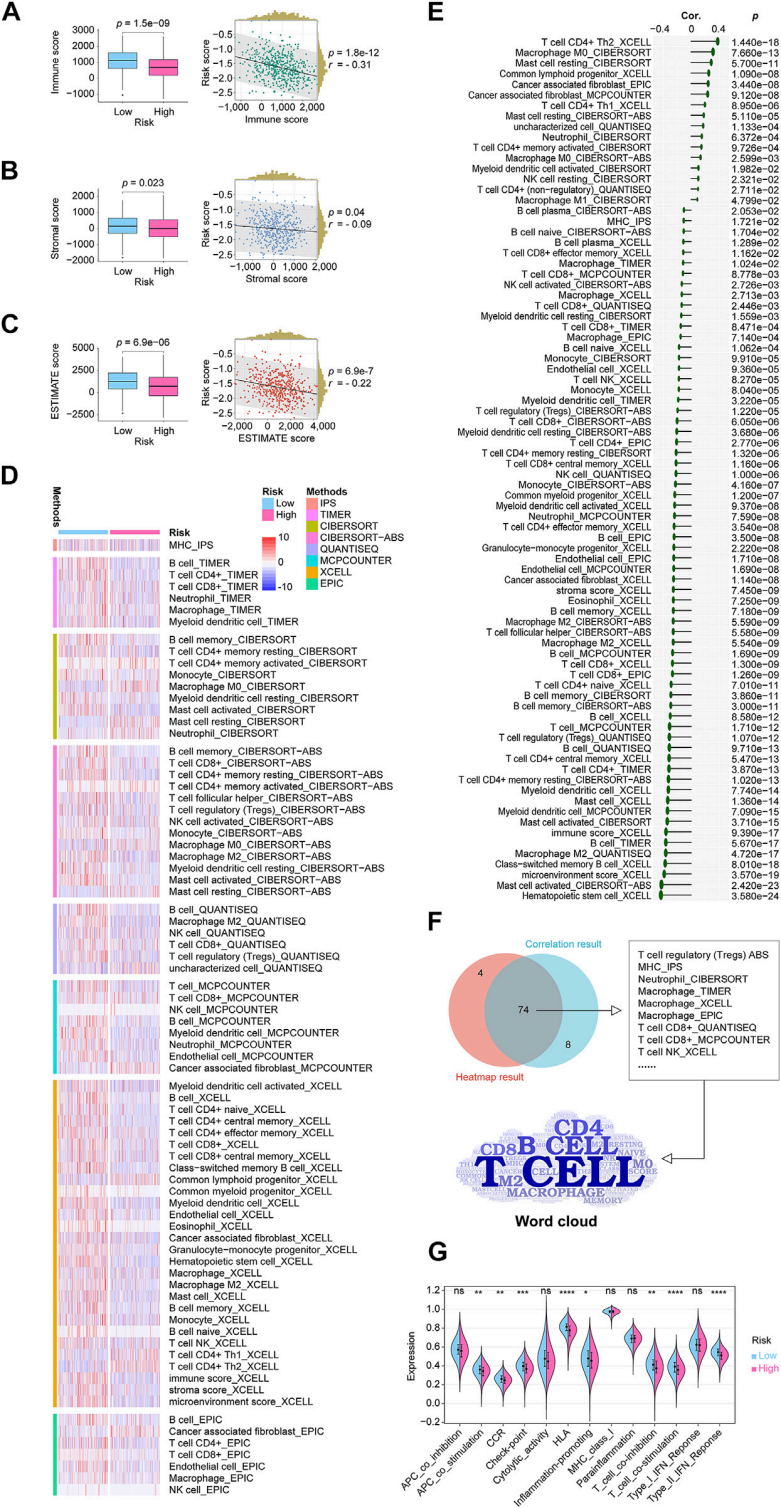
In-depth analytics on the relationship between the CRLncSig and the tumor microenvironment condition, immune cell infiltration, immune function. **(A)** A boxplot and a scatterplot display the relationship between the CRLncSig and immune score. **(B)** A boxplot and a scatterplot display the relationship between the CRLncSig and stromal score. **(C)** A boxplot and a scatterplot display the relationship between the CRLncSig and ESTIMATE score. **(D)** The heatmap demonstrated the infiltration distribution of eight types of immune cells generated from eight mainstream immunoinformatic algorithms in different CRLncSig scores. Only significantly distributed immune cells are exhibited. **(E)** The lollipop plot describes the infiltration correlations of eight types of immune cells with CRLncSig scores. Only significantly immune cells are shown. **(F)** The Venn diagram shows the intersection of the results from the heatmap and the lollipop plots, detailed showing that the CD4 T cell, B cell, CD8 T cell, and macrophage were the top cells that our risk correlated in the outline of the word cloud. **(G)** The violin plot shows the immune functions distributions in the high- and low-risk groups. CRLncSig, cuproptosis-regulated lncRNA signature; *p-value* < 0.05 was considered statistically significant; ns, not significant; *p-value < 0.05; **p-value < 0.01; ***p-value < 0.001; ****p-value < 0.0001.

There is widespread acceptance that cancer is a dynamic ecosystem in which subclonal populations, mainly cancer cells and non-malignant cells in the tumor microenvironment, cooperate to promote disease progression. A general examination of the tumor microenvironment is therefore necessary. Here, using data from the TCGA cohort, we quantify the scores, including immune score, stromal score, and ESTIMATE score, using the R package “ESTIMATE”. Then through the analysis of the Wilcoxon rank sum test and Pearson correlation coefficient test, it was found that all the scores of categories were lower in the high-risk group, and all scores were negatively correlated with the CRLncSig (Figures 7A–C). Eight mainstream immunoinformatic algorithms, including IPS, TIMER, CIBERSORT, CIBERSORT−ABS, QUANTISEQ, MCPCOUNTER, XCELL, and EPIC, were integrated into the process of screening immune cell types involved in tumor infiltration in high- and low-risk groups. These processes were carried out with Wilcoxon rank-sum test and Pearson correlation coefficient test and displayed in the shapes of the heatmap (Figure 7D) and lollipop (Figure 7E) plots, respectively. In the plots, only significant factors were outlined, and much detail was shown in Supplementary Table S7. The Venn diagram shown in Figure 7F demonstrated the intersection of the results from the heatmap and the lollipop plots, detailed showing that the CD4 T cell, B cell, CD8 T cell, and macrophage were the top cells that our risk correlated in the outline of the word cloud. As for the related immune functions, the scores for the APC_co_stimulation, CCR, Check−point, HLA, Inflammation−promoting, T_cell_co−inhibition, T_cell_co−stimulation, and Type_II_IFN_Reponse were lower in the high-risk group than in the low-risk group (Figure 7G). These findings revealed that the lncRNA signature might be associated with the immunological status of LUADs.

### The CRLncSig’s participation in immunotherapy and targeting potential immune checkpoint

**FIGURE 8:**
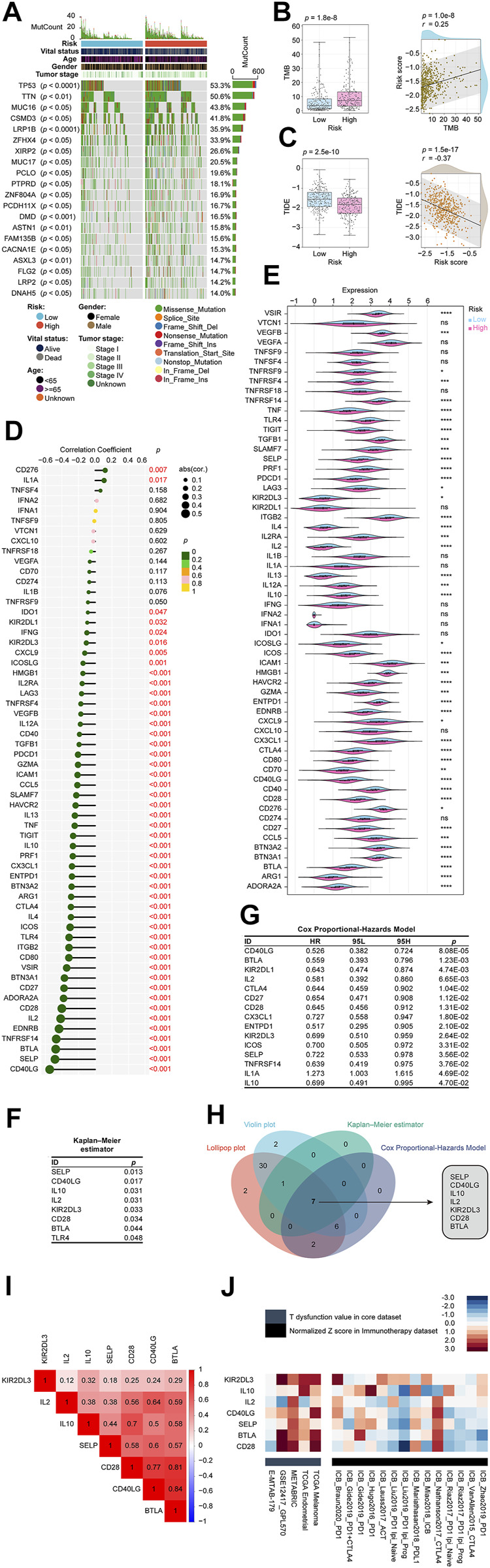
Determination of the relationship between the CRLncSig and immunotherapy. **(A)** The waterfall plot shows the top 20 genes mutated in the LUAD, and their difference in the high and low risk groups. **(B)** The TMB difference in the high and low-risk patients tested by the Wilcoxon rank-sum showing in form of boxplot. The correlation between TMB and the signature tested by the Pearson coefficient showing in form of scatterplot. **(C)** The TIDE difference in the high and low-risk patients tested by the Wilcoxon rank-sum showing in form of boxplot. The correlation between TIDE and the signature tested by the Pearson coefficient showing in form of scatterplot. **(D)** The lollipop plot describes the correlations between CRLncSig and immune checkpoints explained by the Pearson coefficient. **(E)** The violin plot shows the distribution of immune checkpoints in low and high- CRLncSig score tested by the Wilcoxon rank-sum. **(F)** The Kaplan–Meier estimator found that 8 checkpoints affected the prognosis of LUAD, namely SELP, CD40LG, IL10, IL2, KIR2DL3, CD28, BTLA, and TLR4. **(G)** The Cox Proportional-Hazards Model revealed 15 prognosis-related genes among 60 checkpoints. **(H)** The Venn diagram shows the intersection of the above results and found that SELP, CD40LG, IL10, IL2, KIR2DL3, CD28, and BTLA not only closely related to our CRLncSig, but also affected the LUAD prognosis. **(I)** The correlations between seven found checkpoint genes are shown as a heatmap and performed *via* the Pearson coefficient. All displayed correlations were statistically significant. **(J)** Heatmap exhibits these seven checkpoint genes’ roles in the immune system and immunotherapy from multiple immune datasets. In the immunotherapy cohorts (black module), the seven checkpoints were ranked descendingly based on the average score of a group with the immunotherapy cohorts, and they were ranked from high to low as KIR2DL3, IL10, IL2, CD40LG, SELP, BTLA, and CD28. CRLncSig: cuproptosis-regulated lncRNA signature; ns: not significant; *p-value < 0.05; **p-value < 0.01; ***p-value < 0.001; ****p-value < 0.0001; *p-value* < 0.05 was considered statistically significant; TMB, Tumor mutational burden; TIDE, Tumor Immune Dysfunction and Exclusion.

We thoroughly explored the mutation characteristics of all tumor samples in TCGA-LUAD cohort. Only genes with significant mutation differences between low- and high-risk groups (*p* < 0.05) were selected and were arranged according to mutation frequency. As shown in the Figure 8A, we listed the top 20 mutated genes showing TP53 mutated most frequently approximately accounting for 53.3% in the cohort, followed by TTN (50.6%) and MUC16 (43.8%). Among the alterations, missense mutation was the most common variant classification. Wilcoxon tests revealed that groups with higher risk scores had higher TMB than groups with lower risk scores. Among risk scores and TMB, Pearson coefficients showed a positive correlation (Figure 8B). According to clinical trials and preclinical studies, the immune checkpoint blockade offers more durable clinical benefits, including treatment responses and long-term survival, to patients with higher TMB (Samstein et al., 2019; Stenzinger et al., 2019). Our results here demonstrated that our high risk LUADs might expect more responses from immunotherapy to a certain extent. Following that, we examined the potential clinical efficacy of immunotherapy based on the risk score subgroups using the TIDE. As a surrogate biomarker, TIDE scores can provide insight into whether a NSCLC patient will respond to immune checkpoint blockades, including anti-PD1 and anti-CTLA4, if therapy is initiated. Immune evasion is more likely to occur in patients with higher TIDE prediction scores, suggesting that immunotherapy has a lesser chance of being effective (Jiang et al., 2018; Fu et al., 2020; Chen et al., 2021a). Based on our results, high-risk patients had lower TIDE scores, meaning that immunotherapy was more beneficial for them (Figure 8C), which corresponded with our “TMB difference” findings above.

We curated 60 immune checkpoint genes based on past studies, and we found that 48 of the 60 genes were significantly associated with risk score using the Pearson correlation coefficient (Figure 8D). The top 5 were CD40LG (coefficient = −0.55), SELP (coefficient = −0.48), BTLA (coefficient = −0.48), TNFRSF14 (coefficient = −0.47), and EDNRB (coefficient = −0.44) (Figure 8D). As shown in Figure 8E, 46 checkpoint genes were differentially distributed between high-risk and low-risk groups based on the Wilcoxon signed-rank test. The Kaplan–Meier estimator found that 8 checkpoints affected the prognosis of LUAD, namely, SELP, CD40LG, IL10, IL2, KIR2DL3, CD28, BTLA, and TLR4 (Figure 8F). The Cox Proportional-Hazards Model revealed 15 prognosis-related genes among 60 checkpoints (Figure 8G). In order to simplify the results and balance the outputs of each analysis, we used Venn diagrams to intersect the above results and found that SELP, CD40LG, IL10, IL2, KIR2DL3, CD28, and BTLA not only closely related to our CRLncSig, but also affected the LUAD prognosis, which deserves more attention (Figure 8H). Interestingly, the relationships between each two of the seven checkpoints were calculated, showing all of them have positive correlations and are statistically significant (Figure 8I).

To further evaluate the potential impact of these seven checkpoint genes on the immune system and immunotherapy, we collected data from multiple immune datasets and presented them in a heatmap format (Figure 8J). It can be seen that these seven checkpoint genes exhibited higher T dysfunction values in the GSE12417_GPL570, METABRIC, TCGA Endometrial, and TCGA Melanoma cohorts than in the E-MTAB-179 cohort (grey module). In the immunotherapy cohorts (black module), the seven checkpoints were ranked descendingly based on the average score of a group with the immunotherapy cohorts, and they were ranked from high to low as KIR2DL3, IL10, IL2, CD40LG, SELP, BTLA, and CD28. These findings potentially hinted at the direction of future crosstalk research between our CRLncSig and immunotherapy.

### Identification and validation of potential therapeutic agents for high risk score LUADs

**FIGURE 9:**
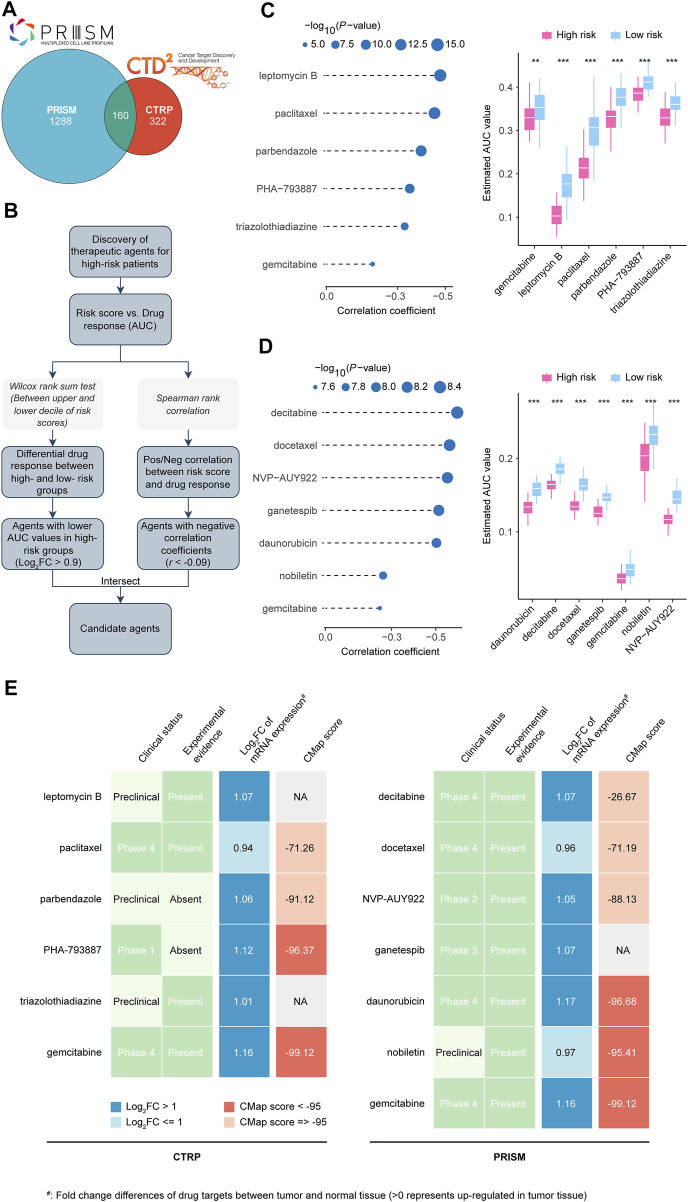
Identification of candidate agents with higher drug sensitivity in high CRLncSig risk score patients. **(A)** A venn diagram for summarizing included compounds from CTRP and PRISM datasets. **(B)** Schematic outlining the strategy to identify agents with higher drug sensitivity in high risk score patients. **(C)** The results of Spearman’s correlation analysis and differential drug response analysis of six CTRP-derived compounds. The lower the value of the *y*-axis, the greater the drug sensitivity. **(D)** The results of Spearman’s correlation analysis and differential drug response analysis of seven PRISM-derived compounds. The lower the value of the *y*-axis, the greater the drug sensitivity. **(E)** Identification of most promising therapeutic agents for high risk score patients according to the evidence from multiple sources). Six CTRP-derived agents and seven PRISM-derived agents were shown on the left and right of the diagram, respectively. CRLncSig, cuproptosis-regulated lncRNA signature; Pos/Neg, Positively/Negatively; **p-value < 0.01; ***p-value < 0.001; *p-value* < 0.05 was considered statistically significant.

CTRP and PRISM datasets contain gene expression profiles and drug sensitivity profiles of hundreds of CCLs, which can be used to build a drug response prediction model. There were 160 compounds shared between the two datasets. In total, 1770 compounds were found in the two datasets after removing duplication (Figure 9A; Supplementary Table S8). We identified candidate agents with greater drug sensitivity for patients with high-risk scores using two different approaches (Figure 9B). Based on CTRP and PRISM data, the analyses were conducted. The first step was to identify compounds with lower estimated AUC values in the high risk score group by performing a differential drug response analysis (log2FC > 0.9) between the highest and lowest risk score decile groups. Following that, having examined the correlation between AUC values and risk scores, compounds with a negative Spearman coefficient of 0.09 were selected. These analyses yielded six CTRP-derived compounds (including leptomycin B, paclitaxel, parbendazole, PHA−793887, triazolothiadiazine, and gemcitabine) (Figure 9C) and seven PRISM-derived compounds (including decitabine, docetaxel, NVP−AUY922, ganetespib, daunorubicin, nobiletin, and gemcitabine) (Figure 9D). AUC values for all of these compounds were lower in the risk score-high group, and there was a negative correlation between risk scores and AUC values.

In spite of the 13 candidate compounds showing a higher drug sensitivity in high-risk patients, the above analyses alone cannot support the conclusion that these compounds are good enough. Further multi-angle analysis was carried out in order to assess their therapeutic potential in LUADs. First, we used CMap analysis to identify compounds whose gene expression patterns were opposed to the LUAD-specific expression patterns (i.e., gene expression increased in tumor tissues but decreased by treatment of certain compounds). Four compounds, including PHA-793887, gemcitabine, daunorubicin, and nobiletin, had CMap scores <−95, representing that these compounds might have potential therapeutic effects in LUADs (Figure 9E; Supplementary Table S9). Second, we calculated the fold-change value representing the difference between the candidate drug target in tumor and normal tissues. An increased fold-change value indicates that the candidate drug is more likely to be effective at treating LUAD (Figure 9E; Supplementary Table S9). The third step was to conduct a comprehensive literature review in PubMed (https://www.ncbi.nlm.nih. gov/pubmed/) to determine the experimental and clinical evidence that candidate compounds are beneficial in treating LUAD (Figure 9E; Supplementary Table S9) (Burkes and Shepherd, 1995; Fossella, 2002; Ramalingam and Belani, 2004; Shimamura et al., 2012; Garon et al., 2013; Momparler, 2013; Liu and Gao, 2017; Wang et al., 2019; Han et al., 2021; Sever et al., 2021). On a whole, gemcitabine, daunorubicin, and nobiletin are considered promising for treating high risk score LUADs based on our robust *in silico* evidence and confirmed *in vitro* findings. In contrast, PHA-793887, which showed an excellent CMap score but lacked vitro experiment validation, also deserved attention in further research.

### Confirming the nine lncRNAs expression patterns in human tissues using real-time PCR and their potential in pan-cancer

**FIGURE 10:**
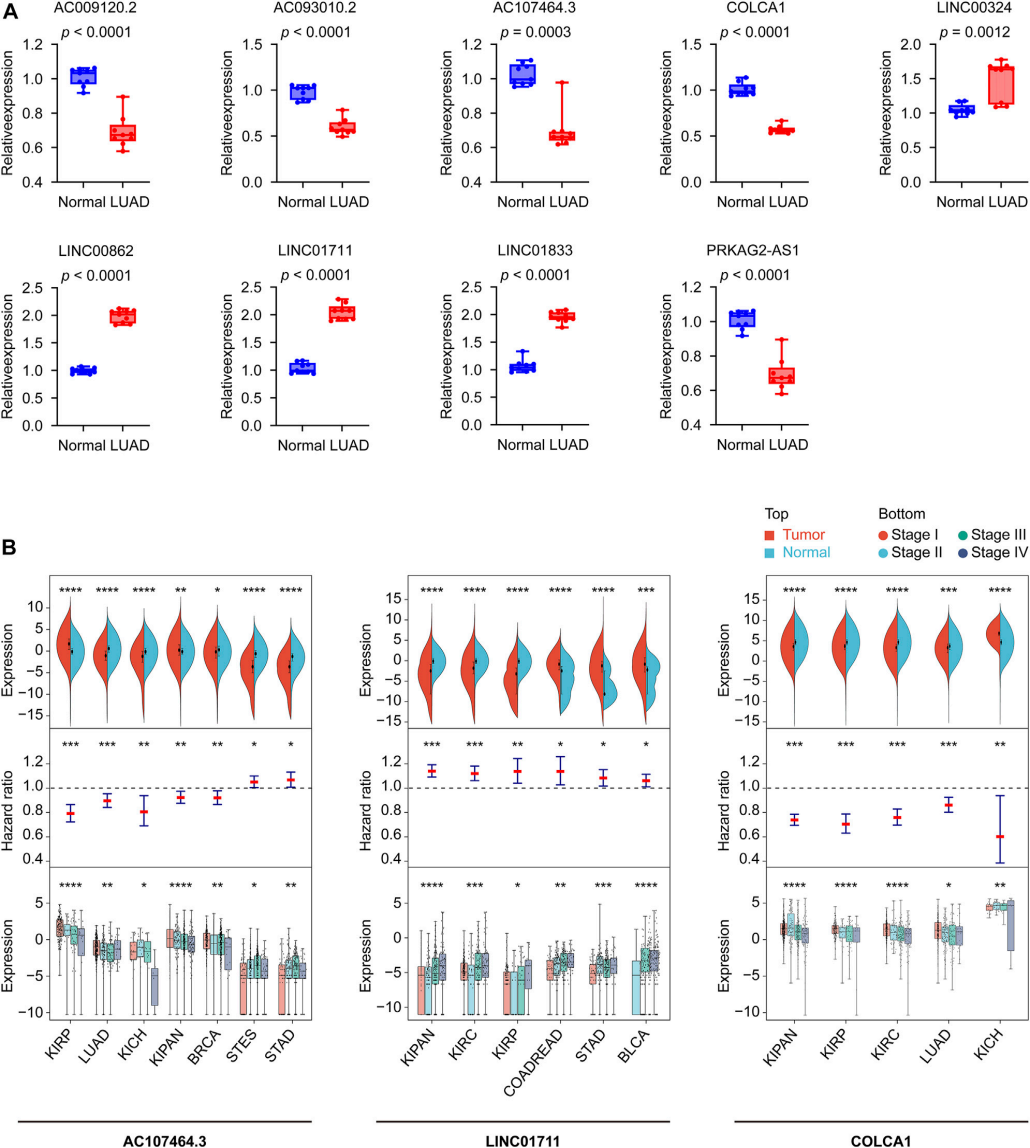
Confirming the CRLncSig’s nine lncRNAs expression patterns in human tissues using real-time PCR and their potential in pan-cancer. **(A)** The expression levels of the 9 signature lncRNAs in normal lung (*n* = 9) and LUAD (*n* = 9) tissues detected by real-time PCR. Data were means ± standard deviation. **(B)** The diagrams show the ability of AC107464.3, LINC01711, and COLCA1 in tumor and normal differential expression, prognosis, and tumor staging. CRLncSig: cuproptosis-regulated lncRNA signature; *p-value < 0.05; **p-value < 0.01; ***p-value < 0.001; ****p-value < 0.0001; *p-value* < 0.05 was considered statistically significant; BLCA, Bladder Urothelial Carcinoma; BRCA, Breast invasive carcinoma; COADREAD, Colon adenocarcinoma/Rectum adenocarcinoma Esophageal carcinoma; KICH, Kidney Chromophobe; KIPAN, Pan-kidney cohort; KIRC, Kidney renal clear cell carcinoma; KIRP, Kidney renal papillary cell carcinoma; LUAD, Lung adenocarcinoma; STAD, Stomach adenocarcinoma; STES, Stomach and Esophageal carcinoma.

To better understand the real-world expression pattern of each gene in the gene signature, we applied the real-time PCR to detect the above lncRNAs in human LUAD tissues (*n* = 9) and human normal lung tissues (*n* = 9) difference in expression. Table 3 shows the primer sequences for the nine lncRNAs, AC009120.2, AC093010.2, AC107464.3, COLCA1, LINC00324, LINC00862, LINC01711, LINC01833, and PRKAG2-AS1. Notably, all lncRNAs had different expression in LUAD and normal lung tissues (Figure 10A). It was found that LINC00324, LINC00862, LINC01711, and LINC01833 lncRNAs were upregulated in LUAD tissues, while the remaining lncRNAs were underexpressed. The upregulated genes in LUAD like LINC00862, LINC01711, and LINC01833 were also showed having unfavorable prognosis powerful in Supplementary Figure S2, and the downregulated genes in LUAD like AC009120.2, AC093010.2, AC107464.3, COLCA1, and PRKAG2-AS1 displayed owning protectable function for the LUAD outcomes, which further proved the validity of the gene signatures we found and provided clues for upcoming deeper research.

**TABLE 3:** The nine lncRNAs’ primer sequences and their potential in pan-cancer.

Gene symbol	Sequence (5′–3′)		Pan-cancer potential				Cancer types with significant differential expression, prognostic ability, and staging power	
	Forward	Reverse	Significantly differentially expressed between tumor and normal, cancer type counts (total 34 types)	Significant prognostic ability, cancer type counts (total 44 types)	Significantly distributed in tumor stage subtypes, cancer type counts (total 30 types)	Sum counts	Cancer type counts	Cancer type names
AC009120.2	CCC​TGT​TGG​CAG​AGG​TGT​AT	GGG​TGC​AGA​GAC​CAG​GAA​TA	31	12	7	50	2	COAD, LUAD
AC093010.2	GTG​AGG​TTC​GAA​GCA​GGA​AG	TTC​CCA​GTA​TGG​CGT​TTC​TC	19	10	3	32	2	LUAD, KIPAN
AC107464.3	CCT​GGG​GAT​GCA​GCA​TAT​T	GGC​AAG​AGA​GAC​CAG​CAT​TC	30	17	8	55	7	KIRP, LUAD, KICH, KIPAN, BRCA, STES, STAD
COLCA1	ATC​TTC​ACC​CCA​AGC​CTT​CT	CTG​AGG​TCA​ATG​GCA​AGG​AT	30	10	8	48	5	KIPAN, KIRP, KIRC, LUAD, KICH
LINC00324	AGA​GCC​CAG​GAA​CTG​TCA​AA	GGG​TTC​TGT​TCT​TCC​AAC​CA	24	14	8	46	3	LIHC, LUAD, KIRP
LINC00862	GCA​GCG​ATT​GGA​GTG​ATG​TA	CAG​AAG​TCC​CAA​GTC​CCA​AA	25	14	4	43	1	KICH
LINC01711	ACT​CTC​CGA​GGG​TCA​AGG​AT	GCC​TTT​GAG​TAA​GCC​GTT​TG	30	16	12	58	6	KIPAN, KIRC, KIRP, COADREAD, STAD, BLCA
LINC01833	ACC​ATC​GGA​CTG​ACG​TTC​TC	CTA​GAA​GCG​GTT​CCT​TGT​GC	31	9	3	43	1	KICH
PRKAG2-AS1	GGG​ACT​TCT​GGG​TCT​TCT​CC	CTT​GAG​CAT​TCA​GTG​GGA​CA	31	9	8	48	2	KIPAN, BRCA

Using pan-cancer expression patterns as a starting point, we assessed the potential of these nine lncRNAs. We analyzed the expression data of 34 cancer types, finding six of the nine lncRNAs, AC009120.2, LINC01833, PRKAG2-AS1, AC107464.3, COLCA1, and LINC01711, showed significant differences (tumor vs. normal) in 30 or more cancer types (Supplementary Figure S4; Table 3). Taking a step further, we then explored the prognostic ability of nine lncRNAs in pan-cancer. For this reason, we curated 44 cancer types according to the screening criteria and built the Cox models. The results displayed that the top five lncRNAs were AC107464.3, LINC01711, LINC00862, LINC00324, and AC009120.2; they affected 17, 16, 14, 14, and 12 cancer types, respectively (Supplementary Figure S5; Table 3). Finally, we tested the expression distribution of nine lncRNAs in different cancer stages, and according to the criteria we set, we found data from 30 cancer types for this analysis. The results showed that LINC01711, AC107464.3, LINC00324, COLCA1, and PRKAG2-AS1 were the top five lncRNAs, and they were differentially expressed in cancer stages of 12, 8, 8, 8, and 8 cancer types, respectively (Supplementary Figure S6; Table 3). According to our above results, we tried to find which lncRNA was significant in tumor and normal differential expression, prognostic ability, and tumor staging power and found that AC107464.3, LINC01711, and COLCA1 occupied the top 3 most counts of cancer types, which was 7, 6, and 5, respectively **(** Figure 10B; Table 3). Our brief exploration of the nine lncRNAs and pan-cancer prove the importance of our CRLncSig, which may shed light on future research in other cancers.

## Discussion

In the past few years, copper has become increasingly recognized as an essential mineral nutrient for cell proliferation and death due to its intrinsic redox properties (Lelievre et al., 2020; Ge et al., 2022). This field has brought together researchers from various disciplines due to its rapid development. Including researchers could help translate basic copper chemistry and biology research into clinical applications, especially for cancer treatment (Ge et al., 2022). Interestingly, Tsvetkov found that copper can bind directly to the lipid acylation component of the tricarboxylic acid (TCA) cycle, causing it to aggregate lipid acylated proteins and iron-sulfur clusters (Tsvetkov et al., 2022). Unlike known forms of cell death such as pyroptosis, apoptosis, ferroptosis, and necroptosis, cuproptosis is completely new. Considering that the prognostic outcomes of LUAD vary widely, the development of a robust classifier is crucial to maximizing the benefits of personalized treatment and timely follow-up for patients of varying risk and prognosis. In the present research, we innovatively adopt the novel cuproptosis concept to establish CRLncSig predicting LUAD outcomes by digging TCGA and GEO databases (with a total human sample size exceeding 1,000 cases). Specifically, our novelty lay in adopting comprehensive bioinformatics analysis, including consensus clustering, LASSO regression, Kaplan-Meier curves, Cox models, ROC curves, and tAUC, and the validations in a large cohort. Our analysis revealed that the CRLncSig is potentially linked to the LUADs immunological status and participates in immunotherapy and targeting potential immune checkpoints like SELP, CD40LG, IL10, IL2, KIR2DL3, CD28, and BTLA. Above all, the *in silico* screening of 1770 compounds identified three drugs with potential therapeutic implications for high-risk LUADs, gemcitabine, daunorubicin, and nobiletin. Lastly, we describe signature lncRNAs’ real-world expression patterns using real-time PCR and their potential in pan-cancer by analyzing multi-cohort.

Many studies have revealed relevant risk parameters for its progression in malignant hepatocellular neoplasms (Moriyama et al., 2022; Park et al., 2022; Yang et al., 2022). However, the increased incidence of hepatocellular carcinoma in Wilson’s patients and in animal models has raised researchers’ concerns that abnormal copper accumulation may be related to an unidentified mechanism of tumor progression (Ge et al., 2022). Many studies have shown that tumors require a greater amount of copper than healthy tissue, leading to a link between copper and cancer (Ge et al., 2022). The balance between gastrointestinal absorption and biliary excretion is maintained for copper homeostasis once mammalian growth and development has been completed. Nevertheless, anabolic status requires significantly more copper in the diet to meet metabolic demands (Ge et al., 2022). Copper deficiency during embryonic and fetal development results in numerous structural and biochemical abnormalities (Ge et al., 2022). Since copper is necessary for mitochondrial cytochrome c oxidase, which is required for rapid cell division, cancer cells have an increased requirement for copper (Ge et al., 2022). Various cancers, including lung cancer, have been linked to elevated copper concentrations in animal models and patients (Ge et al., 2022). Copper imbalance affects mitochondrial respiration and interferes with glycolysis, insulin resistance, and lipid metabolism (Ge et al., 2022). Additionally, copper pathways, such as the ATOX-ATP7A-LOX pathway, contribute to metastatic spread (Ge et al., 2022). Aside from affecting tumor angiogenesis, copper regulates autophagy through ULK1 and ULK2, as well as promoting tumor formation, growth, and metastasis (Ge et al., 2022). As a result of this metal nutrient, copper, a number of pro-angiogenic factors are activated, including vascular endothelial growth factor, fibroblast growth factor 2, tumor necrosis factor, and interleukin 1^56^. Many recent studies link copper signaling to cancer cell proliferation, tumor growth, and metastasis (Ge et al., 2022). Copper and cancer metabolism must be studied intensively in order to understand all aspects of cancer biology, such as tumor initiation, growth, and metastasis (Ge et al., 2022). It is urgently needed to find meaningful cuproptosis-related signatures for predicting tumor prognosis, which will be useful for understanding cuproptosis’ mechanism in cancer. According to Zhang and collogues, a prognostic lncRNA profile related to cuproptosis may offer new perspectives on hepatocellular carcinoma therapy (Zhang et al., 2022b). Bian’s team constructed a cuproptosis-related gene signature as a potential prognostic predictor in patients with clear cell renal cell carcinoma, offering novel insights into cancer treatment (Bian et al., 2022). For the moment, in LUAD, there is still no study showing that any cuproptosis-related signature can predict prognosis. Our present study attempts to fill this gap and construct a cuproptosis related lncRNA signature for predicting the outcomes of LUAD, providing more clues for this “virgin land” in follow-up studies.

Our signature contains nine lncRNAs (Table 2), which were AC009120.2, AC093010.2, AC107464.3, COLCA1, LINC00324, LINC00862, LINC01711, LINC01833, and PRKAG2-AS1. Furthermore, we performed real-time PCR validation and found that all lncRNAs were significantly expressed differently between LUAD and normal human lung tissues. A negative effect was demonstrated by LINC00862, LINC01711, and LINC01833 on LUAD outcomes, while the remaining lncRNAs detected a positive impact (Supplementary Figure S2). There are rare studies on LINC00862, which were only mentioned in one recent study. According to Yu and colleagues, LINC00862 levels were elevated in HCC tumor nodules and cirrhotic livers, suggesting it might be involved in the progression of HCC (Yu et al., 2021). Moreover, these researchers also examined the expression of LINC00862 in human HCC cell lines and found that it is upregulated in several HCC cell lines in comparison to THLE-2, a normal human liver cell line (Yu et al., 2021). Elevated expression of LINC01711 in bladder cancer correlates with reduced survival (Du et al., 2021). Through its interaction with miR-326 and FSCN1, LINC01711 has been shown to promote esophageal squamous cell carcinoma initiation and progression (Xu et al., 2021). Additionally, LINC01711 expression was positively correlated with TGF-β1, a critical factor in the TGF-β signaling pathway (Lee et al., 2005). Unfortunately, the research on LINC01711 and lung cancer is still lacking, and more efforts are needed. A study showed that LINC01833 sponging miR-519e-3p and regulating S100A4 expression promote LUAD invasion (Zhang et al., 2020). Overexpression of LINC01833 improves the proliferation and invasion ability of lung cancer cells, as well as promotes the transition from epithelial to mesenchymal state (Zhang et al., 2020).

In their study, Mitra et al. (2012) pointed out that excessive copper exposure can lead to apoptosis and cell cycle arrest, potentially causing immunotoxicity, and found that copper-induced immunosuppression appears to be regulated by the apoptotic pathway. In several human diseases, including cancer, necroptosis causes caspase-independent programmed cell death mediated by the MLKL signaling cascade (Zhang et al., 2022c). A high level of inflammation results in pyroptosis, a form of lysis-programmed cell death that occurs mainly during intracellular pathogens within the cell and may form part of an antimicrobial response (Tan et al., 2021). Ferroptosis is characterized by the inactivation of GPX4, followed by the accumulation of ROS and the binding of free Fe2+ to membrane lipids (Li et al., 2022c). Copper depletion limited GPX4 protein expression, the only enzyme capable of protecting against lipid peroxidation, according to Li et al. (2022c). In the presence of copper depletion, the dermal papilla cells (DPCs) were less sensitive to erastin (an inducer of ferroptosis), while the ferroptosis inhibitor ferrostatin-1 (Fer-1) partly prevented bathocuproinedisulfonic (BCS)-induced cell death (Li et al., 2022c). While the way of regulated cell death of cuproptosis, apoptosis, necroptosis, pyroptosis, and ferroptosis varies (Chen et al., 2021b; Tsvetkov et al., 2022), from our research, they seem to be somewhat related, such as our cuproptosis-related signature correlated with 2,321/3,681 (63.05%) apoptosis-related genes, 11/20 (55.00%) necroptosis-related genes, 34/50 (68.00%) pyroptosis-related genes, and 222/380 (58.42%) ferroptosis-related genes, providing potential explanations and inspirations for further research of cell death-related tumor mechanism.

Cancer immunotherapy prolongs the survival of deadly cancer patients and as more cancer patients become eligible for immune-based cancer treatments, reflecting that this approach is revolutionizing the field of oncology (Vanneman and Dranoff, 2012; Waldman et al., 2020). New therapeutic combinations and newly discovered drug targets are expanding the use of immunotherapy in cancer treatment (Vanneman and Dranoff, 2012; Waldman et al., 2020). Targeted strategies inhibit tumor progression by interfering with crucial molecular pathways, while immunotherapy produces durable and effective tumor destruction by stimulating the host’s own response (Vanneman and Dranoff, 2012; Waldman et al., 2020). The main challenge of immunotherapy is how to determine whether a certain biomarker is suitable for the host, and how to adjust its application strategy to maximize the benefits (Suresh et al., 2018). This study gives hints about which immunotherapy targets to use or under what circumstances to apply. We first found that our risk score was associated with TMB and TIDE, suggesting that our signature appeared to guide immunotherapy. Next, we followed the trail and found seven checkpoints, including SELP, CD40LG, IL10, IL2, KIR2DL3, CD28, and BTLA elated to our signature score. In the immunotherapy cohorts we chosen, the seven checkpoints were ranked from high to low as KIR2DL3, IL10, IL2, CD40LG, SELP, BTLA, and CD28. KIR2DL3, a transmembrane glycoprotein expressed by natural killer cells and T cell subsets, is responsible for sending inhibitory signals throughout the cell (Sim et al., 2016). KIR2DL3’s structure is important for cancer development (Sim et al., 2016). Neuroblastoma development is influenced by KIR2DL3, according to Sezgin et al. (2022). IL-10 is a cytokine with potent anti-inflammatory properties. The function of IL-10 is to prevent damage to the host and maintain normal tissue homeostasis by limiting the host’s immune response to pathogens (Oft, 2014). IL-10 suppresses inflammatory Th17 T cells and macrophages, which can trigger or promote tumor formation (Oft, 2014). The research by Vahl and colleagues showed that IL-10 competes with IFN-γ on the PD1/PDL1 pathway, potentially contributing to resistance to PD1/PDL1 immunotherapy in lung cancer patients (Vahl et al., 2017). A key function of IL-2 is activating the immune system, which could potentially eradicate cancer (Jiang et al., 2016). The FDA has approved the use of IL-2 as a monotherapy for metastatic renal cell carcinoma and metastatic melanoma (Jiang et al., 2016). A decrease in IL-2 levels and a marked increase in soluble IL-2 receptor concentrations have been observed in advanced NSCLC, and these are associated with poor outcomes (Jiang et al., 2016). The role of IL-2 activation in restoring lymphocyte immunocompetence against lung cancer has also been demonstrated (Jiang et al., 2016).

Individuals with LUAD exhibit high levels of heterogeneity, making it nearly impossible to find a treatment that is effective for everyone (Zito Marino et al., 2019). All current treatments for advanced LUAD lack corresponding biomarkers, so they do not achieve satisfactory therapeutic effects because they are population-based (Zito Marino et al., 2019). One of the main goals of this study is to find a tailored treatment strategy for a specific population, which is crucial to maximizing treatment effectiveness. As well as providing information on prognosis, the CRLncSig risk score can be used in precision oncology to guide targeted therapy. In particular, our study discovered three potential therapeutic agents for high-risk LUAD patients: gemcitabine, daunorubicin, and nobiletin. Non-small cell lung cancer is commonly treated with gemcitabine, a synthetic antimetabolite tumor drug (Sederholm et al., 2005). A synthetic version of gemcitabine was developed by Larry Hertel in the early 1980s for use as an antiviral medication. However, preclinical tests showed it could kill leukemia cells *in vitro* as well (Burkes and Shepherd, 1995). Gemcitabine was approved for the treatment of non-small cell lung cancer by the FDA in 1998 (Barton-Burke, 1999). In NSCLC, gemcitabine monotherapy has shown a response rate greater than 20%, a median survival of 7–9 months, and favorable side effects in more than 500 patients in six phase II studies (Sederholm et al., 2005). Although gemcitabine, which has been extensively studied, is effective for most lung cancers, some patients cannot get effective drug responses due to the heterogeneity of lung cancers (Zito Marino et al., 2019). The CRLncSig score of our research can potentially be an excellent indicator to guide clinical gemcitabine medication. Known as daunorubicin, daunomycin is an anthracycline antibiotic that binds to DNA and causes helical unwinding, ultimately inhibiting DNA synthesis and DNA-dependent RNA synthesis (Murphy and Yee, 2017). This drug slows or stops the growth of cancer cells and can be used to treat acute myeloid leukemia, acute lymphoblastic leukemia, chronic myelogenous leukemia, and Kaposi’s sarcoma (Murphy and Yee, 2017). Potapov et al. demonstrated that compared to doxorubicin, daunomycin lowered the proportion of the DNA-synthesizing cells in the drug-sensitive xenografts of lung cancer more significantly (Potapov Iu et al., 1986). The experimental data suggested that daunomycin would be highly efficient in the chemotherapy of patients with lung cancer (Potapov Iu et al., 1986). Using co-delivery liposomes targeting daunorubicin and dioscin, Wang and colleagues demonstrated significant antitumor effects in tumor-bearing mice, which may provide an effective strategy for the treatment of NSCLC (Wang et al., 2019). Nobiletin is a naturally occurring compound with potential therapeutic effects against a variety of cancer types (Ashrafizadeh et al., 2020). Compound 29d, a derivative of nobiletin, has been shown to increase paclitaxel accumulation in lung cancer cells by reducing P-gp activity, thereby enhancing its antitumor activity (Feng et al., 2020). In terms of enhancing the antitumor activity of adriamycin, nobiletin inhibits Akt and Wnt/β-catenin signaling pathway by increasing GSK-3β activity, and leads to decreased lung cancer cells viability (Moon et al., 2018).

There are some limitations to this study. We generated this CRLncSig from publicly accessible data. Although it has been confirmed to have stable prognosis ability through applied to another large independent cohort and have differential expression patterns in tumor and normal tissues *via* silico and real-time PCR approaches, its clinical applicability needs further confirmation with more parameters. Furthermore, there are still no wet laboratory facts to hold up the nine lncRNAs’ parts in cuproptosis-related mechanisms. Therefore, more research, which focuses *in vivo* and *in vitro*, is urgently needed to reveal more clues that support the signature’s potential future.

## Conclusion

The present research constructed a novel and capable cuproptosis-related lncRNA signature, CRLncSig, for LUAD. Applying the signature to a large independent cohort and assessing its human tissue expression pattern using real-time PCR validated its stability and broad applicability. The signature owns the potential ability to undertake the role of precise immunotherapy. Our study identified potential immunotherapy targets and agents which might improve patients’ prognoses most effectively for those with high CRLncSig scores. In summary, this study has introduced new insights into personalized prognostication approaches and shed light on the integration of tailored prognosis prediction and precision therapy.

## Data Availability

Publicly available datasets were analyzed in this study. This data can be found here: This study used publicly available datasets. Their names and where to get them are as follows: TCGA and pan-cancer TCGA TARGET GTEx, https://xenabrowser.net; GSE29013, GSE30219, GSE31210, GSE37745, and GSE50081, https://www.ncbi.nlm.nih.gov/geo; CCLs database, https://depmap.org/portal; CTRP database, https://portals.broadinstitute.org/ctrp; PRISM database, https://depmap.org/portal/prism.
