# Supplementary material for: Prognosis and personalized treatment prediction in lung adenocarcinoma: An in silico and in vitro strategy adopting cuproptosis related lncRNA towards precision oncology
Source: Front Pharmacol. 2023 Feb 15;14:1113808. doi: 10.3389/fphar.2023.1113808 (PMC9975170; doi:10.3389/fphar.2023.1113808)
Supplement: Supplementary file 1 [file DataSheet1.PDF]

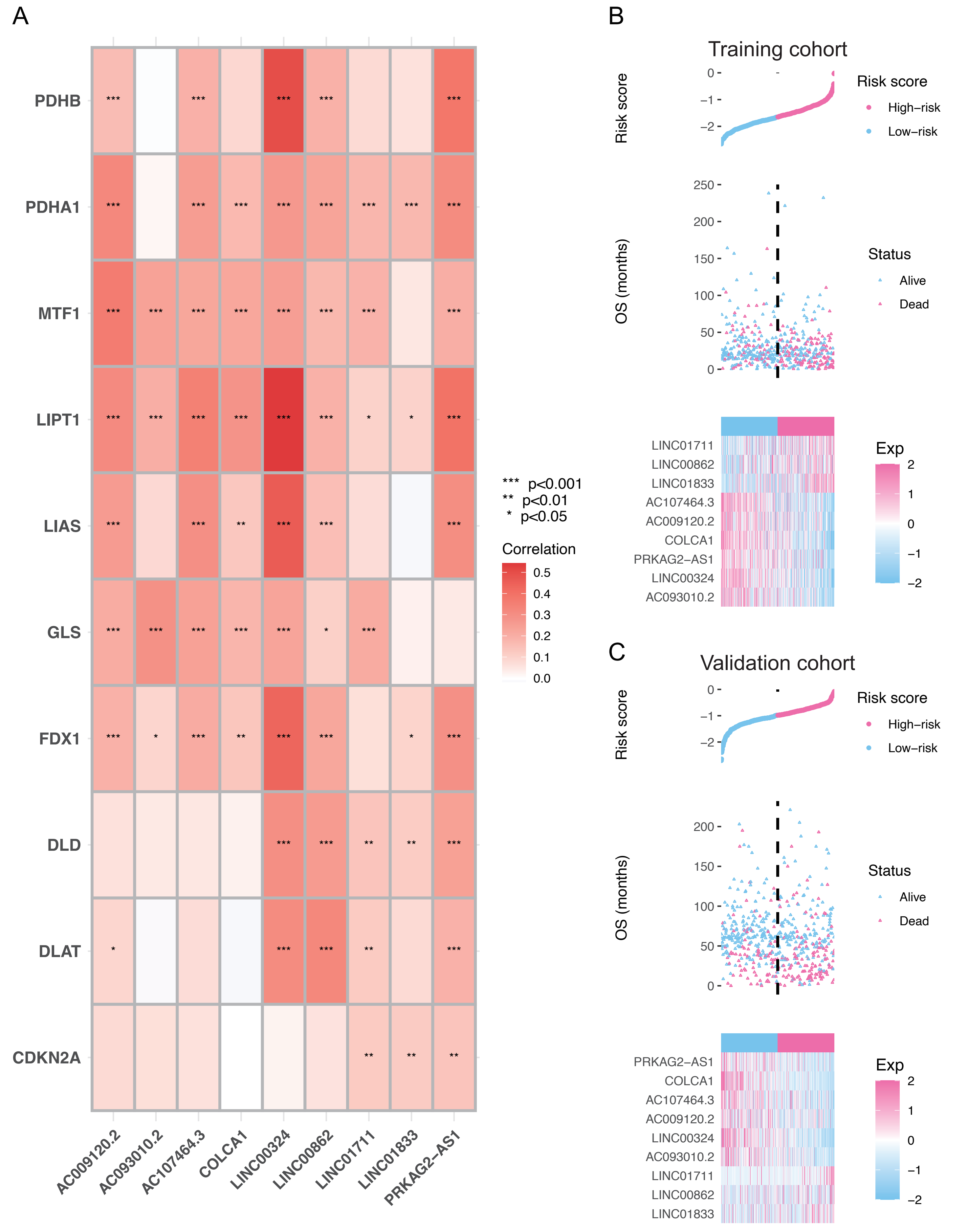

Figure S1. The profile of the signature lncRNAs. (A) The heatmap shows the signature's nine lncRNAs' correlations with the cuproptosis-related genes. **(B and C)** The distributions of the risk score, survival status, survival time, and nine lncRNAs' levels for LUAD cases in the training cohort and validation cohort.

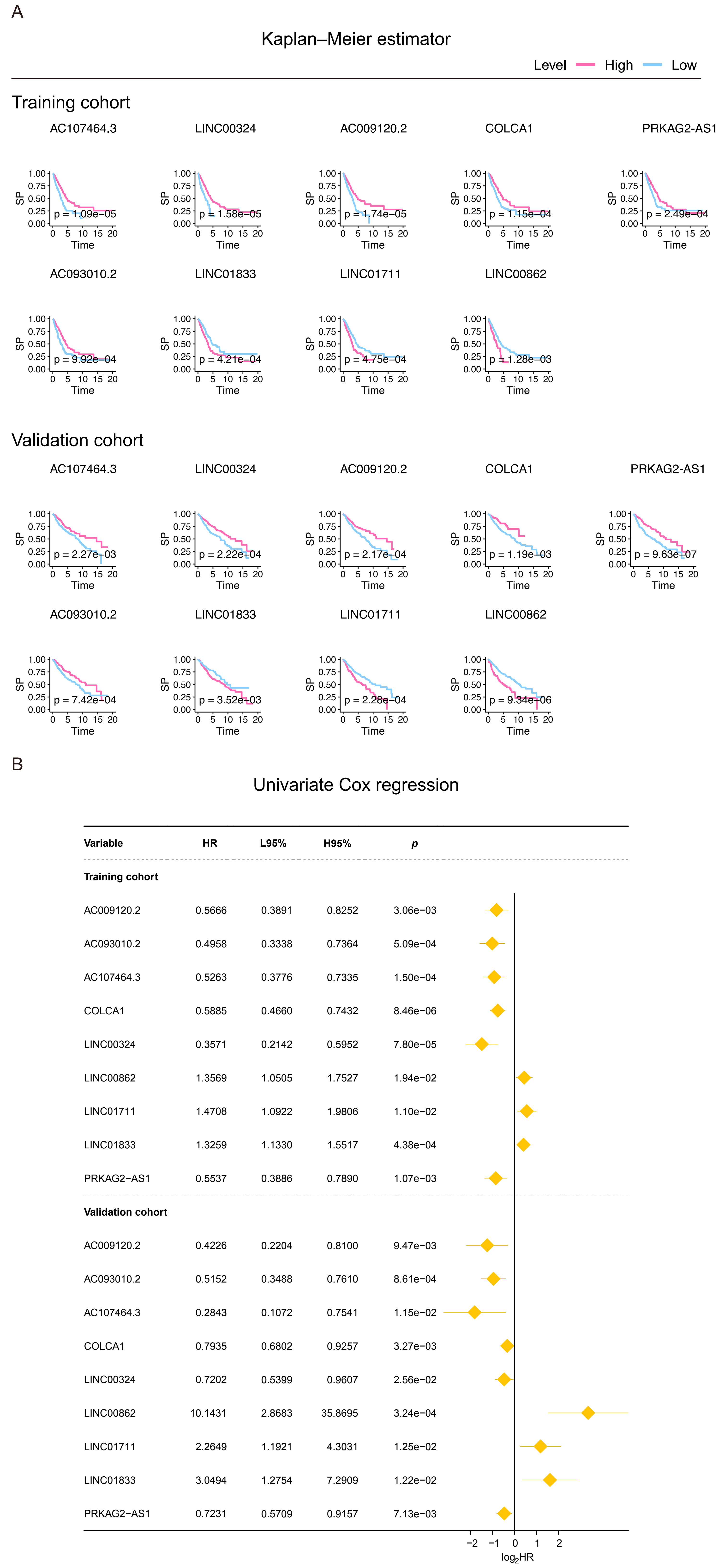

Figure S2. The Kaplan-Meier analysis (A) and univariate Cox models (B) established in the studied cohorts testing the predictive ability of each of the nine lncRNAs. Patients were grouped based on their median risk score. The Kaplan-Meier method compared the survival difference between high and low-risk patients, and the log-rank test examined the significance.

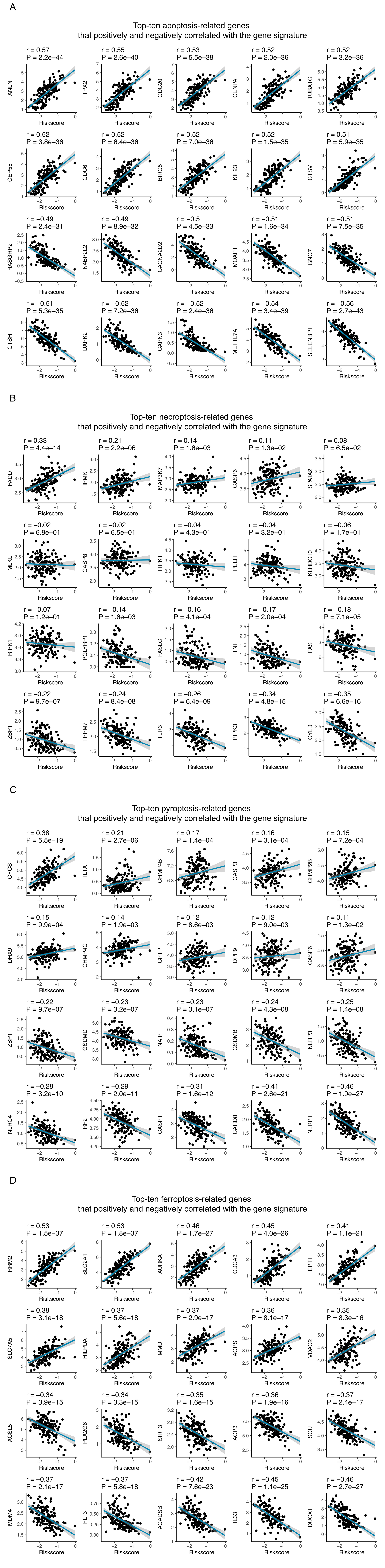

Figure S3. The correlations between the CRLncSig and apoptosis (A), necroptosis (B), pyroptosis (C), and ferroptosis (D) related genes in the training cohort. Only top correlations were plotted. The Pearson examination was carried out for correlation test.

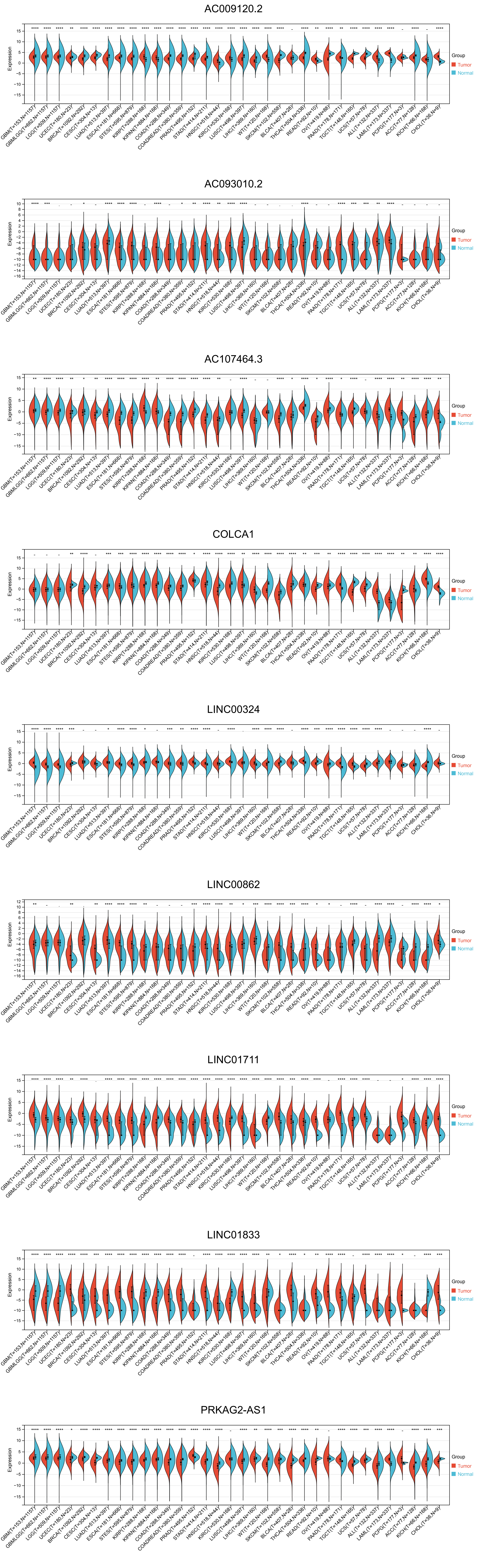

Figure S4. The CRLncSig's lncRNAs' expression pattern in pan-cancer.

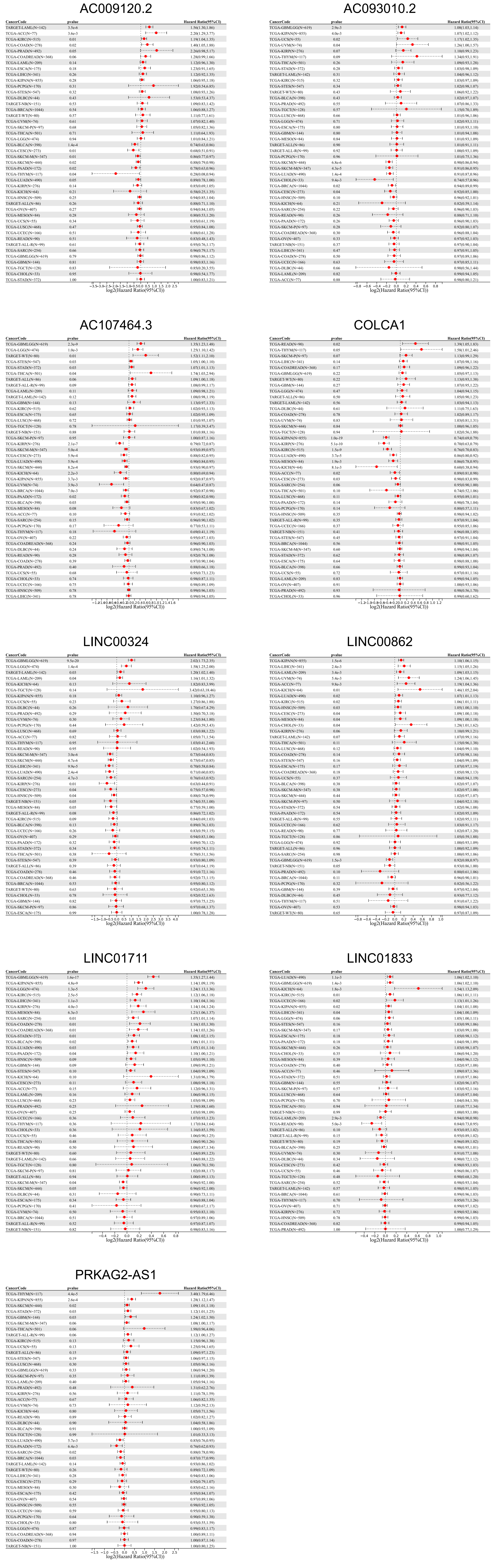

Figure S5. The CRLncSig's lncRNAs' prognostic ability assessment in pan-cancer.

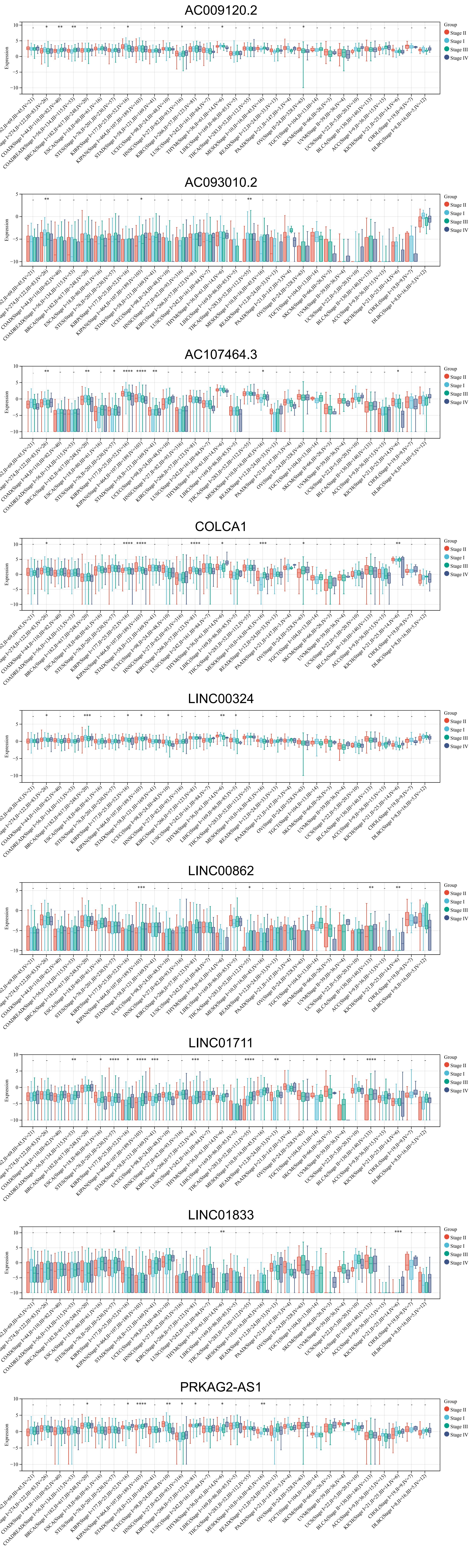

Figure S6. The CRLncSig's lncRNAs' staging power measurement in pan-cancer.

Table S1. 60 immune checkpoints obtained from previous studies.

ID	Type
VSIR	Inhibitory
VTCN1	Inhibitory
VEGFB	Inhibitory
VEGFA	Inhibitory
TNFSF9	Stimulaotry
TNFSF4	Stimulaotry
TNFRSF9	Stimulaotry
TNFRSF4	Stimulaotry
TNFRSF18	Stimulaotry
TNFRSF14	Stimulaotry
TNF	Stimulaotry
TLR4	Stimulaotry
TIGIT	Inhibitory
TGFB1	Inhibitory
SLAMF7	Inhibitory
SELP	Stimulaotry
PRF1	Stimulaotry
PDCD1	Inhibitory
LAG3	Inhibitory
KIR2DL3	Inhibitory
KIR2DL1	Inhibitory
ITGB2	Stimulaotry
IL4	Inhibitory
IL2RA	Stimulaotry
IL2	Stimulaotry
IL1B	Stimulaotry
IL1A	Stimulaotry
IL13	Inhibitory
IL12A	Inhibitory
IL10	Inhibitory
IFNG	Stimulaotry
IFNA2	Stimulaotry
IFNA1	Stimulaotry
IDO1	Inhibitory
ICOSLG	Stimulaotry
ICOS	Stimulaotry
ICAM1	Stimulaotry
HMGB1	Stimulaotry
HAVCR2	Inhibitory

GZMA	Stimulaotry
ENTPD1	Stimulaotry
EDNRB	Inhibitory
CXCL9	Stimulaotry
CXCL10	Stimulaotry
CX3CL1	Stimulaotry
CTLA4	Inhibitory
CD80	Stimulaotry
CD70	Stimulaotry
CD40LG	Stimulaotry
CD40	Stimulaotry
CD28	Stimulaotry
CD276	Inhibitory
CD274	Inhibitory
CD27	Stimulaotry
CCL5	Stimulaotry
BTN3A2	Stimulaotry
BTN3A1	Stimulaotry
BTLA	Inhibitory
ARG1	Inhibitory
ADORA2A	Inhibitory

Table S2. Co-expression genes of the cuproptosis-regulated genes.

Cuproptosis-regulated genes	Co-expression genes	Correlation coefficient	P-value	Regulation
MTF1	A2M	0.494077462	3.91E-32	postive
MTF1	AASS	0.50763251	4.18E-34	postive
MTF1	ABAT	0.412674669	5.55E-22	postive
MTF1	ABCA6	0.406591652	2.50E-21	postive
FDX1	ABHD14A	0.434839308	1.76E-24	postive
LIAS	ABHD14A	0.483314975	1.25E-30	postive
LIPT1	ABHD14A	0.45859148	2.27E-27	postive
PDHA1	ABHD14A	0.493115271	5.36E-32	postive
PDHB	ABHD14A	0.677836652	1.57E-68	postive
LIAS	AC004771.2	0.405541661	3.24E-21	postive
LIPT1	AC004771.2	0.49364862	4.50E-32	postive
PDHA1	AC004771.2	0.445411411	9.72E-26	postive
MTF1	AC004771.2	0.527341114	3.90E-37	postive
PDHA1	AC004825.2	0.40114699	9.39E-21	postive
LIPT1	AC004865.2	0.415669329	2.62E-22	postive
FDX1	AC005076.2	0.42132842	6.18E-23	postive
LIAS	AC005076.2	0.480561807	2.97E-30	postive
LIPT1	AC005076.2	0.490827376	1.13E-31	postive
PDHA1	AC005076.2	0.424569145	2.67E-23	postive
MTF1	AC005154.2	0.448455704	4.14E-26	postive
LIPT1	AC005332.4	0.433398953	2.59E-24	postive
MTF1	AC005332.4	0.464426684	4.08E-28	postive
GLS	AC005332.4	0.438758142	6.09E-25	postive
LIAS	AC006449.2	0.503891837	1.49E-33	postive
LIPT1	AC006449.2	0.524864149	9.62E-37	postive
DLAT	AC006449.2	0.485439491	6.36E-31	postive
PDHA1	AC006449.2	0.507723345	4.05E-34	postive
PDHB	AC006449.2	0.575248746	2.17E-45	postive
MTF1	AC006449.2	0.445059276	1.07E-25	postive
FDX1	AC006449.7	0.434745223	1.81E-24	postive
LIAS	AC006449.7	0.524823552	9.76E-37	postive
LIPT1	AC006449.7	0.592177879	1.21E-48	postive
PDHA1	AC006449.7	0.532692382	5.42E-38	postive
PDHB	AC006449.7	0.569356361	2.67E-44	postive
LIPT1	AC006547.1	0.424973725	2.40E-23	postive
PDHA1	AC006547.1	0.495283284	2.63E-32	postive
MTF1	AC006547.1	0.604921968	3.18E-51	postive
LIPT1	AC007066.2	0.505865243	7.64E-34	postive
PDHA1	AC007066.2	0.448583361	3.99E-26	postive
MTF1	AC007066.2	0.462580531	7.04E-28	postive
FDX1	AC007114.1	0.40457616	4.10E-21	postive
LIAS	AC007114.1	0.484096021	9.75E-31	postive

LIPT1	AC007114.1	0.495480595	2.47E-32	postive
PDHA1	AC007114.1	0.463402899	5.52E-28	postive
PDHB	AC007114.1	0.414263785	3.73E-22	postive
MTF1	AC007114.2	0.456986652	3.62E-27	postive
DLD	AC007318.1	0.507598439	4.22E-34	postive
DLAT	AC007318.1	0.514806965	3.47E-35	postive
PDHA1	AC007318.1	0.458887406	2.08E-27	postive
PDHB	AC007318.1	0.450815183	2.12E-26	postive
MTF1	AC008267.3	0.464320647	4.21E-28	postive
LIPT1	AC008669.1	0.416266157	2.25E-22	postive
LIPT1	AC008763.1	0.439253754	5.32E-25	postive
MTF1	AC008764.2	0.634931351	8.74E-58	postive
MTF1	AC008770.3	0.486164568	5.05E-31	postive
LIPT1	AC008937.3	0.40859718	1.53E-21	postive
LIPT1	AC009053.2	0.400685168	1.05E-20	postive
MTF1	AC009053.2	0.577405713	8.57E-46	postive
LIAS	AC009118.3	0.413675921	4.32E-22	postive
PDHA1	AC009118.3	0.465066821	3.37E-28	postive
MTF1	AC009118.3	0.406451887	2.59E-21	postive
LIPT1	AC009283.1	0.401002298	9.72E-21	postive
PDHA1	AC009283.1	0.475757171	1.32E-29	postive
MTF1	AC009283.1	0.417643227	1.59E-22	postive
FDX1	AC009506.2	0.448783798	3.77E-26	postive
LIAS	AC009506.2	0.458743691	2.17E-27	postive
LIPT1	AC009506.2	0.623189636	3.92E-55	postive
PDHA1	AC009506.2	0.425554884	2.07E-23	postive
PDHB	AC009506.2	0.480497647	3.03E-30	postive
PDHA1	AC009570.1	0.439080292	5.58E-25	postive
LIAS	AC009690.2	0.456448739	4.23E-27	postive
LIPT1	AC009690.2	0.456176348	4.57E-27	postive
PDHA1	AC009690.2	0.473779203	2.43E-29	postive
MTF1	AC009690.2	0.426551682	1.59E-23	postive
PDHA1	AC009812.1	0.403070474	5.90E-21	postive
PDHB	AC009812.1	0.423439235	3.58E-23	postive
LIPT1	AC009831.1	0.433938689	2.24E-24	postive
DLAT	AC009831.1	0.402247412	7.20E-21	postive
PDHA1	AC009831.1	0.4588877	2.08E-27	postive
PDHB	AC009831.1	0.443790632	1.53E-25	postive
MTF1	AC009831.1	0.583746204	5.33E-47	postive
MTF1	AC010260.1	0.449186482	3.37E-26	postive
MTF1	AC010319.1	0.526568502	5.17E-37	postive
LIPT1	AC010422.6	0.447057105	6.13E-26	postive
DLD	AC010422.6	0.446657228	6.86E-26	postive
DLAT	AC010422.6	0.504398551	1.26E-33	postive
PDHA1	AC010422.6	0.482808202	1.47E-30	postive
PDHB	AC010422.6	0.447938856	4.79E-26	postive
MTF1	AC010422.6	0.72414845	2.13E-82	postive
MTF1	AC010615.1	0.516737705	1.76E-35	postive

LIPT1	AC010618.3	0.447588706	5.28E-26	postive
MTF1	AC010618.3	0.419013734	1.12E-22	postive
PDHA1	AC010654.1	0.4358815	1.33E-24	postive
LIPT1	AC011477.1	0.400868864	1.00E-20	postive
MTF1	AC011477.1	0.401122244	9.44E-21	postive
LIPT1	AC011477.2	0.432209904	3.57E-24	postive
MTF1	AC011477.2	0.411550782	7.35E-22	postive
MTF1	AC011477.3	0.412563323	5.71E-22	postive
DLD	AC012085.1	0.499788344	5.92E-33	postive
DLAT	AC012085.1	0.471083893	5.52E-29	postive
PDHB	AC012085.1	0.431155311	4.72E-24	postive
GLS	AC012368.1	0.404946677	3.74E-21	postive
MTF1	AC012557.1	0.452418457	1.35E-26	postive
LIPT1	AC012615.1	0.495691639	2.30E-32	postive
PDHA1	AC012615.1	0.413226994	4.84E-22	postive
LIPT1	AC015802.5	0.423924228	3.16E-23	postive
LIPT1	AC015871.3	0.40810935	1.72E-21	postive
MTF1	AC015871.3	0.493093081	5.40E-32	postive
LIAS	AC015982.2	0.4261967	1.75E-23	postive
LIPT1	AC015982.2	0.585520096	2.43E-47	postive
PDHA1	AC015982.2	0.444675963	1.19E-25	postive
PDHB	AC015982.2	0.507036545	5.12E-34	postive
MTF1	AC016722.2	0.545050318	4.95E-40	postive
FDX1	AC016727.1	0.43743088	8.74E-25	postive
LIAS	AC016727.1	0.489020859	2.02E-31	postive
LIPT1	AC016727.1	0.595022682	3.28E-49	postive
DLAT	AC016727.1	0.413052196	5.05E-22	postive
PDHA1	AC016727.1	0.445530899	9.40E-26	postive
PDHB	AC016727.1	0.457893442	2.78E-27	postive
MTF1	AC016727.1	0.447703139	5.12E-26	postive
LIPT1	AC018845.3	0.416530511	2.10E-22	postive
DLAT	AC019069.1	0.400341327	1.14E-20	postive
LIPT1	AC019205.1	0.474351679	2.04E-29	postive
PDHA1	AC019205.1	0.42917618	7.98E-24	postive
MTF1	AC019205.1	0.551553202	3.86E-41	postive
LIPT1	AC020765.2	0.402678786	6.49E-21	postive
PDHA1	AC020765.2	0.40700262	2.26E-21	postive
FDX1	AC021016.2	0.474348669	2.04E-29	postive
LIPT1	AC021016.2	0.441772632	2.66E-25	postive
PDHB	AC021016.2	0.41964796	9.52E-23	postive
MTF1	AC021016.2	0.416129266	2.33E-22	postive
PDHA1	AC021242.3	0.410378391	9.84E-22	postive
MTF1	AC021739.2	0.460238683	1.40E-27	postive
DLD	AC022149.1	0.47088072	5.87E-29	postive
DLAT	AC022149.1	0.500477995	4.70E-33	postive
MTF1	AC022149.1	0.433257339	2.69E-24	postive
LIPT1	AC022916.2	0.501036535	3.90E-33	postive
PDHA1	AC022916.2	0.482292327	1.72E-30	postive

MTF1	AC022916.2	0.400834583	1.01E-20	postive
MTF1	AC024075.1	0.644637842	4.58E-60	postive
LIPT1	AC024075.2	0.402373601	6.99E-21	postive
MTF1	AC024075.2	0.47352608	2.62E-29	postive
MTF1	AC024075.3	0.642311132	1.64E-59	postive
PDHA1	AC024884.2	0.432237744	3.54E-24	postive
LIPT1	AC024941.2	0.404030542	4.68E-21	postive
LIAS	AC025181.2	0.402563561	6.67E-21	postive
LIPT1	AC025181.2	0.430771776	5.23E-24	postive
PDHA1	AC025181.2	0.470863489	5.90E-29	postive
PDHB	AC025181.2	0.417800721	1.52E-22	postive
MTF1	AC025287.3	0.479135319	4.64E-30	postive
MTF1	AC025917.1	0.488324648	2.53E-31	postive
LIPT1	AC026471.2	0.400083927	1.21E-20	postive
LIAS	AC026801.2	0.441815003	2.63E-25	postive
LIPT1	AC026801.2	0.523551385	1.55E-36	postive
PDHB	AC026801.2	0.412241445	6.19E-22	postive
PDHA1	AC026979.2	0.437139466	9.46E-25	postive
PDHB	AC026979.2	0.449488387	3.09E-26	postive
LIPT1	AC027097.1	0.456754719	3.87E-27	postive
DLAT	AC027097.1	0.408014331	1.77E-21	postive
PDHA1	AC027097.1	0.402328149	7.06E-21	postive
PDHB	AC027097.1	0.530405604	1.27E-37	postive
LIPT1	AC027097.2	0.428179839	1.04E-23	postive
MTF1	AC027097.2	0.420275952	8.10E-23	postive
LIPT1	AC027237.4	0.426093837	1.79E-23	postive
MTF1	AC027601.3	0.401371281	8.89E-21	postive
MTF1	AC060766.6	0.413843052	4.15E-22	postive
LIAS	AC060780.1	0.42033984	7.97E-23	postive
LIPT1	AC060780.1	0.538294983	6.60E-39	postive
PDHA1	AC060780.1	0.503609359	1.64E-33	postive
MTF1	AC060780.1	0.564478785	2.05E-43	postive
MTF1	AC063950.1	0.447989941	4.72E-26	postive
DLAT	AC068228.2	0.429344153	7.63E-24	postive
LIPT1	AC068338.3	0.409191394	1.32E-21	postive
PDHB	AC068338.3	0.420382984	7.88E-23	postive
LIAS	AC068473.5	0.411772957	6.96E-22	postive
LIPT1	AC068473.5	0.537355339	9.42E-39	postive
PDHA1	AC068473.5	0.496729836	1.64E-32	postive
PDHB	AC068473.5	0.506869633	5.42E-34	postive
LIPT1	AC068620.1	0.462714683	6.77E-28	postive
LIPT1	AC068888.1	0.412632305	5.61E-22	postive
PDHA1	AC068888.1	0.475206861	1.57E-29	postive
PDHB	AC068888.1	0.437018838	9.78E-25	postive
MTF1	AC068896.3	0.534074313	3.24E-38	postive
LIPT1	AC069224.2	0.455824323	5.06E-27	postive
LIPT1	AC074032.1	0.45688425	3.72E-27	postive
LIPT1	AC079848.1	0.437309059	9.03E-25	postive

LIAS	AC083798.2	0.450844008	2.11E-26	postive
LIPT1	AC083798.2	0.532934885	4.95E-38	postive
PDHA1	AC083798.2	0.402172298	7.33E-21	postive
PDHB	AC083798.2	0.407160487	2.18E-21	postive
MTF1	AC087163.2	0.564875119	1.74E-43	postive
MTF1	AC087521.2	0.439402275	5.11E-25	postive
MTF1	AC087854.1	0.506157326	6.91E-34	postive
FDX1	AC090617.5	0.412251453	6.17E-22	postive
LIAS	AC090617.5	0.408829551	1.44E-21	postive
LIPT1	AC090617.5	0.522118125	2.59E-36	postive
DLD	AC090617.5	0.406511962	2.55E-21	postive
PDHB	AC090617.5	0.466630364	2.11E-28	postive
MTF1	AC090617.5	0.443360724	1.72E-25	postive
MTF1	AC090948.2	0.485870719	5.55E-31	postive
LIPT1	AC092171.5	0.41118232	8.06E-22	postive
LIPT1	AC092279.1	0.44713696	6.00E-26	postive
PDHB	AC092279.1	0.425619869	2.03E-23	postive
MTF1	AC092279.1	0.436044806	1.27E-24	postive
FDX1	AC092718.4	0.41481649	3.25E-22	postive
DLD	AC092718.4	0.477574116	7.54E-30	postive
DLAT	AC092718.4	0.425509656	2.09E-23	postive
PDHA1	AC092718.4	0.595914156	2.18E-49	postive
PDHA1	AC092803.1	0.549359801	9.18E-41	postive
MTF1	AC092803.1	0.406948976	2.29E-21	postive
MTF1	AC093525.7	0.404778225	3.90E-21	postive
PDHA1	AC097639.1	0.400249499	1.16E-20	postive
MTF1	AC097639.1	0.425114138	2.32E-23	postive
FDX1	AC098484.1	0.401508517	8.61E-21	postive
LIAS	AC098484.1	0.482051426	1.86E-30	postive
LIPT1	AC098484.1	0.560884443	8.99E-43	postive
DLD	AC098484.1	0.487944576	2.86E-31	postive
DLAT	AC098484.1	0.530338992	1.30E-37	postive
PDHB	AC098484.1	0.498937732	7.86E-33	postive
MTF1	AC098484.1	0.624155216	2.40E-55	postive
DLD	AC099850.4	0.514772648	3.52E-35	postive
DLAT	AC099850.4	0.491329272	9.59E-32	postive
MTF1	AC102945.2	0.424847638	2.48E-23	postive
PDHA1	AC103974.1	0.410523129	9.49E-22	postive
MTF1	AC103974.1	0.450210602	2.52E-26	postive
PDHA1	AC104564.3	0.401806767	8.01E-21	postive
MTF1	AC104564.3	0.445369617	9.83E-26	postive
MTF1	AC105020.6	0.475341605	1.50E-29	postive
GLS	AC105942.1	0.421505527	5.91E-23	postive
MTF1	AC107959.1	0.459567011	1.71E-27	postive
MTF1	AC111170.4	0.442040087	2.48E-25	postive
DLD	AC112777.1	0.498077499	1.05E-32	postive
DLAT	AC112777.1	0.539636517	3.96E-39	postive
PDHA1	AC112777.1	0.44338081	1.71E-25	postive

MTF1	AC112777.1	0.495237543	2.67E-32	postive
MTF1	AC114760.2	0.424564477	2.67E-23	postive
PDHA1	AC114810.1	0.401252411	9.15E-21	postive
PDHB	AC114810.1	0.466862363	1.97E-28	postive
GLS	AC119044.1	0.413940957	4.04E-22	postive
MTF1	AC119396.1	0.455479242	5.59E-27	postive
MTF1	AC122129.1	0.596479547	1.68E-49	postive
LIPT1	AC124248.1	0.418437735	1.30E-22	postive
DLAT	AC131009.1	0.424888184	2.46E-23	postive
PDHA1	AC131009.1	0.432832753	3.02E-24	postive
MTF1	AC133552.4	0.437541122	8.48E-25	postive
LIAS	AC135050.6	0.421941088	5.28E-23	postive
LIPT1	AC135050.6	0.493822806	4.25E-32	postive
PDHA1	AC135050.6	0.488449882	2.43E-31	postive
PDHB	AC135050.6	0.417192394	1.78E-22	postive
GLS	AC138207.1	0.417642582	1.59E-22	postive
LIPT1	AC138393.1	0.431454077	4.36E-24	postive
MTF1	AC138393.1	0.462864351	6.48E-28	postive
PDHA1	AC138969.1	0.416194046	2.29E-22	postive
MTF1	AC138969.1	0.464601426	3.87E-28	postive
PDHA1	AC139256.3	0.499546327	6.42E-33	postive
MTF1	AC139256.3	0.48297977	1.39E-30	postive
LIPT1	AC139768.1	0.461456272	9.81E-28	postive
PDHA1	AC139768.1	0.509973719	1.87E-34	postive
PDHB	AC139768.1	0.478227486	6.15E-30	postive
LIPT1	AC139769.1	0.434153853	2.12E-24	postive
MTF1	AC139769.1	0.403914939	4.81E-21	postive
LIPT1	AC145285.2	0.423588673	3.45E-23	postive
PDHA1	AC231657.1	0.441017712	3.28E-25	postive
LIAS	AC233723.2	0.402200041	7.28E-21	postive
LIPT1	AC233723.2	0.434314538	2.03E-24	postive
PDHA1	AC233723.2	0.43039082	5.79E-24	postive
PDHB	AC233723.2	0.429212237	7.91E-24	postive
PDHA1	AC233728.1	0.459093383	1.96E-27	postive
FDX1	AC244090.1	0.400729173	1.04E-20	postive
LIAS	AC244090.1	0.409569458	1.20E-21	postive
LIPT1	AC244090.1	0.451834964	1.59E-26	postive
PDHA1	AC244090.1	0.441399389	2.95E-25	postive
PDHB	AC244090.1	0.455527712	5.51E-27	postive
FDX1	ACAD8	0.412788908	5.40E-22	postive
LIPT1	ACAD8	0.477180048	8.51E-30	postive
MTF1	ACAD8	0.439823857	4.55E-25	postive
LIAS	ACADSB	0.449270224	3.29E-26	postive
LIPT1	ACADSB	0.435401926	1.51E-24	postive
PDHB	ACADSB	0.455071029	6.29E-27	postive
LIAS	ACCS	0.43007921	6.28E-24	postive
LIPT1	ACCS	0.421985249	5.22E-23	postive
PDHA1	ACCS	0.445603606	9.21E-26	postive

LIPT1	ACSS1	0.433526385	2.51E-24	postive
LIPT1	ACVR2B-AS1	0.404575993	4.10E-21	postive
PDHA1	ACVR2B-AS1	0.512824682	6.95E-35	postive
PDHB	ACVR2B-AS1	0.525304921	8.19E-37	postive
MTF1	ACVR2B-AS1	0.42646601	1.63E-23	postive
DLD	ADA	0.447834018	4.93E-26	postive
MTF1	ADA2	0.462275803	7.70E-28	postive
GLS	ADGRE4P	0.41655163	2.09E-22	postive
DLD	ADM	0.408433603	1.59E-21	postive
MTF1	ADPGK-AS1	0.463553417	5.28E-28	postive
MTF1	AF131215.5	0.404625036	4.05E-21	postive
LIPT1	AF274858.1	0.400149787	1.19E-20	postive
PDHA1	AF274858.1	0.419502304	9.88E-23	postive
DLD	AGMAT	0.502772533	2.18E-33	postive
DLAT	AGMAT	0.406983836	2.27E-21	postive
PDHA1	AGMAT	0.41533448	2.85E-22	postive
FDX1	AHSA1	0.50572471	8.01E-34	postive
LIAS	AHSA1	0.549120324	1.01E-40	postive
LIPT1	AHSA1	0.524008744	1.31E-36	postive
DLD	AHSA1	0.644795214	4.20E-60	postive
DLAT	AHSA1	0.590410586	2.70E-48	postive
PDHA1	AHSA1	0.672513702	4.23E-67	postive
PDHB	AHSA1	0.691840562	1.90E-72	postive
MTF1	AHSA1	0.441736665	2.69E-25	postive
FDX1	AK2	0.521046495	3.81E-36	postive
LIAS	AK2	0.580348307	2.38E-46	postive
LIPT1	AK2	0.619670477	2.33E-54	postive
DLD	AK2	0.471242226	5.26E-29	postive
DLAT	AK2	0.522752708	2.06E-36	postive
PDHA1	AK2	0.60420338	4.47E-51	postive
PDHB	AK2	0.730110118	2.16E-84	postive
MTF1	AK2	0.426899296	1.45E-23	postive
DLD	AK4P1	0.423302555	3.71E-23	postive
FDX1	AK9	0.419912286	8.89E-23	postive
LIPT1	AK9	0.476532682	1.04E-29	postive
DLAT	AK9	0.411685544	7.11E-22	postive
PDHA1	AK9	0.417617529	1.60E-22	postive
PDHB	AK9	0.407572259	1.97E-21	postive
MTF1	AK9	0.55146478	4.00E-41	postive
MTF1	AKNA	0.625949672	9.56E-56	postive
FDX1	AKR1A1	0.594302631	4.57E-49	postive
LIAS	AKR1A1	0.548747349	1.17E-40	postive
LIPT1	AKR1A1	0.621849138	7.75E-55	postive
DLD	AKR1A1	0.498392107	9.43E-33	postive
DLAT	AKR1A1	0.495575399	2.39E-32	postive
PDHA1	AKR1A1	0.608671006	5.25E-52	postive
PDHB	AKR1A1	0.63608072	4.74E-58	postive
MTF1	AKR1A1	0.44669616	6.79E-26	postive

FDX1	AKT1S1	0.466358425	2.29E-28	postive
LIAS	AKT1S1	0.41729215	1.73E-22	postive
LIPT1	AKT1S1	0.423990149	3.11E-23	postive
DLD	AKT1S1	0.535851864	1.66E-38	postive
DLAT	AKT1S1	0.510897912	1.36E-34	postive
PDHA1	AKT1S1	0.667373224	9.58E-66	postive
PDHB	AKT1S1	0.573385911	4.83E-45	postive
MTF1	AKT1S1	0.552509448	2.64E-41	postive
LIPT1	AL021368.2	0.442926385	1.94E-25	postive
MTF1	AL021707.4	0.401384751	8.87E-21	postive
LIAS	AL022157.1	0.444732919	1.17E-25	postive
LIPT1	AL022157.1	0.453126066	1.10E-26	postive
PDHA1	AL022157.1	0.435544796	1.46E-24	postive
MTF1	AL031118.1	0.420451601	7.74E-23	postive
MTF1	AL031719.1	0.501506327	3.33E-33	postive
LIPT1	AL035587.1	0.487320818	3.49E-31	postive
DLAT	AL035587.1	0.418592897	1.25E-22	postive
PDHA1	AL035587.1	0.520817794	4.13E-36	postive
PDHB	AL035587.1	0.40872395	1.48E-21	postive
MTF1	AL035587.1	0.669186628	3.21E-66	postive
FDX1	AL049834.1	0.428834502	8.74E-24	postive
LIAS	AL049834.1	0.478726907	5.27E-30	postive
LIPT1	AL049834.1	0.574857924	2.57E-45	postive
DLD	AL049834.1	0.422441321	4.64E-23	postive
DLAT	AL049834.1	0.449206602	3.35E-26	postive
PDHA1	AL049834.1	0.417838962	1.51E-22	postive
PDHB	AL049834.1	0.508032503	3.64E-34	postive
MTF1	AL049834.1	0.441062366	3.24E-25	postive
GLS	AL049834.1	0.411466394	7.51E-22	postive
LIAS	AL050341.2	0.480766395	2.78E-30	postive
LIPT1	AL050341.2	0.634280968	1.23E-57	postive
PDHA1	AL050341.2	0.467803203	1.49E-28	postive
PDHB	AL050341.2	0.460906988	1.15E-27	postive
MTF1	AL096840.1	0.446817489	6.56E-26	postive
LIPT1	AL109955.1	0.422029621	5.16E-23	postive
MTF1	AL109955.1	0.424429182	2.77E-23	postive
PDHA1	AL121832.2	0.54190944	1.66E-39	postive
PDHA1	AL121906.2	0.485579613	6.09E-31	postive
MTF1	AL121906.2	0.406489487	2.57E-21	postive
LIPT1	AL122010.1	0.438974396	5.74E-25	postive
PDHA1	AL122010.1	0.547391315	1.99E-40	postive
PDHB	AL122010.1	0.432599244	3.21E-24	postive
MTF1	AL122010.1	0.641112082	3.16E-59	postive
LIPT1	AL132988.1	0.427432212	1.26E-23	postive
PDHB	AL132988.1	0.413972072	4.01E-22	postive
MTF1	AL133227.1	0.48436017	8.97E-31	postive
MTF1	AL133371.2	0.429206054	7.92E-24	postive
PDHA1	AL133520.1	0.472916271	3.16E-29	postive

MTF1	AL133520.1	0.502146398	2.69E-33	postive
LIPT1	AL135925.1	0.425154406	2.29E-23	postive
PDHA1	AL135925.1	0.483106666	1.33E-30	postive
PDHB	AL135925.1	0.476328988	1.11E-29	postive
LIAS	AL136304.1	0.4012144	9.24E-21	postive
PDHA1	AL136304.1	0.441074073	3.23E-25	postive
LIPT1	AL137003.1	0.457537759	3.08E-27	postive
MTF1	AL137003.1	0.431241218	4.62E-24	postive
GLS	AL137003.1	0.474919842	1.71E-29	postive
MTF1	AL137013.1	0.443279345	1.76E-25	postive
LIPT1	AL139353.1	0.443910453	1.48E-25	postive
PDHB	AL139353.1	0.42098881	6.75E-23	postive
MTF1	AL139811.1	0.410629013	9.25E-22	postive
MTF1	AL158163.1	0.441992346	2.51E-25	postive
MTF1	AL161457.2	0.418619512	1.24E-22	postive
LIAS	AL162377.1	0.406131558	2.80E-21	postive
LIPT1	AL162377.1	0.518047786	1.11E-35	postive
PDHA1	AL162377.1	0.47442485	1.99E-29	postive
PDHB	AL162377.1	0.510070569	1.81E-34	postive
MTF1	AL162377.1	0.448667917	3.90E-26	postive
PDHA1	AL163051.1	0.437388169	8.84E-25	postive
PDHB	AL163051.1	0.4099091	1.11E-21	postive
MTF1	AL353588.1	0.42159532	5.77E-23	postive
LIPT1	AL353743.1	0.448584169	3.99E-26	postive
PDHA1	AL353743.1	0.457944629	2.74E-27	postive
PDHB	AL353743.1	0.456903737	3.70E-27	postive
MTF1	AL353743.1	0.441141837	3.17E-25	postive
MTF1	AL353763.1	0.499477283	6.57E-33	postive
MTF1	AL353804.1	0.43859015	6.38E-25	postive
FDX1	AL354714.2	0.442325544	2.29E-25	postive
DLD	AL354714.2	0.614904927	2.50E-53	postive
DLAT	AL354714.2	0.611900371	1.09E-52	postive
PDHA1	AL354714.2	0.589966184	3.30E-48	postive
PDHB	AL354714.2	0.467810932	1.48E-28	postive
MTF1	AL354714.2	0.497383833	1.32E-32	postive
LIPT1	AL357079.1	0.507562344	4.28E-34	postive
PDHA1	AL357079.1	0.433023296	2.87E-24	postive
MTF1	AL357079.1	0.402352985	7.02E-21	postive
LIAS	AL358472.2	0.461232714	1.05E-27	postive
LIPT1	AL358472.2	0.595913692	2.18E-49	postive
PDHA1	AL358472.2	0.444360285	1.30E-25	postive
PDHB	AL358472.2	0.448242529	4.40E-26	postive
FDX1	AL359183.1	0.409120147	1.34E-21	postive
PDHA1	AL359183.1	0.450208247	2.52E-26	postive
MTF1	AL359183.1	0.531271827	9.18E-38	postive
MTF1	AL359220.1	0.451306844	1.85E-26	postive
DLAT	AL360270.1	0.41531947	2.86E-22	postive
MTF1	AL360270.1	0.478722539	5.27E-30	postive

MTF1	AL365203.2	0.421924201	5.30E-23	postive
MTF1	AL390195.2	0.443867866	1.49E-25	postive
LIPT1	AL390728.4	0.505038014	1.01E-33	postive
MTF1	AL390728.4	0.436291187	1.19E-24	postive
LIPT1	AL391069.2	0.45402142	8.50E-27	postive
PDHA1	AL391069.2	0.482183412	1.78E-30	postive
PDHB	AL391069.2	0.458915102	2.06E-27	postive
MTF1	AL445309.1	0.445400291	9.75E-26	postive
MTF1	AL445991.1	0.415367024	2.82E-22	postive
FDX1	AL451165.2	0.479811279	3.76E-30	postive
LIAS	AL451165.2	0.497326765	1.34E-32	postive
LIPT1	AL451165.2	0.545282326	4.52E-40	postive
PDHA1	AL451165.2	0.507923556	3.78E-34	postive
PDHB	AL451165.2	0.518420105	9.71E-36	postive
DLD	AL662899.4	0.452063875	1.49E-26	postive
DLAT	AL662899.4	0.474161942	2.16E-29	postive
PDHA1	AL662899.4	0.418541278	1.26E-22	postive
PDHB	AL662899.4	0.433000823	2.89E-24	postive
MTF1	AL662907.1	0.586738175	1.41E-47	postive
MTF1	AL662907.2	0.516639694	1.82E-35	postive
MTF1	AL683813.2	0.413647399	4.35E-22	postive
LIPT1	AL691432.2	0.459114497	1.95E-27	postive
PDHA1	AL691432.2	0.459937253	1.53E-27	postive
MTF1	AL691432.2	0.490972518	1.08E-31	postive
PDHA1	AL731533.2	0.417168645	1.79E-22	postive
LIPT1	AL731563.4	0.413923644	4.06E-22	postive
MTF1	AL731563.4	0.484880537	7.60E-31	postive
FDX1	ALDH2	0.424917221	2.44E-23	postive
LIAS	ALDH2	0.411389019	7.66E-22	postive
PDHA1	ALDH2	0.444349945	1.31E-25	postive
PDHB	ALDH2	0.480095753	3.44E-30	postive
LIAS	ALDH6A1	0.406238159	2.73E-21	postive
LIPT1	ALDH6A1	0.424241396	2.91E-23	postive
DLD	ALDH6A1	0.463140136	5.97E-28	postive
DLAT	ALDH6A1	0.478448226	5.74E-30	postive
PDHA1	ALDH6A1	0.484263489	9.25E-31	postive
PDHB	ALDH6A1	0.498441159	9.28E-33	postive
MTF1	ALDH6A1	0.480472642	3.05E-30	postive
LIPT1	ALKBH3-AS1	0.40598855	2.90E-21	postive
MTF1	ALKBH3-AS1	0.468222726	1.31E-28	postive
MTF1	AMD1P3	0.409521501	1.22E-21	postive
LIPT1	AMIGO1	0.407131381	2.19E-21	postive
PDHA1	AMIGO1	0.427388447	1.28E-23	postive
PDHB	AMIGO1	0.403544919	5.26E-21	postive
MTF1	AMIGO1	0.503021661	2.00E-33	postive
FDX1	ANGEL1	0.409277911	1.29E-21	postive
LIAS	ANGEL1	0.466076243	2.49E-28	postive
LIPT1	ANGEL1	0.550024575	7.06E-41	postive

DLD	ANGEL1	0.517492041	1.35E-35	postive
DLAT	ANGEL1	0.50379566	1.54E-33	postive
PDHA1	ANGEL1	0.570375076	1.73E-44	postive
PDHB	ANGEL1	0.526397422	5.51E-37	postive
MTF1	ANGEL1	0.628654707	2.36E-56	postive
PDHA1	ANKRD53	0.40036983	1.13E-20	postive
MTF1	ANKRD53	0.410579497	9.36E-22	postive
DLD	ANLN	0.491978177	7.76E-32	postive
DLAT	ANLN	0.463549048	5.29E-28	postive
MTF1	ANOS1	0.400242632	1.17E-20	postive
MTF1	AOC4P	0.406121432	2.81E-21	postive
MTF1	AP000763.3	0.400173283	1.19E-20	postive
LIPT1	AP000787.1	0.451446107	1.78E-26	postive
LIPT1	AP000894.4	0.452204447	1.43E-26	postive
PDHA1	AP000894.4	0.468402427	1.24E-28	postive
PDHB	AP000894.4	0.425191414	2.27E-23	postive
PDHA1	AP001453.2	0.406709181	2.43E-21	postive
MTF1	AP001453.2	0.400127803	1.20E-20	postive
LIPT1	AP001462.1	0.419657799	9.49E-23	postive
PDHA1	AP001462.1	0.466515318	2.19E-28	postive
MTF1	AP001462.1	0.45613764	4.62E-27	postive
LIAS	AP002026.1	0.407295442	2.11E-21	postive
LIPT1	AP002026.1	0.443382074	1.71E-25	postive
FDX1	AP002387.2	0.424748091	2.55E-23	postive
LIPT1	AP002387.2	0.432425863	3.37E-24	postive
DLAT	AP002387.2	0.407806643	1.86E-21	postive
PDHA1	AP002387.2	0.51850831	9.41E-36	postive
PDHB	AP002387.2	0.478379886	5.87E-30	postive
MTF1	AP003170.1	0.449117242	3.43E-26	postive
MTF1	AP005131.6	0.407075604	2.22E-21	postive
LIAS	APBB3	0.404187361	4.50E-21	postive
LIPT1	APBB3	0.464839293	3.61E-28	postive
PDHA1	APBB3	0.482849847	1.45E-30	postive
MTF1	APBB3	0.428246492	1.02E-23	postive
MTF1	ARGFXP2	0.415018296	3.08E-22	postive
DLD	ARHGAP11A	0.53800666	7.36E-39	postive
DLAT	ARHGAP11A	0.536838109	1.15E-38	postive
PDHA1	ARHGAP11A	0.44694237	6.33E-26	postive
MTF1	ARHGAP11A	0.418095792	1.41E-22	postive
FDX1	ARHGAP18	0.418012541	1.44E-22	postive
LIPT1	ARHGAP18	0.415031569	3.07E-22	postive
DLD	ARHGAP18	0.401652919	8.31E-21	postive
DLAT	ARHGAP18	0.400337857	1.14E-20	postive
MTF1	ARHGAP18	0.503613827	1.64E-33	postive
GLS	ARHGAP18	0.450985552	2.02E-26	postive
FDX1	ARHGDIB	0.409231445	1.31E-21	postive
DLD	ARHGEF39	0.474252109	2.10E-29	postive
DLAT	ARHGEF39	0.460947985	1.14E-27	postive

PDHA1	ARHGEF39	0.564708452	1.86E-43	postive
PDHB	ARHGEF39	0.40014444	1.19E-20	postive
MTF1	ARHGEF6	0.529059099	2.08E-37	postive
GLS	ARHGEF6	0.446872134	6.46E-26	postive
LIAS	ARID4A	0.411840957	6.84E-22	postive
LIPT1	ARID4A	0.53706189	1.05E-38	postive
DLD	ARID4A	0.428060224	1.07E-23	postive
DLAT	ARID4A	0.470922633	5.80E-29	postive
PDHA1	ARID4A	0.425668536	2.01E-23	postive
PDHB	ARID4A	0.456486944	4.18E-27	postive
MTF1	ARID4A	0.654830607	1.50E-62	postive
GLS	ARID4A	0.405489592	3.28E-21	postive
FDX1	ARIH2OS	0.412535072	5.75E-22	postive
LIAS	ARIH2OS	0.491262264	9.80E-32	postive
LIPT1	ARIH2OS	0.575757545	1.75E-45	postive
PDHA1	ARIH2OS	0.540344436	3.03E-39	postive
PDHB	ARIH2OS	0.611275842	1.48E-52	postive
GLS	ARL2BPP4	0.411827416	6.86E-22	postive
DLD	ARNTL2	0.423709399	3.34E-23	postive
DLAT	ARRB1	0.446607529	6.96E-26	postive
PDHA1	ARRB1	0.426359258	1.67E-23	postive
PDHB	ARRB1	0.481806602	2.01E-30	postive
MTF1	ARRB1	0.427364957	1.29E-23	postive
LIPT1	ASAP3	0.407511753	2.00E-21	postive
PDHA1	ASAP3	0.432816669	3.03E-24	postive
MTF1	ASAP3	0.544889311	5.26E-40	postive
MTF1	ASB14	0.479610146	4.00E-30	postive
DLD	ASF1B	0.493038865	5.49E-32	postive
DLAT	ASF1B	0.457132908	3.47E-27	postive
PDHA1	ASF1B	0.487375737	3.43E-31	postive
LIAS	ASPH	0.401268522	9.12E-21	postive
DLD	ASPH	0.400946489	9.85E-21	postive
DLAT	ASPH	0.403134072	5.81E-21	postive
DLD	ASPM	0.4550599	6.31E-27	postive
DLAT	ASPM	0.435869943	1.33E-24	postive
LIPT1	ATAT1	0.461935854	8.52E-28	postive
DLAT	ATAT1	0.400886394	9.99E-21	postive
PDHA1	ATAT1	0.605485552	2.43E-51	postive
PDHB	ATAT1	0.477690174	7.27E-30	postive
MTF1	ATAT1	0.497913547	1.11E-32	postive
LIPT1	ATF7IP2	0.441955087	2.53E-25	postive
MTF1	ATF7IP2	0.431983406	3.79E-24	postive
PDHA1	ATG16L2	0.40727463	2.12E-21	postive
MTF1	ATG16L2	0.428635273	9.21E-24	postive
MTF1	ATP1B2	0.403465244	5.36E-21	postive
PDHA1	ATP5MC1P4	0.449001799	3.55E-26	postive
PDHB	ATP5MC1P4	0.411517376	7.41E-22	postive
MTF1	ATP6V1G2	0.408660566	1.51E-21	postive

MTF1	ATP8B2	0.540540301	2.81E-39	postive
GLS	ATP8B2	0.436976741	9.89E-25	postive
MTF1	ATP8B4	0.49667239	1.67E-32	postive
GLS	ATP8B4	0.51326033	5.97E-35	postive
LIPT1	ATXN1-AS1	0.49160603	8.76E-32	postive
MTF1	ATXN1-AS1	0.46790136	1.44E-28	postive
GLS	ATXN1-AS1	0.415041037	3.07E-22	postive
PDHA1	ATXN2-AS	0.454450247	7.52E-27	postive
DLD	AUNIP	0.531977636	7.07E-38	postive
DLAT	AUNIP	0.481162579	2.46E-30	postive
PDHA1	AUNIP	0.468279626	1.29E-28	postive
DLD	AURKB	0.454533121	7.34E-27	postive
PDHA1	AURKB	0.468264471	1.29E-28	postive
FDX1	AVEN	0.490035607	1.46E-31	postive
LIAS	AVEN	0.513218372	6.06E-35	postive
LIPT1	AVEN	0.400712782	1.04E-20	postive
DLD	AVEN	0.632270649	3.57E-57	postive
DLAT	AVEN	0.587479672	1.01E-47	postive
PDHA1	AVEN	0.537705764	8.25E-39	postive
PDHB	AVEN	0.664630734	4.93E-65	postive
FDX1	B4GALT1	0.473270876	2.84E-29	postive
DLD	B4GALT1	0.596389044	1.75E-49	postive
DLAT	B4GALT1	0.618417225	4.36E-54	postive
PDHA1	B4GALT1	0.535217171	2.11E-38	postive
PDHB	B4GALT1	0.499632033	6.24E-33	postive
MTF1	B4GALT1	0.586047478	1.92E-47	postive
FDX1	B4GALT4	0.427053415	1.40E-23	postive
DLD	B4GALT4	0.534159514	3.13E-38	postive
DLAT	B4GALT4	0.462546791	7.11E-28	postive
PDHA1	B4GALT4	0.493661886	4.48E-32	postive
MTF1	B4GALT4	0.50981359	1.97E-34	postive
DLD	BCAR3	0.47228782	3.83E-29	postive
DLAT	BCAR3	0.441629871	2.77E-25	postive
MTF1	BCAR3	0.457572173	3.05E-27	postive
MTF1	BCL2	0.407786157	1.87E-21	postive
FDX1	BDH2	0.4278694	1.13E-23	postive
LIAS	BDH2	0.535831294	1.67E-38	postive
LIPT1	BDH2	0.53282159	5.16E-38	postive
PDHA1	BDH2	0.423822695	3.24E-23	postive
PDHB	BDH2	0.51184562	9.76E-35	postive
DLD	BIRC5	0.455038853	6.35E-27	postive
DLAT	BIRC5	0.430741038	5.27E-24	postive
DLD	BRCA1	0.561360998	7.40E-43	postive
DLAT	BRCA1	0.53155678	8.26E-38	postive
PDHA1	BRCA1	0.459729771	1.63E-27	postive
MTF1	BRCA1	0.512984833	6.57E-35	postive
MTF1	BTBD9	0.548368231	1.36E-40	postive
GLS	BTK	0.432035846	3.74E-24	postive

LIPT1	BTN2A2	0.521739798	2.97E-36	postive
PDHA1	BTN2A2	0.453635495	9.50E-27	postive
MTF1	BTN2A2	0.543129244	1.04E-39	postive
GLS	BTN2A2	0.46275104	6.70E-28	postive
LIPT1	BTN2A3P	0.481756998	2.04E-30	postive
PDHA1	BTN2A3P	0.461509867	9.65E-28	postive
MTF1	BTN2A3P	0.557840138	3.11E-42	postive
DLD	BUB1	0.51669982	1.78E-35	postive
DLAT	BUB1	0.502380234	2.48E-33	postive
PDHA1	BUB1	0.412392279	5.96E-22	postive
DLD	BUB1B	0.537671401	8.36E-39	postive
DLAT	BUB1B	0.528523308	2.53E-37	postive
PDHA1	BUB1B	0.462586142	7.03E-28	postive
FDX1	BZW1	0.506610652	5.92E-34	postive
LIAS	BZW1	0.488830174	2.15E-31	postive
LIPT1	BZW1	0.474106314	2.20E-29	postive
DLD	BZW1	0.666941698	1.24E-65	postive
DLAT	BZW1	0.692242654	1.46E-72	postive
PDHA1	BZW1	0.462000423	8.36E-28	postive
PDHB	BZW1	0.619779197	2.20E-54	postive
MTF1	BZW1	0.478301248	6.01E-30	postive
FDX1	C10orf143	0.427738628	1.17E-23	postive
LIAS	C10orf143	0.463339148	5.63E-28	postive
LIPT1	C10orf143	0.467064199	1.86E-28	postive
LIAS	C12orf76	0.427910483	1.11E-23	postive
LIPT1	C12orf76	0.529458257	1.79E-37	postive
DLAT	C12orf76	0.41170109	7.08E-22	postive
PDHA1	C12orf76	0.484273256	9.22E-31	postive
PDHB	C12orf76	0.548248601	1.42E-40	postive
MTF1	C14orf132	0.500139143	5.27E-33	postive
LIAS	C19orf48	0.416330164	2.21E-22	postive
LIPT1	C19orf48	0.406633289	2.48E-21	postive
DLD	C19orf48	0.528783592	2.30E-37	postive
DLAT	C19orf48	0.499531575	6.45E-33	postive
PDHA1	C19orf48	0.596441938	1.70E-49	postive
PDHB	C19orf48	0.560366365	1.11E-42	postive
FDX1	C1orf210	0.432055655	3.72E-24	postive
LIAS	C1orf210	0.457826305	2.83E-27	postive
LIPT1	C1orf210	0.483550572	1.16E-30	postive
PDHA1	C1orf210	0.471311739	5.15E-29	postive
PDHB	C1orf210	0.556672744	4.98E-42	postive
LIPT1	C20orf194	0.448003296	4.70E-26	postive
MTF1	C20orf194	0.672903604	3.33E-67	postive
LIAS	C20orf96	0.401640288	8.34E-21	postive
LIPT1	C20orf96	0.49328043	5.08E-32	postive
PDHA1	C20orf96	0.573715101	4.19E-45	postive
PDHB	C20orf96	0.516275043	2.07E-35	postive
MTF1	C20orf96	0.416155214	2.31E-22	postive

FDX1	C3orf18	0.401716048	8.19E-21	postive
LIAS	C3orf18	0.466729569	2.05E-28	postive
LIPT1	C3orf18	0.51406496	4.50E-35	postive
DLAT	C3orf18	0.440414898	3.87E-25	postive
PDHA1	C3orf18	0.521750035	2.96E-36	postive
PDHB	C3orf18	0.604239803	4.40E-51	postive
MTF1	C3orf18	0.469562901	8.75E-29	postive
FDX1	C3orf62	0.429038106	8.28E-24	postive
LIAS	C3orf62	0.470554829	6.48E-29	postive
LIPT1	C3orf62	0.51662373	1.83E-35	postive
PDHA1	C3orf62	0.53944516	4.26E-39	postive
PDHB	C3orf62	0.538750636	5.55E-39	postive
MTF1	C3orf62	0.596675556	1.53E-49	postive
MTF1	C6orf201	0.459012971	2.01E-27	postive
LIPT1	CA5B	0.495823732	2.20E-32	postive
MTF1	CA5B	0.559231876	1.76E-42	postive
FDX1	CAMK1	0.415430342	2.78E-22	postive
LIAS	CAMK1	0.41373091	4.26E-22	postive
LIPT1	CAMK1	0.497679279	1.19E-32	postive
DLAT	CAMK1	0.417975742	1.46E-22	postive
PDHA1	CAMK1	0.442198771	2.37E-25	postive
PDHB	CAMK1	0.558799965	2.10E-42	postive
MTF1	CAMK1	0.430043269	6.34E-24	postive
GLS	CAMK1	0.481511849	2.20E-30	postive
PDHA1	CAMSAP3	0.542892954	1.14E-39	postive
PDHB	CAMSAP3	0.463744895	4.99E-28	postive
MTF1	CAMSAP3	0.457328293	3.27E-27	postive
LIPT1	CAMTA1-DT	0.407182292	2.17E-21	postive
PDHA1	CAMTA1-DT	0.440543743	3.74E-25	postive
FDX1	CARD8-AS1	0.423208641	3.80E-23	postive
LIPT1	CARD8-AS1	0.505485346	8.69E-34	postive
GLS	CARD8-AS1	0.400688757	1.05E-20	postive
LIPT1	CASP10	0.486948745	3.93E-31	postive
MTF1	CASP10	0.508422418	3.18E-34	postive
GLS	CASP10	0.463960353	4.68E-28	postive
MTF1	CASS4	0.469180436	9.82E-29	postive
PDHA1	CASZ1	0.460051136	1.48E-27	postive
MTF1	CASZ1	0.646305305	1.82E-60	postive
FDX1	CAT	0.51123614	1.21E-34	postive
LIAS	CAT	0.481972159	1.91E-30	postive
LIPT1	CAT	0.547089795	2.24E-40	postive
DLD	CAT	0.405172765	3.54E-21	postive
DLAT	CAT	0.423024527	3.99E-23	postive
PDHB	CAT	0.57311752	5.41E-45	postive
MTF1	CAT	0.436370811	1.17E-24	postive
LIPT1	CATSPERG	0.436876321	1.02E-24	postive
MTF1	CBFA2T3	0.410282952	1.01E-21	postive
LIPT1	CBR3-AS1	0.418072931	1.42E-22	postive

DLD	CBX5	0.495335433	2.59E-32	postive
DLAT	CBX5	0.536251501	1.43E-38	postive
PDHA1	CBX5	0.550274869	6.40E-41	postive
PDHB	CBX5	0.400840224	1.01E-20	postive
MTF1	CBX5	0.675855508	5.38E-68	postive
LIPT1	CBX7	0.427082045	1.39E-23	postive
MTF1	CBX7	0.517854506	1.19E-35	postive
MTF1	CCDC146	0.483932275	1.03E-30	postive
LIPT1	CCDC152	0.483677983	1.11E-30	postive
LIPT1	CCDC153	0.414700151	3.34E-22	postive
LIAS	CCDC159	0.449113304	3.44E-26	postive
LIPT1	CCDC159	0.500155378	5.24E-33	postive
PDHA1	CCDC159	0.50705181	5.09E-34	postive
PDHB	CCDC159	0.445046061	1.08E-25	postive
MTF1	CCDC180	0.416869003	1.93E-22	postive
PDHA1	CCDC189	0.419861185	9.01E-23	postive
LIPT1	CCDC191	0.406210831	2.75E-21	postive
MTF1	CCDC191	0.578613043	5.07E-46	postive
FDX1	CCDC28A	0.537435912	9.14E-39	postive
LIAS	CCDC28A	0.522618091	2.16E-36	postive
LIPT1	CCDC28A	0.571090578	1.28E-44	postive
DLD	CCDC28A	0.43239837	3.39E-24	postive
DLAT	CCDC28A	0.417568855	1.62E-22	postive
PDHA1	CCDC28A	0.472780001	3.29E-29	postive
PDHB	CCDC28A	0.589328711	4.40E-48	postive
MTF1	CCDC28A	0.406984311	2.27E-21	postive
LIPT1	CCDC30	0.418217308	1.37E-22	postive
MTF1	CCDC30	0.549211087	9.73E-41	postive
PDHB	CCDC69	0.40481685	3.86E-21	postive
MTF1	CCDC69	0.439778089	4.61E-25	postive
LIPT1	CCDC84	0.425966711	1.86E-23	postive
PDHA1	CCDC84	0.429955087	6.49E-24	postive
MTF1	CCDC84	0.407028494	2.25E-21	postive
PDHA1	CCDC85B	0.420715479	7.24E-23	postive
DLAT	CCDC88C	0.407669514	1.92E-21	postive
PDHA1	CCDC88C	0.522050342	2.66E-36	postive
MTF1	CCDC88C	0.645887241	2.30E-60	postive
DLD	CCNA2	0.527609388	3.54E-37	postive
DLAT	CCNA2	0.488344129	2.51E-31	postive
DLD	CCNB1	0.535222235	2.10E-38	postive
DLAT	CCNB1	0.512857256	6.87E-35	postive
PDHA1	CCNB1	0.465762379	2.74E-28	postive
PDHB	CCNB1	0.443299108	1.75E-25	postive
DLD	CCNB2	0.513342267	5.80E-35	postive
DLAT	CCNB2	0.487048227	3.81E-31	postive
PDHA1	CCNB2	0.435823883	1.35E-24	postive
DLD	CCNE1	0.483405906	1.21E-30	postive
PDHA1	CCNE1	0.466871917	1.97E-28	postive

CDKN2A	CCNE1	0.434486195	1.94E-24	postive
GLS	CCR2	0.403863714	4.87E-21	postive
FDX1	CCT6A	0.442959808	1.92E-25	postive
LIAS	CCT6A	0.496880119	1.56E-32	postive
LIPT1	CCT6A	0.43379998	2.33E-24	postive
DLD	CCT6A	0.649330667	3.38E-61	postive
DLAT	CCT6A	0.644181815	5.89E-60	postive
PDHA1	CCT6A	0.578868602	4.54E-46	postive
PDHB	CCT6A	0.551409055	4.09E-41	postive
MTF1	CCT6A	0.507256981	4.75E-34	postive
MTF1	CD160	0.482097189	1.83E-30	postive
MTF1	CD180	0.427392217	1.28E-23	postive
LIPT1	CD200R1	0.403547353	5.26E-21	postive
GLS	CD200R1	0.417626685	1.59E-22	postive
MTF1	CD226	0.428923165	8.53E-24	postive
LIPT1	CD244	0.416057537	2.37E-22	postive
MTF1	CD28	0.40454659	4.12E-21	postive
LIPT1	CD302	0.424295963	2.87E-23	postive
MTF1	CD302	0.403518404	5.29E-21	postive
DLD	CD3EAP	0.553344177	1.89E-41	postive
DLAT	CD3EAP	0.567263157	6.42E-44	postive
PDHA1	CD3EAP	0.604575303	3.75E-51	postive
PDHB	CD3EAP	0.525439242	7.80E-37	postive
MTF1	CD3EAP	0.504027809	1.42E-33	postive
LIPT1	CD83	0.440172096	4.14E-25	postive
MTF1	CD83	0.43658242	1.10E-24	postive
GLS	CD83	0.457878505	2.79E-27	postive
FDX1	CD9	0.443332953	1.73E-25	postive
LIPT1	CD9	0.441841088	2.61E-25	postive
DLD	CD9	0.410099538	1.05E-21	postive
DLAT	CD9	0.431025292	4.89E-24	postive
PDHB	CD9	0.50312775	1.93E-33	postive
FDX1	CDADC1	0.570873846	1.40E-44	postive
LIAS	CDADC1	0.565608872	1.28E-43	postive
LIPT1	CDADC1	0.646029361	2.13E-60	postive
DLD	CDADC1	0.562518564	4.60E-43	postive
DLAT	CDADC1	0.573787798	4.06E-45	postive
PDHA1	CDADC1	0.595589371	2.53E-49	postive
PDHB	CDADC1	0.629127195	1.85E-56	postive
MTF1	CDADC1	0.612112293	9.86E-53	postive
LIPT1	CDC14A	0.454831151	6.74E-27	postive
MTF1	CDC14A	0.540830222	2.51E-39	postive
DLD	CDC25C	0.445185717	1.03E-25	postive
DLAT	CDC25C	0.437638539	8.26E-25	postive
PDHA1	CDC25C	0.439628768	4.80E-25	postive
FDX1	CDC37L1	0.489194104	1.91E-31	postive
LIAS	CDC37L1	0.510258	1.69E-34	postive
LIPT1	CDC37L1	0.609039223	4.40E-52	postive

DLD	CDC37L1	0.537735904	8.16E-39	postive
DLAT	CDC37L1	0.549237217	9.63E-41	postive
PDHA1	CDC37L1	0.501592291	3.24E-33	postive
PDHB	CDC37L1	0.641633384	2.38E-59	postive
MTF1	CDC37L1	0.548776789	1.15E-40	postive
DLD	CDC45	0.500840606	4.17E-33	postive
DLAT	CDC45	0.449093061	3.46E-26	postive
PDHA1	CDC45	0.466304886	2.33E-28	postive
DLD	CDC6	0.537210779	9.95E-39	postive
DLAT	CDC6	0.505163332	9.69E-34	postive
PDHA1	CDC6	0.427397596	1.28E-23	postive
DLD	CDCA2	0.480484948	3.04E-30	postive
DLAT	CDCA2	0.473462595	2.67E-29	postive
PDHA1	CDCA2	0.401202968	9.26E-21	postive
DLD	CDCA3	0.452679339	1.25E-26	postive
PDHA1	CDCA3	0.489765456	1.59E-31	postive
DLD	CDCA5	0.529218233	1.96E-37	postive
DLAT	CDCA5	0.502803006	2.15E-33	postive
PDHA1	CDCA5	0.476854089	9.42E-30	postive
DLD	CDCA8	0.511702181	1.03E-34	postive
DLAT	CDCA8	0.488173613	2.66E-31	postive
PDHA1	CDCA8	0.472662607	3.41E-29	postive
MTF1	CDCP1	0.415483374	2.74E-22	postive
MTF1	CDH23	0.482537037	1.60E-30	postive
DLD	CDK1	0.489073356	1.99E-31	postive
DLAT	CDK1	0.450541637	2.30E-26	postive
GLS	CDK15	0.439592222	4.85E-25	postive
GLS	CDKL2	0.438235394	7.02E-25	postive
CDKN2A	CDKN2A-DT	0.754864917	2.84E-93	postive
DLD	CDKN3	0.438024897	7.44E-25	postive
DLD	CENPA	0.446910446	6.39E-26	postive
DLAT	CENPA	0.417675361	1.57E-22	postive
FDX1	CENPC	0.427200321	1.34E-23	postive
LIAS	CENPC	0.516834114	1.70E-35	postive
LIPT1	CENPC	0.603759399	5.52E-51	postive
DLD	CENPC	0.464137292	4.44E-28	postive
DLAT	CENPC	0.516042907	2.25E-35	postive
PDHA1	CENPC	0.458087271	2.63E-27	postive
PDHB	CENPC	0.468082176	1.37E-28	postive
MTF1	CENPC	0.6789257	7.90E-69	postive
DLD	CENPE	0.463046756	6.14E-28	postive
DLAT	CENPE	0.455278927	5.92E-27	postive
PDHA1	CENPE	0.410911584	8.62E-22	postive
DLD	CENPF	0.449758021	2.87E-26	postive
DLAT	CENPF	0.458489321	2.34E-27	postive
PDHA1	CENPF	0.437361315	8.91E-25	postive
MTF1	CENPF	0.412520184	5.77E-22	postive
LIAS	CENPH	0.405049776	3.65E-21	postive

DLD	CENPH	0.597431534	1.08E-49	postive
DLAT	CENPH	0.546343169	2.99E-40	postive
PDHA1	CENPH	0.542727976	1.21E-39	postive
PDHB	CENPH	0.483034373	1.36E-30	postive
DLD	CENPK	0.4700273	7.61E-29	postive
DLAT	CENPK	0.430246184	6.01E-24	postive
DLD	CENPM	0.491934219	7.88E-32	postive
DLAT	CENPM	0.428380113	9.85E-24	postive
PDHA1	CENPM	0.510271705	1.68E-34	postive
DLD	CENPU	0.513021694	6.49E-35	postive
DLAT	CENPU	0.472730894	3.34E-29	postive
PDHA1	CENPU	0.415312542	2.86E-22	postive
DLD	CEP55	0.487067198	3.79E-31	postive
DLAT	CEP55	0.460650452	1.24E-27	postive
LIPT1	CEP68	0.528933147	2.18E-37	postive
DLD	CEP68	0.489296086	1.85E-31	postive
DLAT	CEP68	0.521005126	3.86E-36	postive
PDHA1	CEP68	0.496878265	1.56E-32	postive
PDHB	CEP68	0.482230552	1.76E-30	postive
MTF1	CEP68	0.697890185	3.29E-74	postive
LIPT1	CEP70	0.494914644	2.97E-32	postive
PDHA1	CEP70	0.454820025	6.76E-27	postive
PDHB	CEP70	0.459899376	1.55E-27	postive
MTF1	CEP70	0.448816689	3.74E-26	postive
GLS	CERKL	0.425669964	2.01E-23	postive
PDHA1	CERS4	0.407881788	1.82E-21	postive
GLS	CH25H	0.432246917	3.53E-24	postive
MTF1	CHAD	0.421741332	5.56E-23	postive
PDHA1	CHADL	0.427771033	1.16E-23	postive
PDHB	CHADL	0.430989398	4.94E-24	postive
LIPT1	CHAF1B	0.416682959	2.02E-22	postive
DLD	CHAF1B	0.578453113	5.44E-46	postive
DLAT	CHAF1B	0.55605489	6.39E-42	postive
PDHA1	CHAF1B	0.585942719	2.01E-47	postive
PDHB	CHAF1B	0.408629523	1.52E-21	postive
MTF1	CHAF1B	0.466158083	2.43E-28	postive
DLD	CHEK1	0.555122014	9.29E-42	postive
DLAT	CHEK1	0.613862663	4.18E-53	postive
PDHA1	CHEK1	0.488238302	2.60E-31	postive
LIPT1	CHKA	0.450177113	2.55E-26	postive
PDHA1	CHKA	0.56384768	2.66E-43	postive
MTF1	CHKA	0.434311626	2.03E-24	postive
MTF1	chr22-38_28785274-29006793.1	0.452703124	1.24E-26	postive
MTF1	CIDEB	0.444838936	1.14E-25	postive
MTF1	CIITA	0.508780463	2.82E-34	postive
LIAS	CIRBP	0.428609599	9.27E-24	postive

LIPT1	CIRBP	0.459807278	1.59E-27	postive
PDHA1	CIRBP	0.485389815	6.47E-31	postive
PDHB	CIRBP	0.482520448	1.60E-30	postive
MTF1	CIRBP	0.50094823	4.02E-33	postive
PDHB	CISH	0.431589286	4.21E-24	postive
DLD	CKAP2L	0.500766611	4.27E-33	postive
DLAT	CKAP2L	0.484379369	8.91E-31	postive
DLD	CKAP4	0.579534914	3.40E-46	postive
DLAT	CKAP4	0.54378814	8.06E-40	postive
PDHA1	CKAP4	0.568586607	3.69E-44	postive
PDHB	CKAP4	0.478867453	5.04E-30	postive
MTF1	CKAP4	0.503155289	1.91E-33	postive
FDX1	CKS1B	0.400922519	9.91E-21	postive
LIAS	CKS1B	0.444811727	1.15E-25	postive
LIPT1	CKS1B	0.461199525	1.06E-27	postive
DLD	CKS1B	0.613825759	4.26E-53	postive
DLAT	CKS1B	0.554707811	1.10E-41	postive
PDHA1	CKS1B	0.511177889	1.23E-34	postive
PDHB	CKS1B	0.541806194	1.73E-39	postive
GLS	CLEC7A	0.433760562	2.35E-24	postive
LIAS	CLHC1	0.443861569	1.50E-25	postive
LIPT1	CLHC1	0.453578377	9.66E-27	postive
MTF1	CLHC1	0.441801106	2.64E-25	postive
LIPT1	CLUL1	0.454040679	8.46E-27	postive
LIPT1	CMTM7	0.429805614	6.76E-24	postive
PDHB	CMTM7	0.424313801	2.86E-23	postive
MTF1	CMTM7	0.464027823	4.59E-28	postive
LIAS	CNKSR1	0.466776357	2.02E-28	postive
LIPT1	CNKSR1	0.45918933	1.91E-27	postive
PDHA1	CNKSR1	0.554998652	9.76E-42	postive
PDHB	CNKSR1	0.463885918	4.79E-28	postive
MTF1	CNKSR1	0.476442975	1.07E-29	postive
MTF1	COBLL1	0.55849065	2.39E-42	postive
GLS	CPED1	0.424826111	2.50E-23	postive
MTF1	CR1	0.421781695	5.50E-23	postive
FDX1	CRBN	0.528041386	3.02E-37	postive
LIAS	CRBN	0.582520034	9.17E-47	postive
LIPT1	CRBN	0.680949475	2.20E-69	postive
DLD	CRBN	0.440251245	4.05E-25	postive
DLAT	CRBN	0.478867241	5.04E-30	postive
PDHA1	CRBN	0.402753635	6.37E-21	postive
PDHB	CRBN	0.670165889	1.77E-66	postive
MTF1	CRBN	0.420603448	7.45E-23	postive
LIPT1	CREBRF	0.500270479	5.04E-33	postive
PDHB	CREBRF	0.412827096	5.35E-22	postive
MTF1	CREBRF	0.536104857	1.51E-38	postive
GLS	CREBRF	0.408062555	1.74E-21	postive
LIPT1	CRY2	0.424589148	2.66E-23	postive

PDHA1	CRY2	0.439598729	4.84E-25	postive
PDHB	CRY2	0.452590959	1.28E-26	postive
MTF1	CRY2	0.582048153	1.13E-46	postive
FDX1	CRYL1	0.532168255	6.58E-38	postive
LIAS	CRYL1	0.516652301	1.81E-35	postive
LIPT1	CRYL1	0.536581587	1.26E-38	postive
DLD	CRYL1	0.422641477	4.41E-23	postive
DLAT	CRYL1	0.42115729	6.46E-23	postive
PDHB	CRYL1	0.578444731	5.46E-46	postive
MTF1	CSF2RB	0.49400341	4.01E-32	postive
MTF1	CTC-325H20.2	0.443388335	1.71E-25	postive
FDX1	CTSF	0.431769868	4.01E-24	postive
LIPT1	CTSF	0.444574731	1.23E-25	postive
PDHA1	CTSF	0.452829301	1.20E-26	postive
PDHB	CTSF	0.438904707	5.85E-25	postive
FDX1	CTSZ	0.437929675	7.63E-25	postive
LIPT1	CTSZ	0.406820326	2.37E-21	postive
DLD	CTSZ	0.433413039	2.58E-24	postive
DLAT	CTSZ	0.411719991	7.05E-22	postive
PDHA1	CTSZ	0.504016097	1.43E-33	postive
PDHB	CTSZ	0.499597754	6.31E-33	postive
MTF1	CTSZ	0.52210147	2.61E-36	postive
LIAS	CUL9	0.425580342	2.05E-23	postive
LIPT1	CUL9	0.504730364	1.12E-33	postive
DLD	CUL9	0.455404522	5.71E-27	postive
DLAT	CUL9	0.451828797	1.59E-26	postive
PDHA1	CUL9	0.616421873	1.18E-53	postive
PDHB	CUL9	0.475639082	1.37E-29	postive
MTF1	CUL9	0.700803285	4.50E-75	postive
GLS	CXorf21	0.407636816	1.94E-21	postive
LIAS	CYB5A	0.41314066	4.94E-22	postive
LIPT1	CYB5A	0.45423831	7.99E-27	postive
PDHB	CYB5A	0.465925858	2.61E-28	postive
LIAS	CYB5RL	0.444066524	1.41E-25	postive
LIPT1	CYB5RL	0.585603655	2.34E-47	postive
DLD	CYB5RL	0.468836076	1.09E-28	postive
DLAT	CYB5RL	0.505496397	8.66E-34	postive
PDHA1	CYB5RL	0.518472406	9.53E-36	postive
PDHB	CYB5RL	0.409869477	1.12E-21	postive
MTF1	CYB5RL	0.703068324	9.41E-76	postive
FDX1	CYCS	0.526318185	5.67E-37	postive
LIAS	CYCS	0.487073119	3.78E-31	postive
LIPT1	CYCS	0.476921714	9.22E-30	postive
DLD	CYCS	0.630987162	7.00E-57	postive
DLAT	CYCS	0.615047443	2.33E-53	postive
PDHA1	CYCS	0.533936205	3.41E-38	postive
PDHB	CYCS	0.597991796	8.31E-50	postive
FDX1	CYLD	0.400352741	1.14E-20	postive

LIPT1	CYLD	0.449674978	2.93E-26	postive
MTF1	CYLD	0.666489215	1.63E-65	postive
GLS	CYLD	0.432497709	3.30E-24	postive
FDX1	CYP20A1	0.566728426	8.03E-44	postive
LIAS	CYP20A1	0.55265707	2.49E-41	postive
LIPT1	CYP20A1	0.671355373	8.59E-67	postive
DLD	CYP20A1	0.539399046	4.34E-39	postive
DLAT	CYP20A1	0.600708455	2.33E-50	postive
PDHA1	CYP20A1	0.521068692	3.78E-36	postive
PDHB	CYP20A1	0.618948787	3.34E-54	postive
MTF1	CYP20A1	0.618079704	5.16E-54	postive
LIPT1	CYP27A1	0.415060937	3.05E-22	postive
LIPT1	CYP2U1	0.442786373	2.01E-25	postive
MTF1	CYP2U1	0.526120181	6.09E-37	postive
GLS	CYP2U1	0.421803175	5.47E-23	postive
MTF1	CYSLTR2	0.517899344	1.17E-35	postive
MTF1	DAAM2	0.427164576	1.36E-23	postive
MTF1	DAAM2-AS1	0.405903069	2.96E-21	postive
LIPT1	DAPK2	0.405109511	3.60E-21	postive
FDX1	DARS2	0.429329699	7.66E-24	postive
LIAS	DARS2	0.539606342	4.01E-39	postive
LIPT1	DARS2	0.48176188	2.04E-30	postive
DLD	DARS2	0.720915319	2.44E-81	postive
DLAT	DARS2	0.731579254	6.85E-85	postive
PDHA1	DARS2	0.60372228	5.62E-51	postive
PDHB	DARS2	0.643613012	8.05E-60	postive
MTF1	DARS2	0.481903711	1.95E-30	postive
MTF1	DBH	0.412427017	5.91E-22	postive
LIPT1	DBP	0.490889713	1.11E-31	postive
PDHA1	DBP	0.524582823	1.06E-36	postive
PDHB	DBP	0.42384869	3.22E-23	postive
DLD	DDIAS	0.521228519	3.57E-36	postive
DLAT	DDIAS	0.504581275	1.18E-33	postive
LIPT1	DDX39B	0.417890247	1.49E-22	postive
PDHA1	DDX39B	0.514321975	4.12E-35	postive
MTF1	DDX39B	0.471916192	4.29E-29	postive
LIPT1	DEF6	0.43448031	1.94E-24	postive
PDHA1	DEF6	0.488835443	2.15E-31	postive
MTF1	DEF6	0.491806477	8.21E-32	postive
FDX1	DENND1C	0.473286488	2.82E-29	postive
LIAS	DENND1C	0.412060142	6.48E-22	postive
LIPT1	DENND1C	0.459310143	1.84E-27	postive
DLD	DENND1C	0.420805528	7.07E-23	postive
DLAT	DENND1C	0.432225274	3.55E-24	postive
PDHA1	DENND1C	0.534842401	2.43E-38	postive
PDHB	DENND1C	0.50414473	1.37E-33	postive
MTF1	DENND1C	0.614374544	3.25E-53	postive
DLD	DEPDC1	0.484626495	8.24E-31	postive

DLAT	DEPDC1	0.490916464	1.10E-31	postive
DLD	DEPDC1B	0.449251891	3.31E-26	postive
DLAT	DEPDC1B	0.430356985	5.84E-24	postive
LIPT1	DHDH	0.410308804	1.00E-21	postive
FDX1	DHRS1	0.493335048	4.99E-32	postive
LIAS	DHRS1	0.569788941	2.22E-44	postive
LIPT1	DHRS1	0.500502496	4.67E-33	postive
PDHA1	DHRS1	0.576172363	1.46E-45	postive
PDHB	DHRS1	0.575490845	1.96E-45	postive
MTF1	DHRS1	0.446388304	7.40E-26	postive
LIPT1	DHRS4-AS1	0.484007121	1.00E-30	postive
DLAT	DHRS4-AS1	0.453848999	8.93E-27	postive
PDHA1	DHRS4-AS1	0.461765221	8.95E-28	postive
PDHB	DHRS4-AS1	0.603218716	7.14E-51	postive
DLD	DIAPH3	0.546274244	3.07E-40	postive
DLAT	DIAPH3	0.513679303	5.15E-35	postive
PDHA1	DLG4	0.408490951	1.57E-21	postive
PDHB	DLG4	0.415462254	2.76E-22	postive
MTF1	DLG4	0.511955381	9.40E-35	postive
LIAS	DLGAP1-AS2	0.415652598	2.63E-22	postive
LIPT1	DLGAP4-AS1	0.411931037	6.69E-22	postive
DLD	DLGAP5	0.471955447	4.24E-29	postive
DLAT	DLGAP5	0.445692079	8.99E-26	postive
PDHA1	DMTN	0.432755887	3.08E-24	postive
PDHB	DMTN	0.490231889	1.37E-31	postive
MTF1	DNAH5	0.420777536	7.12E-23	postive
LIPT1	DNAJC27	0.565974893	1.10E-43	postive
DLD	DNAJC27	0.438934802	5.80E-25	postive
DLAT	DNAJC27	0.500204419	5.15E-33	postive
PDHA1	DNAJC27	0.524637142	1.04E-36	postive
PDHB	DNAJC27	0.476658155	1.00E-29	postive
MTF1	DNAJC27	0.692342084	1.36E-72	postive
LIAS	DNAJC28	0.511154852	1.24E-34	postive
LIPT1	DNAJC28	0.581045732	1.75E-46	postive
PDHA1	DNAJC28	0.4262627	1.72E-23	postive
PDHB	DNAJC28	0.452406937	1.35E-26	postive
MTF1	DNAJC28	0.455820613	5.07E-27	postive
DLAT	DOCK1	0.407741994	1.89E-21	postive
PDHA1	DOCK1	0.495527329	2.43E-32	postive
PDHB	DOCK1	0.410540204	9.45E-22	postive
MTF1	DOCK1	0.580849311	1.91E-46	postive
LIPT1	DOCK4	0.408124883	1.72E-21	postive
MTF1	DOCK4	0.62551143	1.20E-55	postive
MTF1	DOCK8	0.554047315	1.43E-41	postive
GLS	DOCK8	0.406009836	2.89E-21	postive
LIPT1	DOCK9-DT	0.410850076	8.75E-22	postive
FDX1	DOK1	0.453611363	9.56E-27	postive
LIAS	DOK1	0.440507723	3.77E-25	postive

LIPT1	DOK1	0.531684247	7.88E-38	postive
DLD	DOK1	0.42309417	3.92E-23	postive
PDHA1	DOK1	0.493314879	5.02E-32	postive
PDHB	DOK1	0.562178963	5.29E-43	postive
MTF1	DOK1	0.436188395	1.22E-24	postive
LIPT1	DOP1A	0.419461994	9.98E-23	postive
DLAT	DOP1A	0.407677937	1.92E-21	postive
PDHA1	DOP1A	0.435992487	1.29E-24	postive
MTF1	DOP1A	0.688830158	1.38E-71	postive
DLAT	DPY19L1	0.40990881	1.11E-21	postive
GLS	DPY19L1	0.506421024	6.32E-34	postive
MTF1	DPYSL2	0.49202764	7.64E-32	postive
PDHB	DRAM1	0.410282133	1.01E-21	postive
MTF1	DRC3	0.447234198	5.84E-26	postive
DLD	DSC2	0.415094737	3.03E-22	postive
DLAT	DSC2	0.477487048	7.74E-30	postive
MTF1	DSC2	0.439627656	4.80E-25	postive
DLD	DSCC1	0.536329942	1.39E-38	postive
DLAT	DSCC1	0.519491927	6.63E-36	postive
PDHA1	DSCC1	0.42645447	1.63E-23	postive
DLD	DSG2	0.511366056	1.15E-34	postive
DLAT	DSG2	0.544933676	5.17E-40	postive
MTF1	DSG2	0.444600977	1.22E-25	postive
DLAT	DSP	0.439459379	5.03E-25	postive
FDX1	DTYMK	0.445132874	1.05E-25	postive
LIAS	DTYMK	0.497003168	1.49E-32	postive
LIPT1	DTYMK	0.505338344	9.13E-34	postive
DLD	DTYMK	0.585490009	2.46E-47	postive
DLAT	DTYMK	0.559436619	1.62E-42	postive
PDHA1	DTYMK	0.574967215	2.45E-45	postive
PDHB	DTYMK	0.591461901	1.67E-48	postive
FDX1	DUSP28	0.478265725	6.08E-30	postive
LIAS	DUSP28	0.550602478	5.62E-41	postive
LIPT1	DUSP28	0.661016408	4.16E-64	postive
DLD	DUSP28	0.453230347	1.07E-26	postive
DLAT	DUSP28	0.426933059	1.44E-23	postive
PDHA1	DUSP28	0.559082478	1.88E-42	postive
PDHB	DUSP28	0.53147726	8.51E-38	postive
MTF1	DUSP28	0.477185354	8.50E-30	postive
DLD	E2F7	0.441358911	2.99E-25	postive
MTF1	E2F7	0.40393265	4.79E-21	postive
FDX1	EAF2	0.410409223	9.77E-22	postive
LIPT1	EAF2	0.550278375	6.39E-41	postive
LIAS	EBLN3P	0.407395137	2.06E-21	postive
LIPT1	EBLN3P	0.567265456	6.41E-44	postive
DLD	EBLN3P	0.525388624	7.95E-37	postive
DLAT	EBLN3P	0.549437896	8.90E-41	postive
PDHA1	EBLN3P	0.477395636	7.96E-30	postive

PDHB	EBLN3P	0.551279116	4.30E-41	postive
MTF1	EBLN3P	0.574829928	2.60E-45	postive
DLD	ECT2	0.596169291	1.93E-49	postive
DLAT	ECT2	0.598558198	6.38E-50	postive
PDHA1	ECT2	0.418828471	1.17E-22	postive
PDHB	ECT2	0.417231616	1.76E-22	postive
MTF1	ECT2	0.418695602	1.21E-22	postive
LIPT1	EFHC1	0.48457509	8.38E-31	postive
MTF1	EFHC1	0.501790975	3.03E-33	postive
FDX1	EGLN1	0.563390843	3.21E-43	postive
LIAS	EGLN1	0.527526244	3.65E-37	postive
LIPT1	EGLN1	0.481629226	2.12E-30	postive
DLD	EGLN1	0.619113304	3.08E-54	postive
DLAT	EGLN1	0.620983947	1.20E-54	postive
PDHA1	EGLN1	0.507458288	4.43E-34	postive
PDHB	EGLN1	0.563833052	2.67E-43	postive
MTF1	EGLN1	0.533958511	3.38E-38	postive
MTF1	EGR2	0.407827332	1.85E-21	postive
FDX1	EHBP1	0.41434568	3.65E-22	postive
LIAS	EHBP1	0.445958188	8.34E-26	postive
LIPT1	EHBP1	0.430065602	6.31E-24	postive
DLD	EHBP1	0.646907454	1.31E-60	postive
DLAT	EHBP1	0.63190551	4.33E-57	postive
PDHA1	EHBP1	0.527627523	3.51E-37	postive
PDHB	EHBP1	0.505112713	9.86E-34	postive
MTF1	EHBP1	0.609879495	2.93E-52	postive
FDX1	EIF3B	0.418001605	1.45E-22	postive
LIAS	EIF3B	0.467873552	1.46E-28	postive
DLD	EIF3B	0.63308724	2.32E-57	postive
DLAT	EIF3B	0.621734955	8.21E-55	postive
PDHA1	EIF3B	0.682934669	6.23E-70	postive
PDHB	EIF3B	0.554750198	1.08E-41	postive
MTF1	EIF3B	0.594127033	4.95E-49	postive
LIAS	ELOA-AS1	0.506133812	6.97E-34	postive
LIPT1	ELOA-AS1	0.551029574	4.75E-41	postive
PDHA1	ELOA-AS1	0.465261419	3.18E-28	postive
PDHB	ELOA-AS1	0.40324475	5.66E-21	postive
FDX1	EMC6	0.434115693	2.14E-24	postive
DLD	EMC6	0.48943854	1.77E-31	postive
DLAT	EMC6	0.456027408	4.77E-27	postive
PDHA1	EMC6	0.544484362	6.16E-40	postive
PDHB	EMC6	0.566731063	8.02E-44	postive
LIPT1	EMID1	0.415085931	3.03E-22	postive
PDHA1	ENO3	0.434162701	2.11E-24	postive
FDX1	ENPP4	0.407526013	1.99E-21	postive
LIPT1	ENPP4	0.526713548	4.91E-37	postive
DLAT	ENPP4	0.44627384	7.64E-26	postive
PDHB	ENPP4	0.434046254	2.18E-24	postive

MTF1	ENPP4	0.430741724	5.27E-24	postive
LIPT1	ENPP5	0.448399432	4.21E-26	postive
MTF1	EP300-AS1	0.46149739	9.69E-28	postive
LIPT1	EPB41	0.458612542	2.25E-27	postive
DLD	EPB41	0.491249794	9.84E-32	postive
DLAT	EPB41	0.522896543	1.96E-36	postive
PDHA1	EPB41	0.541002697	2.35E-39	postive
PDHB	EPB41	0.410980352	8.48E-22	postive
MTF1	EPB41	0.727374956	1.80E-83	postive
FDX1	EPB41L5	0.412251687	6.17E-22	postive
LIPT1	EPB41L5	0.559803421	1.40E-42	postive
DLD	EPB41L5	0.415901673	2.47E-22	postive
DLAT	EPB41L5	0.522728358	2.08E-36	postive
PDHA1	EPB41L5	0.48813576	2.69E-31	postive
PDHB	EPB41L5	0.517548998	1.32E-35	postive
MTF1	EPB41L5	0.444730601	1.17E-25	postive
FDX1	EPC1	0.442100624	2.43E-25	postive
LIAS	EPC1	0.478638719	5.41E-30	postive
LIPT1	EPC1	0.594681247	3.84E-49	postive
DLD	EPC1	0.507322853	4.64E-34	postive
DLAT	EPC1	0.56562119	1.27E-43	postive
PDHA1	EPC1	0.549359299	9.18E-41	postive
PDHB	EPC1	0.578348535	5.69E-46	postive
MTF1	EPC1	0.688116831	2.20E-71	postive
LIPT1	EPCAM-DT	0.416833467	1.95E-22	postive
DLD	EPG5	0.503247019	1.85E-33	postive
DLAT	EPG5	0.518321568	1.01E-35	postive
PDHA1	EPG5	0.507200728	4.84E-34	postive
PDHB	EPG5	0.42024691	8.16E-23	postive
MTF1	EPG5	0.738360738	3.07E-87	postive
LIAS	EPHX1	0.426385548	1.66E-23	postive
PDHB	EPHX1	0.479446316	4.21E-30	postive
LIAS	EPHX2	0.436305556	1.19E-24	postive
PDHB	EPHX2	0.410041956	1.07E-21	postive
MTF1	EPX	0.480808744	2.75E-30	postive
DLD	ERCC6L	0.519896947	5.74E-36	postive
DLAT	ERCC6L	0.485505758	6.23E-31	postive
PDHA1	ERCC6L	0.428458275	9.65E-24	postive
MTF1	ERG	0.517666147	1.27E-35	postive
DLD	ERO1A	0.478777215	5.18E-30	postive
DLAT	ERO1A	0.479997724	3.54E-30	postive
PDHB	ERO1B	0.405434074	3.32E-21	postive
DLD	ESCO2	0.535100616	2.20E-38	postive
DLAT	ESCO2	0.510908154	1.35E-34	postive
DLD	ESPL1	0.476420249	1.08E-29	postive
DLAT	ESPL1	0.440404339	3.88E-25	postive
PDHA1	ESPL1	0.500716882	4.34E-33	postive
PDHA1	ETV5	0.403973917	4.74E-21	postive

PDHB	ETV5	0.456091558	4.69E-27	postive
MTF1	ETV5	0.449109102	3.44E-26	postive
DLD	EXO1	0.539660768	3.93E-39	postive
DLAT	EXO1	0.518489868	9.47E-36	postive
PDHA1	EXO1	0.428934479	8.51E-24	postive
DLAT	EXPH5	0.427823429	1.14E-23	postive
MTF1	EXPH5	0.47564031	1.37E-29	postive
FDX1	EXT1	0.424466663	2.74E-23	postive
LIPT1	EXT1	0.406180285	2.77E-21	postive
DLD	EXT1	0.526904841	4.58E-37	postive
DLAT	EXT1	0.543885723	7.76E-40	postive
PDHA1	EXT1	0.504500239	1.21E-33	postive
PDHB	EXT1	0.46049033	1.30E-27	postive
MTF1	EXT1	0.581314184	1.56E-46	postive
FDX1	FAAH	0.418069484	1.42E-22	postive
LIAS	FAAH	0.410146446	1.04E-21	postive
LIPT1	FAAH	0.503672112	1.61E-33	postive
PDHA1	FAAH	0.522637426	2.15E-36	postive
PDHB	FAAH	0.469300988	9.47E-29	postive
MTF1	FAAH	0.414679114	3.36E-22	postive
LIAS	FADS3	0.403998457	4.71E-21	postive
LIPT1	FADS3	0.476420927	1.08E-29	postive
PDHA1	FADS3	0.482936234	1.41E-30	postive
PDHB	FADS3	0.425871995	1.90E-23	postive
MTF1	FADS3	0.410996258	8.44E-22	postive
LIPT1	FAM117A	0.526215535	5.88E-37	postive
PDHA1	FAM117A	0.486519934	4.51E-31	postive
PDHB	FAM117A	0.475575935	1.40E-29	postive
MTF1	FAM117A	0.483236788	1.28E-30	postive
LIPT1	FAM13B	0.508307695	3.31E-34	postive
DLAT	FAM13B	0.421780894	5.50E-23	postive
PDHB	FAM13B	0.40242742	6.89E-21	postive
MTF1	FAM13B	0.670557283	1.40E-66	postive
PDHA1	FAM174B	0.453281714	1.05E-26	postive
PDHB	FAM174B	0.421549595	5.84E-23	postive
PDHA1	FAM184B	0.413109389	4.98E-22	postive
PDHA1	FAM186B	0.459793581	1.60E-27	postive
MTF1	FAM186B	0.456956196	3.65E-27	postive
FDX1	FAM207BP	0.417329855	1.72E-22	postive
LIAS	FAM207BP	0.440383217	3.90E-25	postive
LIPT1	FAM207BP	0.442699363	2.06E-25	postive
DLD	FAM207BP	0.466048802	2.51E-28	postive
DLAT	FAM207BP	0.465777814	2.73E-28	postive
PDHA1	FAM207BP	0.474201723	2.13E-29	postive
PDHB	FAM207BP	0.476970232	9.09E-30	postive
FDX1	FAM210B	0.441817019	2.63E-25	postive
LIAS	FAM210B	0.441663872	2.75E-25	postive
LIPT1	FAM210B	0.511554855	1.08E-34	postive

DLD	FAM210B	0.458075231	2.64E-27	postive
DLAT	FAM210B	0.461994022	8.37E-28	postive
PDHA1	FAM210B	0.538707551	5.64E-39	postive
PDHB	FAM210B	0.597964266	8.41E-50	postive
MTF1	FAM210B	0.477793293	7.04E-30	postive
FDX1	FAM217B	0.481086417	2.52E-30	postive
LIAS	FAM217B	0.452905628	1.17E-26	postive
LIPT1	FAM217B	0.616355755	1.22E-53	postive
DLD	FAM217B	0.583797134	5.22E-47	postive
DLAT	FAM217B	0.611788161	1.16E-52	postive
PDHA1	FAM217B	0.474140977	2.17E-29	postive
PDHB	FAM217B	0.54422384	6.81E-40	postive
MTF1	FAM217B	0.569376678	2.64E-44	postive
LIPT1	FAM21FP	0.492974322	5.61E-32	postive
MTF1	FAM49A	0.48832772	2.53E-31	postive
GLS	FAM49A	0.407792749	1.86E-21	postive
DLD	FAM72A	0.509437406	2.25E-34	postive
DLAT	FAM72A	0.481426208	2.26E-30	postive
PDHA1	FAM72A	0.464351895	4.17E-28	postive
DLD	FAM72B	0.489019417	2.02E-31	postive
DLAT	FAM72B	0.480577752	2.95E-30	postive
PDHA1	FAM72B	0.423665662	3.38E-23	postive
DLD	FAM72D	0.437106032	9.55E-25	postive
FDX1	FAM76A	0.503146664	1.92E-33	postive
LIAS	FAM76A	0.519651974	6.26E-36	postive
LIPT1	FAM76A	0.675005455	9.12E-68	postive
DLD	FAM76A	0.435905731	1.32E-24	postive
DLAT	FAM76A	0.49031218	1.33E-31	postive
PDHA1	FAM76A	0.467592055	1.58E-28	postive
PDHB	FAM76A	0.519344292	6.99E-36	postive
MTF1	FAM76A	0.595857212	2.23E-49	postive
GLS	FAM76A	0.420032112	8.62E-23	postive
DLAT	FAM83A	0.420279437	8.09E-23	postive
DLD	FAM83D	0.501256847	3.62E-33	postive
DLAT	FAM83D	0.470381957	6.83E-29	postive
PDHA1	FAM83D	0.413704537	4.29E-22	postive
LIAS	FANCD2	0.411325639	7.78E-22	postive
LIPT1	FANCD2	0.409219771	1.31E-21	postive
DLD	FANCD2	0.565990411	1.09E-43	postive
DLAT	FANCD2	0.550015308	7.09E-41	postive
PDHA1	FANCD2	0.532128705	6.68E-38	postive
PDHB	FANCD2	0.467459505	1.65E-28	postive
MTF1	FANCD2	0.472073408	4.09E-29	postive
DLD	FANCI	0.621071968	1.15E-54	postive
DLAT	FANCI	0.592230514	1.18E-48	postive
PDHA1	FANCI	0.545781076	3.72E-40	postive
MTF1	FANCI	0.454566354	7.27E-27	postive
MTF1	FBLN5	0.4398047	4.58E-25	postive

PDHB	FBP1	0.411082916	8.26E-22	postive
LIAS	FBXO16	0.412969947	5.16E-22	postive
LIPT1	FBXO16	0.431203923	4.66E-24	postive
DLAT	FBXO16	0.426107166	1.79E-23	postive
PDHB	FBXO16	0.577933308	6.82E-46	postive
FDX1	FBXO44	0.482970599	1.39E-30	postive
LIAS	FBXO44	0.468678249	1.14E-28	postive
LIPT1	FBXO44	0.552376114	2.78E-41	postive
PDHA1	FBXO44	0.551573039	3.83E-41	postive
PDHB	FBXO44	0.471513495	4.85E-29	postive
MTF1	FBXO44	0.441342274	3.00E-25	postive
FDX1	FBXO45	0.415770723	2.55E-22	postive
LIPT1	FBXO45	0.433340989	2.63E-24	postive
DLD	FBXO45	0.657161008	3.92E-63	postive
DLAT	FBXO45	0.641358312	2.76E-59	postive
PDHA1	FBXO45	0.552955189	2.21E-41	postive
PDHB	FBXO45	0.441067825	3.24E-25	postive
MTF1	FBXO45	0.645138916	3.48E-60	postive
FDX1	FBXO9	0.507449438	4.45E-34	postive
LIAS	FBXO9	0.57131832	1.16E-44	postive
LIPT1	FBXO9	0.667009903	1.19E-65	postive
DLD	FBXO9	0.513619929	5.26E-35	postive
DLAT	FBXO9	0.551403497	4.09E-41	postive
PDHA1	FBXO9	0.547782015	1.71E-40	postive
PDHB	FBXO9	0.587434797	1.03E-47	postive
MTF1	FBXO9	0.588364396	6.79E-48	postive
FDX1	FCGRT	0.476242483	1.14E-29	postive
LIPT1	FCGRT	0.501228324	3.66E-33	postive
PDHA1	FCGRT	0.43957684	4.87E-25	postive
PDHB	FCGRT	0.532673673	5.46E-38	postive
FDX1	FCHSD2	0.478664055	5.37E-30	postive
LIPT1	FCHSD2	0.537683694	8.32E-39	postive
DLD	FCHSD2	0.429002124	8.36E-24	postive
DLAT	FCHSD2	0.517735311	1.24E-35	postive
PDHA1	FCHSD2	0.445995542	8.26E-26	postive
PDHB	FCHSD2	0.42759121	1.21E-23	postive
MTF1	FCHSD2	0.668505546	4.84E-66	postive
GLS	FCHSD2	0.403079425	5.89E-21	postive
LIAS	FHIT	0.503652741	1.62E-33	postive
LIPT1	FHIT	0.460146348	1.44E-27	postive
PDHA1	FHIT	0.447701872	5.12E-26	postive
PDHB	FHIT	0.570592698	1.58E-44	postive
FDX1	FKBP4	0.415404169	2.80E-22	postive
LIAS	FKBP4	0.485339616	6.57E-31	postive
DLD	FKBP4	0.63960087	7.16E-59	postive
DLAT	FKBP4	0.560068276	1.26E-42	postive
PDHA1	FKBP4	0.543510735	8.97E-40	postive
PDHB	FKBP4	0.53892999	5.19E-39	postive

MTF1	FKBP4	0.418518695	1.27E-22	postive
FDX1	FMC1	0.440253313	4.05E-25	postive
LIAS	FMC1	0.511312581	1.17E-34	postive
LIPT1	FMC1	0.503998513	1.44E-33	postive
DLD	FMC1	0.46111065	1.09E-27	postive
PDHA1	FMC1	0.578222901	6.01E-46	postive
PDHB	FMC1	0.601467618	1.63E-50	postive
MTF1	FMNL3	0.619569875	2.45E-54	postive
GLS	FMNL3	0.459022775	2.00E-27	postive
LIAS	FMO4	0.413761461	4.23E-22	postive
LIPT1	FMO4	0.490473636	1.26E-31	postive
MTF1	FMO4	0.402077116	7.50E-21	postive
FDX1	FOSL2	0.401033325	9.65E-21	postive
DLD	FOSL2	0.494154246	3.82E-32	postive
DLAT	FOSL2	0.502058162	2.77E-33	postive
PDHA1	FOSL2	0.421777156	5.51E-23	postive
PDHB	FOSL2	0.403697656	5.07E-21	postive
MTF1	FOSL2	0.645072242	3.61E-60	postive
DLD	FOXM1	0.529998508	1.47E-37	postive
DLAT	FOXM1	0.478148935	6.30E-30	postive
PDHA1	FOXM1	0.477961439	6.68E-30	postive
FDX1	FRG1HP	0.491549539	8.92E-32	postive
LIAS	FRG1HP	0.545175529	4.71E-40	postive
LIPT1	FRG1HP	0.629721091	1.36E-56	postive
DLD	FRG1HP	0.505678487	8.14E-34	postive
DLAT	FRG1HP	0.523232347	1.74E-36	postive
PDHA1	FRG1HP	0.472550794	3.53E-29	postive
PDHB	FRG1HP	0.574870947	2.56E-45	postive
MTF1	FRG1HP	0.529123385	2.03E-37	postive
FDX1	FUCA1	0.533866172	3.50E-38	postive
LIAS	FUCA1	0.409171932	1.33E-21	postive
LIPT1	FUCA1	0.524570913	1.07E-36	postive
PDHB	FUCA1	0.418407122	1.31E-22	postive
MTF1	FUCA1	0.477630869	7.40E-30	postive
PDHA1	FUT1	0.466779238	2.02E-28	postive
PDHB	FUT1	0.426210433	1.74E-23	postive
MTF1	FUT1	0.406341841	2.66E-21	postive
FDX1	FUT4	0.458208003	2.54E-27	postive
DLD	FUT4	0.478758058	5.22E-30	postive
DLAT	FUT4	0.434685937	1.84E-24	postive
MTF1	FUT4	0.513684046	5.15E-35	postive
DLAT	FYCO1	0.482545864	1.59E-30	postive
PDHA1	FYCO1	0.435584335	1.44E-24	postive
PDHB	FYCO1	0.485565916	6.11E-31	postive
MTF1	FYCO1	0.732275613	3.96E-85	postive
MTF1	FYN	0.532719339	5.36E-38	postive
GLS	FYN	0.460866029	1.17E-27	postive
DLAT	FZD3	0.409246444	1.30E-21	postive

PDHB	FZD3	0.432163554	3.61E-24	postive
MTF1	GAB3	0.463943047	4.71E-28	postive
GLS	GAB3	0.42858858	9.32E-24	postive
PDHA1	GADD45G	0.401865078	7.90E-21	postive
PDHB	GADD45G	0.416421325	2.16E-22	postive
LIAS	GALNT11	0.446740737	6.70E-26	postive
LIPT1	GALNT11	0.481049227	2.55E-30	postive
DLD	GALNT11	0.459067734	1.97E-27	postive
DLAT	GALNT11	0.435918232	1.32E-24	postive
PDHA1	GALNT11	0.444230987	1.35E-25	postive
PDHB	GALNT11	0.496714224	1.64E-32	postive
MTF1	GALNT11	0.501154632	3.75E-33	postive
FDX1	GALNT2	0.493554963	4.64E-32	postive
LIAS	GALNT2	0.401071412	9.56E-21	postive
DLD	GALNT2	0.571321692	1.16E-44	postive
DLAT	GALNT2	0.59990604	3.40E-50	postive
PDHA1	GALNT2	0.589793066	3.57E-48	postive
PDHB	GALNT2	0.555222257	8.93E-42	postive
MTF1	GALNT2	0.59168937	1.51E-48	postive
LIAS	GAPDH	0.417533758	1.63E-22	postive
DLD	GAPDH	0.558815262	2.09E-42	postive
DLAT	GAPDH	0.509374762	2.30E-34	postive
PDHA1	GAPDH	0.543036647	1.08E-39	postive
PDHB	GAPDH	0.553560674	1.74E-41	postive
DLD	GAPDHP44	0.403657374	5.12E-21	postive
DLAT	GAPDHP44	0.410370487	9.86E-22	postive
DLD	GAPDHP65	0.516522856	1.90E-35	postive
DLAT	GAPDHP65	0.45691307	3.69E-27	postive
PDHA1	GAPDHP65	0.436598686	1.10E-24	postive
PDHB	GAPDHP65	0.455573716	5.44E-27	postive
DLD	GAPDHP68	0.429970698	6.47E-24	postive
DLAT	GAPDHP68	0.40926583	1.30E-21	postive
PDHB	GAPDHP68	0.407023873	2.25E-21	postive
DLD	GAPDHP71	0.440437894	3.85E-25	postive
DLD	GAPDHP72	0.462689071	6.82E-28	postive
DLAT	GAPDHP72	0.44183673	2.62E-25	postive
PDHB	GAPDHP72	0.430145234	6.18E-24	postive
FDX1	GCDH	0.507468591	4.42E-34	postive
LIAS	GCDH	0.533556004	3.93E-38	postive
LIPT1	GCDH	0.600355373	2.75E-50	postive
DLD	GCDH	0.524764708	9.97E-37	postive
DLAT	GCDH	0.507681158	4.11E-34	postive
PDHA1	GCDH	0.651163766	1.20E-61	postive
PDHB	GCDH	0.677601773	1.81E-68	postive
MTF1	GCDH	0.447172874	5.94E-26	postive
DLD	GCLC	0.416475259	2.13E-22	postive
MTF1	GCSAM	0.436965815	9.92E-25	postive
FDX1	GGA2	0.470462189	6.67E-29	postive

LIAS	GGA2	0.442643802	2.10E-25	postive
LIPT1	GGA2	0.56577283	1.20E-43	postive
DLD	GGA2	0.420575532	7.50E-23	postive
DLAT	GGA2	0.439638801	4.79E-25	postive
PDHA1	GGA2	0.559258642	1.75E-42	postive
PDHB	GGA2	0.457886901	2.78E-27	postive
MTF1	GGA2	0.666778873	1.37E-65	postive
GLS	GGA2	0.430774147	5.23E-24	postive
DLD	GGH	0.43020729	6.07E-24	postive
DLAT	GGH	0.408926637	1.41E-21	postive
LIPT1	GIMAP6	0.41646947	2.14E-22	postive
MTF1	GIMAP6	0.473305836	2.81E-29	postive
FDX1	GKAP1	0.400544498	1.09E-20	postive
LIPT1	GKAP1	0.531520733	8.37E-38	postive
DLD	GKAP1	0.42383818	3.23E-23	postive
DLAT	GKAP1	0.428039423	1.08E-23	postive
PDHA1	GKAP1	0.55998301	1.30E-42	postive
PDHB	GKAP1	0.571830579	9.36E-45	postive
LIPT1	GLCCI1	0.462023759	8.30E-28	postive
MTF1	GLCCI1	0.453957241	8.66E-27	postive
LIAS	GLIS2-AS1	0.407785695	1.87E-21	postive
LIPT1	GLS2	0.430456765	5.69E-24	postive
LIPT1	GLUD1P3	0.432679597	3.15E-24	postive
PDHA1	GLUD1P3	0.429637787	7.06E-24	postive
MTF1	GLUD1P3	0.504266355	1.31E-33	postive
FDX1	GMDS-DT	0.401360663	8.92E-21	postive
LIAS	GMDS-DT	0.492038951	7.61E-32	postive
LIPT1	GMDS-DT	0.528518798	2.53E-37	postive
DLAT	GMDS-DT	0.4452914	1.00E-25	postive
PDHA1	GMDS-DT	0.413581366	4.43E-22	postive
PDHB	GMDS-DT	0.441829063	2.62E-25	postive
MTF1	GMDS-DT	0.465398778	3.05E-28	postive
LIPT1	GMFG	0.428338105	9.96E-24	postive
PDHB	GMPR	0.421383332	6.09E-23	postive
MTF1	GNAZ	0.402473896	6.82E-21	postive
LIAS	GNMT	0.400727148	1.04E-20	postive
PDHA1	GNMT	0.415647501	2.63E-22	postive
DLD	GNPNAT1	0.608494547	5.72E-52	postive
DLAT	GNPNAT1	0.647692523	8.44E-61	postive
PDHA1	GNPNAT1	0.451077362	1.97E-26	postive
PDHB	GNPNAT1	0.503263035	1.84E-33	postive
DLD	GOLM1	0.494209583	3.75E-32	postive
DLAT	GOLM1	0.515452061	2.77E-35	postive
PDHA1	GOLM1	0.470746215	6.12E-29	postive
PDHB	GOLM1	0.455303802	5.88E-27	postive
MTF1	GOLM1	0.419184342	1.07E-22	postive
LIPT1	GPD1L	0.430406016	5.76E-24	postive
PDHB	GPD1L	0.488449558	2.43E-31	postive

MTF1	GPD1L	0.42625422	1.72E-23	postive
FDX1	GPR160	0.427867365	1.13E-23	postive
LIPT1	GPR160	0.472058988	4.10E-29	postive
GLS	GPR34	0.439974974	4.37E-25	postive
LIPT1	GPR65	0.406624633	2.48E-21	postive
GLS	GPR65	0.423496627	3.53E-23	postive
LIPT1	GPR75	0.423768264	3.29E-23	postive
PDHA1	GPR75	0.413595575	4.41E-22	postive
MTF1	GPR75	0.592824349	9.00E-49	postive
MTF1	GPRASP1	0.521907052	2.80E-36	postive
DLD	GPRIN1	0.431088653	4.81E-24	postive
DLAT	GPRIN1	0.424687377	2.59E-23	postive
MTF1	GPRIN1	0.427650009	1.19E-23	postive
PDHB	GPSM3	0.437278602	9.11E-25	postive
LIAS	GS1-124K5.3	0.450848192	2.10E-26	postive
LIPT1	GS1-124K5.3	0.426961847	1.43E-23	postive
MTF1	GS1-124K5.3	0.556890831	4.56E-42	postive
LIPT1	GSTM2	0.477954024	6.70E-30	postive
PDHA1	GSTM2	0.425642407	2.02E-23	postive
DLD	GTSE1	0.487639475	3.15E-31	postive
DLAT	GTSE1	0.445275369	1.01E-25	postive
PDHA1	GTSE1	0.473403472	2.72E-29	postive
PDHA1	GUSBP11	0.439030832	5.65E-25	postive
LIPT1	GUSBP4	0.413586394	4.42E-22	postive
MTF1	GVINP1	0.422236597	4.89E-23	postive
FDX1	H2AX	0.428079428	1.07E-23	postive
DLD	H2AX	0.484868028	7.63E-31	postive
DLAT	H2AX	0.560942939	8.78E-43	postive
PDHA1	H2AX	0.596637927	1.56E-49	postive
PDHB	H2AX	0.483407434	1.21E-30	postive
FDX1	H2AZ1	0.45569449	5.25E-27	postive
LIAS	H2AZ1	0.483226425	1.28E-30	postive
LIPT1	H2AZ1	0.479031535	4.79E-30	postive
DLD	H2AZ1	0.610936114	1.75E-52	postive
DLAT	H2AZ1	0.597641416	9.78E-50	postive
PDHA1	H2AZ1	0.575297514	2.13E-45	postive
PDHB	H2AZ1	0.589825775	3.52E-48	postive
PDHB	H3P39	0.495838715	2.19E-32	postive
LIPT1	HACD4	0.445598204	9.22E-26	postive
MTF1	HACD4	0.483835873	1.06E-30	postive
GLS	HACD4	0.410306558	1.00E-21	postive
FDX1	HAGH	0.552681999	2.46E-41	postive
LIAS	HAGH	0.50502161	1.02E-33	postive
LIPT1	HAGH	0.520733986	4.26E-36	postive
DLD	HAGH	0.438925622	5.82E-25	postive
DLAT	HAGH	0.462088309	8.14E-28	postive
PDHA1	HAGH	0.614478259	3.09E-53	postive
PDHB	HAGH	0.6441771	5.91E-60	postive

FDX1	HCFC2	0.406734714	2.42E-21	postive
LIPT1	HCFC2	0.549509893	8.65E-41	postive
DLD	HCFC2	0.47790223	6.81E-30	postive
DLAT	HCFC2	0.497400297	1.31E-32	postive
PDHB	HCFC2	0.471451074	4.94E-29	postive
MTF1	HCFC2	0.547938117	1.60E-40	postive
GLS	HCFC2	0.518856294	8.31E-36	postive
LIPT1	HDAC5	0.491492511	9.09E-32	postive
DLD	HDAC5	0.401857175	7.91E-21	postive
DLAT	HDAC5	0.448612658	3.96E-26	postive
PDHA1	HDAC5	0.592601376	9.96E-49	postive
PDHB	HDAC5	0.546513297	2.80E-40	postive
MTF1	HDAC5	0.583660216	5.54E-47	postive
FDX1	HEIH	0.516116378	2.19E-35	postive
LIAS	HEIH	0.53261221	5.58E-38	postive
LIPT1	HEIH	0.532109873	6.73E-38	postive
DLD	HEIH	0.431891828	3.88E-24	postive
DLAT	HEIH	0.424926262	2.43E-23	postive
PDHA1	HEIH	0.581481121	1.45E-46	postive
PDHB	HEIH	0.609434491	3.63E-52	postive
DLD	HERC1	0.40499424	3.70E-21	postive
DLAT	HERC1	0.454120691	8.26E-27	postive
PDHA1	HERC1	0.426427261	1.64E-23	postive
MTF1	HERC1	0.785922601	4.82E-106	postive
FDX1	HERPUD1	0.439153894	5.47E-25	postive
LIPT1	HERPUD1	0.459236205	1.88E-27	postive
PDHB	HERPUD1	0.454788176	6.82E-27	postive
MTF1	HERPUD1	0.402563196	6.67E-21	postive
FDX1	HINT3	0.510433261	1.59E-34	postive
LIPT1	HINT3	0.506623896	5.90E-34	postive
DLD	HINT3	0.585348907	2.62E-47	postive
DLAT	HINT3	0.561130628	8.13E-43	postive
PDHA1	HINT3	0.568569703	3.71E-44	postive
PDHB	HINT3	0.565951688	1.11E-43	postive
MTF1	HINT3	0.635210917	7.54E-58	postive
DLD	HJURP	0.453019083	1.13E-26	postive
DLAT	HJURP	0.404481124	4.19E-21	postive
PDHA1	HJURP	0.402755688	6.37E-21	postive
FDX1	HLA-DMA	0.408501286	1.57E-21	postive
LIPT1	HLA-DMA	0.430006663	6.41E-24	postive
GLS	HLA-DPA1	0.40017223	1.19E-20	postive
MTF1	HMCN1	0.460004371	1.50E-27	postive
MTF1	HMCN2	0.460212413	1.41E-27	postive
DLD	HMGA1	0.609127118	4.21E-52	postive
DLAT	HMGA1	0.584069071	4.62E-47	postive
PDHA1	HMGA1	0.534688706	2.57E-38	postive
PDHB	HMGA1	0.450530864	2.30E-26	postive
DLD	HMGA1P3	0.510089587	1.79E-34	postive

DLAT	HMGA1P3	0.437872262	7.75E-25	postive
PDHA1	HMGA1P3	0.445553917	9.34E-26	postive
FDX1	HMGN3	0.434824537	1.77E-24	postive
LIAS	HMGN3	0.410428931	9.72E-22	postive
LIPT1	HMGN3	0.505830113	7.73E-34	postive
PDHA1	HMGN3	0.491649814	8.64E-32	postive
PDHB	HMGN3	0.449882512	2.77E-26	postive
DLD	HMMR	0.515639252	2.59E-35	postive
DLAT	HMMR	0.502601007	2.31E-33	postive
GLS	HPGDS	0.438064065	7.36E-25	postive
DLD	HROB	0.495161759	2.74E-32	postive
DLAT	HROB	0.475198171	1.57E-29	postive
PDHA1	HROB	0.514539853	3.81E-35	postive
FDX1	HSD11B1L	0.412647404	5.59E-22	postive
LIAS	HSD11B1L	0.407143172	2.19E-21	postive
LIPT1	HSD11B1L	0.514321303	4.12E-35	postive
PDHA1	HSD11B1L	0.474198797	2.13E-29	postive
PDHB	HSD11B1L	0.51594095	2.33E-35	postive
PDHA1	HSD17B14	0.438443311	6.64E-25	postive
LIPT1	HSD17B7P2	0.430459908	5.68E-24	postive
FDX1	HSDL1	0.435369027	1.53E-24	postive
LIAS	HSDL1	0.454032945	8.48E-27	postive
LIPT1	HSDL1	0.550656878	5.50E-41	postive
DLD	HSDL1	0.532334693	6.19E-38	postive
DLAT	HSDL1	0.55769414	3.29E-42	postive
PDHA1	HSDL1	0.655098498	1.28E-62	postive
PDHB	HSDL1	0.565132193	1.56E-43	postive
MTF1	HSDL1	0.523189912	1.76E-36	postive
LIAS	HSF2BP	0.424603468	2.65E-23	postive
LIPT1	HSF2BP	0.400620069	1.07E-20	postive
DLD	HSF2BP	0.47882147	5.11E-30	postive
DLAT	HSF2BP	0.552323629	2.84E-41	postive
DLD	HSPD1P6	0.419080198	1.10E-22	postive
MTF1	ICA1L	0.411100057	8.23E-22	postive
PDHB	IER3-AS1	0.409931488	1.10E-21	postive
PDHA1	IER5L	0.412465963	5.85E-22	postive
FDX1	IFT172	0.420556201	7.54E-23	postive
LIAS	IFT172	0.401621534	8.37E-21	postive
LIPT1	IFT172	0.508936343	2.67E-34	postive
PDHA1	IFT172	0.511270644	1.19E-34	postive
PDHB	IFT172	0.417290204	1.74E-22	postive
MTF1	IFT172	0.619486283	2.55E-54	postive
MTF1	IGSF10	0.45069262	2.20E-26	postive
DLAT	IKZF4	0.47276073	3.31E-29	postive
PDHA1	IKZF4	0.563481543	3.09E-43	postive
PDHB	IKZF4	0.484781824	7.84E-31	postive
MTF1	IKZF4	0.640257745	5.02E-59	postive
FDX1	IKZF5	0.488495601	2.39E-31	postive

LIAS	IKZF5	0.555510813	7.95E-42	postive
LIPT1	IKZF5	0.649507445	3.06E-61	postive
DLD	IKZF5	0.513290197	5.91E-35	postive
DLAT	IKZF5	0.576306884	1.38E-45	postive
PDHA1	IKZF5	0.451548273	1.72E-26	postive
PDHB	IKZF5	0.62666582	6.61E-56	postive
MTF1	IKZF5	0.53348432	4.03E-38	postive
MTF1	IL16	0.412936251	5.20E-22	postive
MTF1	IL7R	0.408027166	1.76E-21	postive
FDX1	ILDR1	0.408246188	1.67E-21	postive
LIPT1	ILDR1	0.472329513	3.78E-29	postive
PDHA1	ILDR1	0.46727484	1.74E-28	postive
PDHB	ILDR1	0.432808283	3.04E-24	postive
MTF1	ILDR1	0.497826518	1.14E-32	postive
LIAS	ILF3-DT	0.442888314	1.96E-25	postive
LIPT1	ILF3-DT	0.526503199	5.30E-37	postive
PDHA1	ILF3-DT	0.519354052	6.96E-36	postive
PDHB	ILF3-DT	0.501323527	3.54E-33	postive
MTF1	ILF3-DT	0.415669009	2.62E-22	postive
PDHA1	INAFM2	0.466953627	1.92E-28	postive
PDHB	INAFM2	0.511350811	1.16E-34	postive
MTF1	INAFM2	0.494476917	3.43E-32	postive
LIAS	INCA1	0.480458926	3.07E-30	postive
LIPT1	INCA1	0.563594606	2.95E-43	postive
DLD	INCA1	0.403596348	5.20E-21	postive
DLAT	INCA1	0.418407869	1.31E-22	postive
PDHA1	INCA1	0.564103207	2.39E-43	postive
PDHB	INCA1	0.523190202	1.76E-36	postive
MTF1	INCA1	0.48251372	1.61E-30	postive
FDX1	ING4	0.532094556	6.77E-38	postive
LIAS	ING4	0.56609703	1.04E-43	postive
LIPT1	ING4	0.648641245	4.97E-61	postive
DLD	ING4	0.430539343	5.56E-24	postive
DLAT	ING4	0.462663326	6.87E-28	postive
PDHA1	ING4	0.554376611	1.25E-41	postive
PDHB	ING4	0.648903788	4.29E-61	postive
FDX1	INPP5A	0.442577949	2.13E-25	postive
LIAS	INPP5A	0.434671007	1.84E-24	postive
LIPT1	INPP5A	0.443940382	1.46E-25	postive
DLD	INPP5A	0.459355734	1.82E-27	postive
DLAT	INPP5A	0.510142698	1.76E-34	postive
PDHA1	INPP5A	0.553417095	1.84E-41	postive
PDHB	INPP5A	0.555018541	9.69E-42	postive
MTF1	INPP5A	0.591491103	1.65E-48	postive
LIPT1	INPP5B	0.51178013	9.99E-35	postive
MTF1	INPP5B	0.603633606	5.86E-51	postive
PDHA1	INPP5J	0.431184719	4.69E-24	postive
MTF1	INPP5J	0.405621117	3.17E-21	postive

LIPT1	IPO5P1	0.510677088	1.46E-34	postive
LIPT1	IQCB1	0.595265029	2.94E-49	postive
DLD	IQCB1	0.52895769	2.16E-37	postive
DLAT	IQCB1	0.467834119	1.47E-28	postive
PDHA1	IQCB1	0.533008357	4.82E-38	postive
PDHB	IQCB1	0.485729024	5.80E-31	postive
MTF1	IQCB1	0.564581051	1.96E-43	postive
LIAS	IQCC	0.469217576	9.71E-29	postive
LIPT1	IQCC	0.572318751	7.61E-45	postive
DLAT	IQCC	0.452312422	1.39E-26	postive
PDHA1	IQCC	0.506731371	5.68E-34	postive
PDHB	IQCC	0.421791786	5.49E-23	postive
MTF1	IQCC	0.564189303	2.31E-43	postive
LIPT1	IRAK1BP1	0.502709332	2.22E-33	postive
DLAT	IRAK1BP1	0.435884943	1.33E-24	postive
PDHB	IRAK1BP1	0.484766889	7.88E-31	postive
PDHB	IRX3	0.402955466	6.07E-21	postive
LIAS	ITFG2-AS1	0.47921895	4.52E-30	postive
LIPT1	ITFG2-AS1	0.447797905	4.98E-26	postive
PDHB	ITFG2-AS1	0.417274064	1.74E-22	postive
MTF1	ITGA4	0.491091108	1.04E-31	postive
GLS	ITGA4	0.41076443	8.94E-22	postive
MTF1	ITGA8	0.403490808	5.33E-21	postive
MTF1	ITGA9	0.431358402	4.47E-24	postive
LIPT1	ITGA9-AS1	0.485842374	5.60E-31	postive
DLD	ITGB1-DT	0.40807908	1.74E-21	postive
FDX1	IVD	0.458814746	2.13E-27	postive
LIAS	IVD	0.481901305	1.95E-30	postive
LIPT1	IVD	0.423792472	3.27E-23	postive
PDHA1	IVD	0.508601971	2.99E-34	postive
PDHB	IVD	0.514929113	3.33E-35	postive
MTF1	IVD	0.465058062	3.38E-28	postive
LIPT1	KANK1	0.430078724	6.29E-24	postive
PDHA1	KANK1	0.461700248	9.13E-28	postive
PDHB	KANK1	0.441887299	2.58E-25	postive
MTF1	KANK1	0.548631341	1.22E-40	postive
LIPT1	KAT2B	0.449398793	3.17E-26	postive
MTF1	KAT2B	0.526168768	5.99E-37	postive
GLS	KAT2B	0.4156871	2.61E-22	postive
FDX1	KAT8	0.470654271	6.29E-29	postive
LIAS	KAT8	0.51622753	2.11E-35	postive
LIPT1	KAT8	0.597512373	1.04E-49	postive
DLD	KAT8	0.418520263	1.27E-22	postive
DLAT	KAT8	0.450731201	2.17E-26	postive
PDHA1	KAT8	0.636021309	4.89E-58	postive
PDHB	KAT8	0.578691859	4.90E-46	postive
MTF1	KAT8	0.502101963	2.73E-33	postive
MTF1	KCNAB1	0.439896104	4.46E-25	postive

MTF1	KCNE1	0.44486503	1.13E-25	postive
PDHA1	KCNJ14	0.406513364	2.55E-21	postive
MTF1	KCNJ14	0.498271951	9.81E-33	postive
MTF1	KCNMA1	0.401687983	8.24E-21	postive
MTF1	KCTD12	0.497224821	1.39E-32	postive
GLS	KCTD12	0.466941383	1.93E-28	postive
PDHA1	KIAA0895L	0.541500538	1.94E-39	postive
MTF1	KIAA0895L	0.412354884	6.02E-22	postive
DLAT	KIAA1109	0.437311657	9.03E-25	postive
PDHA1	KIAA1109	0.411114574	8.20E-22	postive
MTF1	KIAA1109	0.719883245	5.27E-81	postive
LIAS	KIAA1328	0.404076537	4.62E-21	postive
LIPT1	KIAA1328	0.526424441	5.45E-37	postive
DLAT	KIAA1328	0.439531902	4.93E-25	postive
PDHA1	KIAA1328	0.407208727	2.15E-21	postive
PDHB	KIAA1328	0.472039282	4.13E-29	postive
MTF1	KIAA1328	0.56658832	8.51E-44	postive
DLD	KIF11	0.531489675	8.47E-38	postive
DLAT	KIF11	0.519870831	5.79E-36	postive
PDHA1	KIF11	0.448731026	3.83E-26	postive
DLD	KIF14	0.4842712	9.22E-31	postive
DLAT	KIF14	0.483620879	1.13E-30	postive
PDHA1	KIF14	0.402482997	6.80E-21	postive
DLD	KIF18A	0.545897706	3.56E-40	postive
DLAT	KIF18A	0.571201028	1.22E-44	postive
PDHA1	KIF18A	0.412694713	5.53E-22	postive
DLD	KIF18B	0.433015195	2.88E-24	postive
DLAT	KIF18B	0.402031651	7.59E-21	postive
PDHA1	KIF18B	0.486917778	3.97E-31	postive
DLD	KIF20A	0.461566659	9.49E-28	postive
DLAT	KIF20A	0.457571763	3.05E-27	postive
PDHA1	KIF20A	0.461957083	8.46E-28	postive
DLD	KIF20B	0.58895511	5.21E-48	postive
DLAT	KIF20B	0.598550589	6.40E-50	postive
PDHA1	KIF20B	0.440537701	3.74E-25	postive
MTF1	KIF20B	0.50769951	4.08E-34	postive
DLD	KIF23	0.52862065	2.44E-37	postive
DLAT	KIF23	0.507767471	3.99E-34	postive
PDHA1	KIF23	0.445131973	1.05E-25	postive
DLD	KIF2C	0.485001438	7.32E-31	postive
DLAT	KIF2C	0.474177666	2.15E-29	postive
PDHA1	KIF2C	0.454475233	7.46E-27	postive
DLD	KIF4A	0.503306452	1.82E-33	postive
DLAT	KIF4A	0.478954189	4.91E-30	postive
PDHA1	KIF4A	0.460435657	1.32E-27	postive
DLD	KIFC1	0.497902451	1.11E-32	postive
DLAT	KIFC1	0.471580797	4.75E-29	postive
PDHA1	KIFC1	0.503951272	1.46E-33	postive

LIAS	KLHDC1	0.426470337	1.63E-23	postive
LIPT1	KLHDC1	0.491874058	8.03E-32	postive
MTF1	KLHDC1	0.440038786	4.29E-25	postive
LIAS	KLHDC8B	0.43168358	4.10E-24	postive
LIPT1	KLHDC8B	0.450721339	2.18E-26	postive
PDHA1	KLHDC8B	0.489322017	1.83E-31	postive
PDHB	KLHDC8B	0.62670877	6.47E-56	postive
PDHA1	KLHDC9	0.44356769	1.62E-25	postive
PDHB	KLHDC9	0.470340318	6.92E-29	postive
LIPT1	KLRG1	0.450190208	2.54E-26	postive
LIPT1	KMT2E	0.445975648	8.30E-26	postive
DLD	KMT2E	0.59859048	6.29E-50	postive
DLAT	KMT2E	0.536634154	1.24E-38	postive
PDHA1	KMT2E	0.490756003	1.15E-31	postive
PDHB	KMT2E	0.433479526	2.54E-24	postive
MTF1	KMT2E	0.769709496	3.99E-99	postive
DLD	KNL1	0.513186215	6.12E-35	postive
DLAT	KNL1	0.494694154	3.20E-32	postive
MTF1	KNL1	0.405273642	3.46E-21	postive
FDX1	KNSTRN	0.411797817	6.91E-22	postive
LIAS	KNSTRN	0.433494835	2.53E-24	postive
DLD	KNSTRN	0.59051063	2.58E-48	postive
DLAT	KNSTRN	0.580677739	2.06E-46	postive
PDHA1	KNSTRN	0.53539303	1.97E-38	postive
PDHB	KNSTRN	0.518518885	9.37E-36	postive
DLD	KPNA2	0.569692137	2.31E-44	postive
DLAT	KPNA2	0.562214091	5.21E-43	postive
PDHA1	KPNA2	0.488207257	2.63E-31	postive
PDHB	KPNA2	0.405612617	3.18E-21	postive
FDX1	KRT18	0.48326958	1.27E-30	postive
DLD	KRT18	0.448454746	4.14E-26	postive
DLAT	KRT18	0.519636476	6.30E-36	postive
PDHA1	KRT18	0.568844548	3.31E-44	postive
PDHB	KRT18	0.573686674	4.24E-45	postive
DLD	KRT18P17	0.421863927	5.38E-23	postive
DLAT	KRT18P17	0.457349473	3.25E-27	postive
PDHA1	KRT18P17	0.424140318	2.99E-23	postive
PDHB	KRT18P17	0.442343731	2.28E-25	postive
FDX1	KRT18P28	0.45761977	3.01E-27	postive
DLD	KRT18P28	0.44784795	4.91E-26	postive
DLAT	KRT18P28	0.526378614	5.54E-37	postive
PDHA1	KRT18P28	0.461632724	9.31E-28	postive
PDHB	KRT18P28	0.514929005	3.33E-35	postive
DLAT	KRT18P38	0.440012521	4.32E-25	postive
DLD	KRT18P68	0.40680209	2.38E-21	postive
DLAT	KRT18P68	0.443551321	1.63E-25	postive
FDX1	KRT8	0.478023532	6.55E-30	postive
DLD	KRT8	0.464935671	3.50E-28	postive

DLAT	KRT8	0.506138557	6.96E-34	postive
PDHA1	KRT8	0.550812821	5.17E-41	postive
PDHB	KRT8	0.531936354	7.18E-38	postive
DLAT	KRT8P14	0.420317463	8.02E-23	postive
DLD	KRT8P18	0.401117585	9.45E-21	postive
DLAT	KRT8P18	0.406531318	2.54E-21	postive
LIPT1	L3MBTL2-AS1	0.468627695	1.16E-28	postive
MTF1	L3MBTL2-AS1	0.556858717	4.62E-42	postive
GLS	L3MBTL2-AS1	0.414718974	3.33E-22	postive
MTF1	LAMA2	0.501094098	3.83E-33	postive
MTF1	LCA5	0.525604478	7.35E-37	postive
FDX1	LDB1	0.462382122	7.47E-28	postive
LIAS	LDB1	0.477720358	7.20E-30	postive
LIPT1	LDB1	0.551924507	3.33E-41	postive
DLD	LDB1	0.461250627	1.04E-27	postive
DLAT	LDB1	0.503112439	1.94E-33	postive
PDHA1	LDB1	0.653323328	3.54E-62	postive
PDHB	LDB1	0.600695465	2.35E-50	postive
MTF1	LDB1	0.608996981	4.49E-52	postive
MTF1	LDB2	0.471537376	4.81E-29	postive
FDX1	LDHA	0.478021355	6.56E-30	postive
LIAS	LDHA	0.442705329	2.06E-25	postive
LIPT1	LDHA	0.400508272	1.09E-20	postive
DLD	LDHA	0.553498915	1.78E-41	postive
DLAT	LDHA	0.573562284	4.48E-45	postive
PDHA1	LDHA	0.514247246	4.23E-35	postive
PDHB	LDHA	0.588462463	6.50E-48	postive
PDHA1	LDHD	0.428569752	9.37E-24	postive
PDHB	LDHD	0.417730644	1.55E-22	postive
MTF1	LDLRAD3	0.459876789	1.56E-27	postive
DLAT	LGR4	0.458423638	2.38E-27	postive
MTF1	LIFR	0.433819967	2.32E-24	postive
MTF1	LILRB1	0.47466873	1.85E-29	postive
MTF1	LIMCH1	0.426238565	1.73E-23	postive
FDX1	LIMD1	0.405368098	3.38E-21	postive
LIAS	LIMD1	0.405416066	3.34E-21	postive
LIPT1	LIMD1	0.446336361	7.50E-26	postive
DLAT	LIMD1	0.452275959	1.40E-26	postive
PDHA1	LIMD1	0.483398649	1.22E-30	postive
PDHB	LIMD1	0.564645184	1.91E-43	postive
MTF1	LIMD1	0.568897442	3.24E-44	postive
LIPT1	LIMD1-AS1	0.409738387	1.15E-21	postive
LIAS	LINC00240	0.41523627	2.92E-22	postive
LIPT1	LINC00240	0.443728637	1.55E-25	postive
FDX1	LINC00324	0.435008007	1.68E-24	postive
LIAS	LINC00324	0.462885114	6.44E-28	postive
LIPT1	LINC00324	0.542299969	1.43E-39	postive
PDHB	LINC00324	0.500707307	4.36E-33	postive

FDX1	LINC00339	0.52004444	5.45E-36	postive
LIAS	LINC00339	0.577671014	7.64E-46	postive
LIPT1	LINC00339	0.677036473	2.58E-68	postive
DLD	LINC00339	0.435406391	1.51E-24	postive
DLAT	LINC00339	0.452451942	1.33E-26	postive
PDHA1	LINC00339	0.543115245	1.05E-39	postive
PDHB	LINC00339	0.56058747	1.02E-42	postive
MTF1	LINC00339	0.424556667	2.68E-23	postive
LIPT1	LINC00539	0.458991589	2.02E-27	postive
MTF1	LINC00639	0.437884252	7.73E-25	postive
LIPT1	LINC00652	0.426100048	1.79E-23	postive
MTF1	LINC00654	0.480737694	2.81E-30	postive
LIAS	LINC00663	0.433173343	2.76E-24	postive
LIPT1	LINC00663	0.472046491	4.12E-29	postive
MTF1	LINC00663	0.451675372	1.66E-26	postive
FDX1	LINC00847	0.479705612	3.88E-30	postive
LIAS	LINC00847	0.471598745	4.72E-29	postive
LIPT1	LINC00847	0.570266994	1.82E-44	postive
DLD	LINC00847	0.464176711	4.39E-28	postive
DLAT	LINC00847	0.426408832	1.65E-23	postive
PDHA1	LINC00847	0.55968569	1.47E-42	postive
PDHB	LINC00847	0.537067777	1.05E-38	postive
MTF1	LINC00847	0.404720251	3.95E-21	postive
FDX1	LINC00857	0.418333907	1.33E-22	postive
DLD	LINC00857	0.41235219	6.02E-22	postive
DLAT	LINC00857	0.485146201	6.99E-31	postive
PDHB	LINC00857	0.427397219	1.28E-23	postive
LIAS	LINC00957	0.425584029	2.05E-23	postive
LIPT1	LINC00957	0.415906311	2.46E-22	postive
PDHA1	LINC00957	0.418761625	1.19E-22	postive
PDHB	LINC00957	0.460586056	1.27E-27	postive
MTF1	LINC00987	0.468715012	1.13E-28	postive
MTF1	LINC01132	0.478216986	6.17E-30	postive
LIPT1	LINC01144	0.481163271	2.46E-30	postive
PDHA1	LINC01144	0.528605623	2.45E-37	postive
PDHB	LINC01144	0.405564389	3.22E-21	postive
LIAS	LINC01635	0.40178673	8.05E-21	postive
MTF1	LINC02649	0.401016038	9.69E-21	postive
FDX1	LINC02732	0.440527208	3.75E-25	postive
GLS	LINC02861	0.42259875	4.45E-23	postive
DLD	LMNB2	0.55226481	2.91E-41	postive
DLAT	LMNB2	0.579376856	3.64E-46	postive
PDHA1	LMNB2	0.596298925	1.82E-49	postive
PDHB	LMNB2	0.441730089	2.70E-25	postive
MTF1	LMNB2	0.584397088	4.00E-47	postive
GLS	LPCAT1	0.418904455	1.15E-22	postive
FDX1	LPGAT1	0.406539936	2.54E-21	postive
LIPT1	LPGAT1	0.403945734	4.77E-21	postive

DLD	LPGAT1	0.535968912	1.59E-38	postive
DLAT	LPGAT1	0.644018201	6.45E-60	postive
PDHA1	LPGAT1	0.449536078	3.05E-26	postive
PDHB	LPGAT1	0.447870762	4.88E-26	postive
MTF1	LPGAT1	0.554551615	1.17E-41	postive
PDHA1	LPIN1	0.477651623	7.36E-30	postive
MTF1	LPIN1	0.594718135	3.78E-49	postive
DLD	LRFN4	0.44727717	5.77E-26	postive
DLAT	LRFN4	0.427810997	1.14E-23	postive
PDHA1	LRFN4	0.471093326	5.51E-29	postive
PDHB	LRIG1	0.447820841	4.95E-26	postive
MTF1	LRIG1	0.467950912	1.42E-28	postive
FDX1	LRMDA	0.519210869	7.33E-36	postive
LIAS	LRMDA	0.410774749	8.92E-22	postive
LIPT1	LRMDA	0.453791877	9.08E-27	postive
PDHB	LRMDA	0.416634566	2.05E-22	postive
MTF1	LRP2BP	0.408361804	1.62E-21	postive
FDX1	LRRC27	0.454098794	8.32E-27	postive
LIAS	LRRC27	0.530629325	1.16E-37	postive
LIPT1	LRRC27	0.466712618	2.06E-28	postive
PDHA1	LRRC27	0.575180388	2.24E-45	postive
PDHB	LRRC27	0.538721498	5.61E-39	postive
MTF1	LRRC27	0.494724493	3.16E-32	postive
LIPT1	LRRC56	0.415515056	2.72E-22	postive
PDHA1	LRRC56	0.445120023	1.05E-25	postive
MTF1	LRRC56	0.428269522	1.01E-23	postive
LIPT1	LRRC8C-DT	0.415017389	3.08E-22	postive
MTF1	LRRC8C-DT	0.439289295	5.27E-25	postive
MTF1	LSAMP	0.438308293	6.88E-25	postive
FDX1	LTA4H	0.438045509	7.40E-25	postive
LIAS	LTA4H	0.512362476	8.16E-35	postive
LIPT1	LTA4H	0.460606331	1.26E-27	postive
DLD	LTA4H	0.418376369	1.32E-22	postive
DLAT	LTA4H	0.437245995	9.19E-25	postive
PDHA1	LTA4H	0.491179869	1.01E-31	postive
PDHB	LTA4H	0.618089521	5.14E-54	postive
MTF1	LTA4H	0.454986821	6.44E-27	postive
LIPT1	LUC7L	0.464823893	3.62E-28	postive
PDHA1	LUC7L	0.528107229	2.95E-37	postive
PDHB	LUC7L	0.402415551	6.91E-21	postive
MTF1	LUC7L	0.445181448	1.04E-25	postive
FDX1	LYRM9	0.452463791	1.33E-26	postive
LIAS	LYRM9	0.50424452	1.32E-33	postive
LIPT1	LYRM9	0.511973169	9.34E-35	postive
PDHA1	LYRM9	0.426597168	1.57E-23	postive
PDHB	LYRM9	0.463054452	6.12E-28	postive
FDX1	LZTFL1	0.448113624	4.56E-26	postive
LIAS	LZTFL1	0.470139029	7.35E-29	postive

LIPT1	LZTFL1	0.558368326	2.51E-42	postive
DLD	LZTFL1	0.464218544	4.34E-28	postive
DLAT	LZTFL1	0.522603423	2.18E-36	postive
PDHB	LZTFL1	0.622624566	5.23E-55	postive
MTF1	LZTFL1	0.551579076	3.82E-41	postive
LIPT1	LZTS3	0.438837007	5.96E-25	postive
PDHA1	LZTS3	0.483538048	1.16E-30	postive
PDHB	LZTS3	0.466220031	2.39E-28	postive
FDX1	MACO1	0.475880029	1.27E-29	postive
LIAS	MACO1	0.412255935	6.17E-22	postive
LIPT1	MACO1	0.519296499	7.11E-36	postive
DLD	MACO1	0.443187911	1.80E-25	postive
DLAT	MACO1	0.566057409	1.06E-43	postive
PDHA1	MACO1	0.617696078	6.25E-54	postive
PDHB	MACO1	0.552055378	3.16E-41	postive
MTF1	MACO1	0.704748623	2.92E-76	postive
DLD	MAD2L1	0.557783438	3.18E-42	postive
DLAT	MAD2L1	0.52925373	1.93E-37	postive
LIPT1	MAGEH1	0.469535423	8.82E-29	postive
PDHA1	MAGEH1	0.44522294	1.02E-25	postive
PDHB	MAGEH1	0.485579045	6.09E-31	postive
FDX1	MAGIX	0.423271215	3.74E-23	postive
LIAS	MAGIX	0.400983117	9.77E-21	postive
LIPT1	MAGIX	0.414746176	3.30E-22	postive
PDHA1	MAGIX	0.584053707	4.66E-47	postive
PDHB	MAGIX	0.456661109	3.97E-27	postive
MTF1	MAN1C1	0.549573791	8.44E-41	postive
MTF1	MAOB	0.421015559	6.70E-23	postive
LIPT1	MAP3K3	0.45769667	2.94E-27	postive
DLAT	MAP3K3	0.422850367	4.17E-23	postive
PDHA1	MAP3K3	0.479768898	3.81E-30	postive
PDHB	MAP3K3	0.422608813	4.44E-23	postive
MTF1	MAP3K3	0.708326043	2.35E-77	postive
GLS	MAP3K3	0.412482016	5.83E-22	postive
PDHA1	MAPRE3	0.44666072	6.85E-26	postive
MTF1	MAPRE3	0.445904514	8.47E-26	postive
PDHB	MBIP	0.405083932	3.62E-21	postive
FDX1	MCCC1	0.467724787	1.52E-28	postive
LIAS	MCCC1	0.479511237	4.12E-30	postive
LIPT1	MCCC1	0.529622024	1.69E-37	postive
DLD	MCCC1	0.4908486	1.12E-31	postive
DLAT	MCCC1	0.463232203	5.81E-28	postive
PDHA1	MCCC1	0.55199465	3.24E-41	postive
PDHB	MCCC1	0.519811637	5.92E-36	postive
MTF1	MCCC1	0.543924681	7.65E-40	postive
DLD	MCM4	0.572390453	7.38E-45	postive
DLAT	MCM4	0.585723009	2.22E-47	postive
PDHA1	MCM4	0.550561711	5.71E-41	postive

PDHB	MCM4	0.435778899	1.37E-24	postive
MTF1	MCM4	0.420487713	7.67E-23	postive
FDX1	MCM5	0.402785488	6.32E-21	postive
LIAS	MCM5	0.412995664	5.13E-22	postive
LIPT1	MCM5	0.430183086	6.11E-24	postive
DLD	MCM5	0.546908238	2.40E-40	postive
DLAT	MCM5	0.523324999	1.68E-36	postive
PDHA1	MCM5	0.59927962	4.56E-50	postive
PDHB	MCM5	0.480281505	3.24E-30	postive
MTF1	MCM5	0.508665445	2.93E-34	postive
FDX1	MEAF6	0.568996559	3.10E-44	postive
LIAS	MEAF6	0.544535909	6.04E-40	postive
LIPT1	MEAF6	0.651495938	9.98E-62	postive
DLD	MEAF6	0.490680589	1.18E-31	postive
DLAT	MEAF6	0.564369009	2.14E-43	postive
PDHA1	MEAF6	0.576051392	1.54E-45	postive
PDHB	MEAF6	0.596490018	1.67E-49	postive
MTF1	MEAF6	0.610146147	2.57E-52	postive
FDX1	MEAK7	0.484717344	8.01E-31	postive
LIAS	MEAK7	0.463453109	5.44E-28	postive
LIPT1	MEAK7	0.42578842	1.94E-23	postive
DLD	MEAK7	0.583496571	5.96E-47	postive
DLAT	MEAK7	0.58342	6.16E-47	postive
PDHA1	MEAK7	0.573929369	3.83E-45	postive
PDHB	MEAK7	0.492570408	6.40E-32	postive
MTF1	MEAK7	0.542396193	1.38E-39	postive
MTF1	MEF2C	0.401197623	9.27E-21	postive
GLS	MEF2C	0.47297882	3.10E-29	postive
DLD	MELK	0.466598011	2.13E-28	postive
DLAT	MELK	0.435206922	1.60E-24	postive
FDX1	MFSD12	0.466753115	2.04E-28	postive
DLD	MFSD12	0.49267144	6.19E-32	postive
DLAT	MFSD12	0.480938071	2.64E-30	postive
PDHA1	MFSD12	0.550973179	4.86E-41	postive
PDHB	MFSD12	0.463854288	4.83E-28	postive
MTF1	MFSD12	0.493289675	5.06E-32	postive
LIAS	MFSD2B	0.426570441	1.58E-23	postive
DLD	MFSD2B	0.538638615	5.79E-39	postive
DLAT	MFSD2B	0.53965495	3.94E-39	postive
PDHA1	MFSD2B	0.463367314	5.58E-28	postive
LIPT1	MHENCR	0.400920183	9.91E-21	postive
PDHA1	MHENCR	0.532339133	6.18E-38	postive
DLAT	MID1IP1-AS1	0.404996122	3.70E-21	postive
MTF1	MID1IP1-AS1	0.542080504	1.56E-39	postive
LIPT1	MIPEPP3	0.510159478	1.75E-34	postive
DLD	MIPEPP3	0.413678058	4.32E-22	postive
DLAT	MIPEPP3	0.428102765	1.06E-23	postive
PDHA1	MIPEPP3	0.478087824	6.42E-30	postive

PDHB	MIPEPP3	0.480933152	2.64E-30	postive
MTF1	MIPEPP3	0.431391634	4.43E-24	postive
MTF1	MIR186	0.419204181	1.07E-22	postive
MTF1	MIR34A	0.479146896	4.62E-30	postive
MTF1	MIR34AHG	0.449432562	3.14E-26	postive
PDHA1	MIR600HG	0.407080972	2.22E-21	postive
MTF1	MIR600HG	0.528529582	2.52E-37	postive
LIPT1	MIR635	0.403260441	5.64E-21	postive
MTF1	MIR635	0.621086129	1.14E-54	postive
LIAS	MIR762HG	0.456128487	4.64E-27	postive
LIPT1	MIR762HG	0.527577375	3.58E-37	postive
PDHA1	MIR762HG	0.556192416	6.04E-42	postive
PDHB	MIR762HG	0.508500694	3.10E-34	postive
DLD	MKI67	0.466249947	2.37E-28	postive
DLAT	MKI67	0.455534377	5.50E-27	postive
PDHA1	MKI67	0.444450509	1.27E-25	postive
MTF1	MKI67	0.454670507	7.06E-27	postive
DLAT	MLLT3	0.412927157	5.21E-22	postive
PDHB	MLLT3	0.409336661	1.27E-21	postive
MTF1	MLLT3	0.45052962	2.30E-26	postive
LIPT1	MLLT6	0.452428858	1.34E-26	postive
DLD	MLLT6	0.419108311	1.09E-22	postive
DLAT	MLLT6	0.457504686	3.11E-27	postive
PDHA1	MLLT6	0.579887637	2.91E-46	postive
PDHB	MLLT6	0.460666954	1.24E-27	postive
MTF1	MLLT6	0.711118482	3.21E-78	postive
DLD	MND1	0.482534252	1.60E-30	postive
DLAT	MND1	0.457914389	2.76E-27	postive
PDHA1	MND1	0.449498627	3.08E-26	postive
GLS	MNDA	0.479714319	3.87E-30	postive
FDX1	MOAP1	0.472453254	3.64E-29	postive
LIAS	MOAP1	0.509093356	2.53E-34	postive
LIPT1	MOAP1	0.578248177	5.94E-46	postive
DLD	MOAP1	0.457006957	3.59E-27	postive
DLAT	MOAP1	0.444888828	1.12E-25	postive
PDHA1	MOAP1	0.56040132	1.10E-42	postive
PDHB	MOAP1	0.64174507	2.24E-59	postive
MTF1	MOAP1	0.473810937	2.40E-29	postive
LIPT1	MOCS1	0.410854753	8.74E-22	postive
PDHA1	MOCS1	0.414452768	3.56E-22	postive
PDHB	MOCS1	0.417442439	1.67E-22	postive
MTF1	MOCS1	0.480652774	2.89E-30	postive
MTF1	MPEG1	0.496771596	1.61E-32	postive
PDHA1	MPL	0.416910089	1.91E-22	postive
MTF1	MPL	0.533466803	4.06E-38	postive
LIPT1	MPP7	0.405549795	3.23E-21	postive
DLD	MPP7	0.407605696	1.95E-21	postive
DLAT	MPP7	0.489472451	1.75E-31	postive

PDHB	MPP7	0.504942846	1.04E-33	postive
GLS	MRC1	0.457383813	3.22E-27	postive
LIAS	MRNIP	0.434221056	2.08E-24	postive
LIPT1	MRNIP	0.484415622	8.81E-31	postive
PDHA1	MRNIP	0.484376765	8.92E-31	postive
MTF1	MRNIP	0.454194992	8.09E-27	postive
LIPT1	MROH8	0.460604247	1.26E-27	postive
PDHA1	MROH8	0.412189116	6.27E-22	postive
PDHB	MROH8	0.460655048	1.24E-27	postive
MTF1	MROH8	0.473682998	2.50E-29	postive
MTF1	MS4A7	0.419192721	1.07E-22	postive
GLS	MS4A7	0.44198294	2.51E-25	postive
FDX1	MSANTD3	0.456905264	3.70E-27	postive
LIAS	MSANTD3	0.424883739	2.46E-23	postive
LIPT1	MSANTD3	0.491019853	1.06E-31	postive
DLD	MSANTD3	0.645126613	3.50E-60	postive
DLAT	MSANTD3	0.62165321	8.56E-55	postive
PDHA1	MSANTD3	0.541860383	1.69E-39	postive
PDHB	MSANTD3	0.58221327	1.05E-46	postive
MTF1	MSANTD3	0.444629286	1.21E-25	postive
DLD	MTBP	0.491589162	8.81E-32	postive
DLAT	MTBP	0.512863184	6.85E-35	postive
PDHA1	MTBP	0.462429291	7.36E-28	postive
MTF1	MTBP	0.445343935	9.90E-26	postive
MTF1	MTCL1	0.407039072	2.24E-21	postive
DLD	MTHFD2	0.588791041	5.61E-48	postive
DLAT	MTHFD2	0.595986769	2.10E-49	postive
PDHA1	MTHFD2	0.453920438	8.75E-27	postive
PDHB	MTHFD2	0.451108023	1.95E-26	postive
FDX1	MTMR10	0.472550492	3.53E-29	postive
LIAS	MTMR10	0.43400174	2.21E-24	postive
LIPT1	MTMR10	0.458042078	2.66E-27	postive
DLD	MTMR10	0.425097851	2.33E-23	postive
DLAT	MTMR10	0.444801456	1.15E-25	postive
PDHA1	MTMR10	0.447129573	6.01E-26	postive
PDHB	MTMR10	0.483848625	1.05E-30	postive
MTF1	MTMR10	0.626313369	7.93E-56	postive
FDX1	MTMR12	0.465158395	3.28E-28	postive
LIAS	MTMR12	0.444600317	1.22E-25	postive
LIPT1	MTMR12	0.547633534	1.81E-40	postive
DLD	MTMR12	0.553091298	2.09E-41	postive
DLAT	MTMR12	0.596990175	1.32E-49	postive
PDHA1	MTMR12	0.492018359	7.66E-32	postive
PDHB	MTMR12	0.491499318	9.07E-32	postive
MTF1	MTMR12	0.627066089	5.38E-56	postive
MTF1	MTMR8	0.420102442	8.47E-23	postive
DLAT	MTUS1	0.407159416	2.18E-21	postive
PDHB	MTUS1	0.482748792	1.49E-30	postive

MTF1	MTUS1	0.418192823	1.38E-22	postive
PDHA1	MVB12B	0.45579742	5.10E-27	postive
MTF1	MVB12B	0.55121807	4.41E-41	postive
DLD	MYBL2	0.437898834	7.70E-25	postive
PDHA1	MYBL2	0.413021503	5.09E-22	postive
PDHA1	MYCL	0.403786316	4.96E-21	postive
LIAS	MYL5	0.533288107	4.34E-38	postive
LIPT1	MYL5	0.453661635	9.43E-27	postive
PDHA1	MYL5	0.467101268	1.84E-28	postive
PDHB	MYL5	0.418584466	1.25E-22	postive
LIAS	MYLIP	0.403395997	5.45E-21	postive
LIPT1	MYLIP	0.541295009	2.10E-39	postive
DLD	MYLIP	0.404954938	3.73E-21	postive
DLAT	MYLIP	0.443301853	1.75E-25	postive
PDHA1	MYLIP	0.45276655	1.22E-26	postive
PDHB	MYLIP	0.51362824	5.25E-35	postive
MTF1	MYLIP	0.522644026	2.14E-36	postive
FDX1	MYO1E	0.458935469	2.05E-27	postive
DLD	MYO1E	0.481056501	2.54E-30	postive
DLAT	MYO1E	0.516105518	2.20E-35	postive
PDHA1	MYO1E	0.42714668	1.36E-23	postive
MTF1	MYO1E	0.565899211	1.13E-43	postive
LIPT1	MYO6	0.47367965	2.50E-29	postive
DLD	MYO6	0.408225853	1.68E-21	postive
DLAT	MYO6	0.51573049	2.51E-35	postive
PDHA1	MYO6	0.439160305	5.46E-25	postive
PDHB	MYO6	0.429591692	7.15E-24	postive
MTF1	MYO6	0.575844666	1.68E-45	postive
GLS	MYO6	0.452005381	1.51E-26	postive
LIPT1	N4BP2L1	0.509338425	2.32E-34	postive
GLS	N4BP2L1	0.434178724	2.10E-24	postive
MTF1	NAIP	0.531875899	7.34E-38	postive
GLS	NAIP	0.42546804	2.11E-23	postive
MTF1	NAIPP2	0.512719394	7.21E-35	postive
LIPT1	NBPF1	0.461374295	1.00E-27	postive
DLD	NBPF1	0.461959302	8.46E-28	postive
DLAT	NBPF1	0.51843834	9.64E-36	postive
PDHA1	NBPF1	0.490967167	1.08E-31	postive
MTF1	NBPF1	0.733336882	1.71E-85	postive
LIAS	NBPF3	0.403244312	5.66E-21	postive
LIPT1	NBPF3	0.52508159	8.89E-37	postive
DLD	NBPF3	0.434397562	1.98E-24	postive
DLAT	NBPF3	0.457895503	2.78E-27	postive
PDHA1	NBPF3	0.410613035	9.29E-22	postive
PDHB	NBPF3	0.404978434	3.71E-21	postive
MTF1	NBPF3	0.590852857	2.21E-48	postive
DLD	NCAPG	0.50736763	4.57E-34	postive
DLAT	NCAPG	0.498782008	8.28E-33	postive

PDHA1	NCAPG	0.43613688	1.24E-24	postive
DLD	NCAPG2	0.646390255	1.74E-60	postive
DLAT	NCAPG2	0.582312159	1.00E-46	postive
PDHA1	NCAPG2	0.499985328	5.55E-33	postive
MTF1	NCAPG2	0.515684196	2.55E-35	postive
DLD	NCAPH	0.510222341	1.71E-34	postive
DLAT	NCAPH	0.473453493	2.68E-29	postive
PDHA1	NCAPH	0.431228826	4.63E-24	postive
DLD	NDC80	0.476737332	9.76E-30	postive
DLAT	NDC80	0.461966401	8.44E-28	postive
PDHA1	NDC80	0.424300458	2.87E-23	postive
LIPT1	NDRG2	0.459243082	1.88E-27	postive
LIPT1	NDUFA6-DT	0.49345614	4.79E-32	postive
PDHA1	NDUFA6-DT	0.404692973	3.98E-21	postive
MTF1	NDUFA6-DT	0.443549926	1.63E-25	postive
FDX1	NEDD1	0.403945739	4.77E-21	postive
LIPT1	NEDD1	0.448531238	4.05E-26	postive
DLD	NEDD1	0.654485345	1.83E-62	postive
DLAT	NEDD1	0.673667552	2.08E-67	postive
PDHA1	NEDD1	0.539169158	4.74E-39	postive
PDHB	NEDD1	0.530420332	1.26E-37	postive
MTF1	NEDD1	0.634972063	8.56E-58	postive
MTF1	NEDD9	0.408754248	1.47E-21	postive
DLD	NEIL3	0.500042023	5.44E-33	postive
DLAT	NEIL3	0.406030809	2.87E-21	postive
DLD	NEK2	0.504531153	1.20E-33	postive
DLAT	NEK2	0.48759897	3.19E-31	postive
FDX1	NEU1	0.490865058	1.11E-31	postive
LIAS	NEU1	0.436387922	1.16E-24	postive
LIPT1	NEU1	0.531848906	7.41E-38	postive
DLD	NEU1	0.455831476	5.05E-27	postive
DLAT	NEU1	0.464011328	4.61E-28	postive
PDHA1	NEU1	0.562755216	4.17E-43	postive
PDHB	NEU1	0.549254827	9.57E-41	postive
MTF1	NFATC1	0.523492259	1.58E-36	postive
MTF1	NFIX	0.477974661	6.65E-30	postive
MTF1	NHSL2	0.54610084	3.29E-40	postive
FDX1	NICN1	0.462730726	6.74E-28	postive
LIAS	NICN1	0.571194545	1.23E-44	postive
LIPT1	NICN1	0.513549705	5.39E-35	postive
PDHA1	NICN1	0.535573698	1.84E-38	postive
PDHB	NICN1	0.59875615	5.82E-50	postive
MTF1	NICN1	0.487433783	3.37E-31	postive
FDX1	NINJ2-AS1	0.417774099	1.53E-22	postive
LIAS	NINJ2-AS1	0.423162691	3.85E-23	postive
LIPT1	NINJ2-AS1	0.445168726	1.04E-25	postive
PDHA1	NINJ2-AS1	0.4327124	3.12E-24	postive
PDHB	NINJ2-AS1	0.435713617	1.39E-24	postive

MTF1	NINJ2-AS1	0.4149052	3.17E-22	postive
LIPT1	NIPSNAP3B	0.433068551	2.83E-24	postive
LIPT1	NISCH	0.435250902	1.58E-24	postive
PDHA1	NISCH	0.560789799	9.35E-43	postive
PDHB	NISCH	0.538069419	7.19E-39	postive
MTF1	NISCH	0.65025729	2.01E-61	postive
MTF1	NLRC4	0.480443043	3.08E-30	postive
GLS	NLRC4	0.514599103	3.74E-35	postive
MTF1	NLRP1	0.421392365	6.08E-23	postive
LIPT1	NOD1	0.401604485	8.41E-21	postive
MTF1	NOD1	0.463726448	5.02E-28	postive
LIAS	NPAS1	0.412595305	5.67E-22	postive
PDHB	NPC2	0.401060465	9.59E-21	postive
LIPT1	NPHP1	0.506137003	6.96E-34	postive
MTF1	NPHP1	0.470519915	6.55E-29	postive
PDHA1	NPIPA1	0.459967764	1.52E-27	postive
MTF1	NPIPA1	0.42953791	7.25E-24	postive
PDHA1	NPIPP1	0.519888405	5.76E-36	postive
FDX1	NPRL2	0.527572263	3.59E-37	postive
LIAS	NPRL2	0.626792285	6.20E-56	postive
LIPT1	NPRL2	0.625127247	1.46E-55	postive
DLD	NPRL2	0.454713276	6.97E-27	postive
DLAT	NPRL2	0.489806681	1.57E-31	postive
PDHA1	NPRL2	0.616568605	1.10E-53	postive
PDHB	NPRL2	0.789889102	7.91E-108	postive
MTF1	NR3C2	0.415475888	2.75E-22	postive
LIPT1	NRIP2	0.416198372	2.29E-22	postive
PDHA1	NRIP2	0.41226167	6.16E-22	postive
MTF1	NRIP2	0.563722949	2.80E-43	postive
FDX1	NRL	0.474766176	1.79E-29	postive
LIAS	NRL	0.465595647	2.88E-28	postive
LIPT1	NRL	0.559471803	1.60E-42	postive
DLD	NRL	0.404232985	4.45E-21	postive
PDHA1	NRL	0.558253407	2.63E-42	postive
PDHB	NRL	0.603613034	5.92E-51	postive
DLD	NUF2	0.481866528	1.97E-30	postive
DLAT	NUF2	0.448863402	3.69E-26	postive
PDHA1	NUF2	0.413589494	4.42E-22	postive
FDX1	NUPR1	0.417518773	1.64E-22	postive
MTF1	NXPE3	0.557273229	3.91E-42	postive
DLAT	OCLN	0.406242102	2.73E-21	postive
MTF1	OCLN	0.443130784	1.83E-25	postive
LIAS	OGFRP1	0.46087394	1.16E-27	postive
LIPT1	OGFRP1	0.448017593	4.68E-26	postive
DLD	OGFRP1	0.502877194	2.10E-33	postive
DLAT	OGFRP1	0.500347451	4.91E-33	postive
PDHA1	OGFRP1	0.43953658	4.92E-25	postive
MTF1	OGFRP1	0.497252073	1.38E-32	postive

LIPT1	OGT	0.432569382	3.24E-24	postive
PDHA1	OGT	0.41940149	1.01E-22	postive
MTF1	OGT	0.461771113	8.94E-28	postive
GLS	OGT	0.406577758	2.51E-21	postive
DLD	OIP5	0.512891374	6.79E-35	postive
DLAT	OIP5	0.488654878	2.27E-31	postive
PDHA1	OIP5	0.416771008	1.98E-22	postive
DLAT	OPHN1	0.4185729	1.25E-22	postive
PDHA1	OPHN1	0.425123947	2.31E-23	postive
MTF1	OPHN1	0.556353519	5.66E-42	postive
PDHA1	OR7E128P	0.434141258	2.13E-24	postive
FDX1	ORMDL3	0.467006426	1.89E-28	postive
LIAS	ORMDL3	0.423816033	3.25E-23	postive
LIPT1	ORMDL3	0.505738072	7.97E-34	postive
DLD	ORMDL3	0.40093987	9.87E-21	postive
DLAT	ORMDL3	0.453469887	9.96E-27	postive
PDHA1	ORMDL3	0.589141382	4.79E-48	postive
PDHB	ORMDL3	0.560194051	1.19E-42	postive
MTF1	ORMDL3	0.583489065	5.98E-47	postive
FDX1	OSGEP	0.502432937	2.44E-33	postive
LIAS	OSGEP	0.561829163	6.10E-43	postive
LIPT1	OSGEP	0.621749498	8.15E-55	postive
DLD	OSGEP	0.440117941	4.20E-25	postive
DLAT	OSGEP	0.430905178	5.05E-24	postive
PDHA1	OSGEP	0.60867048	5.26E-52	postive
PDHB	OSGEP	0.617484287	6.95E-54	postive
FDX1	OSGIN2	0.430102518	6.25E-24	postive
LIAS	OSGIN2	0.41965313	9.50E-23	postive
LIPT1	OSGIN2	0.42700656	1.41E-23	postive
DLD	OSGIN2	0.588397331	6.69E-48	postive
DLAT	OSGIN2	0.636558942	3.67E-58	postive
PDHA1	OSGIN2	0.452866236	1.18E-26	postive
PDHB	OSGIN2	0.451997195	1.52E-26	postive
MTF1	OSGIN2	0.574863248	2.56E-45	postive
MTF1	P2RY8	0.41610192	2.35E-22	postive
MTF1	PAICSP1	0.496998216	1.50E-32	postive
DLD	PAICSP4	0.43973469	4.66E-25	postive
DLAT	PAICSP4	0.484036244	9.94E-31	postive
MTF1	PAIP2B	0.415981874	2.42E-22	postive
LIPT1	PAN3-AS1	0.463928435	4.73E-28	postive
MTF1	PARD3B	0.594426003	4.32E-49	postive
LIPT1	PBXIP1	0.467830487	1.47E-28	postive
PDHA1	PBXIP1	0.501138879	3.77E-33	postive
PDHB	PBXIP1	0.55697154	4.41E-42	postive
MTF1	PBXIP1	0.473780935	2.43E-29	postive
FDX1	PCBP1-AS1	0.416840967	1.95E-22	postive
LIAS	PCBP1-AS1	0.454068655	8.39E-27	postive
LIPT1	PCBP1-AS1	0.615809846	1.60E-53	postive

PDHA1	PCBP1-AS1	0.453729901	9.25E-27	postive
PDHB	PCBP1-AS1	0.465286157	3.16E-28	postive
MTF1	PCBP1-AS1	0.502909503	2.08E-33	postive
DLD	PCLAF	0.498558508	8.92E-33	postive
DLAT	PCLAF	0.462673973	6.85E-28	postive
PDHA1	PCLAF	0.426419687	1.65E-23	postive
LIPT1	PCP2	0.404351199	4.33E-21	postive
MTF1	PDE4B	0.426622755	1.56E-23	postive
LIPT1	PDE6B	0.408365459	1.62E-21	postive
FDX1	PDIK1L	0.442889186	1.96E-25	postive
LIAS	PDIK1L	0.466300812	2.33E-28	postive
LIPT1	PDIK1L	0.597674053	9.63E-50	postive
DLD	PDIK1L	0.436721573	1.06E-24	postive
DLAT	PDIK1L	0.523610675	1.51E-36	postive
PDHA1	PDIK1L	0.490564382	1.23E-31	postive
PDHB	PDIK1L	0.475523921	1.42E-29	postive
MTF1	PDIK1L	0.60329088	6.90E-51	postive
FDX1	PDK2	0.427496858	1.24E-23	postive
LIAS	PDK2	0.416782187	1.97E-22	postive
LIPT1	PDK2	0.480560412	2.97E-30	postive
DLAT	PDK2	0.422185728	4.96E-23	postive
PDHA1	PDK2	0.539313079	4.48E-39	postive
PDHB	PDK2	0.526282457	5.74E-37	postive
MTF1	PDK2	0.502312352	2.54E-33	postive
MTF1	PDLIM1P4	0.420619038	7.42E-23	postive
FDX1	PEBP1	0.575471036	1.97E-45	postive
LIAS	PEBP1	0.537350252	9.44E-39	postive
LIPT1	PEBP1	0.517174206	1.51E-35	postive
DLD	PEBP1	0.477612194	7.45E-30	postive
DLAT	PEBP1	0.49354937	4.65E-32	postive
PDHA1	PEBP1	0.709149352	1.31E-77	postive
PDHB	PEBP1	0.71113073	3.18E-78	postive
FDX1	PERP	0.450446109	2.36E-26	postive
DLD	PERP	0.502842877	2.13E-33	postive
DLAT	PERP	0.49623101	1.93E-32	postive
MTF1	PERP	0.420872812	6.95E-23	postive
DLD	PFKP	0.522053808	2.65E-36	postive
DLAT	PFKP	0.473474413	2.66E-29	postive
PDHA1	PFKP	0.488383079	2.48E-31	postive
DLD	PGAM4	0.460802337	1.19E-27	postive
DLAT	PGAM4	0.459390862	1.80E-27	postive
FDX1	PGAP3	0.423871615	3.20E-23	postive
LIAS	PGAP3	0.45521457	6.03E-27	postive
LIPT1	PGAP3	0.479609734	4.00E-30	postive
PDHA1	PGAP3	0.54070747	2.63E-39	postive
PDHB	PGAP3	0.52379872	1.41E-36	postive
MTF1	PGAP3	0.419453434	1.00E-22	postive
FDX1	PGM2	0.511134381	1.25E-34	postive

LIAS	PGM2	0.531840074	7.44E-38	postive
LIPT1	PGM2	0.449567087	3.02E-26	postive
DLD	PGM2	0.640345516	4.78E-59	postive
DLAT	PGM2	0.632360414	3.41E-57	postive
PDHB	PGM2	0.412822838	5.35E-22	postive
MTF1	PGM2	0.531029753	1.00E-37	postive
FDX1	PGPEP1	0.406305977	2.69E-21	postive
LIAS	PGPEP1	0.422451885	4.63E-23	postive
LIPT1	PGPEP1	0.525434535	7.82E-37	postive
PDHA1	PGPEP1	0.485153179	6.97E-31	postive
PDHB	PGPEP1	0.476866886	9.38E-30	postive
MTF1	PGPEP1	0.634743622	9.66E-58	postive
FDX1	PHF1	0.47097895	5.70E-29	postive
LIAS	PHF1	0.511003894	1.31E-34	postive
LIPT1	PHF1	0.572764564	6.29E-45	postive
DLD	PHF1	0.443020468	1.89E-25	postive
DLAT	PHF1	0.431988922	3.78E-24	postive
PDHA1	PHF1	0.667681165	7.96E-66	postive
PDHB	PHF1	0.597073725	1.27E-49	postive
MTF1	PHF1	0.533847032	3.52E-38	postive
FDX1	PHKG2	0.413055995	5.05E-22	postive
LIAS	PHKG2	0.488677973	2.26E-31	postive
LIPT1	PHKG2	0.540587983	2.76E-39	postive
PDHA1	PHKG2	0.624686231	1.83E-55	postive
PDHB	PHKG2	0.508005445	3.67E-34	postive
MTF1	PHKG2	0.422603143	4.45E-23	postive
LIPT1	PIK3CD	0.409763853	1.15E-21	postive
MTF1	PIK3CD	0.584925281	3.16E-47	postive
GLS	PIK3CD	0.451045594	1.99E-26	postive
MTF1	PIK3CG	0.478251646	6.11E-30	postive
MTF1	PIK3R1	0.616303478	1.25E-53	postive
LIPT1	PIK3R3	0.438547277	6.45E-25	postive
PDHA1	PIK3R3	0.450721591	2.18E-26	postive
MTF1	PIK3R3	0.629954554	1.20E-56	postive
DLD	PIMREG	0.487368154	3.44E-31	postive
DLAT	PIMREG	0.446525666	7.12E-26	postive
PDHA1	PIMREG	0.431322216	4.52E-24	postive
DLD	PKMYT1	0.481029262	2.56E-30	postive
DLAT	PKMYT1	0.427688737	1.18E-23	postive
PDHA1	PKMYT1	0.512891742	6.79E-35	postive
DLD	PLAUR	0.410600989	9.31E-22	postive
DLD	PLCD3	0.424931044	2.43E-23	postive
MTF1	PLCD3	0.437899035	7.70E-25	postive
DLD	PLEC	0.412076241	6.45E-22	postive
DLAT	PLEC	0.417637526	1.59E-22	postive
PDHA1	PLEC	0.468153944	1.34E-28	postive
MTF1	PLEC	0.597996865	8.29E-50	postive
MTF1	PLEK	0.425924272	1.88E-23	postive

MTF1	PLEKHA3P1	0.461541864	9.56E-28	postive
PDHA1	PLEKHB1	0.405657989	3.15E-21	postive
PDHB	PLEKHB1	0.414221392	3.77E-22	postive
MTF1	PLEKHH2	0.436479348	1.13E-24	postive
FDX1	PLIN3	0.482828856	1.46E-30	postive
LIAS	PLIN3	0.439129368	5.50E-25	postive
DLD	PLIN3	0.549389197	9.07E-41	postive
DLAT	PLIN3	0.565646037	1.26E-43	postive
PDHA1	PLIN3	0.447947202	4.78E-26	postive
PDHB	PLIN3	0.54074967	2.59E-39	postive
MTF1	PLIN3	0.473385311	2.74E-29	postive
DLD	PLK1	0.534142807	3.15E-38	postive
DLAT	PLK1	0.498542182	8.97E-33	postive
PDHA1	PLK1	0.49654305	1.74E-32	postive
DLD	PLK4	0.530470709	1.24E-37	postive
DLAT	PLK4	0.525033533	9.04E-37	postive
PDHA1	PLK4	0.455739193	5.19E-27	postive
MTF1	PLK4	0.40266406	6.51E-21	postive
LIPT1	PNISR	0.483015509	1.37E-30	postive
PDHA1	PNISR	0.515851217	2.41E-35	postive
MTF1	PNISR	0.581587333	1.38E-46	postive
FDX1	PNRC2	0.588122288	7.58E-48	postive
LIAS	PNRC2	0.552971431	2.20E-41	postive
LIPT1	PNRC2	0.699307164	1.25E-74	postive
DLD	PNRC2	0.561774509	6.24E-43	postive
DLAT	PNRC2	0.599149673	4.84E-50	postive
PDHA1	PNRC2	0.54950913	8.66E-41	postive
PDHB	PNRC2	0.613699712	4.53E-53	postive
MTF1	PNRC2	0.67230492	4.81E-67	postive
DLD	POLQ	0.454949525	6.51E-27	postive
PDHA1	POLQ	0.412253374	6.17E-22	postive
PDHA1	POU6F1	0.482320451	1.71E-30	postive
MTF1	POU6F1	0.534278861	3.00E-38	postive
FDX1	PPFIBP2	0.400046894	1.22E-20	postive
LIPT1	PPFIBP2	0.487764355	3.03E-31	postive
PDHA1	PPFIBP2	0.442186628	2.38E-25	postive
MTF1	PPFIBP2	0.433502709	2.52E-24	postive
FDX1	PPM1M	0.456150191	4.61E-27	postive
LIAS	PPM1M	0.431839917	3.94E-24	postive
LIPT1	PPM1M	0.569729212	2.28E-44	postive
PDHA1	PPM1M	0.413740442	4.25E-22	postive
PDHB	PPM1M	0.558761926	2.14E-42	postive
MTF1	PPM1M	0.417318796	1.72E-22	postive
FDX1	PPOX	0.440250235	4.05E-25	postive
LIAS	PPOX	0.550459692	5.95E-41	postive
LIPT1	PPOX	0.603418807	6.49E-51	postive
PDHA1	PPOX	0.609796762	3.05E-52	postive
PDHB	PPOX	0.588166777	7.43E-48	postive

PDHA1	PPP1R13B	0.507448417	4.45E-34	postive
PDHB	PPP1R13B	0.47067797	6.25E-29	postive
MTF1	PPP1R13B	0.479481813	4.16E-30	postive
FDX1	PPP1R14B-AS 1	0.400255949	1.16E-20	postive
DLAT	PPP1R14B-AS 1	0.411815937	6.88E-22	postive
PDHA1	PPP1R14B-AS 1	0.411195932	8.03E-22	postive
PDHB	PPP1R14B-AS 1	0.423599451	3.44E-23	postive
MTF1	PPP1R16B	0.488726398	2.22E-31	postive
LIPT1	PPP1R3E	0.443274424	1.76E-25	postive
PDHA1	PPP1R3E	0.510547932	1.53E-34	postive
MTF1	PPP1R3E	0.423822163	3.24E-23	postive
DLD	PPT2	0.406813199	2.37E-21	postive
DLAT	PPT2	0.413139578	4.94E-22	postive
PDHA1	PPT2	0.42976457	6.83E-24	postive
MTF1	PPT2	0.429493795	7.34E-24	postive
DLD	PRC1	0.558069969	2.83E-42	postive
DLAT	PRC1	0.54752756	1.88E-40	postive
PDHA1	PRC1	0.539402642	4.33E-39	postive
LIPT1	PRCD	0.447655238	5.18E-26	postive
FDX1	PRDM2	0.415247656	2.91E-22	postive
LIPT1	PRDM2	0.544009267	7.40E-40	postive
DLD	PRDM2	0.444894972	1.12E-25	postive
DLAT	PRDM2	0.519885861	5.76E-36	postive
PDHA1	PRDM2	0.488329903	2.53E-31	postive
PDHB	PRDM2	0.406870871	2.34E-21	postive
MTF1	PRDM2	0.828903572	9.92E-128	postive
MTF1	PREX2	0.432465548	3.33E-24	postive
MTF1	PRICKLE2	0.562730197	4.21E-43	postive
MTF1	PRKCB	0.457367272	3.24E-27	postive
LIPT1	PRKCD	0.4173108	1.73E-22	postive
DLAT	PRKCD	0.422740742	4.29E-23	postive
PDHA1	PRKCD	0.564516466	2.01E-43	postive
PDHB	PRKCD	0.628514369	2.54E-56	postive
MTF1	PRKCD	0.521250553	3.54E-36	postive
LIAS	PRKCZ	0.424668969	2.60E-23	postive
LIPT1	PRKCZ	0.420441508	7.76E-23	postive
DLAT	PRKCZ	0.417355255	1.71E-22	postive
PDHA1	PRKCZ	0.424042004	3.06E-23	postive
PDHB	PRKCZ	0.433941939	2.24E-24	postive
MTF1	PRKCZ	0.444398725	1.29E-25	postive
DLD	PRR11	0.496526393	1.75E-32	postive
DLAT	PRR11	0.490127635	1.41E-31	postive
PDHA1	PRR11	0.406381658	2.64E-21	postive
MTF1	PRRT3	0.479440162	4.22E-30	postive

FDX1	PSMD11	0.456088779	4.69E-27	postive
LIAS	PSMD11	0.463675031	5.09E-28	postive
LIPT1	PSMD11	0.470873777	5.88E-29	postive
DLD	PSMD11	0.663228104	1.13E-64	postive
DLAT	PSMD11	0.631578171	5.14E-57	postive
PDHA1	PSMD11	0.697858585	3.36E-74	postive
PDHB	PSMD11	0.561599192	6.71E-43	postive
MTF1	PSMD11	0.543558692	8.81E-40	postive
FDX1	PSMD2	0.479480401	4.16E-30	postive
LIAS	PSMD2	0.436233327	1.21E-24	postive
LIPT1	PSMD2	0.460314394	1.37E-27	postive
DLD	PSMD2	0.662874046	1.40E-64	postive
DLAT	PSMD2	0.640013067	5.73E-59	postive
PDHA1	PSMD2	0.654066299	2.32E-62	postive
PDHB	PSMD2	0.54679274	2.51E-40	postive
MTF1	PSMD2	0.614348886	3.29E-53	postive
MTF1	PTK2B	0.561231541	7.80E-43	postive
FDX1	PTPN6	0.535279691	2.06E-38	postive
LIAS	PTPN6	0.482194301	1.78E-30	postive
LIPT1	PTPN6	0.550033283	7.04E-41	postive
DLD	PTPN6	0.477095832	8.74E-30	postive
DLAT	PTPN6	0.426849693	1.47E-23	postive
PDHA1	PTPN6	0.570264021	1.82E-44	postive
PDHB	PTPN6	0.556041539	6.42E-42	postive
MTF1	PTPN6	0.500154684	5.24E-33	postive
PDHA1	PTTG1	0.44622823	7.74E-26	postive
DLD	PVR	0.531060251	9.93E-38	postive
DLAT	PVR	0.587132832	1.18E-47	postive
PDHA1	PVR	0.589696706	3.73E-48	postive
PDHB	PVR	0.51844518	9.62E-36	postive
MTF1	PVR	0.5570423	4.29E-42	postive
LIPT1	PXK	0.486675253	4.29E-31	postive
DLD	PXK	0.47317779	2.92E-29	postive
DLAT	PXK	0.491391584	9.39E-32	postive
PDHA1	PXK	0.407702352	1.91E-21	postive
PDHB	PXK	0.54513151	4.79E-40	postive
MTF1	PXK	0.644448335	5.09E-60	postive
GLS	PXK	0.504701417	1.13E-33	postive
LIPT1	RAB40B	0.463802673	4.91E-28	postive
PDHA1	RAB40B	0.536502844	1.30E-38	postive
PDHB	RAB40B	0.501770775	3.05E-33	postive
DLD	RAC1P2	0.475613735	1.38E-29	postive
DLAT	RAC1P2	0.448432254	4.17E-26	postive
PDHA1	RAC1P2	0.465798092	2.71E-28	postive
PDHB	RAC1P2	0.408402569	1.60E-21	postive
MTF1	RAC1P2	0.45500348	6.41E-27	postive
DLD	RACGAP1	0.600737486	2.30E-50	postive
DLAT	RACGAP1	0.584776988	3.38E-47	postive

PDHA1	RACGAP1	0.498269283	9.82E-33	postive
PDHB	RACGAP1	0.423818804	3.25E-23	postive
DLD	RAD51	0.577922903	6.85E-46	postive
DLAT	RAD51	0.527428472	3.78E-37	postive
PDHA1	RAD51	0.458695395	2.20E-27	postive
MTF1	RAI2	0.450034343	2.65E-26	postive
LIPT1	RALGPS1	0.474053146	2.23E-29	postive
PDHA1	RALGPS1	0.448009837	4.69E-26	postive
MTF1	RALGPS1	0.554797352	1.06E-41	postive
MTF1	RASGRP4	0.51285712	6.87E-35	postive
GLS	RASGRP4	0.410917433	8.61E-22	postive
LIPT1	RASSF1-AS1	0.421892644	5.34E-23	postive
PDHA1	RASSF1-AS1	0.456398357	4.29E-27	postive
PDHB	RASSF1-AS1	0.416217491	2.28E-22	postive
MTF1	RASSF2	0.549675492	8.11E-41	postive
FDX1	RBL2	0.462637497	6.92E-28	postive
LIAS	RBL2	0.463159582	5.93E-28	postive
LIPT1	RBL2	0.50013711	5.27E-33	postive
DLD	RBL2	0.453834165	8.97E-27	postive
DLAT	RBL2	0.52744068	3.76E-37	postive
PDHA1	RBL2	0.441168947	3.15E-25	postive
PDHB	RBL2	0.486535337	4.49E-31	postive
MTF1	RBL2	0.663776416	8.19E-65	postive
PDHA1	RBM6	0.548182671	1.46E-40	postive
PDHB	RBM6	0.431590425	4.21E-24	postive
MTF1	RBM6	0.557965459	2.95E-42	postive
LIPT1	RBP5	0.450208449	2.52E-26	postive
MTF1	RBP5	0.403779778	4.97E-21	postive
FDX1	RCBTB2	0.430174602	6.13E-24	postive
LIPT1	RCBTB2	0.49200297	7.70E-32	postive
PDHB	RCBTB2	0.403384051	5.47E-21	postive
MTF1	RCBTB2	0.542313933	1.42E-39	postive
GLS	RCBTB2	0.400004436	1.24E-20	postive
MTF1	RCSD1	0.458181838	2.56E-27	postive
FDX1	REV1	0.429950378	6.50E-24	postive
LIAS	REV1	0.466106819	2.47E-28	postive
LIPT1	REV1	0.630123292	1.10E-56	postive
DLD	REV1	0.419084338	1.10E-22	postive
DLAT	REV1	0.492049886	7.58E-32	postive
PDHA1	REV1	0.559981375	1.30E-42	postive
PDHB	REV1	0.490211683	1.38E-31	postive
MTF1	REV1	0.670472066	1.47E-66	postive
DLAT	REV3L	0.448887326	3.66E-26	postive
MTF1	REV3L	0.737839743	4.68E-87	postive
PDHB	RFTN1	0.473656836	2.52E-29	postive
LIAS	RFXAP	0.420990706	6.74E-23	postive
LIPT1	RFXAP	0.613536663	4.91E-53	postive
DLD	RFXAP	0.448510372	4.08E-26	postive

DLAT	RFXAP	0.46989183	7.92E-29	postive
PDHA1	RFXAP	0.482176194	1.79E-30	postive
PDHB	RFXAP	0.511126863	1.25E-34	postive
MTF1	RFXAP	0.543214927	1.01E-39	postive
LIPT1	RGL1	0.44712826	6.01E-26	postive
MTF1	RGL1	0.550429083	6.02E-41	postive
GLS	RGL1	0.437938527	7.61E-25	postive
MTF1	RGPD8	0.486820922	4.10E-31	postive
PDHB	RHOBTB2	0.428412596	9.76E-24	postive
FDX1	RHOF	0.458563845	2.29E-27	postive
DLD	RHOF	0.513924073	4.73E-35	postive
DLAT	RHOF	0.483483121	1.18E-30	postive
PDHA1	RHOF	0.403038417	5.95E-21	postive
FDX1	RHOQ	0.499855159	5.79E-33	postive
LIPT1	RHOQ	0.566750021	7.96E-44	postive
DLD	RHOQ	0.541367911	2.05E-39	postive
DLAT	RHOQ	0.575528235	1.93E-45	postive
PDHA1	RHOQ	0.502742585	2.20E-33	postive
PDHB	RHOQ	0.554934322	1.00E-41	postive
MTF1	RHOQ	0.524736077	1.01E-36	postive
FDX1	RHOQP2	0.40969128	1.17E-21	postive
LIPT1	RHOQP2	0.439326031	5.22E-25	postive
DLD	RHOQP2	0.476718135	9.82E-30	postive
DLAT	RHOQP2	0.525393206	7.94E-37	postive
PDHB	RHOQP2	0.431136196	4.75E-24	postive
MTF1	RHOQP2	0.499233332	7.13E-33	postive
DLD	RHPN2	0.471265188	5.23E-29	postive
DLAT	RHPN2	0.480186499	3.34E-30	postive
PDHA1	RHPN2	0.452230869	1.42E-26	postive
MTF1	RHPN2	0.408782183	1.46E-21	postive
FDX1	RILPL2	0.517993943	1.13E-35	postive
LIPT1	RILPL2	0.50475613	1.11E-33	postive
PDHA1	RILPL2	0.4343794	1.99E-24	postive
PDHB	RILPL2	0.445419156	9.70E-26	postive
MTF1	RILPL2	0.562899653	3.93E-43	postive
FDX1	RIPK2	0.40664543	2.47E-21	postive
DLD	RIPK2	0.559560855	1.54E-42	postive
DLAT	RIPK2	0.545629185	3.95E-40	postive
PDHA1	RIPK2	0.469843136	8.04E-29	postive
PDHB	RIPK2	0.468686739	1.14E-28	postive
MTF1	RIPK2	0.413657586	4.34E-22	postive
FDX1	RIPK3	0.433678193	2.41E-24	postive
LIAS	RIPK3	0.416414027	2.17E-22	postive
LIPT1	RIPK3	0.488527104	2.37E-31	postive
PDHA1	RIPK3	0.406149152	2.79E-21	postive
PDHB	RIPK3	0.429954006	6.50E-24	postive
LIAS	RMDN2	0.428816389	8.78E-24	postive
LIPT1	RMDN2	0.485250452	6.76E-31	postive

PDHB	RMDN2	0.434809078	1.78E-24	postive
MTF1	RN7SL268P	0.427536519	1.23E-23	postive
MTF1	RNA5SP317	0.457284384	3.32E-27	postive
MTF1	RNF125	0.472702094	3.37E-29	postive
FDX1	RNF130	0.511633922	1.05E-34	postive
LIPT1	RNF130	0.531591241	8.16E-38	postive
DLD	RNF130	0.424759646	2.54E-23	postive
DLAT	RNF130	0.433707456	2.39E-24	postive
PDHA1	RNF130	0.513392155	5.70E-35	postive
PDHB	RNF130	0.531231477	9.32E-38	postive
MTF1	RNF130	0.507038041	5.12E-34	postive
FDX1	RNF144B	0.40537907	3.37E-21	postive
LIPT1	RNF144B	0.448743887	3.82E-26	postive
MTF1	RNF144B	0.548610952	1.23E-40	postive
LIPT1	RNFT1-DT	0.400163807	1.19E-20	postive
MTF1	RNU6-1262P	0.431998034	3.77E-24	postive
LIPT1	RNU6-8	0.410696316	9.10E-22	postive
FDX1	ROM1	0.401256564	9.14E-21	postive
LIAS	ROM1	0.425953362	1.86E-23	postive
LIPT1	ROM1	0.467710728	1.53E-28	postive
PDHA1	ROM1	0.499222972	7.15E-33	postive
PDHB	ROM1	0.466336018	2.31E-28	postive
MTF1	RORA	0.532747502	5.31E-38	postive
LIPT1	RP11-15H20.6	0.475564225	1.40E-29	postive
DLD	RP11-267M23.1	0.428737993	8.96E-24	postive
PDHA1	RP11-267M23.1	0.439868276	4.50E-25	postive
GLS	RP11-462G12.2	0.459050209	1.98E-27	postive
PDHA1	RP11-876N24.5	0.408072557	1.74E-21	postive
MTF1	RP11-876N24.5	0.601863536	1.35E-50	postive
FDX1	RPARP-AS1	0.423650201	3.39E-23	postive
LIAS	RPARP-AS1	0.589344573	4.37E-48	postive
LIPT1	RPARP-AS1	0.60044298	2.64E-50	postive
PDHA1	RPARP-AS1	0.473035806	3.05E-29	postive
PDHB	RPARP-AS1	0.507761763	3.99E-34	postive
MTF1	RPL18AP7	0.478839788	5.08E-30	postive
MTF1	RPL23AP64	0.544977214	5.09E-40	postive
MTF1	RPL5P30	0.510427462	1.60E-34	postive
FDX1	RPS6KA1	0.568568516	3.71E-44	postive
LIAS	RPS6KA1	0.514981391	3.27E-35	postive
LIPT1	RPS6KA1	0.5676746	5.41E-44	postive
DLD	RPS6KA1	0.442316142	2.29E-25	postive
DLAT	RPS6KA1	0.426148931	1.77E-23	postive
PDHA1	RPS6KA1	0.544214684	6.84E-40	postive

PDHB	RPS6KA1	0.54863121	1.22E-40	postive
MTF1	RPS6KA1	0.544171529	6.95E-40	postive
LIPT1	RPS6KA5	0.474759419	1.80E-29	postive
MTF1	RPS6KA5	0.534156526	3.14E-38	postive
DLD	RRM2	0.513694709	5.13E-35	postive
DLAT	RRM2	0.471086855	5.52E-29	postive
LIAS	RTCA-AS1	0.427115031	1.37E-23	postive
LIPT1	RTCA-AS1	0.52150526	3.23E-36	postive
MTF1	RTCA-AS1	0.445236643	1.02E-25	postive
MTF1	RTL5	0.440556943	3.72E-25	postive
MTF1	RTN1	0.417074767	1.83E-22	postive
GLS	RTN1	0.416417122	2.17E-22	postive
MTF1	RUBCNL	0.460782173	1.20E-27	postive
LIAS	RUFY3	0.443803107	1.52E-25	postive
LIPT1	RUFY3	0.511785466	9.97E-35	postive
DLAT	RUFY3	0.436534139	1.11E-24	postive
PDHA1	RUFY3	0.495858096	2.18E-32	postive
PDHB	RUFY3	0.428102857	1.06E-23	postive
MTF1	RUFY3	0.589022817	5.05E-48	postive
GLS	RUNDC3A-AS1	0.402725037	6.42E-21	postive
FDX1	S100A10	0.439896921	4.46E-25	postive
LIAS	S100A10	0.400286205	1.15E-20	postive
FDX1	S100A16	0.44901042	3.54E-26	postive
DLAT	S100A16	0.400512874	1.09E-20	postive
PDHB	S100A16	0.414319369	3.68E-22	postive
FDX1	SARAF	0.55752937	3.52E-42	postive
LIAS	SARAF	0.464585776	3.89E-28	postive
LIPT1	SARAF	0.488340545	2.52E-31	postive
DLD	SARAF	0.471913886	4.29E-29	postive
DLAT	SARAF	0.506853472	5.45E-34	postive
PDHA1	SARAF	0.436981364	9.88E-25	postive
PDHB	SARAF	0.631185801	6.31E-57	postive
MTF1	SARAF	0.473060123	3.02E-29	postive
LIPT1	SARM1	0.439614402	4.82E-25	postive
PDHA1	SARM1	0.427810908	1.14E-23	postive
MTF1	SARM1	0.58458489	3.68E-47	postive
FDX1	SAT2	0.470590184	6.41E-29	postive
LIAS	SAT2	0.494911171	2.98E-32	postive
LIPT1	SAT2	0.475331721	1.51E-29	postive
DLD	SAT2	0.413422193	4.61E-22	postive
PDHA1	SAT2	0.567514091	5.78E-44	postive
PDHB	SAT2	0.657393454	3.43E-63	postive
MTF1	SATB1	0.447946452	4.78E-26	postive
FDX1	SCAMP1-AS1	0.494095803	3.89E-32	postive
LIAS	SCAMP1-AS1	0.601824605	1.38E-50	postive
LIPT1	SCAMP1-AS1	0.656194153	6.85E-63	postive
DLD	SCAMP1-AS1	0.427594293	1.21E-23	postive
PDHA1	SCAMP1-AS1	0.46725307	1.75E-28	postive

PDHB	SCAMP1-AS1	0.574454984	3.05E-45	postive
MTF1	SCARF1	0.459903446	1.55E-27	postive
FDX1	SCMH1	0.458335215	2.44E-27	postive
LIAS	SCMH1	0.420521948	7.61E-23	postive
LIPT1	SCMH1	0.524309684	1.18E-36	postive
DLD	SCMH1	0.467461921	1.65E-28	postive
DLAT	SCMH1	0.508850599	2.75E-34	postive
PDHA1	SCMH1	0.637247377	2.54E-58	postive
PDHB	SCMH1	0.531494712	8.45E-38	postive
MTF1	SCMH1	0.671323098	8.76E-67	postive
GLS	SCN2B	0.428941393	8.49E-24	postive
LIPT1	SCPEP1	0.525754515	6.96E-37	postive
MTF1	SCPEP1	0.425578634	2.05E-23	postive
PDHB	SEC11C	0.468902412	1.07E-28	postive
PDHB	SELENBP1	0.413433369	4.59E-22	postive
FDX1	SELENOK	0.54016917	3.24E-39	postive
LIAS	SELENOK	0.523274267	1.71E-36	postive
LIPT1	SELENOK	0.612585238	7.83E-53	postive
DLD	SELENOK	0.446883473	6.44E-26	postive
DLAT	SELENOK	0.47158728	4.74E-29	postive
PDHA1	SELENOK	0.475317519	1.51E-29	postive
PDHB	SELENOK	0.786767457	2.02E-106	postive
FDX1	SELENOP	0.437114097	9.53E-25	postive
LIPT1	SELENOP	0.47242021	3.68E-29	postive
LIPT1	SEMA4A	0.407653523	1.93E-21	postive
PDHA1	SEMA4A	0.451149437	1.93E-26	postive
PDHB	SEMA4A	0.452597331	1.28E-26	postive
MTF1	SEMA4A	0.452042043	1.50E-26	postive
DLD	SEMA4B	0.417522391	1.64E-22	postive
DLAT	SEMA4B	0.492401679	6.76E-32	postive
PDHA1	SEMA4B	0.512841342	6.91E-35	postive
PDHB	SEMA4B	0.478230716	6.15E-30	postive
MTF1	SEMA4B	0.427101319	1.38E-23	postive
MTF1	SEMA6D	0.454645203	7.11E-27	postive
FDX1	SENP8	0.558315312	2.56E-42	postive
LIAS	SENP8	0.603902187	5.16E-51	postive
LIPT1	SENP8	0.622912091	4.52E-55	postive
DLD	SENP8	0.424470712	2.74E-23	postive
DLAT	SENP8	0.505575849	8.43E-34	postive
PDHB	SENP8	0.512656885	7.36E-35	postive
MTF1	SENP8	0.46570272	2.79E-28	postive
LIAS	SEPSECS-AS1	0.461034675	1.11E-27	postive
LIPT1	SEPSECS-AS1	0.515235003	2.99E-35	postive
PDHA1	SEPSECS-AS1	0.465603601	2.87E-28	postive
PDHB	SEPSECS-AS1	0.420193685	8.27E-23	postive
MTF1	SEPTIN4	0.425338145	2.19E-23	postive
LIPT1	SERF1B	0.488274494	2.57E-31	postive
DLD	SERF1B	0.407021089	2.25E-21	postive

DLAT	SERF1B	0.440149415	4.16E-25	postive
PDHB	SERF1B	0.51078581	1.41E-34	postive
FDX1	SESN1	0.422795062	4.23E-23	postive
LIPT1	SESN1	0.474939628	1.70E-29	postive
MTF1	SESN1	0.462153566	7.99E-28	postive
GLS	SESN3	0.406164119	2.78E-21	postive
MTF1	SETBP1	0.446229194	7.73E-26	postive
FDX1	SETDB2	0.465159526	3.28E-28	postive
LIAS	SETDB2	0.510497375	1.56E-34	postive
LIPT1	SETDB2	0.589413397	4.23E-48	postive
DLD	SETDB2	0.404794552	3.88E-21	postive
DLAT	SETDB2	0.429740316	6.88E-24	postive
PDHA1	SETDB2	0.410473908	9.61E-22	postive
PDHB	SETDB2	0.481509052	2.21E-30	postive
MTF1	SETDB2	0.618695417	3.79E-54	postive
DLD	SGO1	0.504843219	1.08E-33	postive
DLAT	SGO1	0.477086669	8.76E-30	postive
DLD	SGO2	0.603843547	5.31E-51	postive
DLAT	SGO2	0.594318912	4.54E-49	postive
MTF1	SGO2	0.438529492	6.48E-25	postive
DLAT	SH3BP5	0.422402666	4.69E-23	postive
PDHB	SH3BP5	0.454453958	7.51E-27	postive
MTF1	SH3BP5	0.575186389	2.23E-45	postive
MTF1	SH3BP5-AS1	0.449406032	3.17E-26	postive
MTF1	SH3TC2	0.410169864	1.04E-21	postive
FDX1	SHC1	0.42770894	1.18E-23	postive
LIAS	SHC1	0.428746625	8.94E-24	postive
LIPT1	SHC1	0.411295577	7.84E-22	postive
DLD	SHC1	0.56104758	8.41E-43	postive
DLAT	SHC1	0.570695761	1.51E-44	postive
PDHA1	SHC1	0.617998267	5.38E-54	postive
PDHB	SHC1	0.585345558	2.62E-47	postive
MTF1	SHC1	0.53611534	1.50E-38	postive
DLD	SHCBP1	0.538568269	5.95E-39	postive
DLAT	SHCBP1	0.485336222	6.58E-31	postive
PDHA1	SHCBP1	0.445652389	9.09E-26	postive
FDX1	SIDT2	0.543888298	7.76E-40	postive
LIAS	SIDT2	0.40025077	1.16E-20	postive
LIPT1	SIDT2	0.454273571	7.91E-27	postive
DLD	SIDT2	0.416976068	1.88E-22	postive
DLAT	SIDT2	0.548563789	1.26E-40	postive
PDHA1	SIDT2	0.559950214	1.32E-42	postive
PDHB	SIDT2	0.517956831	1.14E-35	postive
MTF1	SIDT2	0.638744636	1.14E-58	postive
LIAS	SIRT4	0.463686944	5.08E-28	postive
LIPT1	SIRT4	0.534340083	2.93E-38	postive
PDHA1	SIRT4	0.47966996	3.92E-30	postive
PDHB	SIRT4	0.548449637	1.31E-40	postive

DLAT	SIX5	0.421867779	5.38E-23	postive
PDHA1	SIX5	0.572595429	6.76E-45	postive
PDHB	SIX5	0.509499978	2.20E-34	postive
MTF1	SIX5	0.446887951	6.43E-26	postive
DLD	SKA1	0.515482468	2.74E-35	postive
DLAT	SKA1	0.466278833	2.35E-28	postive
PDHA1	SKA1	0.426041516	1.82E-23	postive
DLD	SKA3	0.520671732	4.35E-36	postive
DLAT	SKA3	0.4755312	1.42E-29	postive
PDHA1	SKA3	0.423892473	3.19E-23	postive
MTF1	SLA	0.460754141	1.21E-27	postive
DLD	SLC16A3	0.454615	7.17E-27	postive
DLAT	SLC16A3	0.471840794	4.39E-29	postive
PDHA1	SLC16A3	0.435507548	1.47E-24	postive
MTF1	SLC16A3	0.474317112	2.06E-29	postive
PDHA1	SLC22A23	0.432270558	3.51E-24	postive
MTF1	SLC22A23	0.483260714	1.27E-30	postive
MTF1	SLC24A3	0.408206882	1.68E-21	postive
LIAS	SLC25A16	0.476559499	1.03E-29	postive
LIPT1	SLC25A16	0.465613782	2.86E-28	postive
PDHA1	SLC25A16	0.40001543	1.23E-20	postive
PDHB	SLC25A16	0.478153068	6.30E-30	postive
FDX1	SLC25A33	0.554510511	1.19E-41	postive
LIAS	SLC25A33	0.42354732	3.48E-23	postive
LIPT1	SLC25A33	0.580301285	2.43E-46	postive
DLD	SLC25A33	0.524959222	9.29E-37	postive
DLAT	SLC25A33	0.558836404	2.07E-42	postive
PDHA1	SLC25A33	0.581864786	1.22E-46	postive
PDHB	SLC25A33	0.53259276	5.62E-38	postive
LIPT1	SLC25A42	0.461152142	1.07E-27	postive
PDHA1	SLC25A42	0.469076821	1.01E-28	postive
PDHB	SLC25A42	0.469110339	1.00E-28	postive
MTF1	SLC25A42	0.477863888	6.89E-30	postive
FDX1	SLC25A5-AS1	0.419229057	1.06E-22	postive
LIAS	SLC25A5-AS1	0.471369101	5.06E-29	postive
LIPT1	SLC25A5-AS1	0.557474466	3.60E-42	postive
PDHA1	SLC25A5-AS1	0.532147948	6.63E-38	postive
PDHB	SLC25A5-AS1	0.40947161	1.23E-21	postive
MTF1	SLC25A5-AS1	0.45741715	3.19E-27	postive
FDX1	SLC26A11	0.41436171	3.64E-22	postive
LIPT1	SLC26A11	0.515398577	2.82E-35	postive
DLD	SLC26A11	0.423045933	3.97E-23	postive
DLAT	SLC26A11	0.439090628	5.56E-25	postive
PDHA1	SLC26A11	0.629460662	1.55E-56	postive
PDHB	SLC26A11	0.516928683	1.65E-35	postive
MTF1	SLC26A11	0.529040737	2.09E-37	postive
DLD	SLC2A1	0.46734244	1.71E-28	postive
DLAT	SLC2A1	0.44597089	8.31E-26	postive

LIAS	SLC35E2B	0.402697635	6.46E-21	postive
LIPT1	SLC35E2B	0.472419819	3.68E-29	postive
DLAT	SLC35E2B	0.446708025	6.76E-26	postive
PDHA1	SLC35E2B	0.507807427	3.93E-34	postive
PDHB	SLC35E2B	0.428312692	1.00E-23	postive
MTF1	SLC35E2B	0.687393069	3.52E-71	postive
GLS	SLC43A3	0.415553547	2.69E-22	postive
FDX1	SLC46A3	0.40187959	7.87E-21	postive
DLD	SLC6A8	0.416015357	2.40E-22	postive
PDHA1	SLC6A8	0.430253452	6.00E-24	postive
MTF1	SLFNL1	0.501428845	3.42E-33	postive
MTF1	SLIT3	0.425401795	2.15E-23	postive
LIPT1	SMAP2	0.411318577	7.79E-22	postive
PDHA1	SMAP2	0.449068696	3.48E-26	postive
PDHB	SMAP2	0.44602089	8.20E-26	postive
MTF1	SMAP2	0.64870969	4.78E-61	postive
PDHB	SMARCD3	0.44209759	2.44E-25	postive
FDX1	SMDT1	0.586071226	1.90E-47	postive
LIAS	SMDT1	0.570900974	1.39E-44	postive
LIPT1	SMDT1	0.594265073	4.65E-49	postive
DLD	SMDT1	0.430317288	5.90E-24	postive
DLAT	SMDT1	0.423058647	3.95E-23	postive
PDHA1	SMDT1	0.530953725	1.03E-37	postive
PDHB	SMDT1	0.682916403	6.30E-70	postive
FDX1	SMS	0.492632114	6.27E-32	postive
LIAS	SMS	0.414292217	3.70E-22	postive
LIPT1	SMS	0.419239155	1.06E-22	postive
DLD	SMS	0.626622606	6.76E-56	postive
DLAT	SMS	0.614903767	2.50E-53	postive
PDHA1	SMS	0.697664276	3.83E-74	postive
PDHB	SMS	0.490607984	1.21E-31	postive
MTF1	SMS	0.493896503	4.15E-32	postive
LIPT1	SNAI3	0.428438472	9.70E-24	postive
LIAS	SNHG19	0.402194323	7.29E-21	postive
LIPT1	SNHG19	0.403748336	5.01E-21	postive
PDHA1	SNHG19	0.491783974	8.27E-32	postive
PDHB	SNHG19	0.478340782	5.94E-30	postive
FDX1	SNN	0.431095922	4.80E-24	postive
LIPT1	SNN	0.516352879	2.02E-35	postive
DLD	SNN	0.498103244	1.04E-32	postive
DLAT	SNN	0.54705344	2.27E-40	postive
PDHA1	SNN	0.586929165	1.29E-47	postive
PDHB	SNN	0.5482569	1.42E-40	postive
MTF1	SNN	0.618093035	5.13E-54	postive
LIPT1	SNORD104	0.402105166	7.45E-21	postive
PDHA1	SNORD104	0.428192095	1.03E-23	postive
LIAS	SNX22	0.410338343	9.94E-22	postive
LIPT1	SNX22	0.433731496	2.37E-24	postive

PDHA1	SNX22	0.430273324	5.97E-24	postive
PDHB	SNX22	0.439288673	5.27E-25	postive
MTF1	SNX30	0.433513228	2.52E-24	postive
FDX1	SORT1	0.468869439	1.08E-28	postive
LIPT1	SORT1	0.408098034	1.73E-21	postive
DLD	SORT1	0.403708708	5.06E-21	postive
DLAT	SORT1	0.490652965	1.19E-31	postive
PDHA1	SORT1	0.447707739	5.11E-26	postive
PDHB	SORT1	0.428393363	9.81E-24	postive
MTF1	SORT1	0.596472277	1.68E-49	postive
FDX1	SOWAHC	0.467111557	1.83E-28	postive
LIAS	SOWAHC	0.426888906	1.46E-23	postive
LIPT1	SOWAHC	0.449180624	3.37E-26	postive
DLD	SOWAHC	0.569695565	2.31E-44	postive
DLAT	SOWAHC	0.554254521	1.32E-41	postive
PDHA1	SOWAHC	0.461873616	8.67E-28	postive
PDHB	SOWAHC	0.484556369	8.43E-31	postive
MTF1	SOWAHC	0.518570423	9.20E-36	postive
LIAS	SPACA9	0.409179059	1.32E-21	postive
PDHA1	SPACA9	0.444187296	1.37E-25	postive
PDHB	SPACA9	0.48597379	5.37E-31	postive
DLD	SPAG5	0.521303717	3.47E-36	postive
DLAT	SPAG5	0.490824398	1.13E-31	postive
PDHA1	SPAG5	0.542485009	1.33E-39	postive
MTF1	SPARCL1	0.455886422	4.97E-27	postive
LIPT1	SPATA13	0.443328468	1.73E-25	postive
PDHA1	SPATA13	0.412128521	6.37E-22	postive
MTF1	SPATA13	0.609598465	3.35E-52	postive
MTF1	SPATA6	0.483359042	1.23E-30	postive
DLD	SPC24	0.439383924	5.13E-25	postive
PDHA1	SPC24	0.464482815	4.01E-28	postive
DLD	SPC25	0.518373371	9.87E-36	postive
DLAT	SPC25	0.447031392	6.18E-26	postive
PDHA1	SPC25	0.425713834	1.98E-23	postive
LIAS	SPDL1	0.41390619	4.08E-22	postive
LIPT1	SPDL1	0.413878072	4.11E-22	postive
DLD	SPDL1	0.585347709	2.62E-47	postive
DLAT	SPDL1	0.569024264	3.07E-44	postive
PDHA1	SPDL1	0.548391159	1.34E-40	postive
PDHB	SPDL1	0.482237857	1.75E-30	postive
MTF1	SPDL1	0.452438182	1.34E-26	postive
DLD	SPECC1	0.475967133	1.24E-29	postive
DLAT	SPECC1	0.474094435	2.20E-29	postive
PDHA1	SPECC1	0.415619927	2.65E-22	postive
MTF1	SPECC1	0.492753196	6.03E-32	postive
LIPT1	SPNS3	0.431599842	4.20E-24	postive
LIPT1	ST3GAL6	0.432817463	3.03E-24	postive
MTF1	ST6GAL1	0.503769798	1.55E-33	postive

FDX1	ST6GALNAC6	0.400706641	1.04E-20	postive
LIAS	ST6GALNAC6	0.453332535	1.04E-26	postive
LIPT1	ST6GALNAC6	0.499023892	7.64E-33	postive
PDHA1	ST6GALNAC6	0.433098479	2.81E-24	postive
PDHB	ST6GALNAC6	0.569693412	2.31E-44	postive
MTF1	ST6GALNAC6	0.458034993	2.67E-27	postive
LIPT1	STK17B	0.486541025	4.48E-31	postive
DLAT	STK17B	0.426854238	1.47E-23	postive
PDHB	STK17B	0.430979544	4.95E-24	postive
MTF1	STK17B	0.429602143	7.13E-24	postive
GLS	STK32A	0.418095634	1.41E-22	postive
LIAS	STKLD1	0.413112333	4.98E-22	postive
LIPT1	STKLD1	0.495812536	2.21E-32	postive
PDHA1	STKLD1	0.488197085	2.64E-31	postive
PDHB	STKLD1	0.409896378	1.11E-21	postive
PDHA1	STPG3	0.407780307	1.87E-21	postive
MTF1	STRA6LP	0.48522891	6.81E-31	postive
FDX1	STX3	0.512747602	7.14E-35	postive
LIAS	STX3	0.452309301	1.39E-26	postive
LIPT1	STX3	0.554313969	1.28E-41	postive
DLD	STX3	0.580434233	2.29E-46	postive
DLAT	STX3	0.600750997	2.29E-50	postive
PDHA1	STX3	0.571444277	1.10E-44	postive
PDHB	STX3	0.526354785	5.59E-37	postive
MTF1	STX3	0.598611117	6.23E-50	postive
LIPT1	SUGT1P3	0.472453492	3.64E-29	postive
FDX1	SUOX	0.515400108	2.82E-35	postive
LIAS	SUOX	0.511075821	1.28E-34	postive
LIPT1	SUOX	0.570153799	1.91E-44	postive
DLD	SUOX	0.468411197	1.24E-28	postive
DLAT	SUOX	0.480689432	2.85E-30	postive
PDHA1	SUOX	0.646144333	1.99E-60	postive
PDHB	SUOX	0.63106284	6.73E-57	postive
MTF1	SUOX	0.484054961	9.88E-31	postive
LIPT1	SYDE2	0.408156675	1.70E-21	postive
DLAT	SYDE2	0.414641695	3.39E-22	postive
MTF1	SYDE2	0.490070609	1.44E-31	postive
MTF1	SYT11	0.530833312	1.08E-37	postive
LIPT1	SZT2	0.407748728	1.88E-21	postive
PDHA1	SZT2	0.548183561	1.46E-40	postive
MTF1	SZT2	0.748783522	5.39E-91	postive
DLD	TACC3	0.541090523	2.28E-39	postive
DLAT	TACC3	0.512041482	9.12E-35	postive
PDHA1	TACC3	0.614115273	3.69E-53	postive
PDHB	TACC3	0.410452744	9.66E-22	postive
MTF1	TACC3	0.418943975	1.14E-22	postive
LIPT1	TANC1	0.459794028	1.60E-27	postive
DLD	TANC1	0.437910739	7.67E-25	postive

DLAT	TANC1	0.509383018	2.29E-34	postive
PDHA1	TANC1	0.464986146	3.45E-28	postive
PDHB	TANC1	0.449187353	3.37E-26	postive
MTF1	TANC1	0.665249085	3.41E-65	postive
FDX1	TAPT1	0.429270591	7.78E-24	postive
LIAS	TAPT1	0.501210168	3.68E-33	postive
LIPT1	TAPT1	0.527462271	3.73E-37	postive
PDHA1	TAPT1	0.455779016	5.13E-27	postive
PDHB	TAPT1	0.465187241	3.25E-28	postive
MTF1	TAPT1	0.470499559	6.59E-29	postive
FDX1	TCEANC	0.510264584	1.69E-34	postive
LIAS	TCEANC	0.475573988	1.40E-29	postive
LIPT1	TCEANC	0.673333685	2.56E-67	postive
DLD	TCEANC	0.404786479	3.89E-21	postive
DLAT	TCEANC	0.420036926	8.61E-23	postive
PDHA1	TCEANC	0.563493955	3.08E-43	postive
PDHB	TCEANC	0.474275878	2.08E-29	postive
MTF1	TCEANC	0.533097373	4.66E-38	postive
PDHB	TCIM	0.446698248	6.78E-26	postive
FDX1	TCTA	0.486567105	4.44E-31	postive
LIAS	TCTA	0.47851838	5.62E-30	postive
LIPT1	TCTA	0.524442044	1.12E-36	postive
DLD	TCTA	0.42934597	7.63E-24	postive
DLAT	TCTA	0.459217062	1.89E-27	postive
PDHA1	TCTA	0.554038323	1.43E-41	postive
PDHB	TCTA	0.616955521	9.04E-54	postive
MTF1	TCTA	0.403377054	5.48E-21	postive
DLD	TEDC2	0.504689331	1.14E-33	postive
DLAT	TEDC2	0.469837368	8.06E-29	postive
PDHA1	TEDC2	0.578700976	4.88E-46	postive
PDHB	TEDC2	0.403426195	5.41E-21	postive
MTF1	TEK	0.448806027	3.75E-26	postive
DLD	TESMIN	0.408378739	1.61E-21	postive
LIPT1	TFEB	0.421140728	6.49E-23	postive
PDHA1	TFEB	0.48057874	2.95E-30	postive
PDHB	TFEB	0.454272746	7.91E-27	postive
MTF1	TFEB	0.458385802	2.41E-27	postive
FDX1	THRA	0.433898905	2.27E-24	postive
LIPT1	THRA	0.487492631	3.30E-31	postive
DLD	THRA	0.420699962	7.27E-23	postive
DLAT	THRA	0.435997983	1.29E-24	postive
PDHA1	THRA	0.557208478	4.01E-42	postive
PDHB	THRA	0.480515916	3.01E-30	postive
MTF1	THRA	0.635285375	7.24E-58	postive
MTF1	THSD1	0.496242475	1.92E-32	postive
FDX1	THYN1	0.599549805	4.02E-50	postive
LIAS	THYN1	0.533444372	4.09E-38	postive
LIPT1	THYN1	0.658719628	1.59E-63	postive

DLD	THYN1	0.415563893	2.69E-22	postive
DLAT	THYN1	0.522452783	2.30E-36	postive
PDHA1	THYN1	0.528326917	2.72E-37	postive
PDHB	THYN1	0.644847826	4.08E-60	postive
FDX1	TICAM1	0.457194724	3.40E-27	postive
LIAS	TICAM1	0.431980701	3.79E-24	postive
DLD	TICAM1	0.495827446	2.20E-32	postive
DLAT	TICAM1	0.47796821	6.67E-30	postive
PDHA1	TICAM1	0.529211622	1.96E-37	postive
PDHB	TICAM1	0.519709105	6.14E-36	postive
MTF1	TICAM1	0.499541456	6.43E-33	postive
DLD	TICRR	0.46318728	5.89E-28	postive
DLAT	TICRR	0.453705876	9.31E-27	postive
PDHA1	TICRR	0.484254562	9.27E-31	postive
MTF1	TICRR	0.415146316	2.99E-22	postive
DLD	TK1	0.480555505	2.98E-30	postive
DLAT	TK1	0.442509985	2.17E-25	postive
PDHA1	TK1	0.473379466	2.74E-29	postive
FDX1	TLE1	0.433956017	2.23E-24	postive
LIPT1	TLE1	0.414493773	3.52E-22	postive
DLD	TLE1	0.540559756	2.79E-39	postive
DLAT	TLE1	0.511843755	9.77E-35	postive
PDHA1	TLE1	0.517707182	1.25E-35	postive
PDHB	TLE1	0.421408171	6.06E-23	postive
MTF1	TLE1	0.524535079	1.08E-36	postive
DLD	TLE1P1	0.535394906	1.97E-38	postive
DLAT	TLE1P1	0.518874133	8.26E-36	postive
PDHA1	TLE1P1	0.414444698	3.56E-22	postive
MTF1	TLE1P1	0.478947347	4.92E-30	postive
PDHB	TLE2	0.43795037	7.59E-25	postive
MTF1	TLR2	0.408853828	1.44E-21	postive
GLS	TLR2	0.403068176	5.91E-21	postive
MTF1	TLR7	0.436423596	1.15E-24	postive
GLS	TLR7	0.416727074	2.00E-22	postive
MTF1	TM6SF1	0.442014935	2.49E-25	postive
GLS	TM6SF1	0.504572816	1.18E-33	postive
DLD	TMED7-TICAM2	0.475835397	1.29E-29	postive
DLAT	TMED7-TICAM2	0.527700279	3.42E-37	postive
MTF1	TMED7-TICAM2	0.476222602	1.14E-29	postive
FDX1	TMEM125	0.401166325	9.34E-21	postive
LIPT1	TMEM125	0.449807987	2.83E-26	postive
PDHB	TMEM125	0.4712228	5.29E-29	postive
LIAS	TMEM150A	0.434339799	2.02E-24	postive
LIPT1	TMEM150A	0.473944461	2.31E-29	postive
PDHA1	TMEM150A	0.525134412	8.72E-37	postive

PDHB	TMEM150A	0.566130097	1.03E-43	postive
LIPT1	TMEM170B	0.451484363	1.76E-26	postive
DLAT	TMEM170B	0.452526631	1.30E-26	postive
PDHB	TMEM170B	0.440755061	3.53E-25	postive
MTF1	TMEM170B	0.508570895	3.03E-34	postive
LIAS	TMEM241	0.401045422	9.62E-21	postive
LIPT1	TMEM241	0.528226359	2.82E-37	postive
DLD	TMEM241	0.497306082	1.35E-32	postive
DLAT	TMEM241	0.571721639	9.81E-45	postive
PDHA1	TMEM241	0.529964261	1.49E-37	postive
PDHB	TMEM241	0.573753888	4.12E-45	postive
MTF1	TMEM241	0.505111514	9.87E-34	postive
FDX1	TMEM243	0.403751478	5.00E-21	postive
LIAS	TMEM243	0.429847618	6.68E-24	postive
LIPT1	TMEM243	0.479791507	3.78E-30	postive
LIPT1	TMEM273	0.416058182	2.37E-22	postive
GLS	TMEM273	0.415571217	2.68E-22	postive
FDX1	TMEM50B	0.50853008	3.07E-34	postive
LIAS	TMEM50B	0.51790613	1.16E-35	postive
LIPT1	TMEM50B	0.624402111	2.11E-55	postive
DLAT	TMEM50B	0.443212181	1.79E-25	postive
PDHB	TMEM50B	0.567184913	6.63E-44	postive
LIPT1	TMEM8B	0.444570316	1.23E-25	postive
PDHA1	TMEM8B	0.509578838	2.14E-34	postive
PDHB	TMEM8B	0.413239027	4.82E-22	postive
MTF1	TMEM8B	0.544807522	5.43E-40	postive
FDX1	TMX4	0.416185699	2.30E-22	postive
LIPT1	TMX4	0.51221	8.60E-35	postive
DLD	TMX4	0.412753458	5.45E-22	postive
DLAT	TMX4	0.48934832	1.82E-31	postive
PDHB	TMX4	0.520050888	5.43E-36	postive
MTF1	TMX4	0.475055505	1.64E-29	postive
PDHB	TNFRSF10C	0.464631605	3.84E-28	postive
FDX1	TNFSF12	0.464797664	3.65E-28	postive
LIPT1	TNFSF12	0.455655399	5.31E-27	postive
PDHA1	TNFSF12	0.412615505	5.64E-22	postive
PDHB	TNFSF12	0.480253543	3.27E-30	postive
FDX1	TNFSF13	0.476728838	9.79E-30	postive
LIAS	TNFSF13	0.43134909	4.49E-24	postive
LIPT1	TNFSF13	0.445907918	8.46E-26	postive
PDHB	TNFSF13	0.52446521	1.11E-36	postive
MTF1	TNFSF13	0.427343489	1.29E-23	postive
GLS	TNIK	0.447506673	5.41E-26	postive
MTF1	TNK2-AS1	0.482812985	1.46E-30	postive
MTF1	TNRC18P1	0.511481781	1.11E-34	postive
DLD	TOP2A	0.476357459	1.10E-29	postive
DLAT	TOP2A	0.49852418	9.02E-33	postive
PDHA1	TOP2A	0.404685734	3.99E-21	postive

DLD	TPX2	0.477546776	7.60E-30	postive
DLAT	TPX2	0.451440069	1.78E-26	postive
PDHA1	TPX2	0.420306637	8.04E-23	postive
LIPT1	TRAF3IP2-AS1	0.541419059	2.01E-39	postive
PDHA1	TRAF3IP2-AS1	0.402984114	6.03E-21	postive
MTF1	TRAF3IP2-AS1	0.470747345	6.12E-29	postive
LIPT1	TRDMT1	0.508744975	2.85E-34	postive
DLD	TRDMT1	0.401991047	7.66E-21	postive
DLAT	TRDMT1	0.453124182	1.10E-26	postive
PDHB	TRDMT1	0.477926609	6.75E-30	postive
MTF1	TRDMT1	0.461929232	8.53E-28	postive
MTF1	TREML2	0.405161192	3.55E-21	postive
LIPT1	TRIM24	0.440037655	4.29E-25	postive
DLD	TRIM24	0.521222651	3.57E-36	postive
DLAT	TRIM24	0.490412447	1.29E-31	postive
PDHA1	TRIM24	0.529058746	2.08E-37	postive
PDHB	TRIM24	0.442721328	2.05E-25	postive
MTF1	TRIM24	0.581754942	1.28E-46	postive
FDX1	TRIM28	0.440434665	3.85E-25	postive
LIAS	TRIM28	0.415047847	3.06E-22	postive
LIPT1	TRIM28	0.449578791	3.01E-26	postive
DLD	TRIM28	0.585322761	2.65E-47	postive
DLAT	TRIM28	0.586358303	1.67E-47	postive
PDHA1	TRIM28	0.698634687	1.98E-74	postive
PDHB	TRIM28	0.628090289	3.17E-56	postive
MTF1	TRIM28	0.533505352	4.00E-38	postive
LIPT1	TRIM62	0.487596975	3.19E-31	postive
DLD	TRIM62	0.463887442	4.78E-28	postive
DLAT	TRIM62	0.454976017	6.46E-27	postive
PDHA1	TRIM62	0.588125876	7.56E-48	postive
PDHB	TRIM62	0.469387911	9.23E-29	postive
MTF1	TRIM62	0.703988663	4.96E-76	postive
DLD	TRIP13	0.494594352	3.30E-32	postive
DLAT	TRIP13	0.474602024	1.89E-29	postive
PDHA1	TRIP13	0.400734427	1.04E-20	postive
LIAS	TRMT10B	0.427544907	1.23E-23	postive
LIPT1	TRMT10B	0.546861727	2.44E-40	postive
PDHA1	TRMT10B	0.54055325	2.79E-39	postive
PDHB	TRMT10B	0.452888788	1.18E-26	postive
MTF1	TRMT10B	0.588518214	6.34E-48	postive
DLD	TROAP	0.408880559	1.43E-21	postive
PDHA1	TROAP	0.4680624	1.38E-28	postive
MTF1	TSPOAP1	0.4093	1.29E-21	postive
MTF1	TSPOAP1-AS1	0.452286298	1.40E-26	postive
PDHA1	TSPYL2	0.567788543	5.15E-44	postive
PDHB	TSPYL2	0.409426011	1.25E-21	postive
MTF1	TSPYL2	0.537671768	8.36E-39	postive
LIPT1	TSPYL4	0.468674997	1.14E-28	postive

DLD	TSPYL4	0.458296991	2.47E-27	postive
DLAT	TSPYL4	0.471944646	4.25E-29	postive
PDHA1	TSPYL4	0.491556869	8.90E-32	postive
PDHB	TSPYL4	0.427461425	1.25E-23	postive
MTF1	TSPYL4	0.699523966	1.08E-74	postive
LIPT1	TSSK3	0.402978493	6.03E-21	postive
MTF1	TSSK3	0.493086966	5.41E-32	postive
MTF1	TTC21A	0.519337678	7.01E-36	postive
LIPT1	TTC23L	0.416273374	2.25E-22	postive
MTF1	TTC23L	0.408346344	1.63E-21	postive
DLD	TTK	0.504222215	1.33E-33	postive
DLAT	TTK	0.474850323	1.75E-29	postive
FDX1	TTLL12	0.454976803	6.46E-27	postive
LIAS	TTLL12	0.507713701	4.06E-34	postive
DLD	TTLL12	0.636632845	3.53E-58	postive
DLAT	TTLL12	0.59951985	4.07E-50	postive
PDHA1	TTLL12	0.600949122	2.08E-50	postive
PDHB	TTLL12	0.581742472	1.29E-46	postive
MTF1	TTLL12	0.536132197	1.49E-38	postive
FDX1	TUBA1C	0.437446273	8.70E-25	postive
LIAS	TUBA1C	0.424606421	2.65E-23	postive
LIPT1	TUBA1C	0.430791659	5.20E-24	postive
DLD	TUBA1C	0.656585835	5.47E-63	postive
DLAT	TUBA1C	0.617208122	7.97E-54	postive
PDHA1	TUBA1C	0.556864788	4.61E-42	postive
PDHB	TUBA1C	0.572512257	7.01E-45	postive
MTF1	TUBB1	0.432538133	3.27E-24	postive
MTF1	TXLNB	0.417577039	1.61E-22	postive
FDX1	TXN	0.454436091	7.55E-27	postive
LIAS	TXN	0.511694702	1.03E-34	postive
LIPT1	TXN	0.438530362	6.48E-25	postive
DLD	TXN	0.557421538	3.68E-42	postive
DLAT	TXN	0.417949423	1.47E-22	postive
PDHA1	TXN	0.526283752	5.74E-37	postive
PDHB	TXN	0.576032749	1.55E-45	postive
FDX1	TXNDC11	0.49113435	1.02E-31	postive
LIPT1	TXNDC11	0.516111222	2.20E-35	postive
DLAT	TXNDC11	0.441899363	2.57E-25	postive
PDHA1	TXNDC11	0.536847565	1.14E-38	postive
PDHB	TXNDC11	0.548103896	1.50E-40	postive
MTF1	TXNDC11	0.444832662	1.14E-25	postive
FDX1	TXNDC15	0.50246727	2.41E-33	postive
LIPT1	TXNDC15	0.572588917	6.78E-45	postive
DLAT	TXNDC15	0.443449412	1.68E-25	postive
PDHA1	TXNDC15	0.414153257	3.83E-22	postive
PDHB	TXNDC15	0.595639185	2.47E-49	postive
DLD	TYMS	0.538604706	5.87E-39	postive
DLAT	TYMS	0.518577834	9.18E-36	postive

PDHA1	TYMS	0.481020445	2.57E-30	postive
PDHB	TYMS	0.413485038	4.53E-22	postive
LIAS	U91328.1	0.469392884	9.21E-29	postive
LIPT1	U91328.1	0.540555054	2.79E-39	postive
DLD	U91328.1	0.46702138	1.88E-28	postive
DLAT	U91328.1	0.44225824	2.33E-25	postive
PDHA1	U91328.1	0.516782194	1.73E-35	postive
PDHB	U91328.1	0.510411481	1.60E-34	postive
MTF1	U91328.1	0.422697805	4.34E-23	postive
MTF1	UBE2FP3	0.454480267	7.45E-27	postive
FDX1	UBE2MP1	0.481124248	2.49E-30	postive
LIAS	UBE2MP1	0.415160954	2.98E-22	postive
LIPT1	UBE2MP1	0.447912047	4.82E-26	postive
DLD	UBE2MP1	0.560334699	1.13E-42	postive
DLAT	UBE2MP1	0.561122568	8.16E-43	postive
PDHA1	UBE2MP1	0.55420827	1.34E-41	postive
PDHB	UBE2MP1	0.58642347	1.62E-47	postive
MTF1	UBE2MP1	0.444673361	1.19E-25	postive
DLD	UBE2S	0.461634886	9.30E-28	postive
DLAT	UBE2S	0.412359324	6.01E-22	postive
PDHA1	UBE2S	0.519706117	6.14E-36	postive
DLD	UBE2SP1	0.419168689	1.08E-22	postive
PDHA1	UBE2SP1	0.402732146	6.41E-21	postive
LIAS	UBXN11	0.435382086	1.52E-24	postive
LIPT1	UBXN11	0.485434754	6.37E-31	postive
PDHA1	UBXN11	0.494319284	3.61E-32	postive
MTF1	UBXN11	0.458648432	2.23E-27	postive
DLD	UCK2	0.50691707	5.33E-34	postive
DLAT	UCK2	0.453139326	1.09E-26	postive
PDHA1	UCK2	0.455415916	5.69E-27	postive
FDX1	UGDH-AS1	0.412314742	6.08E-22	postive
LIAS	UGDH-AS1	0.609324869	3.83E-52	postive
LIPT1	UGDH-AS1	0.573796815	4.05E-45	postive
DLAT	UGDH-AS1	0.453888678	8.83E-27	postive
PDHA1	UGDH-AS1	0.441412254	2.94E-25	postive
PDHB	UGDH-AS1	0.467973974	1.41E-28	postive
MTF1	UGDH-AS1	0.430422919	5.74E-24	postive
PDHA1	UNC13B	0.441271853	3.06E-25	postive
PDHB	UNC13B	0.487338838	3.47E-31	postive
MTF1	UNC13B	0.409521332	1.22E-21	postive
FDX1	UPRT	0.49279662	5.95E-32	postive
LIAS	UPRT	0.508575927	3.02E-34	postive
LIPT1	UPRT	0.522449459	2.30E-36	postive
DLD	UPRT	0.519468322	6.69E-36	postive
DLAT	UPRT	0.502912085	2.08E-33	postive
PDHA1	UPRT	0.569890345	2.13E-44	postive
PDHB	UPRT	0.563505512	3.06E-43	postive
MTF1	UPRT	0.553624449	1.69E-41	postive

LIPT1	VAMP1	0.428295659	1.01E-23	postive
PDHA1	VAMP1	0.40898423	1.39E-21	postive
MTF1	VAMP1	0.471589618	4.73E-29	postive
FDX1	VAMP2	0.422104272	5.06E-23	postive
LIAS	VAMP2	0.447737472	5.07E-26	postive
LIPT1	VAMP2	0.455933336	4.90E-27	postive
DLD	VAMP2	0.418813882	1.18E-22	postive
DLAT	VAMP2	0.421889849	5.35E-23	postive
PDHA1	VAMP2	0.522560637	2.21E-36	postive
PDHB	VAMP2	0.564317729	2.19E-43	postive
MTF1	VAMP2	0.54546941	4.20E-40	postive
FDX1	VAMP8	0.514089487	4.47E-35	postive
LIAS	VAMP8	0.499482226	6.56E-33	postive
LIPT1	VAMP8	0.545499889	4.15E-40	postive
PDHA1	VAMP8	0.439150855	5.47E-25	postive
PDHB	VAMP8	0.59684755	1.41E-49	postive
FDX1	VANGL1	0.424106826	3.01E-23	postive
LIPT1	VANGL1	0.410105048	1.05E-21	postive
DLD	VANGL1	0.601488247	1.62E-50	postive
DLAT	VANGL1	0.642099544	1.84E-59	postive
PDHA1	VANGL1	0.520624987	4.43E-36	postive
PDHB	VANGL1	0.438730675	6.14E-25	postive
MTF1	VANGL1	0.718969827	1.04E-80	postive
FDX1	VDAC1	0.525974104	6.42E-37	postive
LIAS	VDAC1	0.501570989	3.26E-33	postive
LIPT1	VDAC1	0.410097113	1.06E-21	postive
DLD	VDAC1	0.638358252	1.40E-58	postive
DLAT	VDAC1	0.63480316	9.36E-58	postive
PDHA1	VDAC1	0.654217586	2.13E-62	postive
PDHB	VDAC1	0.647459115	9.61E-61	postive
MTF1	VDAC1	0.496895304	1.55E-32	postive
DLD	VDAC1P2	0.487855756	2.94E-31	postive
DLAT	VDAC1P2	0.501191616	3.70E-33	postive
PDHA1	VDAC1P2	0.40115718	9.36E-21	postive
PDHB	VDAC1P2	0.457295891	3.31E-27	postive
MTF1	VDAC1P2	0.41289142	5.26E-22	postive
LIAS	VMAC	0.414706745	3.34E-22	postive
LIPT1	VMAC	0.552169581	3.02E-41	postive
PDHA1	VMAC	0.486852501	4.05E-31	postive
PDHB	VMAC	0.483843303	1.06E-30	postive
MTF1	VMAC	0.52089941	4.01E-36	postive
MTF1	VN1R83P	0.458653961	2.23E-27	postive
GLS	VXN	0.424334384	2.84E-23	postive
MTF1	WASF3	0.406374459	2.64E-21	postive
LIAS	WASH4P	0.475846431	1.29E-29	postive
LIPT1	WASH4P	0.497251039	1.38E-32	postive
PDHA1	WASH4P	0.642507011	1.48E-59	postive
PDHB	WASH4P	0.54529591	4.50E-40	postive

FDX1	WBP1	0.468752141	1.12E-28	postive
LIAS	WBP1	0.569269197	2.77E-44	postive
LIPT1	WBP1	0.647716943	8.32E-61	postive
DLD	WBP1	0.409671952	1.17E-21	postive
DLAT	WBP1	0.424197491	2.94E-23	postive
PDHA1	WBP1	0.566522536	8.75E-44	postive
PDHB	WBP1	0.573613442	4.38E-45	postive
MTF1	WBP1	0.469881521	7.95E-29	postive
LIPT1	WBP2NL	0.431890062	3.88E-24	postive
MTF1	WDFY4	0.438646004	6.28E-25	postive
DLD	WDHD1	0.55156189	3.85E-41	postive
DLAT	WDHD1	0.548723332	1.18E-40	postive
PDHA1	WDHD1	0.422523638	4.54E-23	postive
MTF1	WDHD1	0.465011128	3.43E-28	postive
LIAS	WDR19	0.557526554	3.53E-42	postive
LIPT1	WDR19	0.507678719	4.11E-34	postive
DLD	WDR19	0.422178241	4.97E-23	postive
DLAT	WDR19	0.443654189	1.58E-25	postive
PDHA1	WDR19	0.552099739	3.11E-41	postive
PDHB	WDR19	0.486840784	4.07E-31	postive
MTF1	WDR19	0.645398766	3.01E-60	postive
FDX1	WDR37	0.411392037	7.65E-22	postive
LIAS	WDR37	0.45239068	1.36E-26	postive
LIPT1	WDR37	0.491783978	8.27E-32	postive
DLD	WDR37	0.502575142	2.33E-33	postive
DLAT	WDR37	0.492777775	5.98E-32	postive
PDHA1	WDR37	0.535586636	1.84E-38	postive
PDHB	WDR37	0.500833204	4.18E-33	postive
MTF1	WDR37	0.745899636	6.15E-90	postive
FDX1	WDR4	0.43803219	7.42E-25	postive
LIAS	WDR4	0.538817148	5.41E-39	postive
LIPT1	WDR4	0.49019383	1.38E-31	postive
DLD	WDR4	0.580141187	2.61E-46	postive
DLAT	WDR4	0.573960566	3.78E-45	postive
PDHA1	WDR4	0.620540448	1.50E-54	postive
PDHB	WDR4	0.535682336	1.77E-38	postive
MTF1	WDR4	0.535890254	1.64E-38	postive
FDX1	WDR47	0.430263682	5.98E-24	postive
LIPT1	WDR47	0.506286344	6.61E-34	postive
DLD	WDR47	0.54149726	1.95E-39	postive
DLAT	WDR47	0.610404288	2.27E-52	postive
PDHA1	WDR47	0.4638628	4.82E-28	postive
PDHB	WDR47	0.492639192	6.26E-32	postive
MTF1	WDR47	0.759273994	5.75E-95	postive
GLS	WDR47	0.419200009	1.07E-22	postive
LIPT1	WDR91	0.456541171	4.11E-27	postive
PDHA1	WDR91	0.453517703	9.82E-27	postive
MTF1	WDR91	0.517248996	1.47E-35	postive

MTF1	WHAMMP3	0.426947768	1.44E-23	postive
MTF1	XCR1	0.421241599	6.32E-23	postive
DLD	XRCC2	0.522355587	2.38E-36	postive
DLAT	XRCC2	0.467461016	1.65E-28	postive
PDHA1	XRCC2	0.425733985	1.97E-23	postive
FDX1	YPEL3	0.40478357	3.89E-21	postive
LIPT1	YPEL3	0.4398663	4.50E-25	postive
PDHA1	YPEL3	0.501240691	3.64E-33	postive
PDHB	YPEL3	0.498238027	9.92E-33	postive
FDX1	YWHAG	0.427511882	1.24E-23	postive
LIAS	YWHAG	0.451063636	1.98E-26	postive
DLD	YWHAG	0.7572889	3.36E-94	postive
DLAT	YWHAG	0.683512026	4.31E-70	postive
PDHA1	YWHAG	0.581966167	1.17E-46	postive
PDHB	YWHAG	0.550380008	6.14E-41	postive
MTF1	YWHAG	0.642529099	1.46E-59	postive
FDX1	YWHAZ	0.428367077	9.88E-24	postive
LIAS	YWHAZ	0.443988463	1.44E-25	postive
LIPT1	YWHAZ	0.424329204	2.84E-23	postive
DLD	YWHAZ	0.604625438	3.66E-51	postive
DLAT	YWHAZ	0.657098276	4.07E-63	postive
PDHA1	YWHAZ	0.535968418	1.59E-38	postive
PDHB	YWHAZ	0.590927994	2.13E-48	postive
MTF1	YWHAZ	0.456279614	4.44E-27	postive
FDX1	Z83851.4	0.442778359	2.02E-25	postive
LIAS	Z83851.4	0.501525935	3.31E-33	postive
LIPT1	Z83851.4	0.488021259	2.79E-31	postive
DLD	Z83851.4	0.486567969	4.44E-31	postive
DLAT	Z83851.4	0.465033403	3.40E-28	postive
PDHA1	Z83851.4	0.525001854	9.15E-37	postive
PDHB	Z83851.4	0.475962868	1.24E-29	postive
MTF1	Z83851.4	0.402632643	6.56E-21	postive
LIPT1	Z93930.3	0.405448092	3.31E-21	postive
LIPT1	Z97989.1	0.419741318	9.29E-23	postive
MTF1	Z97989.1	0.412423464	5.91E-22	postive
PDHA1	ZBTB18	0.41174795	7.00E-22	postive
PDHB	ZDHHC2	0.453879213	8.86E-27	postive
FDX1	ZFAND4	0.449940995	2.72E-26	postive
LIPT1	ZFAND4	0.592092649	1.26E-48	postive
DLD	ZFAND4	0.541748048	1.77E-39	postive
DLAT	ZFAND4	0.573700974	4.22E-45	postive
PDHA1	ZFAND4	0.516754811	1.75E-35	postive
PDHB	ZFAND4	0.502225113	2.62E-33	postive
MTF1	ZFAND4	0.684773213	1.92E-70	postive
LIPT1	ZFP3	0.417031777	1.85E-22	postive
MTF1	ZFP3	0.509888218	1.92E-34	postive
LIAS	ZFP90	0.401870637	7.89E-21	postive
LIPT1	ZFP90	0.514786802	3.50E-35	postive

DLD	ZFP90	0.422095334	5.07E-23	postive
DLAT	ZFP90	0.488466861	2.42E-31	postive
PDHA1	ZFP90	0.486240372	4.93E-31	postive
PDHB	ZFP90	0.475002339	1.67E-29	postive
MTF1	ZFP90	0.664333457	5.88E-65	postive
GLS	ZFP90	0.463423988	5.49E-28	postive
FDX1	ZKSCAN4	0.478415614	5.80E-30	postive
LIAS	ZKSCAN4	0.540036318	3.40E-39	postive
LIPT1	ZKSCAN4	0.656700602	5.12E-63	postive
DLD	ZKSCAN4	0.554646851	1.12E-41	postive
DLAT	ZKSCAN4	0.576612652	1.21E-45	postive
PDHA1	ZKSCAN4	0.581178511	1.65E-46	postive
PDHB	ZKSCAN4	0.611214388	1.53E-52	postive
MTF1	ZKSCAN4	0.59873666	5.87E-50	postive
FDX1	ZMYND12	0.415205334	2.94E-22	postive
LIPT1	ZMYND12	0.437000729	9.82E-25	postive
PDHB	ZMYND12	0.430918737	5.03E-24	postive
LIPT1	ZNF100	0.436609059	1.09E-24	postive
MTF1	ZNF100	0.465219666	3.22E-28	postive
GLS	ZNF100	0.556928766	4.49E-42	postive
LIPT1	ZNF101	0.580296832	2.43E-46	postive
DLD	ZNF101	0.422608118	4.44E-23	postive
DLAT	ZNF101	0.428624628	9.23E-24	postive
PDHB	ZNF101	0.424629727	2.63E-23	postive
MTF1	ZNF101	0.515982332	2.30E-35	postive
GLS	ZNF101	0.433938567	2.24E-24	postive
LIPT1	ZNF124	0.553268332	1.95E-41	postive
DLD	ZNF124	0.425021411	2.37E-23	postive
DLAT	ZNF124	0.498041371	1.06E-32	postive
PDHB	ZNF124	0.428767609	8.89E-24	postive
MTF1	ZNF124	0.450286186	2.47E-26	postive
LIPT1	ZNF14	0.627039641	5.45E-56	postive
DLAT	ZNF14	0.407338874	2.08E-21	postive
PDHB	ZNF14	0.467227645	1.77E-28	postive
MTF1	ZNF14	0.471382445	5.04E-29	postive
GLS	ZNF14	0.413954906	4.03E-22	postive
MTF1	ZNF154	0.49888693	8.00E-33	postive
GLS	ZNF154	0.419998215	8.70E-23	postive
LIAS	ZNF182	0.469238911	9.65E-29	postive
LIPT1	ZNF182	0.551480921	3.97E-41	postive
DLD	ZNF182	0.439581683	4.86E-25	postive
DLAT	ZNF182	0.495428229	2.51E-32	postive
PDHA1	ZNF182	0.665109228	3.71E-65	postive
PDHB	ZNF182	0.497332572	1.34E-32	postive
MTF1	ZNF182	0.620860003	1.28E-54	postive
FDX1	ZNF19	0.413756792	4.24E-22	postive
LIAS	ZNF19	0.495259602	2.65E-32	postive
LIPT1	ZNF19	0.499018555	7.65E-33	postive

PDHA1	ZNF19	0.431899179	3.87E-24	postive
PDHB	ZNF19	0.501477806	3.36E-33	postive
MTF1	ZNF19	0.458275122	2.49E-27	postive
LIAS	ZNF205-AS1	0.502940887	2.06E-33	postive
LIPT1	ZNF205-AS1	0.52850417	2.55E-37	postive
PDHA1	ZNF205-AS1	0.447816722	4.95E-26	postive
PDHB	ZNF205-AS1	0.419337076	1.03E-22	postive
MTF1	ZNF205-AS1	0.428060598	1.07E-23	postive
LIPT1	ZNF211	0.44374991	1.54E-25	postive
PDHA1	ZNF211	0.422708743	4.33E-23	postive
MTF1	ZNF211	0.605614916	2.28E-51	postive
LIAS	ZNF25	0.447660666	5.18E-26	postive
LIPT1	ZNF25	0.546134419	3.25E-40	postive
DLD	ZNF25	0.418954221	1.14E-22	postive
DLAT	ZNF25	0.441226949	3.10E-25	postive
PDHB	ZNF25	0.472277494	3.84E-29	postive
MTF1	ZNF25	0.605791866	2.10E-51	postive
GLS	ZNF25	0.43547723	1.48E-24	postive
LIPT1	ZNF253	0.513523117	5.44E-35	postive
DLAT	ZNF253	0.431445252	4.37E-24	postive
PDHB	ZNF253	0.446930611	6.35E-26	postive
MTF1	ZNF253	0.470921538	5.80E-29	postive
LIPT1	ZNF266	0.564570928	1.97E-43	postive
PDHA1	ZNF266	0.422067313	5.11E-23	postive
MTF1	ZNF266	0.484874436	7.62E-31	postive
GLS	ZNF266	0.406759638	2.40E-21	postive
LIPT1	ZNF287	0.470538939	6.51E-29	postive
MTF1	ZNF287	0.434718057	1.82E-24	postive
LIAS	ZNF330	0.440729781	3.55E-25	postive
LIPT1	ZNF330	0.437767776	7.98E-25	postive
DLD	ZNF330	0.463513319	5.34E-28	postive
DLAT	ZNF330	0.497706514	1.18E-32	postive
PDHA1	ZNF330	0.564244141	2.26E-43	postive
PDHB	ZNF330	0.619119047	3.07E-54	postive
MTF1	ZNF330	0.494905138	2.98E-32	postive
LIAS	ZNF396	0.481895823	1.95E-30	postive
LIPT1	ZNF396	0.547536277	1.88E-40	postive
DLAT	ZNF396	0.402077189	7.50E-21	postive
PDHB	ZNF396	0.533292824	4.33E-38	postive
MTF1	ZNF396	0.456710597	3.92E-27	postive
LIPT1	ZNF429	0.483322891	1.25E-30	postive
MTF1	ZNF429	0.44425197	1.34E-25	postive
LIPT1	ZNF433-AS1	0.424856915	2.48E-23	postive
LIPT1	ZNF439	0.403921515	4.80E-21	postive
MTF1	ZNF439	0.434467621	1.95E-24	postive
GLS	ZNF439	0.4033795	5.48E-21	postive
LIAS	ZNF44	0.438419263	6.68E-25	postive
LIPT1	ZNF44	0.568301895	4.15E-44	postive

PDHA1	ZNF44	0.461373509	1.00E-27	postive
PDHB	ZNF44	0.43238305	3.40E-24	postive
MTF1	ZNF44	0.514599063	3.74E-35	postive
LIPT1	ZNF442	0.444613228	1.21E-25	postive
MTF1	ZNF442	0.426825474	1.48E-23	postive
GLS	ZNF442	0.416920534	1.91E-22	postive
MTF1	ZNF483	0.436521644	1.12E-24	postive
LIPT1	ZNF490	0.437846489	7.81E-25	postive
DLD	ZNF490	0.422799141	4.23E-23	postive
DLAT	ZNF490	0.520847653	4.09E-36	postive
MTF1	ZNF490	0.723177937	4.44E-82	postive
GLS	ZNF490	0.423020501	3.99E-23	postive
MTF1	ZNF493	0.441618045	2.78E-25	postive
GLS	ZNF493	0.489657649	1.65E-31	postive
LIPT1	ZNF501	0.480467853	3.06E-30	postive
DLAT	ZNF501	0.422048189	5.13E-23	postive
MTF1	ZNF501	0.459392795	1.80E-27	postive
GLS	ZNF501	0.415463586	2.76E-22	postive
LIPT1	ZNF506	0.436182949	1.23E-24	postive
MTF1	ZNF506	0.456515299	4.14E-27	postive
GLS	ZNF506	0.402735614	6.40E-21	postive
MTF1	ZNF516	0.445468031	9.57E-26	postive
LIPT1	ZNF540	0.40407817	4.62E-21	postive
FDX1	ZNF552	0.400746205	1.03E-20	postive
LIPT1	ZNF552	0.559460055	1.61E-42	postive
MTF1	ZNF552	0.455399672	5.72E-27	postive
LIAS	ZNF555	0.401579663	8.46E-21	postive
LIPT1	ZNF555	0.560967192	8.69E-43	postive
DLAT	ZNF555	0.511646925	1.05E-34	postive
PDHB	ZNF555	0.518551842	9.26E-36	postive
MTF1	ZNF555	0.559654184	1.49E-42	postive
GLS	ZNF555	0.445094471	1.06E-25	postive
LIAS	ZNF56	0.442717279	2.05E-25	postive
LIPT1	ZNF56	0.550823011	5.15E-41	postive
PDHB	ZNF56	0.432016913	3.75E-24	postive
MTF1	ZNF56	0.426093869	1.79E-23	postive
LIAS	ZNF563	0.445293134	1.00E-25	postive
LIPT1	ZNF563	0.523616788	1.51E-36	postive
GLS	ZNF563	0.453464726	9.98E-27	postive
FDX1	ZNF564	0.422931628	4.09E-23	postive
LIAS	ZNF564	0.412023201	6.54E-22	postive
LIPT1	ZNF564	0.569559405	2.45E-44	postive
DLD	ZNF564	0.432951818	2.92E-24	postive
DLAT	ZNF564	0.471864046	4.36E-29	postive
PDHB	ZNF564	0.451835085	1.59E-26	postive
MTF1	ZNF564	0.568402455	3.98E-44	postive
LIPT1	ZNF572	0.403126386	5.82E-21	postive
PDHA1	ZNF572	0.409340956	1.27E-21	postive

LIPT1	ZNF582-AS1	0.439286554	5.27E-25	postive
LIAS	ZNF589	0.401416352	8.80E-21	postive
LIPT1	ZNF589	0.440080058	4.24E-25	postive
PDHA1	ZNF589	0.499835025	5.83E-33	postive
PDHB	ZNF589	0.410342199	9.93E-22	postive
MTF1	ZNF589	0.590525999	2.56E-48	postive
LIPT1	ZNF630	0.498131701	1.03E-32	postive
PDHA1	ZNF630	0.424304545	2.86E-23	postive
PDHB	ZNF630	0.44952229	3.06E-26	postive
MTF1	ZNF660	0.437833556	7.83E-25	postive
LIPT1	ZNF671	0.405742773	3.08E-21	postive
MTF1	ZNF671	0.453383357	1.02E-26	postive
GLS	ZNF671	0.425209377	2.26E-23	postive
LIPT1	ZNF674-AS1	0.537426244	9.17E-39	postive
DLAT	ZNF674-AS1	0.418098353	1.41E-22	postive
PDHA1	ZNF674-AS1	0.601746194	1.43E-50	postive
PDHB	ZNF674-AS1	0.437900794	7.69E-25	postive
MTF1	ZNF674-AS1	0.403235371	5.67E-21	postive
FDX1	ZNF684	0.485137952	7.01E-31	postive
LIAS	ZNF684	0.515537871	2.69E-35	postive
LIPT1	ZNF684	0.668065891	6.31E-66	postive
DLD	ZNF684	0.417417849	1.68E-22	postive
DLAT	ZNF684	0.460479965	1.31E-27	postive
PDHA1	ZNF684	0.467074025	1.85E-28	postive
PDHB	ZNF684	0.527403223	3.81E-37	postive
MTF1	ZNF684	0.470445091	6.70E-29	postive
DLD	ZNF697	0.520382988	4.83E-36	postive
DLAT	ZNF697	0.555700883	7.36E-42	postive
PDHA1	ZNF697	0.463161841	5.93E-28	postive
MTF1	ZNF697	0.607462782	9.41E-52	postive
MTF1	ZNF704	0.461323812	1.02E-27	postive
LIPT1	ZNF708	0.525724013	7.04E-37	postive
MTF1	ZNF708	0.498151514	1.02E-32	postive
GLS	ZNF708	0.47189281	4.32E-29	postive
LIPT1	ZNF709	0.447527921	5.37E-26	postive
PDHA1	ZNF710-AS1	0.460823276	1.18E-27	postive
MTF1	ZNF710-AS1	0.527561149	3.60E-37	postive
GLS	ZNF737	0.43713726	9.47E-25	postive
LIPT1	ZNF77	0.605268768	2.69E-51	postive
DLD	ZNF77	0.402313675	7.09E-21	postive
DLAT	ZNF77	0.483290239	1.26E-30	postive
PDHA1	ZNF77	0.459386817	1.80E-27	postive
PDHB	ZNF77	0.522388611	2.35E-36	postive
MTF1	ZNF77	0.465730975	2.76E-28	postive
LIPT1	ZNF780B	0.43446514	1.95E-24	postive
MTF1	ZNF780B	0.605642424	2.25E-51	postive
LIPT1	ZNF785	0.46569316	2.80E-28	postive
PDHA1	ZNF785	0.51135068	1.16E-34	postive

PDHB	ZNF785	0.431810539	3.97E-24	postive
MTF1	ZNF785	0.545193489	4.68E-40	postive
LIAS	ZNF821	0.405991698	2.90E-21	postive
LIPT1	ZNF821	0.506187406	6.84E-34	postive
DLD	ZNF821	0.436410492	1.15E-24	postive
DLAT	ZNF821	0.499033458	7.62E-33	postive
PDHA1	ZNF821	0.581043916	1.75E-46	postive
PDHB	ZNF821	0.548943858	1.08E-40	postive
MTF1	ZNF821	0.537973288	7.46E-39	postive
LIPT1	ZNF844	0.463055713	6.12E-28	postive
MTF1	ZNF844	0.446284127	7.62E-26	postive
LIAS	ZNF846	0.406805658	2.38E-21	postive
LIPT1	ZNF846	0.525952964	6.47E-37	postive
MTF1	ZNF846	0.408191041	1.69E-21	postive
GLS	ZNF846	0.411308323	7.81E-22	postive
LIPT1	ZNF852	0.501927013	2.89E-33	postive
DLAT	ZNF852	0.460753908	1.21E-27	postive
PDHA1	ZNF852	0.409103067	1.35E-21	postive
PDHB	ZNF852	0.489623729	1.66E-31	postive
MTF1	ZNF852	0.619912236	2.06E-54	postive
LIPT1	ZNF879	0.455746454	5.18E-27	postive
LIAS	ZRANB2-AS2	0.435985273	1.29E-24	postive
LIPT1	ZRANB2-AS2	0.445839575	8.62E-26	postive
FDX1	ZSCAN16-AS1	0.473580318	2.58E-29	postive
LIAS	ZSCAN16-AS1	0.5114007	1.14E-34	postive
LIPT1	ZSCAN16-AS1	0.556230143	5.95E-42	postive
PDHA1	ZSCAN16-AS1	0.47459258	1.89E-29	postive
PDHB	ZSCAN16-AS1	0.566726073	8.04E-44	postive
FDX1	ZSCAN26	0.452321614	1.38E-26	postive
LIAS	ZSCAN26	0.465801918	2.71E-28	postive
LIPT1	ZSCAN26	0.662119044	2.18E-64	postive
DLD	ZSCAN26	0.47244176	3.65E-29	postive
DLAT	ZSCAN26	0.493850688	4.21E-32	postive
PDHA1	ZSCAN26	0.535289921	2.05E-38	postive
PDHB	ZSCAN26	0.58551451	2.43E-47	postive
MTF1	ZSCAN26	0.528504641	2.55E-37	postive
FDX1	ZSCAN32	0.463549464	5.29E-28	postive
LIAS	ZSCAN32	0.492342261	6.90E-32	postive
LIPT1	ZSCAN32	0.613223155	5.72E-53	postive
DLD	ZSCAN32	0.566261695	9.75E-44	postive
DLAT	ZSCAN32	0.615643214	1.73E-53	postive
PDHA1	ZSCAN32	0.510597473	1.50E-34	postive
PDHB	ZSCAN32	0.560485741	1.06E-42	postive
MTF1	ZSCAN32	0.707801376	3.41E-77	postive
MTF1	ZSWIM5	0.463947872	4.70E-28	postive
FDX1	ZWILCH	0.459483262	1.75E-27	postive
LIAS	ZWILCH	0.450225294	2.51E-26	postive
LIPT1	ZWILCH	0.413345088	4.70E-22	postive

DLD	ZWILCH	0.686324824	7.04E-71	postive
DLAT	ZWILCH	0.676893002	2.82E-68	postive
PDHA1	ZWILCH	0.528976271	2.14E-37	postive
PDHB	ZWILCH	0.507737789	4.03E-34	postive
MTF1	ZWILCH	0.499099544	7.45E-33	postive

Table S3. 344 CRG cluster-related differentially expressed genes identified.

ID
ABCA8
AC004233.2
AC004947.1
AC005077.4
AC005332.4
AC005884.1
AC006449.2
AC008763.1
AC009570.1
AC010615.1
AC010654.1
AC010980.2
AC010998.1
AC010998.3
AC011899.1
AC012615.1
AC012645.3
AC015802.5
AC017100.1
AC018845.3
AC021755.2
AC022144.1
AC023509.2
AC024075.1
AC037441.1
AC068228.2
AC068473.5
AC091849.2
AC092071.1
AC092171.3
AC092171.5
AC092279.1
AC103974.1
AC104564.3
AC104581.1
AC105942.1
AC110285.1
AC111170.4
AC112777.1
AC124248.1

AC138969.1
AC244090.1
ACAD8
ADAM12
ADAMTS4
ADAMTS7P3
ADAMTS8
ADAMTS9-AS2
ADH1A
ADM
ADPGK-AS1
AFF3
AL008627.1
AL021707.4
AL035587.1
AL078645.1
AL109955.1
AL122010.1
AL136304.1
AL162511.1
AL354714.2
AL355075.2
AL357093.2
AL390195.2
AL391807.1
AL445493.3
AL450996.1
AL590226.2
AL662899.4
ANKRD29
ANKRD65
AP001029.1
AP001453.2
AP003385.4
AQP4
ARHGEF26-AS1
ART4
ASAP3
ASB9P1
ASPM
ATP5MC1P4
ATP8B2
ATXN1-AS1

BTN2A2
BTN2A3P
BTNL9
C1orf210
C1QTNF6
C1QTNF7
C20orf96
CACNA2D2
CADM3-AS1
CASP10
CATSPERG
CCDC154
CCDC36
CCDC88C
CCL20
CCT6A
CD109
CDCA8
CENPE
CENPF
CENPH
CENPM
CERS4
CHAF1B
CHEK1
CHIA
CHIAP2
CHKA
chr22-38_28785274-29006793.1
CHRDL1
CLEC18A
CLEC7A
CMTM7
CSF2RB
CST5
CYB5RL
CYP2B7P
CYP4A22
CYP4B1
CYP4Z1
CYP4Z2P
CYSLTR2
DBP

DHDH
DIAPH3
DNAJC28
DRP2
E2F7
ECRG4
ECT2
EDN3
EPB41
ERO1A
ESPL1
ETV5
F12
FAM131C
FAM72A
FAM76A
FAM83A
FAM83A-AS1
FANCI
FBXO44
FBXO45
FCHSD2
FKBP4
FRG1HP
FSCN1
GADD45G
GALNT11
GAPDH
GAPDHP60
GAPDHP65
GDF10
GFRA1
GJB2
GLB1L2
GNPNAT1
GPR37
GPR75
GPRIN1
GPRIN2
GREB1L
GRIA1
GS1-124K5.3
GTF2IP7

GTSE1
GUSBP11
HLF
HMGA1
HPCAL4
HPSE2
HROB
HSD17B13
HSD17B6
HSF2BP
ICAM3
IKZF4
IL11
INCA1
INPP5J
IPO5P1
KCNMA1
KCP
KIF14
KIF18A
KIF18B
KIF20B
KLHDC8B
KNL1
KREMEN2
KRT8
KSR2
L3MBTL2-AS1
LDHA
LDLRAD4-AS1
LHFPL3
LHFPL3-AS2
LHFPL5
LIFR-AS1
LINC00261
LINC00460
LINC00528
LINC00539
LINC00578
LINC00865
LINC01132
LINC01819
LINC02535

LINC02657
LINC02728
LMNB2
LOXL2
LPCAT1
LRFN4
LRRK2-DT
MAP3K3
MCM4
MCOLN2
MDFI
MELTF
MIR34AHG
MIR4697HG
MIR646HG
MKI67
MLLT6
MMP2-AS1
MOAP1
MTMR8
MTMR9LP
MUSK
MYLIP
MYO6
NAIP
NBPF1
NCAPG2
NDUFA6-DT
NUPR1
OGT
ORMDL3
PAIP2B
PCDH15
PCDH20
PDIK1L
PDK2
PEBP4
PFKP
PGM5P4
PGPEP1
PHF1
PKIB
PKMYT1

PLCG1-AS1
PLEKHD1
POTEF
PPM1M
PPOX
PPP1R13B
PPP1R14B-AS1
PPP1R3E
PPP1R3G
PRDM16-DT
PRDM2
PRKCD
PRRT3
PSMD10P2
RACGAP1
RFXAP
RIC3
RIMKLA
ROM1
RP11-22P4.1
RP11-876N24.5
RPL13AP17
RPL18AP7
RPS6KL1
RUBCNL
SCAT1
SCN7A
SDK1
SFTPB
SFTPD
SGO2
SHCBP1
SLC16A3
SLC22A23
SLC25A42
SLC25A5-AS1
SLC35E2B
SLC4A8
SMAP2
SMCO2
SMIM17
SNAI3
SORT1

SP6
SPAG5
SPDL1
SPHK1
ST3GAL6-AS1
STKLD1
STPG3
SUSD2
TACC3
TAS1R1
TCEANC
TCF21
TCTA
TEDC2
TFAP2A
TICRR
TLE2
TMEM132D
TMEM150A
TMEM170B
TMEM8B
TNK2-AS1
TNRC18P1
TOP2A
TRAV21
TRDMT1
TYMS
VAMP1
VEGFD
VSIG2
WDFY4
WDHD1
WDR86-AS1
WDR91
WSB1
ZFAND4
ZNF253
ZNF266
ZNF43
ZNF502
ZNF708
ZNF710-AS1
ZNF727

ZNF737
ZNF90
ZSWIM5

Table S4. KEGG pathways identified by GSVA between two CRG-DEG clusters.

id	logFC	AveExpr	t	P.Value	adj.P.Val	B
KEGG_ALPHA_LINOLENIC_ACID_METABOLISM	-0.283047	-0.013956	-13.00883	1.24E-33	2.31E-31	65.58343
KEGG_LINOLEIC_ACID_METABOLISM	-0.278035	-0.020138	-12.17508	3.96E-30	3.68E-28	57.62793
KEGG_ARACHIDONIC_ACID_METABOLISM	-0.234427	-0.013796	-11.91226	4.74E-29	2.94E-27	55.17919
KEGG_DNA_REPLICATION	0.429175	-0.022075	10.96843	2.74E-25	1.28E-23	46.64225
KEGG_TAURINE_AND_HYPOTAURINE_METABOLISM	-0.272619	-0.019764	-10.71142	2.69E-24	1.00E-22	44.39181
KEGG_CELL_CYCLE	0.333537	-0.018108	10.51275	1.54E-23	4.46E-22	42.67543
KEGG_ABC_TRANSPORTERS	-0.222878	-0.017364	-10.50287	1.68E-23	4.46E-22	42.59054
KEGG_PRIMARY_BILE_ACID_BIOSYNTHESIS	-0.244094	-0.022434	-9.765476	8.99E-21	2.09E-19	36.40865
KEGG_FATTY_ACID_METABOLISM	-0.264414	-0.022206	-9.618667	3.03E-20	6.26E-19	35.21421
KEGG_ETHER_LIPID_METABOLISM	-0.200185	-0.019731	-9.36358	2.43E-19	4.52E-18	33.16876
KEGG_HOMOLOGOUS_RECOMBINATION	0.337241	-0.025433	9.333735	3.09E-19	5.23E-18	32.93196
KEGG_PROXIMAL_TUBULE_BICARBONATE_RECLAMATION	-0.193345	-0.027752	-8.807536	1.98E-17	3.07E-16	28.84642
KEGG_VASCULAR_SMOOTH_MUSCLE_CONTRACTION	-0.201031	-0.014647	-8.700509	4.52E-17	6.47E-16	28.03662
KEGG_PPAR_SIGNALING_PATHWAY	-0.165719	-0.022642	-8.58028	1.13E-16	1.51E-15	27.13568
KEGG_ALDOSTERONE_REGULATED_SODIUM_REABSORPTION	-0.185492	-0.015956	-8.18893	2.11E-15	2.62E-14	24.26862
KEGG_MISMATCH_REPAIR	0.316062	-0.022138	8.171889	2.39E-15	2.70E-14	24.14609
KEGG_NITROGEN_METABOLISM	-0.167484	-0.035188	-8.167609	2.47E-15	2.70E-14	24.11535
KEGG_HISTIDINE_METABOLISM	-0.184021	-0.028017	-7.992691	8.81E-15	9.11E-14	22.86955
KEGG_GNRH_SIGNALING_PATHWAY	-0.173707	-0.01471	-7.870401	2.12E-14	2.07E-13	22.01103
KEGG_BASAL_CELL_CARCINOMA	-0.171307	-0.012722	-7.422872	4.81E-13	4.47E-12	18.95854
KEGG_ASTHMA	-0.280182	0.010845	-7.406102	5.39E-13	4.77E-12	18.84693
KEGG_FC_EPSILON_RI_SIGNALING_PATHWAY	-0.178843	-0.012191	-7.280623	1.26E-12	1.06E-11	18.01831
KEGG_GLYCEROPHOSPHOLIPID_METABOLISM	-0.15748	-0.018392	-7.214473	1.96E-12	1.59E-11	17.58609
KEGG_CALCIUM_SIGNALING_PATHWAY	-0.143602	-0.014154	-7.137318	3.27E-12	2.54E-11	17.08604
KEGG_HEDGEHOG_SIGNALING_PATHWAY	-0.159721	-0.015526	-7.124297	3.57E-12	2.65E-11	17.00208
KEGG_PROTEASOME	0.275482	-0.021759	7.069005	5.13E-12	3.67E-11	16.64696

KEGG_LONG_TERM_DEPRESSION	-0.144492	-0.019707	-7.043336	6.07E-12	4.18E-11	16.48287
KEGG_GLYCOSPHINGOLIPID_BIOSYNTHESIS_GANGLIO_SERIES	-0.193014	-0.020059	-6.457234	2.48E-10	1.65E-09	12.87175
KEGG_COMPLEMENT_AND_COAGULATION_CASCADES	-0.154628	-0.006965	-6.031188	3.12E-09	2.00E-08	10.41423
KEGG_VALINE_LEUCINE_AND_ISOLEUCINE_DEGRADATION	-0.194939	-0.015296	-6.022548	3.28E-09	2.03E-08	10.36589
KEGG_TYROSINE_METABOLISM	-0.130793	-0.025545	-5.987682	4.01E-09	2.40E-08	10.17142
KEGG_DRUG_METABOLISM_CYTOCHROME_P450	-0.151027	-0.026743	-5.972484	4.37E-09	2.54E-08	10.08696
KEGG_TRYPTOPHAN_METABOLISM	-0.125421	-0.024739	-5.687572	2.17E-08	1.22E-07	8.538315
KEGG_RENIN_ANGIOTENSIN_SYSTEM	-0.15427	-0.017151	-5.446726	7.98E-08	4.36E-07	7.281267
KEGG_CELL_ADHESION_MOLECULES_CAMS	-0.160366	3.14E-05	-5.435058	8.49E-08	4.51E-07	7.221595
KEGG_LONG_TERM_POTENTIATION	-0.124803	-0.021488	-5.299893	1.72E-07	8.84E-07	6.538649
KEGG_DILATED_CARDIOMYOPATHY	-0.133233	-0.01128	-5.296189	1.76E-07	8.84E-07	6.520152
KEGG_BETA_ALANINE_METABOLISM	-0.133541	-0.029212	-5.287854	1.84E-07	8.99E-07	6.478567
KEGG_OOCYTE_MEIOSIS	0.139085	-0.023443	5.223907	2.55E-07	1.22E-06	6.161478
KEGG_P53_SIGNALING_PATHWAY	0.131889	-0.022425	5.174832	3.28E-07	1.53E-06	5.920488
KEGG_PENTOSE_AND_GLUCURONATE_INTERCONVERSIONS	0.143392	-0.044244	5.110275	4.55E-07	2.06E-06	5.606591
KEGG_BASE_EXCISION_REPAIR	0.177941	-0.014459	5.100725	4.77E-07	2.11E-06	5.560457
KEGG_HEMATOPOIETIC_CELL_LINEAGE	-0.157614	0.006532	-5.003603	7.75E-07	3.35E-06	5.095711
KEGG_INTESTINAL_IMMUNE_NETWORK_FOR_IGA_PRODUCTION	-0.188341	0.005086	-4.995136	8.08E-07	3.41E-06	5.055583
KEGG_MELANOGENESIS	-0.105757	-0.014975	-4.974445	8.95E-07	3.62E-06	4.957765
KEGG_PHOSPHATIDYLINOSITOL_SIGNALING_SYSTEM	-0.133987	-0.015796	-4.974282	8.95E-07	3.62E-06	4.956997
KEGG_CIRCADIAN_RHYTHM_MAMMAL	-0.16132	-0.014208	-4.952925	9.94E-07	3.94E-06	4.856424
KEGG_SYSTEMIC_LUPUS_ERYTHEMATOSUS	0.114161	-0.020037	4.937444	1.07E-06	4.16E-06	4.783768
KEGG_BUTANOATE_METABOLISM	-0.124374	-0.027218	-4.841337	1.71E-06	6.36E-06	4.33735
KEGG_TYPE_II_DIABETES_MELLITUS	-0.103272	-0.018781	-4.827321	1.83E-06	6.67E-06	4.27291
KEGG_OLFACTORY_TRANSDUCTION	0.101564	0.09938	4.789255	2.19E-06	7.85E-06	4.098768
KEGG_ALLOGRAFT_REJECTION	-0.202178	0.008692	-4.743388	2.73E-06	9.58E-06	3.89061
KEGG_GLYOXYLATE_AND_DICARBOXYLATE_ME	0.155492	-0.024252	4.687208	3.55E-06	1.22E-05	3.638149

TABOLISM						
KEGG_VIRAL_MYOCARDITIS	-0.144868	-0.003898	-4.676038	3.75E-06	1.27E-05	3.588283
KEGG_PYRIMIDINE_METABOLISM	0.143448	-0.019189	4.647998	4.27E-06	1.42E-05	3.463585
KEGG_PENTOSE_PHOSPHATE_PATHWAY	0.13877	-0.018257	4.625615	4.74E-06	1.55E-05	3.364537
KEGG_LYSOSOME	-0.147538	-0.006039	-4.617809	4.91E-06	1.57E-05	3.330096
KEGG_ONE_CARBON_POOL_BY_FOLATE	0.143375	-0.024557	4.419279	1.21E-05	3.81E-05	2.47225
KEGG_PATHOGENIC_ESCHERICHIA_COLI_INFECTION	0.12664	-0.018415	4.39266	1.36E-05	4.15E-05	2.359879
KEGG_PROPANOATE_METABOLISM	-0.141463	-0.022072	-4.392448	1.36E-05	4.15E-05	2.358988
KEGG_PEROXISOME	-0.127235	-0.015619	-4.308214	1.97E-05	5.92E-05	2.007567
KEGG_VASOPRESSIN_REGULATED_WATER_REABSORPTION	-0.130271	-0.0193	-4.252321	2.52E-05	7.43E-05	1.777872
KEGG_GALACTOSE_METABOLISM	0.118629	-0.020419	4.237506	2.68E-05	7.79E-05	1.71746
KEGG_VEGF_SIGNALING_PATHWAY	-0.100841	-0.016058	-4.224061	2.84E-05	8.13E-05	1.662803
KEGG_SULFUR_METABOLISM	-0.117734	-0.020348	-4.202254	3.12E-05	8.54E-05	1.574494
KEGG_LIMONENE_AND_PINENE_DEGRADATION	-0.150423	-0.027811	-4.201929	3.12E-05	8.54E-05	1.573182
KEGG_HYPERTROPHIC_CARDIOMYOPATHY_HCM	-0.100616	-0.01573	-4.143251	4.01E-05	0.000106	1.337705
KEGG_AUTOIMMUNE_THYROID_DISEASE	-0.138409	0.02001	-4.068629	5.48E-05	0.000143	1.042717
KEGG_NUCLEOTIDE_EXCISION_REPAIR	0.144364	-0.014825	3.905912	0.000106	0.000271	0.416933
KEGG_GLYCOSAMINOGLYCAN_DEGRADATION	-0.119636	-0.007653	-3.898011	0.00011	0.000276	0.387159
KEGG_PANTOTHENATE_AND_COA_BIOSYNTHESIS	-0.100148	-0.023307	-3.753755	0.000194	0.000463	-0.146456
KEGG_LEISHMANIA_INFECTION	-0.123112	0.004473	-3.571527	0.000388	0.000914	-0.793281
KEGG_B_CELL_RECEPTOR_SIGNALING_PATHWAY	-0.108922	-0.011728	-3.495003	0.000515	0.001183	-1.05578
KEGG_ASCORBATE_AND_ALDARATE_METABOLISM	0.105141	-0.037442	3.459066	0.000587	0.001332	-1.177184
KEGG_BASAL_TRANSCRIPTION_FACTORS	0.11549	-0.022653	3.360637	0.000836	0.00185	-1.503569
KEGG_OTHER_GLYCAN_DEGRADATION	-0.10821	-0.011183	-3.193832	0.00149	0.003223	-2.036076
KEGG_SPLICEOSOME	0.104986	-0.005334	2.648156	0.008343	0.015213	-3.595158

Table S5. The KM and Cox analysis screened the DELs and found 17 DELs meet our criteria for further LASSO.

ID	KM_pvalue	HR	HR_95L	HR_95H	Cox_pvalue
AC009120.2	0.038352	0.566604	0.389068	0.82515	0.003056
AC009506.2	0.027304	0.487657	0.280052	0.84916	0.011156
AC069224.2	0.018572	0.661396	0.450212	0.971644	0.035157
AC093010.2	0.001083	0.495782	0.333801	0.736366	0.000509
AC107464.3	0.004118	0.526263	0.377603	0.733451	0.00015
AL353622.1	0.04654	0.599205	0.426505	0.841837	0.003152
COLCA1	0.003529	0.588493	0.466014	0.743162	8.46E-06
CYP2B7P	0.00346	0.728416	0.610201	0.869532	0.000453
LINC00324	0.00368	0.357058	0.21421	0.595165	7.80E-05
LINC00862	0.005289	1.356938	1.050535	1.752708	0.019413
LINC01354	0.001945	0.676853	0.491786	0.931564	0.016622
LINC01711	0.004163	1.470837	1.092253	1.980641	0.011049
LINC01833	0.001948	1.325895	1.132956	1.551691	0.000438
PRKAG2-AS1	0.00907	0.553704	0.388591	0.788974	0.001068
SNHG19	0.016694	0.650346	0.438354	0.964861	0.032542
TMEM252-DT	0.022229	0.535436	0.309018	0.927752	0.025924
TSPOAP1-AS1	0.033559	0.479305	0.315157	0.728947	0.000586

Table S6. Correlations between the signature and apoptosis, necroptosis, pyroptosis, and ferroptosis -related genes, respectively, that measured by the Pearson coefficient.

ID	*r*	*p-value*
**Apoptosis**		
ANLN	0.569830353	2.18E-44
SELENBP1	-0.563844601	2.66E-43
TPX2	0.546693724	2.61E-40
METTL7A	-0.540054239	3.38E-39
CDC20	0.532668023	5.47E-38
CENPA	0.522850018	1.99E-36
CAPN3	-0.522364716	2.37E-36
TUBA1C	0.521563489	3.16E-36
CEP55	0.5210149	3.85E-36
CDC6	0.519584677	6.42E-36
BIRC5	0.519348402	6.98E-36
DAPK2	-0.519256754	7.21E-36
KIF23	0.517285299	1.45E-35
CTSH	-0.513602137	5.30E-35
CTSV	0.513274032	5.94E-35
GNG7	-0.512614113	7.47E-35
PLK1	0.512336778	8.23E-35
MOAP1	-0.510387105	1.62E-34
CCNA2	0.508079693	3.58E-34
EXO1	0.507238313	4.78E-34
CCNB1	0.502723055	2.21E-33
PRC1	0.50167831	3.15E-33
CACNA2D2	-0.500603502	4.51E-33
DLGAP5	0.498574946	8.87E-33
NCAPG	0.495299472	2.62E-32
GAPDH	0.495086221	2.81E-32
BUB1	0.493873943	4.18E-32
DEPDC1	0.493037703	5.50E-32
N4BP2L2	-0.491566097	8.88E-32
KIF11	0.490508168	1.25E-31
MYBL2	0.488962681	2.06E-31
RASGRP2	-0.488546991	2.35E-31
TTK	0.48806408	2.75E-31
PPM1G	0.487307348	3.51E-31
HLA-DMA	-0.485987395	5.35E-31
CDKN3	0.485195885	6.88E-31
KIF14	0.48326208	1.27E-30

GGA2	-0.48264378	1.54E-30
CHRDL1	-0.481199525	2.43E-30
UBA7	-0.478706497	5.30E-30
MAD2L1	0.47782522	6.97E-30
BUB1B	0.476417538	1.08E-29
C7	-0.475822513	1.30E-29
CD40LG	-0.472809842	3.26E-29
SPC25	0.472651399	3.43E-29
ARHGAP11A	0.468953678	1.05E-28
TOP2A	0.468627081	1.16E-28
LRRK2	-0.466891429	1.95E-28
UBE2C	0.466523806	2.18E-28
RASSF5	-0.465899537	2.63E-28
ATP8A1	-0.465222145	3.22E-28
SPAG5	0.464148982	4.43E-28
MKI67	0.46339765	5.53E-28
YKT6	0.462684567	6.83E-28
NDC80	0.461664247	9.22E-28
PSMD12	0.460304902	1.37E-27
MELK	0.459868084	1.56E-27
AURKA	0.459623638	1.68E-27
NLRP1	-0.459218003	1.89E-27
KIFC1	0.459123548	1.94E-27
TNFSF13	-0.457874518	2.79E-27
TRIM22	-0.456883561	3.73E-27
CHEK1	0.456693033	3.94E-27
CENPF	0.454056755	8.42E-27
NUSAP1	0.45377993	9.11E-27
GTSE1	0.452988958	1.14E-26
ASPM	0.452160962	1.45E-26
PLK4	0.451924419	1.55E-26
DIAPH3	0.450705797	2.19E-26
AQP4	-0.449643909	2.96E-26
PTK2B	-0.449101738	3.45E-26
CDK1	0.448964576	3.59E-26
S1PR4	-0.448829582	3.73E-26
AGER	-0.448758687	3.80E-26
TFEB	-0.447936011	4.79E-26
RBP5	-0.447881122	4.87E-26
SFTPC	-0.447592981	5.28E-26
PXMP4	-0.44501901	1.08E-25
ZNF44	-0.444330454	1.31E-25
ABCC6	-0.443514953	1.65E-25

RACGAP1	0.443255924	1.77E-25
PSMD11	0.442626183	2.11E-25
CDC25A	0.442587217	2.13E-25
CDC25C	0.441844177	2.61E-25
CYFIP2	-0.441749633	2.68E-25
AURKB	0.441605998	2.79E-25
CD22	-0.440187487	4.12E-25
FAM13B	-0.439302878	5.25E-25
ECT2	0.43842042	6.68E-25
PLA2G1B	-0.438198844	7.09E-25
DPYSL2	-0.43795218	7.59E-25
CYB5A	-0.437028505	9.75E-25
YWHAG	0.435315895	1.55E-24
CTHRC1	0.435251506	1.58E-24
ZWINT	0.434998387	1.69E-24
KIF18A	0.434650735	1.85E-24
UBE2T	0.433078863	2.83E-24
MCM6	0.432802408	3.04E-24
INCA1	-0.43213422	3.64E-24
HLA-DOA	-0.432085205	3.69E-24
MAPK10	-0.432020222	3.75E-24
NPC2	-0.430391292	5.79E-24
PRKCB	-0.429524943	7.28E-24
CENPE	0.428970152	8.43E-24
CKAP4	0.428334778	9.97E-24
PSMD14	0.42813678	1.05E-23
RBM5	-0.425092366	2.33E-23
TWF1	0.424082034	3.03E-23
HMMR	0.423731031	3.32E-23
IRF8	-0.423556761	3.48E-23
ESPL1	0.423281288	3.73E-23
FANCI	0.42276111	4.27E-23
NUF2	0.422670651	4.37E-23
LAMP3	-0.421244611	6.32E-23
SLC27A3	-0.420480687	7.69E-23
PARP15	-0.419627761	9.56E-23
TUBA1B	0.419612539	9.60E-23
LMNB2	0.418168839	1.39E-22
ZNF354B	-0.417828596	1.51E-22
PBK	0.417562351	1.62E-22
HLA-DRB1	-0.417246194	1.76E-22
YWHAZ	0.416069666	2.37E-22
TLR2	-0.414459931	3.55E-22

MCM4	0.414308903	3.69E-22
RASSF2	-0.413495221	4.52E-22
PSMC4	0.413232037	4.83E-22
OGT	-0.413105193	4.99E-22
SARM1	-0.412667735	5.56E-22
RFTN1	-0.412637067	5.61E-22
HLA-DMB	-0.412354333	6.02E-22
CLK1	-0.412274863	6.14E-22
BLM	0.411620116	7.23E-22
CELF2	-0.411225231	7.97E-22
TNFRSF13B	-0.410430425	9.72E-22
BTK	-0.410252109	1.02E-21
TFG	0.409794343	1.14E-21
CENPU	0.409599092	1.19E-21
SYNE1	-0.409495842	1.22E-21
ITM2A	-0.409214734	1.31E-21
ARHGEF2	-0.408459615	1.58E-21
KNSTRN	0.408235431	1.67E-21
RORA	-0.408126651	1.72E-21
CARD8	-0.406485315	2.57E-21
NOD1	-0.406354495	2.65E-21
ZAP70	-0.405917352	2.95E-21
RTN4RL1	-0.40570692	3.11E-21
RAD54L	0.405672574	3.13E-21
PSMD2	0.405053545	3.65E-21
DLC1	-0.404461592	4.21E-21
LDHA	0.404420484	4.25E-21
IL6R	-0.40402569	4.68E-21
RPS6KA1	-0.403275491	5.62E-21
NDRG2	-0.402965635	6.05E-21
RAD51AP1	0.40252761	6.73E-21
SELP	-0.402477345	6.81E-21
GPN1	0.398627847	1.72E-20
GIMAP5	-0.398349797	1.83E-20
ARRB2	-0.397525277	2.23E-20
CLSPN	0.397468807	2.26E-20
SMS	0.397379393	2.31E-20
TEAD4	0.396937911	2.57E-20
PLCG2	-0.396857716	2.61E-20
HLA-DRA	-0.396583614	2.79E-20
DENND1C	-0.395994942	3.21E-20
ARHGAP44	-0.395920269	3.26E-20
DRAM1	-0.395742498	3.40E-20

CISH	-0.395666838	3.47E-20
GPC6	0.395642726	3.48E-20
SPHK1	0.395531395	3.58E-20
ZNF385B	-0.395336635	3.75E-20
NISCH	-0.395075786	3.98E-20
DAPK1	-0.394315815	4.76E-20
CASP12	-0.394110271	5.00E-20
HLA-DPA1	-0.394027323	5.10E-20
PSMD1	0.393448495	5.84E-20
PA2G4	0.392948165	6.56E-20
HDAC2	0.392750017	6.88E-20
NME1	0.392336744	7.57E-20
BLK	-0.39223695	7.75E-20
LRIG1	-0.391949677	8.29E-20
EIF2S2	0.391187393	9.90E-20
UNC13B	-0.390425973	1.18E-19
WDHD1	0.390135733	1.26E-19
MACROD2	-0.389261513	1.55E-19
PARPBP	0.388138306	2.00E-19
RAC1	0.387914529	2.11E-19
EIF4EBP3	-0.387360944	2.40E-19
LMO3	-0.387329254	2.41E-19
TYMS	0.387110196	2.54E-19
HLA-DOB	-0.386413839	2.98E-19
ST3GAL5	-0.386291953	3.06E-19
KIF15	0.386105458	3.19E-19
EIF3B	0.386067824	3.22E-19
ANGPTL4	0.385579826	3.60E-19
MFAP4	-0.385340498	3.80E-19
BRCA1	0.385066306	4.05E-19
NEAT1	-0.384740148	4.36E-19
MAP3K3	-0.384698134	4.40E-19
PDLIM2	-0.38457952	4.52E-19
ZNF700	-0.384249024	4.87E-19
SMARCA2	-0.383905761	5.27E-19
GDF10	-0.383773711	5.43E-19
CYCS	0.383752331	5.45E-19
IKZF1	-0.383457157	5.83E-19
FSCN1	0.383390709	5.92E-19
GINS1	0.383228238	6.14E-19
MAPK6	0.383057107	6.38E-19
NUDT1	0.382919353	6.59E-19
LINC00261	-0.382395983	7.41E-19

CENPC	-0.382390818	7.42E-19
DSG2	0.381596424	8.88E-19
XPC	-0.381053399	1.00E-18
RALA	0.381039096	1.01E-18
PSMB5	0.380521423	1.13E-18
FGFR2	-0.380081149	1.25E-18
SLC16A3	0.378925905	1.61E-18
CCNE1	0.378239957	1.88E-18
ITGB1	0.378235844	1.88E-18
STC1	0.377299963	2.32E-18
KRT18	0.37726199	2.34E-18
YES1	0.377223867	2.36E-18
CTSG	-0.376840778	2.56E-18
MEF2C	-0.376476495	2.78E-18
KIF20B	0.376143069	2.99E-18
SLC7A5	0.375983606	3.10E-18
UBE2K	0.37559487	3.38E-18
PPP3CC	-0.375316264	3.59E-18
MCM2	0.375244345	3.65E-18
STAP1	-0.375054973	3.80E-18
ACTG1	0.374593836	4.21E-18
SACM1L	-0.374450236	4.34E-18
PNISR	-0.374404322	4.39E-18
PPP1R13B	-0.374111176	4.68E-18
FUCA1	-0.373638863	5.19E-18
IKBKB	-0.373591395	5.24E-18
MICAL1	-0.373527356	5.32E-18
PARM1	-0.373246076	5.65E-18
FLT3	-0.373159668	5.76E-18
HLA-DRB5	-0.372780887	6.26E-18
SNRPD1	0.372544046	6.59E-18
DBF4	0.371791119	7.77E-18
PDCL3	0.371587196	8.12E-18
IRX5	-0.370989916	9.24E-18
HDGF	0.370660804	9.92E-18
VDAC1	0.37042164	1.05E-17
LRRN3	-0.370168011	1.10E-17
ALDH2	-0.369181398	1.37E-17
KPNB1	0.36915511	1.37E-17
LMNB1	0.368066994	1.74E-17
LY9	-0.367992965	1.76E-17
MDM4	-0.367241266	2.07E-17
ISCU	-0.366478587	2.44E-17

HLA-E	-0.365704904	2.88E-17
MMD	0.365664292	2.90E-17
EIF5A	0.365201878	3.20E-17
PPAT	0.3650693	3.29E-17
CDC27	0.365049455	3.31E-17
FAM114A1	0.363938838	4.19E-17
KCNJ5	-0.363649138	4.45E-17
HSPD1	0.363566987	4.53E-17
PERP	0.363178384	4.91E-17
TMEM158	0.363059156	5.04E-17
SPDL1	0.362169716	6.08E-17
PSME3	0.361742419	6.65E-17
AGPS	0.360826411	8.06E-17
PSTPIP1	-0.360137608	9.30E-17
PFDN2	0.359414453	1.08E-16
EIF5B	0.358859868	1.21E-16
TP53INP1	-0.358678264	1.26E-16
FCER2	-0.358494038	1.31E-16
RGCC	-0.358479405	1.31E-16
ANGPTL6	-0.358304408	1.36E-16
HLA-DQA1	-0.358186989	1.40E-16
NAA15	0.357982119	1.46E-16
TIMELESS	0.357511918	1.61E-16
SYNE3	-0.357214166	1.71E-16
AQP3	-0.356634658	1.92E-16
VEGFC	0.355811066	2.28E-16
ST3GAL6	-0.35579871	2.29E-16
LDLRAD4	-0.355784893	2.29E-16
E2F8	0.355593664	2.38E-16
CLU	-0.355532135	2.41E-16
PTCH1	-0.355292342	2.54E-16
WDR37	-0.354861975	2.77E-16
PTTG1	0.354724509	2.85E-16
ZBTB18	-0.35432552	3.09E-16
CCDC88B	-0.35363902	3.56E-16
AVEN	0.353273616	3.83E-16
CENPH	0.352848197	4.18E-16
MAP4K1	-0.352210547	4.76E-16
BLNK	-0.35197195	4.99E-16
IL24	-0.351916799	5.05E-16
ABCA5	-0.351882926	5.09E-16
CCL19	-0.351728855	5.25E-16
ASAH1	-0.351702133	5.28E-16

CD79B	-0.351584048	5.40E-16
DDX5	-0.351364126	5.65E-16
NKTR	-0.35090056	6.20E-16
ERGIC2	0.350766705	6.37E-16
PAN2	-0.350670239	6.50E-16
CYLD	-0.350604258	6.59E-16
RIPK2	0.350104436	7.28E-16
MIR22HG	-0.349869012	7.64E-16
FBXO25	-0.349553866	8.14E-16
VDAC2	0.349426954	8.35E-16
CDK2	0.349077904	8.96E-16
PNO1	0.349022359	9.06E-16
OPN3	0.348773778	9.52E-16
PIK3R5	-0.348764144	9.54E-16
TMSB10	0.347824587	1.15E-15
TNNT1	0.34768767	1.18E-15
OTUD7A	-0.347671142	1.19E-15
DNASE1L3	-0.347041453	1.35E-15
CD19	-0.346921314	1.38E-15
PANX1	0.346753591	1.43E-15
BDH2	-0.346624064	1.46E-15
ATAD2	0.346450641	1.51E-15
EGLN3	0.346309719	1.56E-15
SIRT3	-0.346066148	1.63E-15
PTPN13	-0.345904769	1.69E-15
DTHD1	-0.345848676	1.71E-15
HLA-DQB1	-0.345595695	1.79E-15
PIK3R1	-0.345513049	1.82E-15
CSE1L	0.345478546	1.84E-15
PARP8	-0.345218268	1.93E-15
CLEC14A	-0.344896271	2.06E-15
XRCC5	0.344117903	2.40E-15
AGFG1	0.344059574	2.43E-15
PPP2R5A	-0.344012588	2.45E-15
NRAS	0.343836219	2.54E-15
NR1H3	-0.343694067	2.61E-15
TSPYL2	-0.34312187	2.92E-15
BZW2	0.342967602	3.01E-15
TCF25	-0.342814694	3.10E-15
IL5RA	-0.34259478	3.24E-15
PLA2G6	-0.34255724	3.27E-15
NTRK3	-0.342538867	3.28E-15
PPWD1	-0.342444529	3.34E-15

SATB1	-0.342377605	3.38E-15
MTMR2	0.341927189	3.69E-15
PRKCD	-0.341801987	3.79E-15
ACSL5	-0.341715046	3.85E-15
GMFG	-0.341514445	4.01E-15
RGS13	-0.341036632	4.40E-15
PTPRC	-0.340966378	4.46E-15
OLFM1	-0.34094093	4.48E-15
CSF2RB	-0.340699063	4.70E-15
TRIB3	0.340643448	4.75E-15
RIPK3	-0.340544347	4.84E-15
DEF6	-0.340505102	4.88E-15
ZNF397	-0.340434367	4.94E-15
CAMKK1	-0.340431279	4.95E-15
DYNLL1	0.339322905	6.14E-15
SERPINB5	0.339198021	6.29E-15
UBASH3A	-0.338603576	7.05E-15
MECOM	-0.338488595	7.21E-15
EVI2B	-0.33744061	8.83E-15
PFKP	0.33712607	9.38E-15
CPEB3	-0.337052984	9.51E-15
PRAME	0.33688719	9.82E-15
RPAIN	-0.33685109	9.88E-15
IL2	-0.336802099	9.98E-15
ARHGDIB	-0.336740374	1.01E-14
SORCS2	-0.33667767	1.02E-14
P2RX1	-0.336527952	1.05E-14
FHL2	0.336230806	1.11E-14
NFATC2	-0.33588902	1.19E-14
IL11	0.335850938	1.20E-14
DPP7	-0.335473874	1.29E-14
GNRH1	-0.335244273	1.34E-14
TNFSF12	-0.334028983	1.70E-14
SMOX	0.333834576	1.76E-14
AXIN2	-0.333637545	1.83E-14
DOK4	-0.333101583	2.02E-14
DMXL1	-0.332980033	2.07E-14
PARP3	-0.332129808	2.43E-14
FNDC3B	0.332010809	2.48E-14
KDELR2	0.331844206	2.56E-14
BTN3A3	-0.331355591	2.81E-14
PFDN4	0.331205087	2.89E-14
SMAD6	-0.3306777	3.19E-14

DMTF1	-0.330655145	3.21E-14
ICAM3	-0.330400623	3.36E-14
CERKL	-0.330316108	3.42E-14
SEMA4B	0.330241492	3.47E-14
CORO1A	-0.330016383	3.61E-14
ACSM3	-0.32996235	3.65E-14
BRIX1	0.329774086	3.78E-14
MYOM2	-0.329140362	4.26E-14
FADD	0.328996143	4.37E-14
CCNL2	-0.328728566	4.60E-14
LTB	-0.32869789	4.63E-14
ABHD14A	-0.328510055	4.79E-14
POLQ	0.328474963	4.82E-14
VPS13C	-0.327742553	5.53E-14
S100B	-0.327587044	5.69E-14
MIR34C	-0.327584412	5.69E-14
SGSH	-0.327468889	5.81E-14
STIP1	0.327010552	6.33E-14
NRGN	-0.326960527	6.39E-14
YWHAQ	0.326856626	6.51E-14
PRKCQ	-0.326683309	6.72E-14
CHIA	-0.32626603	7.26E-14
LSM5	0.326093679	7.50E-14
GGH	0.325762556	7.97E-14
HOXA10	0.325378457	8.56E-14
PYHIN1	-0.3252017	8.84E-14
CD28	-0.325015458	9.15E-14
FLNC	0.324910675	9.33E-14
TUBB3	0.324837233	9.45E-14
MTUS1	-0.324529461	1.00E-13
SUMO2	0.323967432	1.11E-13
OMA1	-0.323244763	1.27E-13
LPXN	-0.323190233	1.28E-13
TRAIP	0.323119303	1.29E-13
PYCR1	0.322789496	1.38E-13
MRGBP	0.322774493	1.38E-13
SREK1	-0.322548091	1.44E-13
CD247	-0.322527114	1.44E-13
ING4	-0.322436664	1.47E-13
RNF125	-0.321806015	1.65E-13
DDX10	0.321523485	1.73E-13
MAOA	-0.321404728	1.77E-13
CD99	0.321322665	1.80E-13

PGAM1	0.319912668	2.32E-13
TNFAIP6	0.31983552	2.35E-13
REV3L	-0.319815749	2.36E-13
BCL2L12	0.31978964	2.37E-13
C4B	-0.319758578	2.38E-13
STMN1	0.319673548	2.42E-13
IFI30	-0.319627537	2.44E-13
MAP4K4	0.319281409	2.60E-13
TXNIP	-0.319094322	2.69E-13
PSMB2	0.318977661	2.74E-13
PCDH7	0.31888161	2.79E-13
GNPTAB	-0.318705051	2.88E-13
EPC1	-0.318673395	2.90E-13
IKZF3	-0.318522876	2.98E-13
ELOVL6	0.318373894	3.06E-13
TBRG1	-0.317968223	3.29E-13
NAMPT	0.317898934	3.33E-13
UBE2V2	0.317698528	3.45E-13
RND3	0.317622508	3.50E-13
ANKHD1	-0.317573854	3.53E-13
TGIF1	0.317468661	3.60E-13
MEST	0.317442928	3.61E-13
COA7	0.317289437	3.71E-13
DBN1	0.317056611	3.87E-13
RASSF1	-0.317044954	3.88E-13
HMGN3	-0.316831233	4.03E-13
SLC25A16	-0.316540078	4.24E-13
ADIPOR2	0.316393037	4.36E-13
SLC25A4	-0.316272193	4.45E-13
ZNF83	-0.315993585	4.68E-13
CTSE	-0.315194331	5.39E-13
PSMD3	0.315115426	5.47E-13
RRM2B	-0.314877158	5.70E-13
SET	0.314441903	6.16E-13
VRK2	0.314077095	6.57E-13
THRA	-0.313636199	7.10E-13
PAK2	0.313580817	7.17E-13
DENND2D	-0.313396321	7.41E-13
ADAM17	0.313317132	7.51E-13
ADCY9	-0.313216954	7.65E-13
AATF	0.312072126	9.35E-13
TRAF1	-0.311994081	9.48E-13
CAPN8	-0.311915146	9.61E-13

RASSF7	-0.31188238	9.67E-13
SULT1C2	-0.31150174	1.03E-12
INPP5D	-0.31147273	1.04E-12
ACTB	0.311416455	1.05E-12
MAT2A	-0.31141631	1.05E-12
LTA	-0.311385953	1.05E-12
SSB	0.310903452	1.15E-12
RASSF4	-0.310700362	1.19E-12
PSMC5	0.309992008	1.34E-12
P4HA1	0.309982697	1.35E-12
SMC2	0.309971466	1.35E-12
FTSJ1	0.309949291	1.35E-12
COLGALT1	0.309668258	1.42E-12
DNAJB11	0.309574915	1.45E-12
TNFAIP8L2	-0.309472067	1.47E-12
DFFB	-0.309291894	1.52E-12
CASP1	-0.309046546	1.58E-12
IL6ST	-0.308316813	1.80E-12
LUC7L3	-0.308119729	1.86E-12
TAF13	0.307684698	2.00E-12
GPLD1	-0.307682693	2.01E-12
PRSS12	-0.307603512	2.03E-12
CD27	-0.307379008	2.11E-12
TMPO	0.307048197	2.24E-12
TIAF1	-0.306990542	2.26E-12
SERPINE1	0.306944346	2.28E-12
ANKRD10	-0.306630223	2.40E-12
BIN3	-0.30653308	2.44E-12
TNFSF15	-0.306360271	2.52E-12
ADM	0.30634646	2.52E-12
ASB2	-0.306226123	2.58E-12
MAP3K1	-0.305295408	3.02E-12
TGFBR3	-0.304503016	3.46E-12
LEFTY2	-0.304113445	3.69E-12
RTN4	0.303749892	3.93E-12
MPHOSPH8	-0.303665752	3.99E-12
PDK2	-0.303415514	4.16E-12
AMPD1	-0.303066633	4.41E-12
PRPF4	0.30305009	4.42E-12
SECISBP2L	-0.303014405	4.45E-12
ERBB4	-0.302918551	4.52E-12
ARL6IP1	0.30282639	4.60E-12
ACTL6A	0.302532509	4.83E-12

CENPK	0.302249113	5.07E-12
KPNA5	-0.302198299	5.11E-12
FHIT	-0.302086223	5.21E-12
PLAUR	0.302037033	5.25E-12
ERAL1	0.302021237	5.27E-12
CBX3	0.301999233	5.28E-12
CAMK1D	-0.30171676	5.54E-12
TNFRSF13C	-0.301663918	5.59E-12
HOMER1	0.301479183	5.77E-12
TFAP2A	0.301452061	5.80E-12
SMPD3	-0.301382888	5.86E-12
TSC22D3	-0.301355308	5.89E-12
RRM1	0.300912447	6.35E-12
TMEM63A	-0.300617122	6.67E-12
SERBP1	0.300067175	7.31E-12
PRKCE	-0.299743335	7.72E-12
NFATC1	-0.299514336	8.02E-12
TNFSF8	-0.299475049	8.07E-12
PSMB7	0.298835558	8.98E-12
GORASP2	0.298526193	9.46E-12
GLCCI1	-0.298437912	9.60E-12
SEMA3C	0.298340714	9.75E-12
CXCL8	0.298274607	9.86E-12
PRDM5	-0.298023267	1.03E-11
ALG8	0.298002478	1.03E-11
TPM4	0.297865376	1.06E-11
ARRB1	-0.297613073	1.10E-11
NCL	0.297491929	1.12E-11
DOK1	-0.297405675	1.14E-11
ATP2A2	0.29731508	1.16E-11
GNB1	0.297290621	1.16E-11
ARAP1	-0.297175528	1.18E-11
COPS6	0.296899596	1.24E-11
RPP25	0.296389583	1.35E-11
EIF3J	0.296365948	1.35E-11
PIK3CG	-0.296229076	1.38E-11
LRIF1	0.296152689	1.40E-11
PPIA	0.296127069	1.41E-11
HTRA4	-0.296057292	1.42E-11
VRK1	0.295951781	1.45E-11
GPR19	0.295943457	1.45E-11
RPE	0.29591546	1.46E-11
SLC5A6	0.295511297	1.56E-11

BMP3	-0.295141781	1.65E-11
BRD8	-0.294837588	1.74E-11
IRF9	-0.294566907	1.82E-11
MNDA	-0.294501554	1.84E-11
AP1G2	-0.294451829	1.85E-11
SHC1	0.294384538	1.87E-11
MSH6	0.294078503	1.97E-11
IRF2	-0.293983578	2.00E-11
FNIP2	-0.293911337	2.02E-11
HEMGN	-0.29379285	2.06E-11
HOXB7	0.293767328	2.07E-11
LTBR	0.29372118	2.09E-11
BUB3	0.293396134	2.20E-11
CAD	0.293165154	2.29E-11
ALDH1B1	0.292995563	2.35E-11
CD4	-0.292944249	2.37E-11
AQP4-AS1	-0.292932995	2.37E-11
RAD54B	0.292795893	2.43E-11
ARHGEF17	-0.292409754	2.59E-11
GSK3A	0.292083619	2.73E-11
NUDT12	-0.291789781	2.86E-11
IFFO1	-0.291602372	2.95E-11
CCNL1	-0.291512748	2.99E-11
TXNDC9	0.291290287	3.10E-11
ING5	-0.290937977	3.28E-11
E2F1	0.290805954	3.35E-11
SNRPA1	0.29050706	3.52E-11
LLPH	0.289831915	3.92E-11
GAS8	-0.289733617	3.99E-11
KLK6	0.289620568	4.06E-11
SEMA3A	0.289597778	4.07E-11
RUVBL1	0.289433702	4.18E-11
IL3RA	-0.289122835	4.40E-11
MIF	0.288770751	4.65E-11
PSMD7	0.288767327	4.65E-11
DNM1L	0.288718865	4.69E-11
MYO9A	-0.288458707	4.89E-11
NMD3	0.288443173	4.90E-11
CR2	-0.288366058	4.96E-11
PTCHD4	-0.288220601	5.08E-11
ACADVL	-0.28807967	5.20E-11
PECAM1	-0.287700382	5.52E-11
SLC6A8	0.287691313	5.53E-11

RTN1	-0.287687587	5.53E-11
ID2	-0.287567273	5.64E-11
VASP	0.287521433	5.68E-11
DHCR7	0.287348509	5.84E-11
OGN	-0.287294273	5.89E-11
DAD1	0.287012445	6.16E-11
IGFBP3	0.286959333	6.21E-11
PRMT2	-0.286282662	6.92E-11
SULT1A2	-0.286184624	7.02E-11
OPA1	0.2856963	7.59E-11
KANK1	-0.285662868	7.63E-11
GADD45G	-0.285114971	8.32E-11
GUCY1A2	-0.284865447	8.65E-11
FAM214A	-0.284590438	9.04E-11
TSC22D2	0.284542839	9.11E-11
PMAIP1	0.284205791	9.60E-11
TIMM50	0.284033263	9.87E-11
CTSS	-0.283911895	1.01E-10
MYO1E	0.283817738	1.02E-10
ATM	-0.283789914	1.03E-10
RUVBL2	0.283693704	1.04E-10
PDPK1	-0.283296873	1.11E-10
IQGAP2	-0.283108067	1.14E-10
PSMB3	0.283064392	1.15E-10
CDK5R1	0.282651995	1.23E-10
PTHLH	0.282571201	1.24E-10
ERCC5	-0.282141997	1.33E-10
SLA	-0.282030871	1.35E-10
RBMS3	-0.281963755	1.36E-10
GIMAP4	-0.281864808	1.39E-10
RPS6KA5	-0.281797065	1.40E-10
SULF1	0.281673487	1.43E-10
COLQ	-0.281633806	1.44E-10
EGLN2	-0.281563524	1.45E-10
BTC	-0.281137078	1.55E-10
CDIP1	-0.281129247	1.55E-10
KCNA5	-0.280706574	1.66E-10
BCL2	-0.280603861	1.69E-10
FGFR3	-0.280567821	1.69E-10
PPP4C	0.280274645	1.77E-10
C15orf48	0.280257922	1.78E-10
PSMD8	0.280158478	1.81E-10
YPEL3	-0.280032924	1.84E-10

DIABLO	0.279793533	1.91E-10
PCNA	0.279704746	1.94E-10
CTSD	-0.279660845	1.95E-10
SMNDC1	0.279446781	2.02E-10
FGF14	-0.27939953	2.03E-10
TES	0.279329532	2.05E-10
ELMO1	-0.279176993	2.10E-10
SPG11	-0.279162891	2.11E-10
TNS4	0.279007013	2.16E-10
IL17RD	0.278625962	2.29E-10
MGLL	-0.278438703	2.35E-10
IL7R	-0.278245978	2.43E-10
E2F6	0.278114068	2.48E-10
CFLAR	-0.278100957	2.48E-10
KLF2	-0.277299806	2.80E-10
RRP1B	0.277005686	2.93E-10
TCEAL2	-0.276854344	3.00E-10
TWIST1	0.27682872	3.01E-10
MUC1	-0.276748584	3.05E-10
HLA-F	-0.276600382	3.12E-10
ZNF10	-0.276533131	3.15E-10
SUOX	-0.276475067	3.18E-10
ZNF302	-0.276264515	3.28E-10
TNFRSF1B	-0.2760221	3.41E-10
POLK	-0.275674061	3.59E-10
BMP5	-0.275493315	3.69E-10
TRIM13	-0.275481957	3.70E-10
VCAN	0.275257144	3.83E-10
ZRSR2	-0.275202947	3.86E-10
NPRL2	-0.275144997	3.89E-10
CD3E	-0.274977014	3.99E-10
HELLS	0.274266671	4.45E-10
TOX	-0.274118067	4.55E-10
DSP	0.274082831	4.57E-10
SLC3A2	0.273993637	4.64E-10
TDRD3	-0.273785306	4.78E-10
RAB36	-0.273661097	4.87E-10
MBP	-0.273372479	5.09E-10
LTF	-0.273355425	5.10E-10
WDR12	0.273253758	5.18E-10
MARK4	0.273248809	5.19E-10
ITPR2	-0.272974267	5.40E-10
CD180	-0.272616632	5.70E-10

CCNG1	-0.272467498	5.83E-10
MTR	-0.272133671	6.13E-10
GAS6	-0.271907111	6.34E-10
DUSP26	-0.271856324	6.39E-10
PIK3CD	-0.271798453	6.45E-10
PAIP1	0.271783361	6.46E-10
CUL2	0.271608096	6.63E-10
PTRH2	0.27151658	6.72E-10
STAMBP	0.2712271	7.02E-10
SPRY4-IT1	-0.271141494	7.11E-10
RNASE6	-0.271094313	7.16E-10
NRBP2	-0.270842618	7.44E-10
ZNF146	0.270841428	7.44E-10
UPF3A	-0.270819015	7.46E-10
IRF4	-0.270532192	7.79E-10
B4GALT6	0.270456753	7.88E-10
APBB1	-0.270382597	7.96E-10
HIF1A	0.270335077	8.02E-10
PRPF38B	-0.269780811	8.71E-10
CEBPG	0.269485971	9.10E-10
PDCD5	0.268968411	9.82E-10
OCLN	-0.268918248	9.90E-10
ZNF224	-0.268861182	9.98E-10
PPP2R1B	0.268726006	1.02E-09
CORO1C	0.268590665	1.04E-09
PKIB	0.268526585	1.05E-09
TAF1C	-0.268518089	1.05E-09
SNRPG	0.2685039	1.05E-09
SIX3	0.268448213	1.06E-09
LCK	-0.267933898	1.14E-09
PSME4	0.267834872	1.16E-09
RAB11FIP2	-0.267830654	1.16E-09
SUMO1	0.267717849	1.18E-09
POU2AF1	-0.267069207	1.30E-09
LMO2	-0.267028082	1.31E-09
TNFAIP1	0.26692449	1.33E-09
RABGAP1L	-0.266556935	1.40E-09
SERPINI2	-0.266492722	1.41E-09
PDIA4	0.266240966	1.47E-09
SPATA4	-0.266232167	1.47E-09
MSH2	0.266117479	1.49E-09
CHCHD2	0.265466173	1.64E-09
GAS2L1	0.264890454	1.79E-09

S100A11	0.264854716	1.80E-09
UBE2D1	0.264769454	1.82E-09
FERMT3	-0.264740337	1.83E-09
SNRK	-0.264624109	1.86E-09
MBTPS2	0.264346668	1.93E-09
PAK4	0.264320801	1.94E-09
MT1X	0.264272648	1.95E-09
KPNA1	0.264254118	1.96E-09
PRKDC	0.264038603	2.02E-09
SQLE	0.26378213	2.10E-09
NTSR1	0.263718498	2.12E-09
SH3BGRL	-0.263594	2.16E-09
RRAD	-0.263585963	2.16E-09
FGF5	0.263389133	2.22E-09
ETV1	-0.263286167	2.25E-09
BCL11A	-0.263098168	2.32E-09
PAWR	0.262940117	2.37E-09
MLANA	-0.262740941	2.44E-09
CDC34	0.26263487	2.48E-09
ZEB2	-0.262534638	2.51E-09
PNMA1	0.262028823	2.70E-09
PPP2R2C	0.26188559	2.76E-09
DDX21	0.261835555	2.78E-09
PRKCZ	-0.261794444	2.79E-09
TLE1	0.261769225	2.81E-09
AKAP1	-0.261690876	2.84E-09
TAF6	0.261646452	2.85E-09
TMX4	-0.261563855	2.89E-09
FXR1	0.261484719	2.92E-09
PARP11	-0.261330964	2.99E-09
SMPD4	0.260854086	3.20E-09
CD3G	-0.26079135	3.23E-09
FAM111A	-0.260749781	3.25E-09
RPAP3	0.260740868	3.25E-09
MORF4L2	0.260596869	3.32E-09
TIA1	-0.260500877	3.36E-09
RAPGEF6	-0.2604311	3.40E-09
DOCK10	-0.260420534	3.40E-09
GNA12	0.260414553	3.41E-09
CTSZ	-0.260303851	3.46E-09
CCP110	-0.260095976	3.56E-09
BMF	-0.259967131	3.63E-09
SACS	0.259799354	3.72E-09

CHST9	-0.259598083	3.83E-09
PRDM2	-0.259543518	3.86E-09
TACC1	-0.259403583	3.93E-09
GNA14	-0.259353076	3.96E-09
PSMD4	0.259205501	4.05E-09
UBC	0.259018912	4.15E-09
NTF4	-0.258894099	4.23E-09
NPDC1	-0.258686378	4.35E-09
CDK5RAP3	-0.258601688	4.41E-09
SRSF11	-0.258488823	4.48E-09
TOPBP1	0.258113689	4.72E-09
TCL1A	-0.257963391	4.82E-09
PSMA5	0.257868595	4.89E-09
AGAP2	-0.257601816	5.08E-09
TGFBR2	-0.257565545	5.10E-09
CARD9	-0.257422582	5.21E-09
BASP1	0.257397971	5.22E-09
GPC3	-0.257346041	5.26E-09
RUNX3	-0.25700837	5.52E-09
SLC7A1	0.256884316	5.62E-09
MAP3K14	-0.256799963	5.68E-09
FBLN5	-0.256707294	5.76E-09
WDR49	-0.256657094	5.80E-09
MADD	-0.256562728	5.88E-09
ERG	-0.256449141	5.97E-09
CHEK2	0.256408934	6.00E-09
CLIP1	0.256373271	6.03E-09
IL1R2	0.256366617	6.04E-09
GORASP1	-0.256129482	6.24E-09
TAF1A	0.256122496	6.25E-09
RALB	0.256077699	6.29E-09
TLR3	-0.255929962	6.42E-09
ITGAV	0.255667023	6.66E-09
TNFRSF10C	-0.255656495	6.67E-09
GMNN	0.255318227	6.99E-09
DDB2	-0.254978392	7.33E-09
LINC00847	-0.254501498	7.84E-09
ZCRB1	0.254468274	7.88E-09
GLRX2	0.254304879	8.06E-09
UGDH	0.254196311	8.18E-09
PDCD4	-0.254054538	8.34E-09
CADM1	-0.253769981	8.68E-09
AATK	-0.253654252	8.82E-09

MAGEA3	0.253357673	9.19E-09
CAPN9	-0.253345725	9.20E-09
DAG1	0.253141954	9.47E-09
CXCR4	-0.253110021	9.51E-09
ACOX2	-0.252968163	9.70E-09
DHRS3	-0.252915399	9.77E-09
UBE2Z	0.252883256	9.81E-09
NPIPB3	-0.252780704	9.95E-09
GTF2IRD1	0.252634353	1.02E-08
TET2	-0.252564674	1.03E-08
CTSL	0.252495263	1.04E-08
EDRF1	-0.25243497	1.04E-08
NEDD9	-0.252190882	1.08E-08
MPHOSPH10	0.252071987	1.10E-08
WWC1	-0.251758617	1.15E-08
ABCA2	-0.251684211	1.16E-08
ZNF117	-0.251638307	1.16E-08
TRIM28	0.251527678	1.18E-08
EVI2A	-0.251437466	1.20E-08
RNF144B	-0.25135647	1.21E-08
SUZ12	0.250881688	1.29E-08
RMDN3	-0.250611884	1.34E-08
GNG4	0.250483413	1.37E-08
EXT1	0.250380904	1.38E-08
POLA2	0.250280242	1.40E-08
FAP	0.250232365	1.41E-08
NLRP3	-0.250231181	1.41E-08
GCLC	0.250092369	1.44E-08
PSMC3	0.249925566	1.47E-08
NOX4	0.249861072	1.49E-08
PICALM	0.249759765	1.51E-08
SLC4A1AP	0.249678016	1.52E-08
FADS1	0.249487916	1.56E-08
VWA5A	-0.24928414	1.61E-08
MCM3	0.249282786	1.61E-08
ITPKB	-0.249252663	1.61E-08
PSMB6	0.249232595	1.62E-08
HSPA4	0.249047975	1.66E-08
ZER1	-0.24899142	1.67E-08
DNAJC2	0.248951483	1.68E-08
CTSF	-0.24880585	1.72E-08
RNASEL	-0.248667079	1.75E-08
RNF7	0.248601273	1.76E-08

PITPNB	0.248564671	1.77E-08
RDX	0.248380763	1.82E-08
TUBA4A	0.24829445	1.84E-08
PSMC2	0.248209629	1.86E-08
SYNJ2	0.248183252	1.87E-08
SLC16A5	-0.248021752	1.91E-08
ITPR1	-0.247653533	2.01E-08
GNG2	-0.247558944	2.03E-08
RTKN	0.247396606	2.08E-08
ICOS	-0.247251834	2.12E-08
RPN1	0.247201776	2.13E-08
FGF12	0.247009256	2.19E-08
RPS6KA2	-0.24697142	2.20E-08
GPX3	-0.246915199	2.22E-08
BCCIP	0.246899099	2.22E-08
DCUN1D1	0.246705647	2.28E-08
TYK2	-0.246502765	2.34E-08
DNAJB4	0.246327061	2.40E-08
CPVL	-0.246302692	2.41E-08
ACAT1	-0.246264861	2.42E-08
SP140	-0.246190676	2.44E-08
FOS	-0.245930084	2.53E-08
HSPB8	-0.245915779	2.53E-08
BCL2L10	0.24588785	2.54E-08
PCCA	-0.245221121	2.78E-08
TMEM106C	0.245204761	2.79E-08
STRN3	0.244910633	2.90E-08
TNFRSF17	-0.244909425	2.90E-08
TRAC	-0.244806976	2.94E-08
ZNF101	-0.244454939	3.08E-08
NACC1	0.244298336	3.15E-08
ITGB7	-0.244254246	3.17E-08
RRS1	0.244089917	3.24E-08
ATXN3	-0.243782521	3.37E-08
IER3	0.2437416	3.39E-08
IFT57	-0.243700467	3.41E-08
ANP32B	0.243531159	3.49E-08
STAT5B	-0.243485053	3.51E-08
PMEPA1	0.243308163	3.59E-08
ZNF189	-0.243111844	3.68E-08
MSX2	0.243098483	3.69E-08
ZCCHC24	-0.242771099	3.85E-08
PSMB1	0.242640582	3.92E-08

B4GAT1	-0.242431799	4.03E-08
PTPN4	-0.242361075	4.07E-08
HCK	-0.242217092	4.15E-08
CEP112	-0.242184695	4.17E-08
HLA-J	-0.242000883	4.27E-08
GSDMB	-0.241932626	4.31E-08
SLC16A7	-0.241517984	4.55E-08
EPHB2	0.241441491	4.59E-08
NOTCH3	0.241018337	4.86E-08
HSPA13	0.241008159	4.86E-08
ZC3HC1	0.240786668	5.01E-08
TLK1	0.240344755	5.30E-08
PGM1	0.240135809	5.45E-08
FHOD1	-0.240030967	5.53E-08
FANK1	-0.240002032	5.55E-08
TMEM42	-0.239808829	5.69E-08
CCDC59	0.239650254	5.81E-08
XAF1	-0.238923449	6.39E-08
PEBP1	-0.238750767	6.53E-08
ZNF185	0.238744357	6.54E-08
GZMM	-0.238631032	6.63E-08
HMGB2	0.238627415	6.64E-08
KYNU	0.238055217	7.15E-08
ATP6V1B2	-0.237971116	7.23E-08
KRAS	0.237890451	7.30E-08
RALBP1	0.237843467	7.35E-08
TIMM17A	0.237750381	7.44E-08
MARCKS	0.237664476	7.52E-08
MYBPC2	-0.237632433	7.55E-08
PPIF	0.237461902	7.72E-08
RPA3	0.23723939	7.94E-08
DPY19L1	0.237195724	7.99E-08
TNFSF14	-0.237061654	8.13E-08
GSN	-0.236957755	8.24E-08
ZNF443	-0.236932396	8.27E-08
CFD	-0.236788003	8.42E-08
NCAPD3	0.236708775	8.51E-08
EIF4E	0.236399594	8.85E-08
SPP1	0.236335383	8.93E-08
LGALS9	-0.236312806	8.95E-08
TLR4	-0.236266078	9.01E-08
INTS9	-0.236218173	9.06E-08
RECQL	0.236022109	9.29E-08

CASP10	-0.235948105	9.38E-08
PRKCH	-0.235831785	9.52E-08
SERPINH1	0.235777408	9.59E-08
PARP16	-0.235712389	9.67E-08
SPECC1	0.235645086	9.75E-08
USP1	0.235197463	1.03E-07
RNF145	-0.23500256	1.06E-07
CD3D	-0.234903443	1.07E-07
RNF130	-0.234802585	1.09E-07
FBN2	0.234787378	1.09E-07
PSMD9	0.234591579	1.12E-07
PARP10	-0.234501587	1.13E-07
FAM189B	0.234498361	1.13E-07
PSMA4	0.233961613	1.21E-07
CCR5	-0.233893161	1.22E-07
NBEAL2	-0.233851017	1.23E-07
TMEM59	-0.233574363	1.27E-07
ADAM9	0.233506766	1.28E-07
DCC	-0.233324382	1.31E-07
MAGEA6	0.233174553	1.34E-07
PSMB4	0.233166187	1.34E-07
EIF3I	0.23308993	1.35E-07
FOXN3	-0.233016684	1.36E-07
NDUFAF1	-0.23294051	1.38E-07
CDK11A	-0.232740491	1.41E-07
PTPN11	0.232725473	1.41E-07
SHOX2	0.232568894	1.44E-07
RPGR	-0.232560196	1.44E-07
TNFRSF19	-0.232459216	1.46E-07
IRAK1	0.232272508	1.50E-07
PITX1	0.231736574	1.60E-07
ATAD5	0.231632804	1.62E-07
NRADDP	-0.23151086	1.65E-07
ADRM1	0.231466635	1.66E-07
PPIP5K1	-0.231303889	1.69E-07
ZDHHC16	-0.231191361	1.72E-07
ING3	-0.231171198	1.72E-07
CCNT2	-0.231069186	1.74E-07
SLCO3A1	-0.231030912	1.75E-07
PIDD1	-0.230980456	1.76E-07
EIF5A2	0.230909624	1.78E-07
IL5	-0.230252524	1.93E-07
JMJD6	0.230245148	1.93E-07

ADA	0.230156757	1.95E-07
IGHA1	-0.230153306	1.96E-07
SOX4	0.230062328	1.98E-07
DNAJB6	0.229947226	2.01E-07
EEF1E1	0.2299317	2.01E-07
BOK	-0.229755775	2.06E-07
TRIAP1	0.22971343	2.07E-07
TCF19	0.229389119	2.15E-07
GTF2F2	0.229360497	2.16E-07
CLK3	-0.229351312	2.16E-07
RNFT2	0.228876041	2.29E-07
PITPNC1	0.228796833	2.32E-07
SMC4	0.22877754	2.32E-07
PSPH	0.228740149	2.33E-07
PPP3R1	0.228640547	2.36E-07
CHAC2	0.228579461	2.38E-07
PSMB10	-0.228173327	2.50E-07
ZNF137P	-0.228091381	2.53E-07
CTNNBL1	0.22794635	2.57E-07
CABIN1	-0.227672557	2.66E-07
PSMA7	0.227586776	2.69E-07
PCMT1	0.227062598	2.87E-07
CAB39L	-0.226995431	2.89E-07
LDB3	-0.226976261	2.90E-07
ZNF717	-0.226945327	2.91E-07
SLU7	-0.226922537	2.92E-07
IWS1	0.226826772	2.96E-07
CYB5R1	-0.226485803	3.08E-07
NAIP	-0.226415248	3.11E-07
RAB27A	-0.226171217	3.20E-07
GSDMD	-0.226106861	3.23E-07
ERN1	-0.226040762	3.26E-07
TNFRSF12A	0.226038063	3.26E-07
HNRNPK	0.226028932	3.26E-07
FBXL3	-0.225914762	3.31E-07
GUSBP11	-0.225894991	3.31E-07
CNKSR2	-0.225752354	3.37E-07
STK24	0.225742292	3.38E-07
RAPGEF4	-0.225680432	3.40E-07
PLD3	-0.225670052	3.41E-07
RHOBTB3	0.225598582	3.44E-07
CRYZL1	-0.225484088	3.49E-07
KNTC1	0.22534051	3.55E-07

TNFRSF21	0.225323871	3.55E-07
FAM3C	0.224997207	3.70E-07
ID4	-0.22458447	3.89E-07
HBS1L	0.224511965	3.92E-07
NTRK1	-0.224427968	3.97E-07
FGF22	-0.224360099	4.00E-07
CLSTN1	0.224166899	4.09E-07
GLRX3	0.224163947	4.09E-07
ARMC1	0.224122811	4.12E-07
WDR11	-0.224110443	4.12E-07
MYCT1	-0.223802566	4.28E-07
LASP1	0.223773361	4.29E-07
SLC12A4	-0.223758697	4.30E-07
GDF7	-0.223651718	4.36E-07
PRPF39	-0.223546965	4.41E-07
SMO	0.223435363	4.47E-07
GNAQ	-0.223433221	4.47E-07
LAIR1	-0.223346186	4.52E-07
RAD21	0.223290444	4.55E-07
EPB41	-0.223045475	4.69E-07
FIG4	-0.222964693	4.74E-07
DPP4	-0.222944265	4.75E-07
MLLT3	-0.222744064	4.86E-07
SEL1L3	-0.222743037	4.86E-07
SLC38A2	0.222726978	4.87E-07
CAPN12	-0.222426773	5.05E-07
ACER1	-0.222397245	5.07E-07
BIRC7	-0.222393671	5.07E-07
CDK15	-0.222300119	5.13E-07
RGS5	-0.222288158	5.14E-07
PRUNE2	-0.222117783	5.25E-07
PTPRO	-0.22209185	5.26E-07
CAPN11	-0.222020985	5.31E-07
SNAPC1	0.221641393	5.56E-07
TXN	0.221253904	5.82E-07
PTPN22	-0.221109556	5.92E-07
HSPA1B	0.221097877	5.93E-07
MAP3K8	-0.220883381	6.09E-07
AK4	0.220721168	6.20E-07
PRODH	-0.220582522	6.31E-07
GOSR1	0.220383555	6.46E-07
THYN1	-0.220207771	6.60E-07
BACH1	0.220108786	6.68E-07

HK2	0.22008373	6.70E-07
NMT1	0.219994148	6.77E-07
TNNI3	0.219978756	6.78E-07
BID	0.219897161	6.85E-07
RNU6-807P	-0.219860016	6.88E-07
BCL2L14	-0.219795991	6.93E-07
GCC2	-0.219738798	6.98E-07
HSP90AA1	0.219614614	7.08E-07
KCNG1	0.219582972	7.11E-07
SLC15A3	-0.219322231	7.33E-07
GNGT1	0.219210513	7.43E-07
SMAD7	-0.219176823	7.46E-07
NCDN	-0.21917601	7.46E-07
YWHAB	0.218972934	7.64E-07
TMEM147	0.218884549	7.72E-07
HNRNPDL	-0.218842607	7.76E-07
ZNF254	-0.218773827	7.83E-07
HNRNPU	0.218686192	7.91E-07
B3GALNT1	0.218670406	7.92E-07
HGF	-0.218662068	7.93E-07
GSKIP	0.218589462	8.00E-07
NUDT11	0.218417216	8.16E-07
ECSCR	-0.218273323	8.30E-07
TMEM214	0.218128393	8.45E-07
BCL2L13	0.218030264	8.55E-07
ENPP2	-0.217892508	8.69E-07
PIK3C2B	-0.217806642	8.78E-07
MYCBP2	-0.217738119	8.85E-07
DAP3	0.217547777	9.05E-07
TNPO1	0.217519006	9.08E-07
GLA	0.217391287	9.22E-07
TNFRSF1A	0.217134409	9.50E-07
SAC3D1	0.217127452	9.51E-07
TCN2	-0.217058432	9.59E-07
ZBP1	-0.216924173	9.74E-07
TREM2	-0.216919613	9.74E-07
MARCKSL1	0.216900742	9.76E-07
MAGEA1	0.216740104	9.95E-07
PSMC1	0.216644234	1.01E-06
KCNN3	-0.216590433	1.01E-06
PHLDA2	0.2163915	1.04E-06
ZXDA	-0.216376709	1.04E-06
GRWD1	0.216362133	1.04E-06

PPTC7	0.216318719	1.05E-06
CEBPZ	0.216222313	1.06E-06
FANCA	0.21596943	1.09E-06
WDR3	0.21590749	1.10E-06
MFF	0.215857136	1.10E-06
SMC3	0.215854827	1.10E-06
LGALS1	0.215750045	1.12E-06
INTS1	0.21569638	1.12E-06
SLC37A2	-0.21568916	1.13E-06
VCP	0.215681444	1.13E-06
CTSW	-0.215406199	1.16E-06
THUMPD1	-0.215298	1.18E-06
DDX39A	0.215275617	1.18E-06
MAB21L1	-0.215236501	1.19E-06
AKT1S1	0.214887255	1.24E-06
DYRK1A	-0.214582433	1.28E-06
BAZ2B	-0.214561837	1.28E-06
FSTL1	0.214212744	1.34E-06
ESR2	-0.214203128	1.34E-06
STEAP2-AS1	0.214113527	1.35E-06
RPS6KB1	0.214063458	1.36E-06
FXYD6	-0.214054717	1.36E-06
AKTIP	-0.214051973	1.36E-06
CENPQ	0.213947697	1.38E-06
PSMA2	0.213905787	1.39E-06
GDF15	-0.213846147	1.40E-06
DIDO1	-0.213827182	1.40E-06
F13A1	-0.213583122	1.44E-06
SPIN2B	-0.213564772	1.44E-06
CHORDC1	0.212868599	1.56E-06
SYF2	-0.212552426	1.62E-06
ITGB5	0.212532157	1.62E-06
ZDHHC11	-0.212471299	1.64E-06
CEBPB	0.212461496	1.64E-06
TIMM9	0.212459614	1.64E-06
PAK1	0.212385011	1.65E-06
SLC38A6	-0.212372761	1.65E-06
SCP2	-0.212337817	1.66E-06
OAS3	0.212272651	1.67E-06
GPM6B	-0.212253534	1.68E-06
RCN3	0.212177778	1.69E-06
CLEC16A	-0.212143539	1.70E-06
NBN	0.212117329	1.70E-06

BCL2L2	-0.212043775	1.72E-06
HSD17B10	0.211795144	1.77E-06
PTGES	0.211581988	1.81E-06
PUF60	0.211492696	1.83E-06
MTDH	0.211470414	1.83E-06
GLB1L2	-0.211266592	1.88E-06
CERK	-0.211159452	1.90E-06
SGK1	-0.210903707	1.96E-06
TRAPPC6A	-0.210887524	1.96E-06
ITGA7	-0.210827493	1.97E-06
FNBP1	-0.210709396	2.00E-06
HSPA9	0.210576089	2.03E-06
PITPNA	-0.210484756	2.05E-06
BCL10	0.21033163	2.09E-06
NUDT6	-0.210268301	2.10E-06
VPS26A	0.209829292	2.21E-06
SLC35G2	0.209826953	2.21E-06
JMY	-0.209660262	2.26E-06
TBC1D2B	-0.20958014	2.28E-06
EXOG	-0.209339907	2.34E-06
PIEZO2	-0.209254106	2.36E-06
NOC2L	0.209214178	2.37E-06
MTFP1	0.208604334	2.54E-06
AHCTF1	0.208580383	2.55E-06
KIF5B	0.20850977	2.57E-06
OSCAR	-0.20846743	2.58E-06
PPP4R2	0.208432301	2.59E-06
IL1A	0.208174437	2.67E-06
TFRC	0.208002936	2.72E-06
TCEA1	0.207808863	2.78E-06
CXCL13	-0.207782811	2.79E-06
GTF3C3	0.207708253	2.81E-06
MSX1	0.207507159	2.88E-06
HAUS6	0.207363077	2.92E-06
LYRM1	-0.207312205	2.94E-06
NELL1	-0.207171192	2.99E-06
SKIL	0.207012207	3.04E-06
CD2AP	0.206721148	3.14E-06
UCHL1	0.20639305	3.26E-06
PSMA6	0.206029866	3.39E-06
CEP70	-0.205989421	3.41E-06
EIF3M	0.205680305	3.53E-06
ZFP36	-0.205672516	3.53E-06

BRAP	0.205609519	3.56E-06
APOH	-0.20546333	3.62E-06
IFT74	-0.205411345	3.64E-06
TRAPPC13	-0.205267817	3.70E-06
RAC2	-0.205239674	3.71E-06
BCL6	-0.205079538	3.77E-06
XPO1	0.20472217	3.93E-06
IFRD1	0.20469756	3.94E-06
IPPK	0.204690101	3.94E-06
VPS72	0.204663796	3.95E-06
TCF3	0.204562039	4.00E-06
HOXA9	0.204246027	4.14E-06
WDR18	0.204173227	4.17E-06
RAD18	0.203605973	4.44E-06
ABR	-0.203594138	4.45E-06
PARL	0.203575322	4.46E-06
GNG8	-0.203574214	4.46E-06
ANGPT2	0.203433227	4.53E-06
SKAP2	0.203277271	4.61E-06
BEX2	-0.203250095	4.62E-06
PLEC	0.203192182	4.65E-06
MCTS1	0.203047029	4.72E-06
ICAM2	-0.202978673	4.76E-06
HOXB9	0.202773321	4.87E-06
WDR31	-0.202678976	4.92E-06
TCF7	-0.202474364	5.03E-06
GNGT2	-0.202233318	5.17E-06
WDR7	-0.202191965	5.19E-06
KIF3A	-0.202122824	5.23E-06
FAM162A	0.201925693	5.34E-06
BRMS1	0.201920363	5.35E-06
SERPINE2	0.201827532	5.40E-06
GPR132	-0.201811204	5.41E-06
CAPN10	-0.201602537	5.53E-06
PRPSAP1	0.201496091	5.60E-06
RELA	0.201242937	5.76E-06
IL7	-0.201196787	5.79E-06
LRRC4	-0.200941387	5.95E-06
ITGAM	-0.200793886	6.04E-06
TRIM39	-0.200749948	6.07E-06
PTPRN	0.200461114	6.27E-06
HPGD	-0.200449662	6.28E-06
ZNF516	-0.2001844	6.46E-06

SLC1A5	0.20018357	6.46E-06
KLHL20	-0.200169413	6.47E-06
PLXNC1	-0.200100924	6.52E-06
ISG15	0.199818526	6.72E-06
DMAP1	-0.199325927	7.09E-06
TNFRSF25	-0.199296703	7.11E-06
SRP54	0.199177889	7.20E-06
GKAP1	-0.19912637	7.24E-06
IRS1	0.199081967	7.28E-06
DNASE2	-0.198876277	7.44E-06
N4BP1	-0.198851633	7.46E-06
GNA13	0.198837756	7.47E-06
KIT	-0.198714704	7.57E-06
RNF38	-0.198562505	7.69E-06
NUCB1	-0.198554151	7.70E-06
PRDX1	0.198345528	7.88E-06
NUP93	0.198292524	7.92E-06
PCDHB2	0.198254498	7.95E-06
TMEM45A	0.198207109	7.99E-06
ZNF124	-0.198129471	8.06E-06
PPP1CA	0.198106792	8.08E-06
TTC37	-0.197992424	8.18E-06
FDXR	-0.197866862	8.29E-06
FADS2	0.19786276	8.30E-06
QPRT	0.197783307	8.37E-06
TRADD	-0.197246989	8.86E-06
EI24	0.19722516	8.88E-06
KLLN	-0.197221398	8.88E-06
TYROBP	-0.197108472	8.99E-06
DDIT3	0.197061353	9.04E-06
KANK2	-0.196822598	9.27E-06
ACOT9	0.196743865	9.35E-06
CHI3L1	-0.196652184	9.44E-06
USP48	-0.196306231	9.80E-06
FOXF2	-0.196300495	9.80E-06
RPH3AL	-0.196008133	1.01E-05
AMOT	-0.195882708	1.02E-05
HSP90AB1	0.195421281	1.08E-05
ATF1	0.19540543	1.08E-05
LPCAT1	-0.195315411	1.09E-05
NME4	0.19521977	1.10E-05
F12	0.195104957	1.11E-05
ECHDC1	-0.195100421	1.11E-05

IGHM	-0.195030424	1.12E-05
HLTF	0.194946019	1.13E-05
DNAJA1	0.194523486	1.18E-05
ARMCX6	-0.194305714	1.21E-05
TIMP2	0.194205927	1.22E-05
MAP1B	0.193824518	1.27E-05
SRPK2	0.193594274	1.30E-05
ARAP3	-0.193248176	1.35E-05
OAS1	0.193168243	1.36E-05
NCBP2	0.192897182	1.40E-05
NOMO1	0.192840764	1.41E-05
SLCO4A1	0.192826845	1.41E-05
STX16	-0.192764594	1.42E-05
IL1R1	-0.192741228	1.43E-05
COQ6	-0.192612651	1.45E-05
CREM	0.192524742	1.46E-05
LIG4	-0.192208999	1.51E-05
SLC7A11	0.191985479	1.54E-05
PDCD10	0.191946478	1.55E-05
DACH1	-0.191921519	1.55E-05
VAV1	-0.191484773	1.63E-05
SMPD2	-0.191246272	1.67E-05
GCLM	0.190952023	1.72E-05
SPRY2	-0.190877538	1.73E-05
SPTSSA	0.190810565	1.74E-05
CCNH	-0.190654931	1.77E-05
PTPRZ1	-0.190493373	1.80E-05
TCEAL3	-0.190425766	1.81E-05
HLA-B	-0.190138047	1.87E-05
SNX2	-0.189933617	1.91E-05
CUL4B	0.189846426	1.93E-05
PIM2	-0.18982946	1.93E-05
RNF34	0.18982653	1.93E-05
TMBIM4	-0.189817801	1.93E-05
DHCR24	-0.189795521	1.94E-05
TAOK2	-0.189770824	1.94E-05
ATG5	0.189591142	1.98E-05
ZNF264	-0.189517486	1.99E-05
TNFSF4	0.188358934	2.24E-05
SCML1	-0.188171621	2.29E-05
GRK5	-0.188167358	2.29E-05
SGPL1	0.188082701	2.31E-05
YWHAE	0.187923712	2.34E-05

MAGEE1	-0.187850669	2.36E-05
ERBB3	-0.187721396	2.39E-05
TRIM2	-0.187648053	2.41E-05
SAMHD1	-0.18762402	2.42E-05
UBR2	-0.187441622	2.46E-05
RIF1	0.187331787	2.49E-05
TOP1	0.187327311	2.49E-05
STEAP3	-0.187282785	2.50E-05
LGALS8	-0.187249569	2.51E-05
NINJ1	-0.187214601	2.52E-05
CXCR3	-0.187197587	2.52E-05
DHX36	0.187055235	2.56E-05
FOXO1	-0.186925564	2.59E-05
HTATIP2	0.186806117	2.63E-05
RPS6KC1	0.186768835	2.64E-05
SMC6	0.186638513	2.67E-05
CHAC1	0.186530856	2.70E-05
BMP2	-0.186478453	2.71E-05
PPP2CA	0.186464294	2.72E-05
SLC18A1	-0.186388656	2.74E-05
TRMT10C	0.1863272	2.76E-05
LIMK1	0.186321652	2.76E-05
IGLL3P	-0.186309417	2.76E-05
PRF1	-0.18625218	2.78E-05
HIP1	-0.18607155	2.83E-05
HSPA1A	0.185959571	2.86E-05
CSRNP1	-0.185955167	2.86E-05
RABEP1	-0.185922635	2.87E-05
DTWD1	-0.185902391	2.88E-05
PDE4B	-0.185842414	2.89E-05
DHFR	0.185786204	2.91E-05
SAT1	-0.185758823	2.92E-05
PHGDH	0.185754235	2.92E-05
IL1RAP	0.185724902	2.93E-05
MYBL1	0.185710409	2.93E-05
SUSD5	0.185554969	2.98E-05
GAB2	-0.185539997	2.98E-05
KITLG	-0.185415884	3.02E-05
SERINC5	-0.185232212	3.08E-05
TICAM1	0.185225463	3.08E-05
ZFR	0.1852098	3.08E-05
PDE8A	-0.185195281	3.09E-05
ESF1	0.185111917	3.11E-05

SERPINB10	-0.184910193	3.18E-05
DNMT3A	0.184877215	3.19E-05
SIRT2	-0.184645676	3.26E-05
PPP2R1A	0.184644759	3.26E-05
RPTOR	0.184500091	3.31E-05
MT1H	0.184433206	3.33E-05
IL10	-0.184272016	3.39E-05
UCHL5	0.18422547	3.40E-05
CERS6	0.184084446	3.45E-05
AHI1	-0.184071153	3.46E-05
ST6GALNAC1	-0.184055128	3.46E-05
TNFAIP8	-0.18380114	3.55E-05
JAK3	-0.183759086	3.57E-05
PPID	0.183738343	3.57E-05
PHKG2	-0.183637015	3.61E-05
HSP90B1	0.183471211	3.67E-05
ARHGEF7	-0.183348895	3.71E-05
PCM1	-0.183319022	3.73E-05
ALOX12	-0.18318832	3.77E-05
SOCS1	-0.183031917	3.83E-05
IPO5	0.182843872	3.91E-05
RCAN1	-0.182732654	3.95E-05
MEF2A	-0.182619062	3.99E-05
PRR13	0.182514881	4.03E-05
TEAD1	0.18238897	4.09E-05
SYNGR4	0.182140149	4.19E-05
NTS	0.182034597	4.23E-05
RGS4	0.182023987	4.24E-05
ECHDC3	-0.182021284	4.24E-05
HIPK3	-0.181990904	4.25E-05
KIAA0319	0.181955217	4.26E-05
CAP1	0.181730791	4.36E-05
UBE2H	0.181709165	4.37E-05
TRIM16	0.18148785	4.47E-05
OTUD3	-0.181472325	4.47E-05
BNIP3L	-0.181296577	4.55E-05
ZFAND5	-0.181128146	4.63E-05
NUPR1	-0.180803971	4.78E-05
NRIP1	0.180761498	4.80E-05
YY1	0.1806915	4.83E-05
CSNK2A1	0.180649769	4.85E-05
DEK	0.180584362	4.88E-05
MTERF3	0.18004243	5.15E-05

BTG3	-0.179999146	5.17E-05
PTP4A1	0.179950797	5.19E-05
RAD17	-0.179808911	5.26E-05
RFFL	0.179726905	5.31E-05
IRAK3	-0.179682918	5.33E-05
HCP5	-0.179646423	5.35E-05
PRKAR2B	-0.179639399	5.35E-05
MTRF1	-0.179584663	5.38E-05
PAQR7	0.179550826	5.40E-05
C6orf120	0.179540321	5.40E-05
CREG1	-0.179494648	5.43E-05
CACNA1C	-0.179371445	5.49E-05
MAP2K5	-0.179219651	5.57E-05
KIF16B	-0.178954733	5.72E-05
ZNF43	-0.178923489	5.74E-05
FAIM2	-0.178916294	5.74E-05
GDNF	0.178855898	5.78E-05
BCKDHB	-0.178716965	5.85E-05
STK17B	-0.178266756	6.11E-05
PLAGL1	-0.178235211	6.13E-05
LSM2	0.178219055	6.14E-05
TP53I3	0.178207086	6.15E-05
TOLLIP	-0.178180411	6.17E-05
PRPF4B	-0.178134336	6.19E-05
MAST2	0.178124087	6.20E-05
S100A9	0.178013678	6.27E-05
TCIRG1	-0.177969658	6.29E-05
NGFR	-0.177921924	6.32E-05
HLA-C	-0.177888066	6.34E-05
FST	0.177883621	6.35E-05
DUSP1	-0.177862406	6.36E-05
LARP1	0.177827773	6.38E-05
PIWIL4	-0.177475226	6.60E-05
OLR1	-0.177374839	6.66E-05
NFKBIB	0.177306953	6.71E-05
APC	-0.177205575	6.77E-05
RMI1	0.177175141	6.79E-05
PRDM13	0.176944946	6.95E-05
LTBP4	-0.176864581	7.00E-05
FAS	-0.176660484	7.14E-05
KAT5	-0.176343206	7.36E-05
PDCD11	0.176292444	7.39E-05
TMEM184B	0.176276897	7.41E-05

IRF3	-0.17613741	7.50E-05
DDIT4	0.176132864	7.51E-05
CFL2	0.176107382	7.53E-05
HYOU1	0.176065464	7.56E-05
MAPKAPK3	-0.176012477	7.59E-05
PPP3R2	-0.17598133	7.62E-05
NID1	0.175918618	7.66E-05
CSNK2A2	0.175848615	7.71E-05
GGCX	-0.175809338	7.74E-05
RPS6KA3	-0.175787368	7.76E-05
PALLD	0.17575544	7.78E-05
GLI1	-0.175576842	7.92E-05
CETN3	-0.175473194	8.00E-05
PSMC6	0.17538557	8.06E-05
CSNK2B	0.17497306	8.39E-05
ZNF598	0.174935445	8.42E-05
CASP2	0.174840092	8.49E-05
PSMA1	0.174742795	8.57E-05
TMX1	0.174706705	8.60E-05
EID1	-0.174407576	8.85E-05
HLA-DQA2	-0.174361358	8.89E-05
SH3GLB1	0.17427213	8.96E-05
ZFC3H1	-0.174098299	9.11E-05
KLHL9	-0.173976839	9.22E-05
P4HA2	0.17387866	9.30E-05
FASTKD2	0.173845451	9.33E-05
MMP1	0.173734567	9.43E-05
CARD14	0.173704864	9.46E-05
ENAH	0.173387164	9.74E-05
CCPG1	-0.1733607	9.77E-05
CDKN2AIP	-0.173293582	9.83E-05
GNB1L	0.173235744	9.88E-05
MAGEH1	-0.1732003	9.92E-05
FSTL3	0.173170848	9.94E-05
IFNL3	0.173111478	1.00E-04
ARPC1B	0.173048813	0.000100582
FGF9	-0.172906623	0.000101935
PIBF1	-0.172879446	0.000102196
FGF10	-0.172858455	0.000102397
MIB1	0.172837424	0.0001026
TNIP2	0.172757349	0.000103374
ZNF182	-0.172729748	0.000103642
SV2A	0.172724901	0.000103689

CXCL2	-0.172602152	0.00010489
TNFSF10	-0.172562771	0.000105278
CYP4F3	0.172553813	0.000105367
ST3GAL1	-0.172497413	0.000105925
ITCH	0.17247137	0.000106184
PGF	0.172467844	0.000106219
GDF2	-0.17244803	0.000106417
LMO1	0.1724411	0.000106486
MTRNR2L4	-0.172372645	0.000107171
ICE2	-0.172104511	0.000109895
RTEL1	-0.172033319	0.000110629
TBCD	-0.171836599	0.000112681
MSH5	-0.171789588	0.000113177
SETX	-0.171745415	0.000113644
COL6A1	0.171621078	0.000114971
TBC1D9	-0.171553075	0.000115702
DNAJB1	0.171472765	0.000116571
GH1	-0.171202533	0.000119542
KLHL21	-0.171194667	0.00011963
NTRK2	-0.171073853	0.000120982
BMP1	0.170998537	0.000121832
FGFR4	-0.170913218	0.000122802
MICA	-0.170901201	0.000122939
LTBP2	-0.17078798	0.000124239
EGR3	-0.170734191	0.000124861
GTPBP6	-0.170539225	0.000127139
SYT2	-0.170291135	0.000130096
MOB1A	0.170247102	0.000130627
YEATS4	0.17012566	0.000132103
CD5L	-0.170120556	0.000132165
NLE1	0.17009141	0.000132522
NFX1	-0.169912845	0.000134728
GABARAP	-0.169765903	0.000136569
MAZ	0.169670364	0.000137778
TRAF6	-0.169639083	0.000138176
ANXA2	0.16953084	0.000139562
HADHA	0.169499013	0.000139973
SLC2A3	0.169454985	0.000140542
PARP1	0.169412388	0.000141094
NDUFA2	-0.169246462	0.000143267
DYNLT3	0.169233851	0.000143433
ZHX2	-0.169189622	0.000144018
BSCL2	-0.169182288	0.000144115

NFKBIZ	-0.169092961	0.000145305
LSM4	0.169026241	0.000146199
FAM174B	-0.169007441	0.000146452
DDRGK1	-0.16895191	0.000147201
GNG5	0.168849735	0.000148589
KRR1	0.168827835	0.000148888
ITSN2	-0.168714987	0.000150439
TMCC1	0.168559999	0.000152593
GUCY1B2	-0.168539057	0.000152886
WDR5	0.168493615	0.000153524
LRRC40	0.168227869	0.000157307
RNF216	0.168218858	0.000157436
RAPGEF2	-0.167867358	0.000162578
SHF	-0.167780656	0.00016387
PPIP5K2	-0.167728489	0.000164652
ALDH4A1	-0.167594636	0.000166675
SPINT2	0.167409122	0.000169517
NUMA1	-0.167277613	0.000171559
CIDEB	-0.167237717	0.000172183
CD86	-0.167140951	0.000173706
PJA2	-0.167075597	0.000174742
SULF2	0.167049262	0.00017516
XRCC6	0.166937787	0.000176944
ARHGEF28	-0.166575885	0.000182852
BRWD1	-0.166573494	0.000182892
AP1AR	0.166472151	0.00018458
PPP2CB	-0.166375134	0.000186209
TRPV3	0.166215142	0.000188925
BCAP29	0.166098089	0.000190936
RHEB	0.165915273	0.000194117
ARF3	0.165907582	0.000194252
POU2F2	-0.165808037	0.000196006
TNF	-0.165752753	0.000196986
NFIB	-0.165678626	0.000198308
HNRNPA2B1	0.165622805	0.000199309
WDR4	0.16561158	0.000199511
PTP4A3	-0.165357504	0.000204131
ARHGAP4	-0.165332304	0.000204595
G0S2	0.165273621	0.000205678
NME3	-0.165125456	0.000208438
WWOX	-0.164844673	0.000213763
PDGFB	0.16480586	0.000214509
CCNE2	0.164786128	0.000214889

BIK	0.164784327	0.000214924
GLDC	0.164700895	0.000216539
MGST2	-0.164513527	0.000220207
DYRK3	-0.164494734	0.000220578
PARP4	-0.164415346	0.000222152
RPL3	-0.16436883	0.00022308
RNF13	-0.164367744	0.000223101
ABCG1	-0.164239947	0.000225668
HSPBP1	0.164078735	0.000228944
ETAA1	-0.164033269	0.000229877
OSR2	0.1639217	0.000232179
GALK1	0.163641371	0.00023806
TNFSF11	0.163504232	0.000240987
FBXO28	0.163468372	0.000241758
SPHK2	-0.163295742	0.000245502
CD8B	-0.1632493	0.000246519
HSPA5	0.163223282	0.00024709
RNF157	0.163214697	0.000247279
SLC39A6	0.163199365	0.000247616
THBD	-0.163136949	0.000248994
ASAH2	0.16293007	0.000253613
SOX30	0.162901442	0.000254258
EIF2AK2	0.162881	0.00025472
MID1	0.162808364	0.000256367
GADD45A	0.16270259	0.000258784
BNIP2	-0.16251998	0.000263006
RAG1	0.162517406	0.000263066
APIP	-0.162476396	0.000264024
MRPS22	0.162473431	0.000264093
UBFD1	0.162465522	0.000264278
CA2	-0.162108655	0.000272754
MAP3K15	-0.162105117	0.000272839
POU4F1	0.162075051	0.000273565
COX6A1	0.162048921	0.000274197
GAS5	-0.161971198	0.000276086
NMB	0.161911721	0.00027754
PPP2R5C	-0.161868729	0.000278595
PCYT1B	-0.161865895	0.000278664
PTGS2	0.161837126	0.000279373
SFN	0.161716309	0.000282366
GPALPP1	-0.161698899	0.000282799
ACVR1	0.16163733	0.000284338
CALM3	0.161555362	0.000286399

CREBZF	-0.161498653	0.000287833
AKAP11	-0.161166661	0.000296364
TRAF5	-0.161149595	0.000296809
PDLIM1	-0.161089832	0.000298371
GCHFR	-0.160959729	0.0003018
CASP3	0.160663255	0.00030975
SON	-0.160618147	0.000310977
NR1D2	-0.160598933	0.000311501
ERF	0.160177372	0.000323204
LIG1	0.160177251	0.000323208
PPP2R2B	-0.160105926	0.000325228
LTBP1	0.160087421	0.000325754
PI4KB	-0.159873654	0.000331889
MALT1	-0.159810876	0.000333711
MXD1	0.159727482	0.000336146
DEPTOR	-0.159616478	0.000339413
CASP14	0.159575005	0.000340641
MATN2	-0.159472985	0.000343679
VDAC3	0.159359928	0.000347076
MZB1	-0.159325095	0.000348129
LTBP3	-0.159193665	0.000352129
TAOK1	0.159159158	0.000353186
FGF18	-0.159114685	0.000354553
LAMB2	-0.159093824	0.000355196
TSC22D1	-0.159059861	0.000356245
PARD6G	0.159054365	0.000356415
CCDC85B	0.15904108	0.000356826
SPTBN2	0.159013418	0.000357684
AGGF1	-0.158956128	0.000359466
CAMK2G	-0.158588442	0.000371105
SASH1	-0.158562948	0.000371925
ERAP1	-0.158535538	0.000372809
LIMD2	-0.158517	0.000373407
LY75	-0.15843831	0.000375958
TGFA	0.158427278	0.000376317
SSTR2	0.158416041	0.000376683
RABGGTA	-0.158409836	0.000376885
DNAJB14	-0.158365961	0.000378318
CYBA	-0.158347881	0.000378909
TIMM22	0.158277111	0.000381234
PTK7	-0.15820237	0.000383704
PARP2	0.158102915	0.000387013
S1PR3	0.15800525	0.000390289

MAP2K1	0.157734582	0.000399503
AKAP10	-0.157694143	0.000400897
BBC3	-0.157653136	0.000402315
CCR8	-0.1576426	0.00040268
CDKN1B	-0.157490502	0.000407985
TNFSF18	-0.157453665	0.00040928
FASLG	-0.157432468	0.000410026
RELB	0.157424137	0.00041032
IFT27	-0.157367783	0.000412313
LAGE3	0.157277607	0.00041552
PIGA	-0.15718138	0.000418968
TMEM14A	0.157104546	0.000421741
GEMIN2	0.156953583	0.000427237
CROT	-0.156877237	0.000430043
DNAJC7	0.156777105	0.000433748
CNDP2	-0.156750955	0.00043472
MYO6	-0.15664545	0.000438665
THBS4	-0.156488723	0.000444586
BCAR1	0.156286556	0.000452333
UPP1	0.15627168	0.000452908
CBX5	0.155706009	0.000475284
APEX1	0.155669638	0.000476757
TNFSF13B	-0.155584266	0.000480233
ETHE1	0.15558417	0.000480236
TFDP1	0.155583343	0.00048027
KCNMA1	-0.155487801	0.000484187
ZMAT3	-0.155400626	0.000487787
GGCT	0.155311549	0.000491492
ZNF654	-0.155160468	0.000497834
COL4A2	0.155101736	0.00050032
RTN3	0.155095907	0.000500568
ITPR3	-0.155036452	0.000503097
MOB4	0.154905878	0.000508695
PSMD13	0.154656391	0.000519551
HDAC3	-0.154488455	0.000526979
TUBB4A	0.15448539	0.000527115
TPT1	-0.154391564	0.000531309
S100A8	0.154367494	0.00053239
VLDLR	-0.154307086	0.000535112
MSMO1	0.154282065	0.000536243
AP1S2	-0.15412984	0.000543173
DUSP5	0.15403628	0.000547473
ZNF330	-0.154029832	0.000547771

CHUK	0.153992309	0.000549505
CAPNS1	0.153907904	0.000553425
TAF5	0.153602183	0.000567842
CDKN2A	0.153565729	0.000569584
STK11	-0.153532723	0.000571166
CLEC18A	-0.153449596	0.000575168
RCHY1	-0.153349487	0.000580021
KISS1R	0.153288186	0.000583012
SAFB2	-0.153234327	0.000585651
EIF3H	0.153158396	0.000589392
TRAF7	0.153030113	0.000595761
HAVCR2	-0.15297392	0.000598571
LYPLAL1	-0.152866853	0.000603959
HIPK1	-0.152453714	0.000625174
SIAH2	0.152082811	0.000644808
GOSR2	0.151976874	0.00065052
BLZF1	0.151974951	0.000650624
TAX1BP1	0.151968584	0.000650968
PENK	-0.151963059	0.000651268
GSE1	-0.151700849	0.000665623
SLC29A1	-0.151678038	0.000666886
ARID4B	-0.151619485	0.000670137
MAP2K7	-0.15150217	0.000676695
TRPM1	-0.151429778	0.000680771
LSM1	0.151378381	0.000683679
RBBP8	0.151324921	0.000686716
NF2	0.151300688	0.000688097
CSRNP2	0.151262406	0.000690283
NPPA	-0.151212292	0.000693155
PLSCR1	0.151202393	0.000693724
TMEM219	-0.15112806	0.000698007
SMPD1	-0.150998947	0.000705506
IFITM1	-0.15085907	0.000713714
CHMP2B	0.150835147	0.000715127
HUWE1	0.15081415	0.000716369
RNF186	0.150749037	0.000720233
SDAD1	0.150599094	0.000729205
RBM15B	0.15059792	0.000729275
HMGB3	0.150569332	0.000730998
CTTNBP2	-0.150487765	0.000735933
DDN	0.150339433	0.000744987
ZYX	0.150201774	0.000753482
ARHGAP10	0.149980367	0.000767333

ADCY2	-0.149949808	0.000769263
PPP1R13L	0.149938665	0.000769968
PARP6	-0.149921268	0.00077107
ANP32A	0.149722802	0.000783742
TP73	-0.149640107	0.000789079
MGAT4A	-0.149599449	0.000791715
NR4A1	-0.149422618	0.000803276
MAP4	0.149370341	0.000806724
MAPK8IP1	-0.149313753	0.000810471
DZIP1	0.149112165	0.000823952
ITGA6	0.149075267	0.000826441
C1QBP	0.148861974	0.000840971
RTP4	-0.148651563	0.000855535
GALNT1	0.148605481	0.000858755
FOXS1	-0.148376193	0.000874948
QSER1	0.148292425	0.000880933
DAB2IP	-0.148283385	0.000881582
GRK6	-0.148254182	0.000883678
CDK9	-0.148117577	0.000893549
STYXL1	0.147923612	0.00090774
NMI	0.147819372	0.000915452
PTN	-0.147613965	0.000930826
BCAS2	0.147415038	0.000945942
RAP2C	0.147386227	0.00094815
UNC5D	0.147353326	0.000950677
SERPINA1	-0.147269537	0.000957142
ZNF544	0.147082751	0.000971698
CAMLG	-0.147059233	0.000973546
RCN2	0.146932047	0.000983592
DSC1	0.146904979	0.000985742
HOXB5	0.146901173	0.000986045
DHX9	0.146896848	0.000986389
ANXA5	0.14672681	0.001000005
SIDT1	-0.146725867	0.001000081
ATP2A3	-0.146696932	0.001002416
CCAT2	-0.146652098	0.001006043
ARPC2	0.146619123	0.001008718
IL13	-0.146595633	0.001010628
MYEF2	-0.146566412	0.001013009
CLN3	-0.146281374	0.001036502
TUBB2A	0.146111108	0.001050774
USP12	-0.146049882	0.00105595
ZBED1	0.146008154	0.001059492

GFOD1	-0.145938257	0.001065448
FGF11	0.145897264	0.001068956
EIF3L	-0.145868523	0.001071422
ZNF24	-0.145759962	0.001080782
CHMP7	-0.145457054	0.001107301
FAT1	0.145319209	0.001119567
TBC1D1	0.145252055	0.001125588
GPC1	0.145182563	0.001131849
TSFM	0.145173928	0.00113263
EVA1A	-0.145165398	0.001133401
PCOLCE	0.144957852	0.001152318
MAGEA4	0.144857858	0.001161535
ACKR3	0.144784627	0.001168328
POLD1	0.144579094	0.001187591
PHTF2	0.144485314	0.001196477
PARP14	-0.144198881	0.001223998
RNF126	0.144066091	0.001236954
CXCL12	-0.144050495	0.001238483
NEK1	-0.143939311	0.00124944
DSG3	0.143479015	0.001295758
EFHD2	0.143410608	0.001302774
USP8	-0.143393864	0.001304497
ZFX	-0.14328611	0.001315634
DUSP8	-0.142981142	0.001347631
SLMAP	0.142845552	0.001362085
HOXB6	0.142727076	0.001374832
RBMS1	0.142678457	0.001380094
P3H3	0.142649161	0.001383274
IFNGR1	-0.142648022	0.001383398
PRKACG	-0.142486186	0.001401087
PDCD2	0.142460229	0.001403944
PPM1D	-0.142334854	0.001417817
PNPLA6	-0.142289424	0.001422874
TBK1	0.142277825	0.001424169
GSK3B	0.142234352	0.001429028
MAPRE2	-0.142223115	0.001430287
SOS2	-0.142147583	0.001438773
MKNK2	-0.142130884	0.001440655
MFHAS1	-0.142116252	0.001442307
WDR45	-0.142112396	0.001442742
SUCO	0.141923648	0.001464206
LGALS3BP	-0.141858168	0.00147172
STOX2	-0.141766523	0.001482296

MAVS	-0.141741319	0.001485217
SYNE2	-0.141714594	0.00148832
ICAM1	-0.141654853	0.001495278
REEP2	0.141623248	0.001498971
DUSP13	0.141611415	0.001500356
EFNA1	-0.141547728	0.00150783
MRPS31	-0.141289359	0.001538502
PTEN	-0.141126924	0.001558077
MAP3K7	0.140932583	0.001581798
MAGEB1	0.140848503	0.001592162
EIF4G2	0.140698286	0.001610836
WIPI1	0.14068985	0.00161189
INHA	0.140522265	0.001632972
TGFBR1	0.140506809	0.001634929
CAPRIN2	-0.140468877	0.001639741
PGLYRP1	-0.140407205	0.001647592
EAF2	-0.140403482	0.001648068
OXSR1	0.140344456	0.001655616
MED1	0.140297707	0.001661618
REL	-0.140202447	0.001673908
CAPN6	-0.139973652	0.001703768
COL9A3	0.139916976	0.001711241
GFI1B	-0.139913217	0.001711737
TP53AIP1	-0.139853946	0.001719586
MRPL2	0.13985035	0.001720063
CD38	-0.139737929	0.001735043
TBC1D9B	-0.139717208	0.001737818
HINT2	-0.13969903	0.001740255
SLC24A3	-0.13966893	0.001744297
NRG3	-0.139585037	0.001755609
MAGED1	0.139583273	0.001755848
PHLDA1	0.139579811	0.001756316
TRIM21	-0.139478442	0.001770079
ACER2	-0.139398243	0.001781038
BCL2L1	0.139335567	0.001789646
TCEAL4	-0.139227606	0.001804562
MICU2	-0.139198023	0.001808669
CDC5L	0.139173228	0.001812118
USP9X	0.13914216	0.001816448
JDP2	-0.13912837	0.001818373
CAPN7	-0.13906323	0.001827492
TOR1AIP1	-0.138916993	0.001848116
LATS2	0.138907454	0.001849468

USE1	-0.138839288	0.001859161
PPP2R5E	0.138836116	0.001859613
KIDINS220	-0.138822104	0.001861611
MMP9	0.138763176	0.001870038
GTF2H5	0.138626016	0.001889787
MDK	0.138579175	0.001896575
DCT	-0.138471356	0.001912285
PARN	-0.138300407	0.001937437
GAB1	-0.138288721	0.001939167
LGALSL	-0.137965314	0.001987621
FOXP1	-0.137803294	0.002012309
PKN2	0.137790356	0.002014293
IRF5	-0.137699812	0.002028224
CSPG5	-0.13737518	0.002078901
CCL5	-0.137011491	0.002137049
NDUFAF4	0.136954787	0.002146248
THUMPD3	0.13684929	0.002163458
BAG1	-0.136537593	0.002215047
OCA2	-0.136525363	0.002217094
ZBTB5	-0.136260096	0.002261919
TRIM63	-0.136207525	0.0022709
TRIP6	-0.13618585	0.002274613
MYO9B	-0.136148754	0.002280979
MTMR6	-0.1360682	0.00229486
MYDGF	0.135988691	0.002308637
FYN	-0.135966575	0.002312482
CRADD	-0.135964469	0.002312849
MAP3K10	0.135881753	0.002327285
GPBP1	-0.13579874	0.002341856
ITGB4	0.135792719	0.002342916
EIF3D	0.135698011	0.002359648
RARG	0.135687225	0.00236156
PAK6	-0.135318019	0.002427884
BFAR	-0.135287094	0.002433516
CTH	0.135273063	0.002436075
PPFIA1	0.135202435	0.002448994
SBF1	-0.135172186	0.002454546
PPP3CB	-0.135086592	0.002470319
SETMAR	-0.13502702	0.002481351
HIVEP3	-0.134814333	0.002521107
WDR25	-0.134778202	0.002527918
EPCAM	0.134753844	0.002532519
TMSB4X	-0.13451235	0.002578552

CORT	-0.134470225	0.002586659
CAP2	0.134278621	0.00262383
CYP39A1	-0.134243408	0.002630714
PIK3C3	-0.134186998	0.002641776
NAA35	0.134135164	0.002651978
FASTKD3	-0.134094342	0.002660038
DSTYK	-0.13394353	0.002690008
TIMP1	0.133942367	0.002690241
AHCYL1	-0.13385947	0.002706847
CDK5	0.133796547	0.002719514
YWHAH	0.133698766	0.002739305
RAPGEF1	-0.13361208	0.00275696
SLC1A1	-0.133543064	0.002771091
COPS2	0.133527634	0.002774259
HDAC1	0.133391998	0.002802251
DDX47	-0.133240519	0.002833816
ATG2A	-0.133216375	0.002838877
RIMS3	-0.133175058	0.002847557
SYT13	0.133055176	0.002872878
CAPN13	-0.132892476	0.002907571
IFIT5	-0.132876219	0.002911059
BMX	-0.132736613	0.002941164
BAG3	0.132581915	0.002974854
TATDN1	0.132505054	0.002991722
CPT1C	0.132487694	0.002995545
QSOX2	0.132409192	0.003012883
PANO1	-0.132289313	0.003039538
PIK3C2G	-0.132260704	0.00304593
ZNF529	-0.13216688	0.003066981
NDRG1	0.132155267	0.003069596
STK3	0.132142164	0.003072548
FOXE3	0.1321316	0.003074931
STK16	-0.132101962	0.003081623
RERE	-0.132051993	0.003092938
PCK2	-0.132000827	0.003104562
CD44	-0.131980509	0.003109189
ABCA12	0.131953938	0.003115249
CD55	-0.131946998	0.003116834
OASL	0.131932221	0.00312021
BBX	0.131692882	0.003175365
HLA-A	-0.131492207	0.00322229
CCDC91	0.131488508	0.003223161
MAFK	0.131290467	0.003270099

ZNF430	-0.131185011	0.003295346
CDK17	0.131110943	0.003313184
MAFG	0.131099519	0.003315943
ANXA2P2	0.130988833	0.003342784
RAB21	0.130792577	0.00339086
NEK7	-0.13067763	0.003419309
MBOAT7	0.1306443	0.003427598
AGTPBP1	-0.130628499	0.003431534
TIMP4	0.130569653	0.003446229
PDCD1	-0.130496272	0.003464635
STK4	-0.13044708	0.003477023
SMG1	-0.130445173	0.003477504
YTHDC1	-0.130434424	0.003480216
MAPK11	-0.130403942	0.003487919
EIF3A	0.130303785	0.003513338
HSPH1	0.130120813	0.003560208
FLT4	-0.129835518	0.003634422
OSBPL1A	-0.129733977	0.003661173
PLCG1	-0.129731832	0.00366174
SHMT1	-0.129578216	0.003702555
FGF17	-0.129575439	0.003703296
MNAT1	0.129550349	0.003710003
FAF1	0.129418878	0.003745325
EPHB4	0.129412765	0.003746975
ORC3	0.129412703	0.003746991
VEZT	0.129325864	0.003770499
SCHIP1	-0.128766498	0.003925148
ZNF611	-0.128480444	0.004006434
METAP2	0.128349736	0.004044081
COPS3	0.128224001	0.004080597
PSEN1	0.12817451	0.004095052
DNAJC13	0.127941016	0.004163877
TNKS	-0.127928841	0.004167494
MUTYH	-0.127850253	0.004190911
BCL3	0.127815083	0.004201429
NR3C1	-0.127636773	0.004255124
AIMP1	0.127616604	0.004261236
NUAK2	-0.127612717	0.004262415
COTL1	-0.127599081	0.004266553
HSPB2	-0.127460335	0.004308865
GAS1	0.127382165	0.004332871
CORO2A	-0.127370646	0.004336418
THADA	-0.127321065	0.004351718

ZBTB20	-0.12731842	0.004352536
TCTN3	0.127305517	0.004356526
IGLL1	-0.127257914	0.004371276
GALC	-0.127216913	0.004384016
IFT122	-0.127064839	0.004431565
PLEKHH3	-0.126993622	0.004453992
DIO1	-0.126835937	0.004504014
F5	0.126655154	0.004561987
TRAF4	0.126595973	0.004581111
TCF4	-0.126588833	0.004583423
CTNS	-0.126183664	0.004716368
DSG1-AS1	0.126088077	0.004748235
PIK3CA	0.126054225	0.004759567
IRAK4	-0.126017361	0.004771935
POLR3C	0.125909708	0.00480822
GNB4	0.125906565	0.004809283
OGFR	-0.125788194	0.004849476
PRKAR1A	-0.12572751	0.004870198
UBB	-0.12551682	0.004942766
YBX3	0.125493736	0.004950776
BAZ1A	0.125489334	0.004952305
NFKB1	-0.125449398	0.004966193
PLXNB2	-0.125433426	0.004971757
USP17L2	-0.125375074	0.004992135
RGS16	-0.125185877	0.005058722
IKBKE	-0.124925551	0.005151652
PRKRA	0.124839374	0.005182753
HSPA8	0.124784543	0.005202629
TBCA	0.124735087	0.005220615
TRIM46	-0.124724139	0.005224604
GRAMD4	-0.124632007	0.005258284
GPR78	0.12433614	0.005367766
XRCC4	0.124040956	0.005479036
MPZL1	0.124000286	0.005494529
ZFP36L2	-0.123959168	0.005510232
DEDD	0.123867994	0.005545195
CCNC	0.123657988	0.005626489
PPM1A	-0.123633093	0.005636197
GZMA	-0.123588231	0.005653728
CALD1	0.123533274	0.005675272
SH2B2	0.123322212	0.005758698
BMPR1B	0.123217106	0.005800652
THAP1	0.123168699	0.005820067

TPD52	0.123159512	0.005823758
DAPK3	0.123117066	0.00584084
NOL3	-0.123062991	0.005862667
CIDEC	0.123003106	0.005886924
SCO2	-0.122934672	0.005914755
ARPP19	0.122845689	0.005951119
WDR13	-0.122842107	0.005952587
ZNF407	-0.122815014	0.005963701
PUS7L	0.122760095	0.005986288
SPINK2	-0.122596403	0.006054066
FTH1	0.122508352	0.006090809
CDH2	0.122319761	0.00617018
OSBPL8	0.122232334	0.006207289
SORL1	-0.122095598	0.006265729
SLTM	-0.121956362	0.006325745
FATE1	0.121814386	0.006387472
MYO10	0.121783972	0.006400765
SEMA3B	-0.121681275	0.006445836
LSM6	0.121619891	0.00647291
RIOK2	-0.121598479	0.006482379
JUN	-0.12149648	0.006527653
RBM26	-0.121132046	0.006691739
LGALS13	0.121001731	0.006751305
SORT1	-0.12095606	0.006772293
ETS2	-0.120946972	0.006776476
CIAPIN1	0.120868932	0.006812494
PRPS1	0.120762337	0.006861968
HEYL	-0.120706561	0.006887984
ARFGAP3	0.120509479	0.006980618
CPOX	0.120440641	0.007013235
DYNC1I1	0.120269944	0.007094707
CEP170	0.120181348	0.007137326
UACA	0.119936688	0.00725621
AK2	-0.119914246	0.007267203
C1GALT1	0.119846387	0.007300535
RTN4RL2	-0.119703295	0.007371269
PPARD	0.119693096	0.007376333
NRG2	-0.119630353	0.007407561
MAX	-0.119396415	0.007525037
DPM1	0.119288576	0.007579748
IL6	0.119255966	0.007596363
TMED7	0.119254029	0.00759735
MAP2K2	0.119222151	0.007613626

DPP8	-0.119034547	0.007710037
TRIM24	-0.118944857	0.007756514
SPRY1	-0.118550041	0.007964094
NGLY1	-0.118502191	0.007989586
CDC42BPA	-0.118472921	0.008005215
TCAIM	-0.118448389	0.008018336
BAG4	0.118386721	0.008051402
GNB2	0.118316162	0.008089386
LINC00222	-0.118308192	0.008093686
PPEF1	0.118236632	0.00813239
G3BP1	0.118013157	0.008254324
PTPRG	-0.117850281	0.008344218
MOSPD2	-0.117769611	0.008389063
IRAK2	0.117703463	0.008425997
S1PR2	-0.117402023	0.008596144
CPTP	0.117378725	0.008609421
PPM1L	-0.117229917	0.008694656
ACADM	-0.117225429	0.008697239
ULK1	0.11716943	0.008729517
SCOC	0.117141243	0.008745804
TNFSF9	0.117105213	0.008766663
ATP6V1E1	0.117081043	0.008780681
PEX13	0.116994479	0.008831048
FGF23	0.116952304	0.008855681
MAPK8IP2	0.116901936	0.008885179
DGKE	-0.116661974	0.009026918
IGFBP5	0.116522411	0.009110277
PKHD1	-0.116428442	0.009166789
MYL12A	0.116165863	0.009326357
GDF6	0.115967933	0.009448266
FLT1	0.115934385	0.009469069
C8orf33	0.115922067	0.009476717
ZNF93	0.115805108	0.009549614
ADAM19	0.115669258	0.00963491
JADE1	-0.115639879	0.009653445
CHP2	-0.115607365	0.009673995
AGPAT5	0.115599322	0.009679084
TAF9B	-0.115567819	0.009699041
RPS6KA4	0.115448787	0.009774779
NPTX1	-0.115152475	0.009965602
HSPB1	0.115129983	0.009980221
BCL2L11	0.114914869	0.010121001
STX7	-0.114877887	0.010145381

CWC27	0.114789005	0.010204188
MAF	-0.114450118	0.01043119
C1D	0.114414976	0.010454984
MOXD1	-0.114384177	0.010475878
COX17	0.114356171	0.010494908
GLG1	0.114137387	0.010644634
MICB	0.114029918	0.010718873
DOHH	0.114014172	0.010729789
IFNA5	-0.113872674	0.010828322
MAGEC1	0.113658455	0.010979023
CYP2A6	-0.113284707	0.011246403
RAP1B	0.11303143	0.011430859
LITAF	-0.113030671	0.011431416
GDF9	-0.112959577	0.011483673
PLXND1	-0.112910075	0.011520184
SLITRK5	0.112764969	0.011627802
LRRC14	-0.112665269	0.011702259
ADCY3	0.112658084	0.011707641
UNC5B	0.112623646	0.011733469
USP10	0.112417748	0.011888936
CDH13	0.112386059	0.011913025
TMEM123	0.112381317	0.011916633
JAG1	0.11230686	0.011973417
SLC30A10	0.112241717	0.012023294
TTC36	-0.112052302	0.012169364
DUSP4	0.111957573	0.012243002
EPB41L2	-0.111864344	0.012315858
PPIB	0.111844026	0.012331787
FOXO3	-0.111644319	0.012489322
TERT	0.111533899	0.012577185
ING2	0.111529058	0.01258105
OSBPL10	0.111320253	0.012748738
CASP6	0.111262319	0.012795612
EGR1	-0.111260651	0.012796964
AKT2	0.111208754	0.012839086
ACOT2	-0.110971679	0.01303307
ALG12	-0.110861991	0.013123691
APLP1	0.110853817	0.013130466
SH3D21	-0.110821606	0.013157196
SIGLEC15	0.110781522	0.013190525
MAEA	0.110662317	0.013290084
RBM27	0.110616975	0.013328126
DDAH2	0.110609986	0.013333999

GRB2	0.110204734	0.013678429
THBS1	0.110188258	0.013692596
GNB3	-0.110152088	0.013723744
RNF185	-0.110125193	0.013746945
STAT3	-0.110120652	0.013750865
SUPV3L1	0.110084454	0.013782153
HTRA2	0.110078593	0.013787226
IGF1R	0.109990749	0.013863439
MX2	-0.109984922	0.013868509
BAG5	0.109941573	0.013906267
PDGFRL	0.109918264	0.013926607
ZKSCAN4	-0.109902944	0.013939991
TEAD2	0.109793093	0.014036286
SNX13	-0.109610113	0.014197986
C11orf80	0.109608539	0.014199384
IGF2	0.109401339	0.014384479
ZCCHC2	-0.109244919	0.014525614
PAGR1	-0.109121184	0.014638118
SYNGR3	0.10907548	0.014679868
PCSK5	-0.109066124	0.014688427
AEBP1	0.10906276	0.014691506
ETS1	-0.109043808	0.01470886
ZFP36L1	-0.108992779	0.014755678
SLC27A5	-0.108970153	0.01477648
DPAGT1	0.108939052	0.014805113
BCOR	-0.108838725	0.014897815
WASL	0.108541367	0.015175574
BLCAP	-0.10843944	0.015271824
PIK3C2A	-0.108387097	0.01532146
ADAMTSL4	-0.108276331	0.015426964
ACIN1	-0.10825914	0.015443395
HTATSF1	0.108107878	0.015588636
WBP11P1	0.107953804	0.015737807
ALKBH7	-0.107940285	0.015750956
WRAP73	-0.107938785	0.015752415
IGKC	-0.107935595	0.015755519
SLC5A11	0.107884514	0.015805299
PROM1	-0.107702454	0.015983847
PCYT1A	0.107645325	0.016040237
KIF26B	0.107602503	0.016082619
PDK1	0.107586328	0.016098654
PSMA3	0.107580487	0.016104447
STAT1	0.107575761	0.016109137

DYRK2	0.107494953	0.016189498
APP	0.10734257	0.016341995
PCDH11Y	-0.107163441	0.016522867
TRIM8	-0.10710077	0.01658656
PCBD1	-0.106771216	0.016925039
NOL6	0.106695996	0.017003138
TAF9	0.106641938	0.01705946
TAF1B	0.106544836	0.017161037
BIRC2	0.106439116	0.017272231
FGF8	0.106432664	0.017279039
BDNF	0.106429287	0.017282601
PLK3	-0.105940641	0.017805049
CFAP36	-0.105781077	0.017978612
IFITM2	-0.105663169	0.018107813
NDRG3	0.105502671	0.018284985
SH3GL3	-0.105427644	0.018368325
TOP2B	-0.105366816	0.018436135
KDR	-0.105277181	0.018536458
CIB1	0.105151609	0.018677802
DSG1	0.105087416	0.018750421
MLH1	-0.105051373	0.018791303
GULP1	0.105025607	0.018820575
PREP	0.104895139	0.01896941
ZNF629	-0.104861894	0.019007498
PLG	-0.104800757	0.019077717
SH3KBP1	0.104726504	0.019163305
S1PR5	0.104625373	0.019280411
WTAP	0.104587596	0.019324314
HPCAL1	-0.104544444	0.019374571
PCDHGC3	-0.104475577	0.019455015
ACVR1C	0.10439557	0.019548834
CTNNB1	0.10436696	0.01958248
CCAR2	-0.104270681	0.019696073
HSPA4L	0.104232465	0.01974132
LIPF	-0.104158371	0.019829304
BCAS3	-0.104100363	0.019898425
PSMD10	0.104043349	0.019966567
SLK	0.103944286	0.020085445
PAPSS2	-0.103704358	0.020375922
TP53	-0.103693151	0.02038958
KNG1	0.103662908	0.020426474
FAIM	-0.10358114	0.020526516
IGF2R	0.103579493	0.020528535

MRPS33	0.103534163	0.020584184
MTMR9	-0.103316258	0.020853525
RINT1	0.103309086	0.020862442
HEXIM1	0.103183903	0.021018613
GTF3C1	0.103179725	0.021023842
POLR2L	-0.103161125	0.021047139
MFSD10	-0.103064438	0.021168596
RABGGTB	-0.103050366	0.021186324
ZBTB38	0.103022294	0.021221728
ENO2	0.10297199	0.021285299
FAHD2A	-0.102953524	0.021308676
AKT1	-0.102920325	0.021350761
EXOSC9	0.102833745	0.021460856
TRAPPC8	-0.102816256	0.021483154
SRF	0.102701715	0.021629692
PRKX	-0.102680369	0.021657096
GEM	-0.102664637	0.021677313
RAB3GAP1	0.102657133	0.021686962
ITSN1	0.102621814	0.021732426
PDCD4-AS1	-0.102330932	0.022110017
MYLK	-0.10228913	0.022164745
NLRP10	0.102188069	0.02229754
CREB1	-0.102179301	0.022309094
IFNW1	-0.102149953	0.022347805
PDGFC	0.101967951	0.022589167
SUDS3	0.101954361	0.02260728
NET1	0.101913447	0.022661887
FAM168A	0.101897445	0.022683274
AKAP6	-0.101834297	0.02276785
DFFA	0.101820572	0.022786268
DSG4	0.101807958	0.022803206
VPS16	-0.10177589	0.022846317
PLSCR2	0.101733968	0.022902782
PPP6R1	0.101617409	0.023060413
ARHGEF10	-0.101531806	0.023176776
CMIP	0.101529574	0.023179818
EYA4	0.1015246	0.023186595
SLC16A6	-0.10150803	0.023209187
CD14	-0.101362644	0.023408221
SEMA3F	0.101270813	0.023534698
ZFP91	0.101020821	0.023882007
CCR9	-0.10101309	0.023892818
PRKD3	0.101003974	0.023905571

MSRB1	0.100862585	0.024104124
RWDD2B	-0.100810466	0.024177674
F2RL1	0.100584899	0.024498233
PPIL4	0.100562632	0.024530076
HSPA1L	-0.100419907	0.024735026
TTC5	0.100351643	0.024833574
PSME1	-0.100347181	0.024840027
CECR2	-0.100043047	0.025283314
CYB5R2	0.100005484	0.025338533
MT-CO1	-0.09992702	0.025454216
GCG	0.099892365	0.025505455
RNF122	-0.099857374	0.02555728
C1S	-0.099772552	0.025683288
NAT8	-0.099750188	0.0257166
FAM3B	-0.099668997	0.02583785
PPA2	-0.099433433	0.026192433
SMPDL3A	-0.099048237	0.026781286
MKRN1	-0.098993367	0.026866085
ZNF195	-0.098942212	0.026945353
BAZ1B	0.098899057	0.027012381
NUP98	0.098540234	0.027575275
PIR	0.098392559	0.02780985
SIKE1	0.098333923	0.027903465
WDR35	-0.098328315	0.027912432
AEN	0.098324254	0.027918927
AREL1	0.098153002	0.028194021
GLP2R	0.098069728	0.028328631
TJP2	-0.09799231	0.028454269
SRD5A3	0.09799173	0.028455212
NSFL1C	0.097949738	0.02852356
SERPINB3	0.097948919	0.028524895
DHX29	-0.097946598	0.028528677
EPB41L1	-0.097899414	0.028605656
RELL2	-0.097883978	0.028630878
CCDC178	-0.0978696	0.028654389
BAD	-0.09785212	0.028682994
SHISA5	0.097789438	0.028785771
RWDD3	-0.097345482	0.029522799
PINK1	-0.097334211	0.029541719
LGALS16	0.097267343	0.029654181
MAP3K5	-0.097162725	0.029830868
NUAK1	0.097063481	0.029999314
TOM1	-0.097048793	0.030024314

ABHD3	0.096960943	0.030174207
PSMD6	0.096895532	0.030286229
FOXA1	0.096802546	0.030446091
CUL1	0.096718974	0.030590382
MUL1	0.096345778	0.031241897
SEC23IP	0.096332055	0.031266078
KRIT1	-0.09632292	0.031282184
BCL2L2-PABPN1	0.096288214	0.031343439
DYNC1H1	0.096233846	0.031439604
NUCB2	-0.096089494	0.031696153
PDE4D	-0.096070847	0.031729423
ADD3	-0.096055531	0.031756773
EPB41L3	-0.095932544	0.03197712
CD40	-0.095838919	0.032145737
MTOR	0.095812245	0.032193916
PRL	0.095799601	0.032216775
KTN1	0.095749355	0.032307751
AGRN	0.095665184	0.032460643
UGCG	-0.095640637	0.03250535
FANCF	0.095555291	0.03266119
RBMS2	-0.09549298	0.032775372
NCEH1	0.09539928	0.032947713
ESRRG	-0.095372498	0.032997115
FAF2	0.095344939	0.033048016
PCSK1	0.095227264	0.033266114
ENDOD1	-0.095018476	0.033656105
SUB1	0.095000936	0.033689044
DUT	0.094929366	0.033823735
HSPB3	-0.094893165	0.033892038
GM2A	-0.094649296	0.034355233
HNRNPA1	0.094604089	0.034441686
NOC3L	0.094486557	0.034667322
MTRNR2L8	-0.094345699	0.034939397
IGF2-AS	0.094334948	0.034960236
LINC00467	-0.094307821	0.035012868
MAPK13	-0.094301691	0.035024773
NAT8B	-0.094286515	0.035054254
TXNDC5	-0.09413115	0.0353573
KMT2A	-0.094127918	0.035363628
CSPP1	-0.094085732	0.03544631
FRA10AC1	-0.094000525	0.035613812
CCDC8	-0.093978274	0.035657664
IFNA6	-0.093968124	0.035677683

RAB5A	0.093906144	0.035800133
IDH1	0.093826367	0.035958271
IGLC2	-0.093791802	0.036026972
ATP2B4	-0.09374273	0.036124697
SOD2	0.093509677	0.036591896
SATB2	0.093447315	0.036717781
RET	-0.093353266	0.036908323
MAPT	-0.093310042	0.036996175
BST2	-0.09324496	0.03712879
GTF2A1	0.093007768	0.037615521
RNFT1	-0.092998808	0.037634014
LARP7	-0.09287973	0.037880505
S100A4	-0.092806146	0.038033509
CALM1	-0.092774416	0.038099645
DBNL	-0.092620286	0.038422301
NUP210	0.092578956	0.038509213
CD58	-0.092463179	0.038753567
NOX5	0.092346668	0.039000795
L1TD1	-0.09232793	0.039040679
PAX3	0.092126733	0.039471123
CHD8	0.092053917	0.039627892
PMPCA	-0.091832442	0.04010796
GSTO1	0.091798021	0.04018301
GLS	-0.09144611	0.040957144
COL4A6	0.091372395	0.041120888
PSME2	0.091337215	0.041199228
BHLHE40	0.091174017	0.041564296
RSRC2	-0.091168147	0.041577479
BNIP3	0.09115098	0.04161605
PLEKHF2	-0.090900639	0.042181952
ATP1B1	-0.090764115	0.042493293
PBRM1	-0.090753107	0.042518481
BHLHE41	-0.090688675	0.042666164
CARD11	-0.090547964	0.042990185
RARA	-0.090538564	0.043011904
CCAR1	0.09050796	0.043082683
FOXB1	0.090466152	0.043179529
GPER1	-0.090451651	0.043213164
TNFRSF4	-0.090338854	0.043475544
NPM1	0.090253126	0.043675852
SEMA5A	-0.090238704	0.043709626
TGFB1	-0.090228793	0.043732848
FMOD	-0.090029325	0.044202444

SMAD5	-0.089739758	0.044891699
SP1	0.089737084	0.044898106
IKBKG	-0.08952196	0.045416057
ARHGAP5	0.089487994	0.045498294
DECR2	-0.08946946	0.045543221
DYRK1B	-0.089464501	0.045555248
FGF7	-0.089430783	0.045637093
ACHE	-0.089419529	0.045664441
CEACAM5	0.089394936	0.045724243
BCL2L15	-0.089336439	0.04586676
ACOT13	0.089326496	0.04589102
AOC1	-0.089224075	0.046141554
UFL1	-0.089096284	0.046455759
OCEL1	-0.089057971	0.04655031
DCAF8L1	0.088920597	0.046890654
USP13	-0.08887745	0.046997982
DNASE1	-0.088809679	0.047166976
PHF20L1	0.088805965	0.047176253
PPBP	0.088363638	0.048291987
TSC22D4	-0.088328294	0.048382087
PTPRF	0.088143493	0.048855473
POLG	-0.08813908	0.048866825
IFNL2	0.088118841	0.048918914
TRPM4	-0.088030693	0.049146323
PRKD1	-0.087666369	0.050095624
AHNAK	-0.087600051	0.050270064
CHD4	0.087407361	0.050779792
RNF19B	-0.087219714	0.051280318
ABHD10	0.087010299	0.051843765
CEP192	-0.086988343	0.051903135
ENOX2	0.086869078	0.052226638
LEFTY1	0.086791896	0.052436883
NRG4	0.086680248	0.052742267
TXN2	-0.086652308	0.05281892
CELF1	-0.086582349	0.053011261
SPON1	-0.08656121	0.053069494
PYCARD	-0.086469759	0.053322032
MAPK1	0.086326444	0.053719805
UBAP2L	0.086258242	0.053909965
ZNF274	-0.086246546	0.053942632
IFNA1	0.08616811	0.05416213
PIK3R3	-0.086039852	0.054522655
RNPS1	0.085961964	0.054742563

ACYP1	-0.085949216	0.054778626
UBE2I	0.085819456	0.05514683
CTSA	0.085687284	0.05552399
IFNA13	0.085393379	0.05637035
ORAI3	-0.085342075	0.056519182
MAP2K6	0.085281891	0.05669419
RAF1	-0.085253296	0.056777501
TBPL1	0.085141463	0.05710429
CDCA7	0.08508158	0.057279917
SMARCA1	0.08502414	0.057448798
AREG	0.084978944	0.05758197
IFNA21	-0.084977939	0.057584934
BRCC3	0.084942835	0.057688551
BACH2	0.08479383	0.058130092
CASP9	0.084757873	0.05823706
FOCAD	-0.084579977	0.058768683
MYC	0.084507345	0.058986888
DNPH1	-0.084424409	0.059236868
MYCNOS	0.084413117	0.059270971
CRELD2	-0.084395372	0.059324597
COG6	0.08437984	0.059371567
CAPZA2	0.084366822	0.059410958
GTF2I	-0.084262556	0.059727239
SELE	-0.084177312	0.059986852
KLHL14	-0.084076742	0.060294342
ZBTB11	-0.084066791	0.060324838
CDC37	0.084004002	0.06051755
RNU6-547P	-0.083935554	0.060728216
C12orf4	0.083904684	0.060823422
RFC1	0.083873474	0.060919802
ANKMY2	-0.083823922	0.061073082
SPON2	0.083761522	0.061266562
GNAI2	-0.083667121	0.061560225
SREBF1	-0.083580903	0.061829449
MORF4L1	0.08357562	0.061845976
TRIM17	-0.083565155	0.061878726
TAOK3	-0.083496439	0.062094135
BIRC8	0.083433431	0.06229219
ZNF486	-0.083416075	0.062346839
POLR3E	-0.083379963	0.062460668
ALDH7A1	-0.083356269	0.06253545
LIMK2	-0.083289544	0.062746435
OGDH	0.083100541	0.063347255

HRAS	0.082940348	0.063860193
NRP2	0.08292893	0.063896882
ANAPC10	0.08283787	0.064190116
CELSR2	-0.082828903	0.064219052
SPATA2	0.082728436	0.064543986
GYS1	0.082679078	0.064704114
CBR1	-0.08262093	0.06489318
ITGB3BP	0.082612106	0.064921909
CASP8AP2	0.082498592	0.065292438
LAYN	0.082497413	0.065296294
DNAJA4	-0.082404849	0.065599739
MED13L	-0.082249632	0.066111175
COMP	0.0822313	0.066171794
PFKFB3	-0.082118055	0.066547279
PTK6	-0.082001863	0.066934354
SDHB	0.081640411	0.068150294
SPESP1	0.081513554	0.068581316
PDCD6	0.081378591	0.069042332
RELL1	0.081211401	0.069616945
EP400	0.081171036	0.06975626
EMC7	0.081147036	0.0698392
GOLGB1	-0.080985029	0.070401191
ZNF84	-0.080872819	0.070792606
CEP250	0.080807494	0.071021292
SLC22A17	-0.080750923	0.07121982
LXN	-0.080678768	0.071473696
SIRT1	-0.080592121	0.071779536
BAK1	0.080576278	0.071835576
EPHA2	0.080548638	0.071933425
SCAF11	-0.08052484	0.07201776
TYRO3	0.080419248	0.07239293
RYBP	0.080378178	0.072539285
SLC38A10	-0.080221285	0.073100596
CASP7	-0.080149724	0.073357792
ATF3	-0.080110482	0.073499145
EIF5AL1	0.08007602	0.073623461
OR5B12	-0.080022316	0.073817533
DKKL1	0.080014868	0.073844482
SYCP2	0.079860955	0.07440316
TRMT12	0.07971697	0.074928912
CHMP1A	0.079581276	0.075427156
STK38	0.079394612	0.076116955
STX3	-0.079369814	0.076208981

GZMB	0.07932777	0.076365209
NSMCE2	0.079295355	0.076485835
USP25	-0.079259176	0.07662065
DENND5A	-0.079189264	0.076881719
RAD50	-0.079133658	0.077089877
NDUFB6	0.079090186	0.077252933
KCTD20	0.079030862	0.077475897
EEA1	-0.078967387	0.077715042
BCLAF1	-0.078884118	0.07802967
PHLDA3	-0.078739938	0.078576894
SLC37A4	-0.078706795	0.078703124
EEF1D	-0.078690711	0.078764444
NAIF1	0.078686533	0.078780375
RAI14	0.078629113	0.078999634
VSIG10L	0.078522228	0.079409089
SAV1	0.07841198	0.079833228
RNF217	0.078401635	0.079873119
CADPS	-0.078270001	0.080382144
NDRG4	0.078191102	0.080688498
SCRIB	0.078109644	0.081005784
SMAD1	0.078048813	0.081243379
TSPO	-0.078044799	0.081259079
LMNA	0.078006781	0.081407881
MSRB2	0.077924576	0.081730384
BRSK1	0.077824949	0.082122625
FEM1B	0.07781326	0.082168745
SYNM	-0.077720272	0.082536384
ZCCHC8	0.077630189	0.082893802
TUSC2	-0.077517375	0.083343177
RSF1	0.077477773	0.08350139
MET	0.077474806	0.08351325
GLB1	-0.077429114	0.083696116
ALG6	0.077407732	0.083781799
FBXL7	-0.07737626	0.083908049
DST	0.077042668	0.08525568
RBM17	0.07694313	0.08566115
CD9	-0.076880312	0.08591784
FIS1	-0.076817243	0.086176178
VCX3A	0.076807494	0.086216167
CARD6	-0.076680205	0.086739661
ATXN1	-0.07652442	0.087383825
CDKN1A	0.07630596	0.088293628
MXI1	-0.076284573	0.088383104

VCX	0.07628394	0.08838575
LHPP	-0.076271523	0.088437734
LHB	0.07620344	0.088723198
ZYG11B	-0.076080915	0.089238795
HSPA6	0.076057935	0.089335765
PIK3R4	0.076027404	0.089464729
IFI35	0.07598699	0.089635666
PON2	0.075958329	0.089757056
SYVN1	-0.075869799	0.090132833
MEIS2	-0.075796834	0.090443497
OSGIN1	0.075583397	0.091357167
MDC1	0.075493233	0.091745357
PROC	-0.075472856	0.09183327
SPINK1	-0.075470283	0.091844374
RPS6KB2	0.075469157	0.091849236
SUCLG2	-0.075257475	0.092766621
NFE2L2	-0.07525006	0.09279889
CCDC112	-0.075155557	0.093210922
SCIN	-0.075115898	0.09338427
ARHGAP35	-0.0751138	0.093393448
TADA2A	-0.075034007	0.093743032
PDGFA	-0.074537673	0.095941085
DNAJB9	-0.074386996	0.096616446
NDUFB1	-0.074376583	0.096663256
HAS2	0.074347579	0.096793745
SP100	-0.074346572	0.096798277
NAP1L3	-0.074155264	0.097662506
SMARCD2	0.074122199	0.097812494
TRIM44	-0.074051563	0.098133534
TP63	-0.073856318	0.099025279
DNAJC8	0.073701076	0.099738914
FPGT	-0.07360435	0.100185616
TFAP2B	-0.073596744	0.100220809
STK25	0.073535771	0.100503295
IL19	-0.073478231	0.100770453
PEX1	-0.073055638	0.102749876
KMO	-0.073053208	0.102761345
RPL8	0.072981768	0.103099027
WFS1	-0.072938566	0.103303663
BATF3	0.072762631	0.104140332
ULK4P3	0.072753108	0.104185769
IL15	-0.072747665	0.10421175
CRABP2	0.072705182	0.104414692

ME1	0.072648354	0.10468665
LRP4	0.072606863	0.104885568
FGFR1	0.072567843	0.105072909
GNAS	0.072454878	0.105616765
HIVEP1	-0.072417351	0.105797927
MITF	-0.07239092	0.105925671
SRC	0.072259687	0.106561736
CCDC47	0.07208943	0.107391439
FGF20	-0.072034153	0.107661909
INHBE	0.071755885	0.10903166
XKR8	-0.071711267	0.109252559
CTTN	0.071664173	0.109486101
TNFRSF11A	-0.071500012	0.110303262
ARG2	0.071416741	0.110719602
MBTPS1	-0.07115894	0.112016405
BCAP31	0.071093371	0.112348131
GAS2	0.071009904	0.112771525
SFRP1	0.070934573	0.113154727
ABCC10	-0.07075098	0.11409292
GDF5	-0.070405608	0.115874371
MAP3K4	-0.07038906	0.115960269
UXT	0.070189258	0.117001378
LY96	-0.070093526	0.117502791
ACTA2	0.070068984	0.117631607
PEG3	-0.069973872	0.118131864
JUND	-0.069889049	0.118579411
ARMCX3	-0.069846508	0.118804366
ZNF382	-0.069820721	0.118940888
KDM5D	-0.06977875	0.119163354
RIPK1	-0.069602418	0.120101552
BARX1	0.069602388	0.120101711
ADD1	-0.069206977	0.122226508
NACC2	-0.069059614	0.123025835
SERPINB1	-0.069010057	0.123295556
FZD5	-0.06897235	0.123501088
ZNF638	-0.068866776	0.124077971
MYCN	0.068727592	0.124841704
FBXO10	0.068545678	0.125845415
VSNL1	0.068467589	0.126278184
PPM1F	-0.06836985	0.126821486
OSER1	0.068297987	0.127222104
NPY1R	0.068266982	0.127395258
ID3	0.068166032	0.12796028

MAPK9	-0.067862554	0.129670579
TRAPPC12	-0.067823181	0.129893762
MEN1	0.067736101	0.130388427
DNAL4	-0.067718203	0.130490276
CD70	0.067624341	0.131025421
STK26	0.067488485	0.131802986
PDP1	0.067460841	0.131961646
USP17L3	0.067175952	0.133605293
ZNF22	-0.066807659	0.135753492
FKBP8	-0.066796112	0.135821272
PLEKHF1	-0.06678876	0.135864442
EIF3K	0.066781695	0.135905935
SOD1	0.066742276	0.136137629
ATF2	0.066720678	0.136264699
MGAT2	0.066648228	0.136691636
NR4A2	-0.066516017	0.137473388
MBD4	-0.066427368	0.137999477
FNTA	0.066413213	0.138083624
CKMT1A	0.066370974	0.138334955
PARD6A	-0.066362606	0.13838479
PPP2R2A	-0.066271477	0.138928378
NADK	-0.066238185	0.139127372
OBSL1	-0.066174806	0.139506809
FGF1	0.066141559	0.13970617
BAG6	0.06603832	0.140326623
CCDC88A	0.066016012	0.140460963
RNMT	0.065958041	0.140810545
APOE	-0.065779349	0.141892295
FGF2	-0.065660103	0.142617708
USP17L10	-0.065567995	0.143179973
NLRX1	-0.065563173	0.143209456
PMS2	0.065551521	0.14328072
PI4K2B	-0.065522967	0.143455458
IMPDH1	0.065449734	0.143904375
PPFIA4	-0.065416083	0.144111012
PCGEM1	0.06538409	0.144307676
TRIP11	-0.065329383	0.144644445
HMGB1P5	0.06530327	0.144805408
HRK	0.065230161	0.145256775
TENM4	0.065072232	0.146235482
BMP8A	0.065020705	0.146555886
CHM	0.064994972	0.146716103
LAP3	-0.064912068	0.147233171

NAE1	0.064740161	0.148309767
IL2RA	-0.064509521	0.149763602
KCNIP3	-0.064446241	0.15016437
CDK7	0.064418535	0.150340101
KLF10	0.064271182	0.151277323
ARL11	-0.064237967	0.151489192
ABCG4	0.064087191	0.152453804
NSRP1	0.064035396	0.152786237
TAC1	0.063870087	0.153850926
EMP3	0.063618213	0.155483934
PTX3	0.06355001	0.155928379
RELT	-0.063539007	0.156000165
UNC50	-0.06343379	0.156687931
HINT1	-0.063425245	0.156743882
PTGIS	-0.063332631	0.157351321
PIM1	0.063327281	0.157386464
ABL1	0.063229894	0.15802724
RPS27A	0.063222946	0.158073025
METTL13	0.063057817	0.159164254
BCL7B	0.062928023	0.160025949
SMARCE1	0.062811692	0.160801252
COL25A1	-0.062721347	0.161405318
KIAA0930	0.062721106	0.161406936
MYOF	0.062652791	0.161864838
VAV3	0.062500659	0.162888065
KEAP1	-0.062420527	0.163428984
TMED4	0.062317204	0.164128432
PDCD7	-0.06231214	0.164162771
PSMB8	-0.062281693	0.164369345
BMP4	-0.062204368	0.16489485
HGSNAT	-0.062180221	0.165059214
IFNGR2	0.062096716	0.165628559
RRBP1	-0.061957916	0.166578163
EGF	0.061910367	0.166904413
EXOC1	0.061909286	0.166911829
MDM2	-0.061879105	0.167119166
DCLRE1C	-0.061875736	0.167142322
ISG20	-0.061869931	0.167182225
IRF1	-0.061809728	0.1675965
MAFF	0.061799974	0.167663688
MIR7846	-0.06179693	0.167684663
MAPK7	0.061778578	0.167811147
XIAP	-0.061770859	0.167864372

COL2A1	0.061681839	0.168479082
PACSIN3	0.06162675	0.168860327
PDCL	0.061605183	0.169009757
EIF3G	-0.061543736	0.169436053
DNAJC1	0.061497334	0.169758499
TCAF1	0.061455891	0.170046877
GADD45B	-0.061265092	0.171379249
SYPL1	0.06113904	0.172263757
CKMT1B	0.061004977	0.173208202
ENDOG	-0.060958099	0.173539357
IFNA2	0.060910445	0.173876476
TNIP1	-0.060907224	0.173899284
DCAF12L1	0.060902417	0.17393332
CAPN5	-0.060888855	0.174029371
KIF1B	0.060887849	0.174036498
POLR2A	0.060792618	0.174712119
HCAR2	0.060779977	0.174801944
COA3	-0.060706369	0.175325696
DCAF12L2	0.06065747	0.175674279
FABP5	-0.060611427	0.176002971
GBP1	0.060570133	0.176298148
VPREB1	-0.060513814	0.176701316
SNX16	0.060355352	0.177839386
TTR	-0.060265165	0.178489522
MAP4K5	0.060201352	0.178950608
HMGB1	0.060128053	0.179481319
TFDP2	-0.060090395	0.179754427
LRP5	0.060057001	0.179996872
NANOS3	0.060050057	0.180047314
HLA-G	-0.059895256	0.181174581
EGFR	0.059893787	0.181185305
IGFBP4	-0.059843158	0.18155514
GSR	0.059781029	0.182009736
PPIG	0.059746932	0.182259579
XKR4	-0.059742873	0.182289338
MIR3929	0.05974208	0.182295153
KDM5C	-0.059604494	0.183306036
ITFG1	-0.059369233	0.18504414
TP53BP2	0.059284797	0.185670909
CLEC11A	0.059186762	0.186400582
IFNA7	-0.059135405	0.186783677
IFIH1	0.059117384	0.186918242
HIF3A	-0.058952607	0.188151943

CFH	-0.058891952	0.188607579
RBBP6	-0.058819441	0.189153348
DUX4	-0.058700434	0.190051575
THUMPD2	0.058665148	0.190318509
ZNF184	-0.058636768	0.190533397
DAP	0.058499156	0.191577897
C1orf216	0.058424526	0.1921461
EPHA1	0.058357843	0.192654847
CARD10	0.058339022	0.19279862
ZZZ3	0.058044073	0.195061949
ACER3	0.05796492	0.195672639
DNAJB2	-0.057958007	0.195726039
NOSIP	0.057890021	0.196251785
CAPN2	-0.057810397	0.19686884
GSTM1	-0.057809022	0.19687951
HOXD4	0.057805519	0.196906694
PEX3	-0.057622906	0.198327421
TEAD3	0.0575909	0.198577194
IRS4	-0.057588688	0.19859447
ZSWIM2	-0.057254528	0.201216124
LYN	-0.057246875	0.201276457
PEMT	-0.05711668	0.202304912
NF1	0.056963057	0.203523339
PSEN2	-0.056865913	0.204296553
PROS1	-0.056822506	0.204642741
PLEKHO1	-0.056797784	0.204840094
FBLN1	-0.056785674	0.204936822
VPS41	-0.056757346	0.20516321
CDR1	0.05673298	0.205358084
STXBP3	-0.056624914	0.206223981
MAPK8	0.056544982	0.206866148
F2R	0.056421975	0.207857202
TMBIM6	0.056295616	0.208878829
PGDP1	0.056261759	0.209153189
CUL5	-0.056080011	0.210630398
TSR2	0.05603307	0.211013138
JAKMIP2	-0.055962202	0.21159193
OPTN	0.055818301	0.212770711
ZSCAN9	-0.055613737	0.214454555
TRPC1	0.055435625	0.215928438
GNG11	-0.055420756	0.216051803
CWF19L2	-0.055412848	0.216117442
MAP2K4	0.055352101	0.2166221

ZNF217	0.05524357	0.217525831
GNG3	-0.055183769	0.218024943
POU4F2	0.054938098	0.220083991
HTR1A	-0.054877349	0.220595277
CCDC134	-0.05487317	0.22063048
USP47	-0.054819811	0.221080334
SAMD9	0.054755753	0.22162124
PHAX	0.054512928	0.223680258
SGCB	0.054488272	0.223890084
IL9	-0.054456812	0.224158022
RAD51B	-0.054410164	0.224555729
GHRH	0.054374756	0.22485795
GTF2F1	0.05436099	0.224975524
SBNO2	0.054334726	0.225199961
PROK1	-0.054198294	0.226368407
MAG	-0.054192246	0.226420307
MIPEP	-0.05418054	0.226520779
FGF21	0.054156548	0.226726795
ASXL3	-0.054136185	0.226901757
PPP5C	0.054100616	0.227207599
COL16A1	-0.053975388	0.228286715
LAMB4	0.053893892	0.228990936
BDP1	-0.053728916	0.23042124
DCTN6	-0.053724472	0.230459851
CEP57	-0.053617711	0.231388925
TJP1	0.053605565	0.231494786
ACVR1B	-0.053583135	0.231690384
AGO1	-0.05352982	0.232155769
ZNF148	0.053495556	0.232455207
PODXL	-0.053460332	0.232763317
HOXA5	-0.053294863	0.23421458
FGF4	0.053194374	0.235099038
ZFAND6	-0.053173224	0.235285489
NIT1	-0.053171625	0.235299591
LGALS3	-0.053047435	0.236396572
IFI6	0.05299303	0.236878269
IRF6	-0.052619269	0.240206226
ASRGL1	-0.052519262	0.241102226
NECAP1	0.052452786	0.241699107
GRID2	0.05225506	0.2434806
PIM3	-0.052246306	0.243559684
C3orf38	0.05222983	0.243708578
INSIG1	-0.052111744	0.244777593

ESYT1	0.051953186	0.246218159
LIN37	0.051919828	0.246521977
EIF1AX	0.051897099	0.246729147
PLEKHB2	0.051859869	0.247068739
MOB3B	-0.051850147	0.247157478
UNC5C	-0.051820004	0.247432737
NEMF	-0.051755578	0.248021783
CLTB	0.051720135	0.248346252
PPP1R15A	-0.051712297	0.248418046
GRIN1	0.051676244	0.248748468
CEP170B	-0.051669921	0.248806453
TAP1	0.051660693	0.248891084
CEP290	-0.051591156	0.24952951
TAGLN2	-0.051561564	0.249801544
ZMYM1	0.051519887	0.250185023
UBD	-0.051484554	0.250510449
RHOB	-0.05148233	0.250530941
MAP1A	-0.051395768	0.251329488
HSBP1	-0.051338845	0.251855576
CRK	0.051335425	0.251887217
DICER1	-0.051232964	0.252836177
DAPL1	-0.05121205	0.25303018
LSR	0.051124271	0.253845574
RWDD1	0.051033392	0.254691685
EIF3F	-0.051032458	0.254700384
AXL	0.050950436	0.255465729
TUBA1A	-0.050904142	0.255898401
PDS5B	-0.050849181	0.256412725
ABCB6	0.05083654	0.256531127
EIF3CL	0.050516526	0.259541024
PRKG1	-0.050510782	0.259595271
ZNHIT1	0.0504767	0.259917306
MTRNR2L2	-0.050420069	0.260453019
TMF1	-0.050191202	0.262625769
TNFRSF8	-0.050169496	0.262832477
SLC25A6	-0.05011691	0.263333729
CXCL10	0.050103393	0.263462688
FAM32A	0.050080632	0.263679919
LIN52	0.049985894	0.264585453
GH2	0.049979047	0.264650981
RPP25L	-0.049897383	0.265433383
PKN1	0.049892585	0.26547941
RB1CC1	0.049835736	0.266025066

NQO2	0.049795799	0.266408866
RBMS1P1	-0.049755992	0.26679179
PSMD5	0.049743362	0.266913362
STX6	-0.049660683	0.267710145
RASA1	-0.04942038	0.270035204
AGPAT1	0.049323535	0.270976122
CCDC6	0.049268182	0.271514923
SLC25A11	-0.049252293	0.271669721
SLC6A2	0.049231105	0.27187623
MTRNR2L7	0.049192215	0.272255567
ZBTB6	-0.049008023	0.274057078
ENPP3	-0.048859654	0.275514104
BLID	0.048774674	0.276351011
EXOC3	-0.048631104	0.277768834
SIX1	-0.048543666	0.278634751
CEP350	-0.048423094	0.279831787
DCLK1	0.048403145	0.280030179
RRAS2	0.04835014	0.280557769
PRR7	0.048313729	0.28092058
LGALS12	-0.048258889	0.281467621
ACAA2	-0.048200001	0.282055843
GTF2H2	-0.048023879	0.283820052
ZCCHC9	0.047905686	0.285008153
SIK1	0.047892309	0.285142837
CALM2	0.047791418	0.286159984
SKAP1	-0.047725774	0.286823106
ANXA1	0.047420396	0.289921524
PKIG	-0.047328595	0.290857331
CXCL1	0.047325031	0.290893699
XKR9	0.047317488	0.290970693
SFRP4	0.047296606	0.291183891
CTDSP2	-0.047283163	0.291321207
TRIB2	-0.047280102	0.291352479
PIK3CB	0.047206255	0.292107589
PRAP1	0.047011282	0.294107555
HIPK2	-0.046985563	0.294372059
TDGF1	-0.046921507	0.295031508
OLIG2	0.046891359	0.295342217
MPI	-0.046688617	0.297437408
FZD6	0.046670068	0.297629589
FMN2	-0.046657167	0.297763306
DNASE1L1	-0.046592642	0.298432689
IRF7	-0.046566263	0.298706632

MT-RNR2	0.046520052	0.299186929
ADD2	0.046513348	0.299256644
CIDEA	0.046414545	0.300285449
SRP19	0.046373343	0.300715166
FZD2	0.046138849	0.303168587
S100A14	-0.046072798	0.303862045
ZNF85	-0.046057336	0.304024533
MYO5A	-0.045875145	0.30594344
SHB	0.045820122	0.306524534
PEG10	0.045737113	0.307402564
DEDD2	0.045736793	0.307405948
RNF114	-0.045671892	0.308093596
ASH2L	-0.045591264	0.308949301
TOP3B	-0.045549837	0.30938957
CTSK	0.045396182	0.311026163
NMRAL1	-0.045032248	0.314925138
NIT2	0.044911395	0.316226929
PAQR6	-0.044909564	0.316246682
PHTF1	-0.044889535	0.316462777
SERPINB8	-0.044769568	0.317759147
FZD3	-0.044678987	0.318740263
TMSB15A	0.044599621	0.319601527
MIEN1	0.044530067	0.320357553
POLR3F	0.044491272	0.320779756
CCL21	-0.044448766	0.321242752
ZC3HAV1	0.044385342	0.321934417
ASCL1	-0.044097347	0.325087265
CCKBR	-0.044019815	0.325939475
CTSB	0.044002986	0.326124644
HEG1	-0.043984239	0.32633099
EMILIN1	0.043950892	0.326698258
AIFM1	-0.043879241	0.327488292
IFIT2	-0.043801294	0.32834915
HBEGF	-0.043792425	0.328447198
MAPK12	0.043758824	0.328818812
DUSP3	-0.043475273	0.331965619
PPP2R3B	-0.043456685	0.332172579
SOCS6	0.043429329	0.332477315
TMEM102	-0.043407892	0.332716237
APBB2	0.043345033	0.333417476
PCTP	0.0431389	0.335723705
LEF1	-0.043012291	0.337145272
VEGFA	0.042955533	0.337783797

SF1	-0.042830969	0.339187868
VEGFB	-0.042770635	0.339869287
STAG2	-0.042764154	0.339942531
PCYT2	0.042705623	0.340604507
CHGA	-0.042695306	0.340721281
TNFRSF18	0.042540305	0.342478681
MYH10	-0.042465604	0.343327702
NXN	-0.042446154	0.343548985
ASB5	0.042391252	0.344174083
BLVRB	0.042366073	0.344461008
SOS1	0.042128553	0.347175136
NRG1	-0.042110407	0.347383047
HYAL3	0.041875421	0.35008254
CLDN1	0.041791975	0.351044347
NCAM1	-0.041729142	0.351769668
RNU6-857P	-0.041693278	0.352184092
CAST	-0.041687039	0.352256213
SNX10	0.041646708	0.352722681
FLNA	-0.041456391	0.354929149
TSPAN9	-0.041444151	0.355071352
ZEB2P1	0.04143513	0.355176184
THPO	0.041384173	0.355768695
TGDS	-0.041231689	0.357545425
TMEM167A	0.04107425	0.35938572
SQSTM1	-0.041036541	0.359827378
ABLIM3	-0.041019658	0.360025232
NCOR2	0.040892831	0.361513673
PRKAR1B	0.040873502	0.361740847
CNOT7	-0.040869454	0.361788441
SIRT6	0.040794085	0.362675204
CACNA1E	0.040757509	0.363106045
PTPMT1	-0.040719495	0.363554146
MCL1	0.040615352	0.364783544
TNFAIP3	-0.04061109	0.364833913
NDUFA13	-0.040525939	0.365841122
CAPN1	0.040517275	0.365943703
APPL1	-0.040477542	0.36641435
CRTC3	0.040439423	0.366866241
STPG1	-0.04030695	0.368439362
ATRAID	0.040181195	0.369936581
AKT3	0.040091429	0.371007617
UTS2	-0.040049707	0.371506068
UGT2B7	0.040026687	0.37178126

WEE1	0.039976262	0.372384523
NFKB2	0.039851286	0.373882284
TMEM131	0.039812045	0.374353325
MYD88	-0.039796605	0.374538766
PLAT	0.039780849	0.374728059
CDH19	0.039750109	0.375097537
NREP	0.039736	0.375267193
TBC1D30	-0.039679476	0.375947359
GNAI1	0.039587796	0.377052192
HMOX1	-0.039548713	0.377523777
PRKAR2A	-0.039519934	0.377871271
STRADB	-0.039416403	0.37912297
NFKBIA	-0.039395066	0.379381248
MFN2	0.039393216	0.37940365
USP7	0.039163215	0.382194727
XK	-0.038816274	0.38642857
CAV2	0.038723635	0.38756389
MTRNR2L13	0.038670881	0.388211303
PCK1	0.038596605	0.389123958
EIF3E	0.038499837	0.390314941
RIT1	-0.038498302	0.39033385
IFNA16	-0.038448826	0.390943651
CCL4	-0.038403154	0.39150707
NGF	-0.03840008	0.391545018
EDAR	0.038394134	0.391618405
CHP1	-0.038390829	0.391659199
ARNT	-0.038319464	0.392540782
INSM1	-0.038185388	0.394200257
SLC9A3R1	0.038145149	0.394699128
PRDM1	-0.038099116	0.395270299
IFI44L	-0.038037468	0.396035998
DBI	-0.038021041	0.396240175
GLIPR1	-0.037804782	0.398934099
TNFRSF10B	-0.037793491	0.399075052
MTRNR2L5	-0.03765079	0.400859055
NAGA	-0.037644592	0.400936644
GNA15	0.037574827	0.401810661
POR	-0.037514469	0.402567752
CDH11	0.037500655	0.402741138
NDUFS4	0.037443004	0.403465249
PPARGC1A	0.03743103	0.403615746
GOLGA2	0.037387373	0.404164718
MORC1	-0.037341968	0.404736159

RNF183	-0.037281425	0.405498855
F9	0.037254486	0.4058385
NME6	0.037197495	0.406557594
CXCL11	0.037113997	0.407612507
WDR33	-0.037063068	0.408256739
VCAM1	0.036936786	0.409856775
GOLGA4	-0.036902902	0.410286728
PRKCG	0.036898806	0.410338725
TNFRSF10D	-0.036719834	0.412614297
EAPP	-0.03661624	0.413934856
PNN	-0.03661132	0.413997644
MYB	-0.036576145	0.414446639
NT5C	-0.036495245	0.415480402
SERPINF1	-0.036364255	0.417157434
ST20	0.036355875	0.417264854
SGPP1	0.036278865	0.418252794
GNRH2	-0.036163161	0.419739715
URI1	0.036118035	0.420320472
TTC1	-0.036026368	0.421501639
SLC39A4	0.036014751	0.421651466
DDX46	-0.035853485	0.423734593
ATG4D	-0.035804988	0.424362217
ZNF12	0.035771305	0.424798437
CPNE3	-0.035759479	0.424951662
MFAP1	0.035759109	0.424956455
TTLL7	0.035710094	0.425591848
ZNF268	-0.035694644	0.425792245
KLF11	-0.035665716	0.4261676
C18orf21	0.035663492	0.426196474
CLPTM1L	-0.0356561	0.426292415
CD274	0.035507406	0.428225202
NFE2L3	0.035502878	0.428284139
SH3BP1	0.035492685	0.428416825
S100A3	0.03541251	0.429461342
CEP63	-0.035373961	0.429964088
ITPK1	-0.035357722	0.43017597
PRNP	-0.035308874	0.430813702
CTNNAL1	0.035234032	0.431791855
PRKACA	-0.035169997	0.432629783
SMCHD1	0.034949262	0.435525379
CDH4	-0.034911167	0.436026226
GATM	-0.034848054	0.43685673
MEF2D	-0.034827594	0.43712617

IFI16	0.034741584	0.438259837
NQO1	0.034688212	0.438964155
TIMP3	0.034688176	0.438964642
TRIM27	-0.03454311	0.440882281
RLN2	-0.034438206	0.442271994
VWA8	-0.034281548	0.444351969
ESR1	0.034268322	0.444527829
AIFM2	0.034247034	0.444810961
ZNF138	0.03410767	0.446667072
TDRD7	-0.033857084	0.450015521
EMP1	0.033843261	0.450200649
SIVA1	-0.033717083	0.451892435
GNG13	-0.033647843	0.452822329
ZNF292	-0.033613026	0.453290326
IFNB1	0.033553369	0.454092845
MAP1LC3B	-0.033491406	0.45492724
PSIP1	-0.033429478	0.455762005
IREB2	0.033357704	0.456730589
LCN2	-0.033229057	0.458469519
SS18	0.033167162	0.45930748
ZNF623	0.033124642	0.459883629
NDUFA3	0.033061302	0.460742642
BCL2A1	-0.033026656	0.461212884
PANK3	0.033002036	0.461547207
PPM1B	0.032997552	0.461608105
IL3	-0.032896974	0.462975404
TRAM1	-0.032862114	0.463449821
CREB5	0.032815787	0.464080722
FGF16	0.032813471	0.464112266
TM7SF3	-0.032769723	0.464708508
TAP2	0.032765389	0.4647676
TWIST2	0.032743539	0.465065572
SPTAN1	0.032507795	0.468287171
IGSF3	-0.032411129	0.469611731
BDH1	-0.032341502	0.470567059
PTPN2	-0.03228483	0.471345432
SLC30A5	-0.032046696	0.474623852
ATP7B	0.032008822	0.475146419
MTRNR2L12	0.031988262	0.475430226
CAPNS2	0.031933903	0.476181034
ZDHHC14	-0.031930318	0.476230575
PFAS	-0.031886692	0.476833644
DLEU1	0.031878787	0.476942968

MTRNR2L10	0.031830168	0.477615624
CASP4	-0.03175129	0.478708032
DAAM1	-0.031695823	0.479477038
NOS1	0.031483961	0.482420463
UBA3	0.031388675	0.483747482
TTC39A	-0.031369453	0.484015416
CPPED1	-0.031329764	0.4845689
WDR36	0.031318784	0.484722074
RPS6KA6	0.03129411	0.485066403
MEP1B	-0.03124522	0.485749036
MKLN1	0.031150969	0.487066503
MBD2	-0.030829619	0.491572861
CSNK1D	0.030790668	0.492120594
TRPC5	0.030719784	0.493118197
PLS3	0.030513069	0.496033631
PCBP4	0.030490467	0.496352946
SHOC2	0.030453673	0.496873008
MLX	-0.030451492	0.496903848
GDF11	-0.030427032	0.497249748
ATP6AP1	-0.030368592	0.498076699
CHST15	0.030224487	0.500118961
GABARAPL1	0.030194196	0.500548806
IL4R	-0.030171559	0.500870168
MXRA8	0.03013061	0.501451754
PDGFRB	0.030082005	0.502142552
RTN4R	0.030061593	0.502432815
MAP2K3	-0.029917967	0.504477638
PCSK9	-0.029911525	0.504569457
KIAA0100	0.029839061	0.505602909
COL8A1	0.029792542	0.506266911
RBM14	0.029774825	0.506519928
SSPN	0.029736397	0.507068934
UBXN1	-0.029717146	0.507344089
CDC25B	0.029704442	0.507525708
LGALS14	0.029568855	0.509466201
IFNL1	0.029429916	0.511458677
FZR1	0.029394345	0.511969449
ADCY1	-0.029363396	0.512414061
IMPA2	-0.029360562	0.512454778
DYNLL2	-0.029306974	0.513225124
PAGE4	0.029197049	0.514807222
SMIM8	-0.029089353	0.51635969
ID1	0.029037324	0.517110564

POLR2M	0.028993512	0.517743287
VDAC1P4	-0.028986483	0.517844832
PPT1	-0.028945038	0.518443802
CBL	0.028933006	0.51861776
CSGALNACT2	-0.028773992	0.520919564
TSPY1	0.028644651	0.522795687
AKR1C1	-0.028604173	0.523383542
FILIP1L	-0.028590888	0.523576553
TGFB3	0.028533621	0.524408974
TGFB2	-0.028505555	0.524817168
MAPK4	0.028324446	0.527455201
CEACAM1	-0.028118135	0.530468503
HMGN2P47	-0.02801913	0.531917633
TMEM121	0.028016373	0.531958015
ABI2	-0.027945325	0.532999205
OSTM1	0.027900992	0.533649414
PRDX2	0.027873048	0.534059465
GTPBP3	-0.027826115	0.534748502
GNPDA1	0.027691191	0.536731867
PPP2R3A	-0.027673791	0.536987916
PLXNA3	-0.027566515	0.538567868
GOLIM4	0.027558934	0.538679606
P2RY2	-0.027472921	0.539948194
ASPHD1	-0.027470903	0.539977979
LATS1	0.027465367	0.540059676
PTK2	0.027393446	0.541121694
LAMA5	0.027349477	0.541771468
CIZ1	0.027277638	0.542833926
CCK	-0.027269683	0.542951639
GPR27	0.027257584	0.54313071
GRM4	-0.027202311	0.543949099
DHRS2	0.027191875	0.544103688
RHBDD3	0.027179305	0.544289913
EP300	-0.027153451	0.544673039
INF2	0.027136757	0.544920492
PML	0.027128159	0.545047976
PDGFD	-0.02703808	0.546384351
LAMTOR5	0.026971493	0.547373251
MTRNR2L9	0.026869608	0.548888074
NTF3	-0.02673921	0.55082984
ZNF385A	0.026632059	0.552427955
TNFAIP2	-0.026622686	0.55256785
ATL1	0.026597284	0.552947099

TFAP2C	0.026517825	0.554134189
ZNF16	0.026507014	0.554295811
IFI44	-0.026459436	0.5550073
AFP	0.026317686	0.557129729
NDUFB8	0.026200645	0.558885159
IGF1	-0.026111305	0.560226903
NIPBL	0.026100811	0.560384614
FXYD5	0.026072027	0.560817294
HMGN2P9	0.02591478	0.563183888
AQR	0.02590553	0.563323244
IFNA14	0.025729288	0.565981726
FGF19	0.025476904	0.569799163
C18orf25	-0.025475899	0.569814389
RLF	0.025414553	0.570744135
USP17L1	0.02522757	0.573582457
VCX2	-0.025137102	0.574958103
PDLIM3	0.025080779	0.575815339
ROCK1	-0.024986454	0.577252293
SLC45A2	0.024985372	0.577268799
PARP12	0.024910505	0.578410548
TRAF2	0.024659497	0.5822462
TANK	-0.024654496	0.582322753
TGM2	-0.024607181	0.583047132
VIM	-0.024510729	0.584525111
ZC3H8	-0.024509732	0.584540407
JTB	0.024494574	0.584772835
LIMA1	-0.024350954	0.586977224
GNG12	0.02428494	0.587991744
KCTD11	0.024221361	0.588969608
CLIP3	0.023969013	0.592858157
MFGE8	0.023816137	0.595219585
PRKCI	-0.023703357	0.596964402
NRP1	-0.023581733	0.598848639
ZNF420	-0.023570428	0.599023916
TRH	0.023549664	0.599345909
CLCN4	-0.023502717	0.600074211
PPP1R10	-0.023425363	0.601275096
PA2G4P3	-0.023421979	0.601327663
SNAPC2	-0.023393681	0.601767268
APRT	-0.023313623	0.603011735
MNS1	-0.02316487	0.605327094
PTDSS2	0.023131475	0.605847443
LINC01006	0.023130529	0.605862189

PARP9	0.023123869	0.60596598
DNAJA3	0.02300802	0.607772788
SH2D2A	0.022936674	0.608886707
PSMF1	0.022758856	0.611666885
DAXX	0.022589482	0.61432025
CDK5RAP2	-0.022556112	0.614843608
FGF6	-0.022531583	0.615228432
KIN	-0.022403585	0.617238247
ZHX1	0.022392903	0.617406112
MAP2	0.022388506	0.617475209
TFPT	0.022350396	0.618074263
GNA11	-0.022295656	0.61893517
RAD1	0.022186917	0.620646876
BMP6	-0.022120713	0.621690024
RNF152	-0.021818001	0.62646938
PXDC1	0.021807192	0.626640328
ZBTB17	-0.021784294	0.627002534
JUNB	-0.021758332	0.627413326
RAMP1	-0.021734862	0.627784765
IFIT3	-0.021731552	0.62783717
DUSP7	0.021695542	0.62840729
LUM	-0.021658066	0.629000853
SPTBN1	0.021638941	0.629303866
MTRNR2L3	0.021628145	0.629474925
LDOC1	-0.021627313	0.62948811
CHD1	-0.021615714	0.629671944
DDX42	-0.021610315	0.629757515
OAS2	0.02160699	0.629810202
API5	-0.021243437	0.635583865
NSMAF	0.021173934	0.6366902
HDAC9	-0.021132289	0.637353472
EIF5	0.021112101	0.637675109
NFKBIE	0.021079831	0.63818939
RNF138	-0.021059088	0.638520048
OGDHL	0.021033076	0.638934807
PLTP	-0.02094616	0.640321473
MYCBP	-0.020841801	0.641988101
CENPBD1	0.020810299	0.642491539
CASP8	-0.020612329	0.645659107
MUC4	-0.020582838	0.646131514
FLOT1	0.020557567	0.646536438
SIAH1	0.020119526	0.653571831
MAPK14	-0.020119302	0.653575433

ATF6	0.020082656	0.65416541
VSX2	-0.020043378	0.654798012
RASSF6	0.020028942	0.65503057
EBF3	0.019933117	0.656575129
CYP4F2	0.019836331	0.65813668
ZNF239	0.019795135	0.658801778
BMP10	0.01967108	0.66080626
SMARCAD1	0.019638623	0.661331117
PHOX2B	-0.019544751	0.662849998
NAA40	-0.019304137	0.666749509
RFPL1	0.019296767	0.666869106
ERBB2	-0.019213503	0.668220719
RB1	-0.019093983	0.670162748
GAMT	-0.019040658	0.671029913
MAPK3	0.018981688	0.671989388
COX4I1	-0.018961561	0.672316992
MTRNR2L6	0.01890523	0.673234192
PRKCA	0.018887929	0.673515992
GNB5	0.018866151	0.673870782
UBA52	-0.018860155	0.673968477
PKP1	0.018789862	0.675114185
TNFRSF10A	-0.018728437	0.676115968
TOPORS	-0.018635572	0.67763159
SOX2	-0.018525372	0.67943183
NHP2	-0.018490351	0.680004306
COLEC11	-0.01848124	0.680153276
BET1	0.018374298	0.681902738
LSM7	-0.018369303	0.681984489
BIRC3	0.018329068	0.68264317
MRPL57	-0.018302449	0.683079067
MLKL	-0.01827015	0.683608131
PPP1R1C	0.018186391	0.684980822
OR2AT4	0.018068054	0.686921981
ZNF518A	-0.018054815	0.687139278
SIPA1L3	-0.017959573	0.68870327
CTNNA1	0.017925375	0.689265163
NAP1L1	0.017763065	0.691934376
PSMB11	-0.017728162	0.692508847
SRA1	-0.017700466	0.692964836
UROS	-0.017655926	0.69369837
ALDH1L1	0.017573221	0.695061219
FGF13	0.01753417	0.695705052
MRPL34	0.017501034	0.69625154

SOCS3	0.017446069	0.697158383
GHSR	0.017391799	0.698054186
ZNF644	0.017313435	0.699348433
NKAP	-0.017178467	0.70157959
ZSCAN10	-0.017116766	0.702600413
ICA1	-0.017090245	0.703039369
IL1B	0.017066472	0.703432927
FOXJ3	0.016759592	0.708520265
ZNF45	-0.016682818	0.709795033
BAX	0.016654543	0.710264732
MIS18BP1	-0.016526645	0.712390657
UBE2L6	-0.016437572	0.713872567
OTUD4	-0.016346101	0.715395487
ALDOC	0.016295153	0.716244215
STK17A	0.016287058	0.716379103
WWTR1	-0.016259228	0.716842892
INSR	-0.01625662	0.716886364
EFR3A	0.016180015	0.71816357
SCLY	-0.016170266	0.718326172
WWP1	0.016090417	0.719658391
CD24	0.016048678	0.720355123
MTRNR2L11	-0.016037001	0.72055008
TRAF3	-0.015965413	0.721745704
TIAL1	-0.015934595	0.722260618
XPO7	-0.01586937	0.723350839
TRIM33	-0.015813614	0.724283218
BTBD7	-0.015800216	0.724507336
PCSK6	-0.015792807	0.724631277
CCL2	-0.015771128	0.724993983
PPP1R14A	-0.015754765	0.725267773
SFPQ	0.015727455	0.725724829
MIR718	-0.015687608	0.726391871
PRKAA1	-0.01567649	0.726578033
MAP3K9	-0.015622928	0.727475073
KHK	0.01550405	0.72946733
SSX1	0.015321999	0.732521834
PHYH	0.015319266	0.73256772
ALDH1A2	-0.015306068	0.73278933
IDO1	-0.015247908	0.733766169
RNF20	0.015086606	0.736477574
MTRNR2L1	0.015029346	0.73744089
GRHPR	0.015006429	0.73782655
MAK16	0.014918853	0.739300914

NEK3	0.014801006	0.74128641
FAT2	0.014757116	0.742026304
CALCA	-0.014710576	0.742811143
IFNA17	0.014687111	0.743206953
ATP2A1	-0.014661413	0.743640514
USP34	-0.014581083	0.744996275
ARFGAP1	0.014545799	0.745592031
MED21	0.01445233	0.747170947
NOTCH2	0.014419079	0.747732897
RARB	0.014314161	0.749506895
SAP18	0.014269148	0.75026841
TSPY2	0.014164306	0.752043005
PSMB9	-0.014006321	0.754719594
ADRA2C	-0.013976564	0.755224075
MRPL41	0.013962568	0.755461385
GGNBP2	-0.013848989	0.757388046
PRKACB	-0.013738613	0.759261829
ZFAT	-0.013624518	0.761200204
SMAD2	0.013422132	0.764642267
TM2D1	0.013408259	0.76487838
IDI1	-0.013340175	0.766037455
SPATA16	0.013310213	0.766547711
TPD52L1	0.013169412	0.768946884
NGB	-0.013112395	0.76991904
CHRNA3	0.013099172	0.770144547
FABP6	-0.013023975	0.771427367
USP17L6P	0.012995738	0.771909224
LPCAT4	-0.012937416	0.772904768
IFITM3	-0.012823205	0.77485539
HEPACAM2	-0.012722424	0.776577831
VIL1	0.012662589	0.777600983
DECR1	-0.012514404	0.780136525
FER	-0.01246783	0.780933924
ABCB9	-0.012354009	0.782883624
DNAJA2	-0.012289355	0.783991724
IFNA10	-0.012286644	0.78403819
MAPKAPK2	-0.011956676	0.789700333
ZC3H12A	0.011912549	0.790458379
LDLR	-0.011905331	0.790582393
SNAP25	-0.011831812	0.791845869
SLC27A2	0.011798314	0.792421733
IL15RA	0.011589187	0.796019359
ACTG2	-0.011553992	0.79662526

NTN1	0.011510002	0.797382738
CAPN14	0.011498836	0.797575033
ENSA	0.011483891	0.797832433
IPO13	0.011461507	0.798217989
OLFM4	0.011381277	0.799600356
HIRA	0.011223332	0.802323583
GNG10	0.011151739	0.803558745
UBE3A	-0.011070465	0.804961524
PPP2R5B	0.010969543	0.806704314
MTCH1	-0.010899031	0.807922514
ARFGEF1	-0.010884384	0.80817563
UBE2D3	-0.010860502	0.808588359
ISYNA1	0.010704648	0.811283195
UBIAD1	-0.010570555	0.813603559
PYROXD1	0.010408796	0.816404815
PRAF2	0.010389748	0.816734827
SERF2	0.010353871	0.817356499
THOC6	0.010342742	0.817549369
CANX	-0.010328469	0.817796746
AKR1C3	0.010234987	0.819417359
NIN	-0.010154126	0.820819781
FLII	0.01010063	0.82174791
GFER	0.010061519	0.822426637
MEPCE	0.009961922	0.824155597
IFI27	-0.009731312	0.828162157
IER3IP1	0.0096334	0.829864601
MSH3	-0.00924718	0.836587728
UTS2R	-0.009234698	0.836805225
DNAJC3	-0.00920898	0.837253353
NEK6	0.009128482	0.838656387
RNF213	-0.009009381	0.840733185
CTNND2	-0.008872578	0.843120005
IFNA8	0.008790326	0.844555772
ADCK2	-0.008750659	0.845248365
SNX19	-0.008572161	0.848366472
TNPO2	0.00850281	0.849578568
PSMA8	0.008464651	0.850245646
BIRC6	-0.008457465	0.850371292
SRGAP2	-0.008416085	0.851094817
BMPR1A	0.008283915	0.853406684
IDE	-0.008246604	0.854059532
CLDN3	-0.008180623	0.855214275
PIWIL2	0.008097081	0.856676827

STRBP	-0.007942321	0.859387428
MGAT5	0.007909152	0.859968596
MAP4K2	-0.007901219	0.860107612
SEC62	0.007888352	0.860333093
TNFRSF11B	-0.007881209	0.86045827
PPP3CA	-0.007801571	0.861854094
DDX3Y	-0.007778033	0.862266739
PDGFRA	-0.007722587	0.863238895
PIK3R2	0.007667566	0.86420381
ELL2	0.007541417	0.866416869
TMEM161A	-0.007515969	0.866863436
LACTB2	0.007415892	0.868620014
RPS3	0.007399554	0.868906843
NFATC4	-0.007285639	0.870907208
RPS4Y1	0.007031601	0.875371113
CDH1	-0.006981127	0.87625851
MX1	0.006905428	0.877589689
AIPL1	0.006816794	0.879148759
ATF7IP	-0.006811367	0.879244232
SSX2	0.0065052	0.884633379
ZNF271P	-0.006373136	0.886959615
RYK	-0.006229581	0.889489376
UNC5A	0.006130894	0.891229107
CDK11B	-0.005997355	0.893584071
PLEKHN1	0.005924115	0.894876044
IER2	0.005787828	0.89728095
BMP7	0.005712522	0.898610192
ANAPC15	-0.00560139	0.900572337
HECTD1	-0.005565058	0.901213942
PDCD6IP	-0.005548572	0.901505102
IFNA4	-0.005485084	0.902626478
ARHGAP29	0.005444449	0.903344292
LALBA	0.005409876	0.903955096
SRSF2	-0.005340208	0.9051861
RHEBP1	-0.004981363	0.911530251
AKIP1	-0.004923661	0.912550928
FASTK	0.004797654	0.914780317
THOC1	-0.004759396	0.915457344
PRPSAP2	-0.004721099	0.916135106
BNIP1	-0.004674051	0.916967825
SREK1IP1	-0.004669795	0.917043166
PLK2	0.004631467	0.917721628
AXIN1	0.004498333	0.920078736

MLEC	-0.004467039	0.92063289
HTR2B	-0.004407407	0.921688971
NLRP2	0.004384413	0.92209624
DTNA	0.004376212	0.922241497
CLEC2B	-0.004368371	0.922380367
ALKBH8	0.00434615	0.922773966
ING1	0.004239916	0.924655961
FOXD2	0.00418888	0.925560233
UBR4	0.004158472	0.926099052
BCR	0.004123369	0.92672112
CTSC	-0.004038843	0.928219187
ODC1	-0.004013382	0.928670488
NHLH2	-0.003860465	0.931381442
BEGAIN	0.003759172	0.933177636
HAUS3	-0.003610884	0.935807795
TRIM69	-0.003608397	0.935851913
EIF3C	0.003598703	0.936023875
TNFRSF9	0.003562903	0.936658959
ZNF107	-0.003528059	0.93727714
CCNB1IP1	-0.003505361	0.937679854
SEC16A	0.003501981	0.937739818
ZNF257	0.003169807	0.94363507
ATR	-0.003146152	0.944055008
AP3B1	-0.00300525	0.946556709
BPTF	-0.002767087	0.950786447
AIFM3	0.002730228	0.951441185
SKIV2L	-0.002646044	0.952936697
BRD3	0.002629461	0.953231298
GNS	-0.002561407	0.954440406
USP28	-0.00250898	0.955371942
KLF6	-0.002489481	0.955718429
APAF1	-0.002483059	0.955832532
EIF2B5	-0.002468764	0.95608655
CDK14	0.002451254	0.956397713
HSPA12A	0.00241569	0.95702969
PEA15	-0.002343731	0.958308515
EMC2	-0.002277069	0.959493312
BECN1	-0.002196002	0.960934242
CASP5	0.002193296	0.960982339
FZD9	-0.002177052	0.961271081
SLC25A5	-0.001997995	0.964454238
NOL4	0.001890645	0.966362911
WDR26	0.001887964	0.966410578

SEC14L1	0.001862638	0.966860913
TYMP	0.001806902	0.967851994
PHLPP2	-0.001800289	0.967969582
YAP1	-0.001651028	0.97062396
WRN	0.001576473	0.971949919
NOS3	-0.001555975	0.972314503
WBP4	-0.001539302	0.972611039
ZNF329	0.001451913	0.974165407
CUX1	0.001450659	0.974187716
ELSPBP1	0.001383539	0.975381617
CMC4	-0.001364722	0.975716345
SRGN	-0.001360839	0.975785413
CSRNP3	0.001219862	0.978293294
RHOA	-0.001172298	0.979139461
SEC63	0.001136954	0.979768265
MOB1B	-0.001111974	0.980212681
ACTA1	0.001094161	0.980529588
RHBDD1	-0.00095916	0.982931523
MAP3K2	0.000870514	0.984508794
RNF41	0.000783914	0.986049707
BRF2	0.000737901	0.98686848
MTA1	-0.000674331	0.987999661
RPH3A	-0.000657689	0.988295801
TIPARP	0.000535767	0.990465396
MAP1S	0.000494901	0.991192624
RPL36AL	-0.000462265	0.991773411
SEMA4G	0.000390179	0.993056241
DNPEP	0.000183722	0.996730388
POMK	-0.000177115	0.996847959
PHLPP1	-7.99E-05	0.998578761
PHF3	4.84E-05	0.999138422
TICAM2	2.94E-05	0.999476974
**Necroptosis**		
CYLD	-0.350604258	6.59E-16
RIPK3	-0.340544347	4.84E-15
FADD	0.328996143	4.37E-14
TLR3	-0.255929962	6.42E-09
TRPM7	-0.236837406	8.37E-08
ZBP1	-0.216924173	9.74E-07
IPMK	0.210017629	2.17E-06
FAS	-0.176660484	7.14E-05
TNF	-0.165752753	0.000196986
FASLG	-0.157432468	0.000410026

MAP3K7	0.140932583	0.001581798
PGLYRP1	-0.140407205	0.001647592
CASP6	0.111262319	0.012795612
SPATA2	0.082728436	0.064543986
RIPK1	-0.069602418	0.120101552
KLHDC10	-0.06119238	0.171889056
PELI1	-0.044861304	0.316767532
ITPK1	-0.035357722	0.43017597
CASP8	-0.020612329	0.645659107
MLKL	-0.01827015	0.683608131
**Pyroptosis**		
NLRP1	-0.459218003	1.89E-27
CARD8	-0.406485315	2.57E-21
CYCS	0.383752331	5.45E-19
CASP1	-0.309046546	1.58E-12
IRF2	-0.293983578	2.00E-11
NLRC4	-0.27639447	3.22E-10
NLRP3	-0.250231181	1.41E-08
GSDMB	-0.241932626	4.31E-08
NAIP	-0.226415248	3.11E-07
GSDMD	-0.226106861	3.23E-07
ZBP1	-0.216924173	9.74E-07
TREM2	-0.216919613	9.74E-07
IL1A	0.208174437	2.67E-06
CHMP4A	-0.202717143	4.90E-06
UBR2	-0.187441622	2.46E-05
ELANE	-0.179631853	5.36E-05
CHMP4B	0.169724567	0.000137091
APIP	-0.162476396	0.000264024
CASP3	0.160663255	0.00030975
CHMP2B	0.150835147	0.000715127
DHX9	0.146896848	0.000986389
CHMP7	-0.145457054	0.001107301
NLRP6	-0.144950532	0.00115299
CHMP4C	0.138346853	0.001930573
GZMA	-0.123588231	0.005653728
DPP8	-0.119034547	0.007710037
GSDMA	-0.118301699	0.008097191
CPTP	0.117378725	0.008609421
DPP9	0.116703103	0.009002483
CASP6	0.111262319	0.012795612
CHMP6	-0.108459797	0.015252559
TP53	-0.103693151	0.02038958

GSDMC	0.103243644	0.020943958
NLRP9	-0.096423173	0.031105815
IL18	-0.094066884	0.035483304
AIM2	0.086913632	0.05210559
PYCARD	-0.086469759	0.053322032
BAK1	0.080576278	0.071835576
GZMB	0.07932777	0.076365209
TP63	-0.073856318	0.099025279
CHMP2A	0.068042431	0.128654731
IRF1	-0.061809728	0.1675965
HMGB1	0.060128053	0.179481319
CHMP3	-0.045758978	0.307171117
CASP4	-0.03175129	0.478708032
DDX3X	0.027733668	0.536107067
CASP8	-0.020612329	0.645659107
IL1B	0.017066472	0.703432927
BAX	0.016654543	0.710264732
CASP5	0.002193296	0.960982339
**Ferroptosis**		
RRM2	0.529970388	1.49E-37
SLC2A1	0.529436595	1.81E-37
AURKA	0.459623638	1.68E-27
DUOX1	-0.457950014	2.73E-27
CDCA3	0.448611655	3.96E-26
IL33	-0.445110952	1.06E-25
ACADSB	-0.420543451	7.56E-23
EPT1	0.409873561	1.12E-21
SLC7A5	0.375983606	3.10E-18
HILPDA	0.373266266	5.63E-18
FLT3	-0.373159668	5.76E-18
MDM4	-0.367241266	2.07E-17
ISCU	-0.366478587	2.44E-17
MMD	0.365664292	2.90E-17
AGPS	0.360826411	8.06E-17
AQP3	-0.356634658	1.92E-16
VDAC2	0.349426954	8.35E-16
SUV39H1	0.348449131	1.02E-15
PANX1	0.346753591	1.43E-15
SIRT3	-0.346066148	1.63E-15
NRAS	0.343836219	2.54E-15
PLA2G6	-0.34255724	3.27E-15
ACSL5	-0.341715046	3.85E-15
TRIB3	0.340643448	4.75E-15

SLC16A1	0.324435593	1.02E-13
STMN1	0.319673548	2.42E-13
TXNIP	-0.319094322	2.69E-13
ELAVL1	0.316874525	4.00E-13
EIF2S1	0.316389794	4.36E-13
HERPUD1	-0.316186905	4.52E-13
ALOX15B	-0.31169142	9.99E-13
PSAT1	0.309411039	1.49E-12
TFAP2A	0.301452061	5.80E-12
TSC22D3	-0.301355308	5.89E-12
ANGPTL7	-0.300175156	7.18E-12
GLS2	-0.299509824	8.03E-12
AQP5	-0.294447811	1.85E-11
ARNTL	-0.292978643	2.36E-11
SLC39A8	-0.29251137	2.54E-11
ATM	-0.283789914	1.03E-10
EGLN2	-0.281563524	1.45E-10
SLC39A14	0.278313692	2.40E-10
MUC1	-0.276748584	3.05E-10
CISD2	0.276034314	3.40E-10
CDO1	-0.275884936	3.48E-10
HELLS	0.274266671	4.45E-10
SLC3A2	0.273993637	4.64E-10
PLIN2	0.272965292	5.41E-10
ALOX15	-0.272949704	5.42E-10
LINC00472	-0.271161084	7.09E-10
HIF1A	0.270335077	8.02E-10
BCAT2	-0.270266719	8.10E-10
ALOX5	-0.270114675	8.29E-10
CEBPG	0.269485971	9.10E-10
TXNRD1	0.266926555	1.33E-09
ALDH3A2	-0.266458354	1.42E-09
SLC40A1	-0.264403475	1.92E-09
FANCD2	0.260974878	3.14E-09
HSD17B11	-0.260183282	3.52E-09
UBC	0.259018912	4.15E-09
CD82	-0.256277632	6.12E-09
SLC38A1	0.25451901	7.82E-09
ASNS	0.254184556	8.19E-09
P4HB	0.252876385	9.82E-09
PCBP2	0.25020269	1.42E-08
GCLC	0.250092369	1.44E-08
NOX4	0.249861072	1.49E-08

TSC1	-0.249680022	1.52E-08
FADS1	0.249487916	1.56E-08
SLC25A28	-0.246763544	2.26E-08
ARRDC3	-0.246288801	2.41E-08
NEDD4	0.245945905	2.52E-08
GLRX5	0.240849982	4.97E-08
GSS	0.240622021	5.12E-08
NEDD4L	-0.240189935	5.41E-08
PEBP1	-0.238750767	6.53E-08
KRAS	0.237890451	7.30E-08
TLR4	-0.236266078	9.01E-08
GABARAPL2	-0.236138824	9.15E-08
CYBB	-0.231157597	1.72E-07
ULK2	-0.230067813	1.98E-07
DNAJB6	0.229947226	2.01E-07
CYB5R1	-0.226485803	3.08E-07
ALOXE3	0.223284838	4.56E-07
DPP4	-0.222944265	4.75E-07
BACH1	0.220108786	6.68E-07
BID	0.219897161	6.85E-07
SMAD7	-0.219176823	7.46E-07
ENPP2	-0.217892508	8.69E-07
HSF1	0.21757294	9.02E-07
MAP1LC3A	-0.216018109	1.08E-06
ZNF419	-0.214463712	1.30E-06
GDF15	-0.213846147	1.40E-06
SRXN1	0.213428155	1.46E-06
TIMM9	0.212459614	1.64E-06
SCP2	-0.212337817	1.66E-06
PGD	0.212181131	1.69E-06
SAT2	-0.2119832	1.73E-06
MTDH	0.211470414	1.83E-06
SCD	0.209216389	2.37E-06
TFRC	0.208002936	2.72E-06
WIPI2	0.207774882	2.79E-06
LYRM1	-0.207312205	2.94E-06
AR	-0.206936882	3.07E-06
DLD	0.20628415	3.30E-06
CS	0.205715848	3.52E-06
ZFP36	-0.205672516	3.53E-06
LIG3	0.205234275	3.71E-06
ACSL3	0.204357915	4.09E-06
RELA	0.201242937	5.76E-06

SLC1A5	0.20018357	6.46E-06
PRDX1	0.198345528	7.88E-06
FADS2	0.19786276	8.30E-06
DDIT3	0.197061353	9.04E-06
ARF6	0.194505672	1.19E-05
SLC7A11	0.191985479	1.54E-05
GCLM	0.190952023	1.72E-05
CISD1	0.189880507	1.92E-05
AHCY	0.189836737	1.93E-05
TMBIM4	-0.189817801	1.93E-05
ATG5	0.189591142	1.98E-05
LINC00336	-0.18841436	2.23E-05
YWHAE	0.187923712	2.34E-05
STEAP3	-0.187282785	2.50E-05
NCOA4	-0.186961806	2.58E-05
CHAC1	0.186530856	2.70E-05
SAT1	-0.185758823	2.92E-05
RPTOR	0.184500091	3.31E-05
PHKG2	-0.183637015	3.61E-05
SLC2A14	0.183237549	3.76E-05
ALOX12	-0.18318832	3.77E-05
SOCS1	-0.183031917	3.83E-05
ABCC1	0.182761695	3.94E-05
RGS4	0.182023987	4.24E-05
HBA1	-0.181555482	4.44E-05
PRKAA2	0.180339025	5.00E-05
TFAM	0.180319014	5.01E-05
ACSL1	-0.179599455	5.37E-05
DUSP1	-0.177862406	6.36E-05
ZFP69B	0.176653017	7.14E-05
DDIT4	0.176132864	7.51E-05
PCBP1	0.172651041	0.00010441
MAP1LC3C	-0.17263262	0.000104591
CXCL2	-0.172602152	0.00010489
ANO6	0.172487585	0.000106023
KLHL24	-0.171114791	0.000120522
TUBE1	-0.170745566	0.000124729
ACOT1	-0.170093489	0.000132497
SLC2A3	0.169454985	0.000140542
TAZ	-0.16877258	0.000149645
HSPA5	0.163223282	0.00024709
G6PD	0.162065643	0.000273793
BRD4	0.162020262	0.000274892

PHF21A	-0.1619468	0.000276681
PTGS2	0.161837126	0.000279373
NR1D2	-0.160598933	0.000311501
VDAC3	0.159359928	0.000347076
FBXW7	-0.159331854	0.000347925
INTS2	0.159097318	0.000355088
ATG16L1	0.158148402	0.000385496
DUOX2	-0.157307812	0.000414443
FH	0.15583374	0.000470143
VLDLR	-0.154307086	0.000535112
CDKN2A	0.153565729	0.000569584
STK11	-0.153532723	0.000571166
ELOVL5	0.153276362	0.00058359
SIAH2	0.152082811	0.000644808
NF2	0.151300688	0.000688097
KDM3B	-0.150599192	0.000729199
PPP1R13L	0.149938665	0.000769968
SLC11A2	-0.149434537	0.000802492
NR4A1	-0.149422618	0.000803276
GOT1	0.148362668	0.000875911
LONP1	0.144486241	0.001196389
AGPAT3	-0.143914485	0.001251899
PPARA	-0.143413408	0.001302486
TBK1	0.142277825	0.001424169
GSK3B	0.142234352	0.001429028
LPCAT3	-0.142212832	0.001431439
PTEN	-0.141126924	0.001558077
WIPI1	0.14068985	0.00161189
TGFBR1	0.140506809	0.001634929
ACSL6	-0.140485621	0.001637615
OXSR1	0.140344456	0.001655616
NCOA3	0.139145084	0.00181604
JDP2	-0.13912837	0.001818373
CTH	0.135273063	0.002436075
CBS	0.134097476	0.002659418
TFR2	0.133302586	0.002820844
PCK2	-0.132000827	0.003104562
CD44	-0.131980509	0.003109189
MAFG	0.131099519	0.003315943
LURAP1L	-0.129496742	0.003724369
EPAS1	-0.128442649	0.004017287
FNDC5	-0.126895096	0.004485188
PIK3CA	0.126054225	0.004759567

SNCA	-0.125699552	0.004879772
FTH1	0.122508352	0.006090809
LPIN1	-0.122501998	0.006093468
JUN	-0.12149648	0.006527653
IL6	0.119255966	0.007596363
ZEB1	-0.118189695	0.008157866
ULK1	0.11716943	0.008729517
COQ2	0.115469283	0.0097617
HSPB1	0.115129983	0.009980221
MT1G	0.111367797	0.012710384
STAT3	-0.110120652	0.013750865
NNMT	0.108796865	0.014936644
CHMP6	-0.108459797	0.015252559
ATP6V1G2	-0.10782042	0.015867957
AEBP2	0.107085844	0.016601762
PLIN4	-0.107045493	0.016642917
PARK7	0.105810894	0.017946068
TP53	-0.103693151	0.02038958
MLLT1	-0.103331297	0.020834838
NCF2	-0.103250247	0.020935719
HMGCR	0.103160066	0.021048466
AMN	-0.103098479	0.021125766
TF	0.102587534	0.021776631
MT1DP	0.100424082	0.02472901
PRDX6	0.099702598	0.025787611
ACSF2	-0.09907182	0.026744909
SLC2A8	-0.098555013	0.027551894
PIR	0.098392559	0.02780985
MAP3K5	-0.097162725	0.029830868
MTOR	0.095812245	0.032193916
CHMP5	0.095215762	0.033287499
ACSL4	0.094646149	0.034361244
MT3	-0.09449195	0.034656942
IDH1	0.093826367	0.035958271
NOX5	0.092346668	0.039000795
PEX2	-0.091716746	0.040360692
AQP8	-0.091698981	0.040399619
BNIP3	0.09115098	0.04161605
TGFB1	-0.090228793	0.043732848
SP1	0.089737084	0.044898106
AKR1C2	0.08885345	0.04705777
FAR1	-0.087848651	0.049618761
HNF4A	0.086916884	0.052096763

DCAF7	0.086487786	0.053272173
FDFT1	-0.08635614	0.05363718
MAPK1	0.086326444	0.053719805
SLC2A6	-0.086065768	0.054449647
MMP13	0.084805287	0.058096041
FZD7	-0.083679989	0.061520127
SREBF1	-0.083580903	0.061829449
HRAS	0.082940348	0.063860193
BRD2	-0.081325044	0.069225941
SIRT1	-0.080592121	0.071779536
ATF3	-0.080110482	0.073499145
ABHD12	0.076484312	0.087550294
CDKN1A	0.07630596	0.088293628
LAMP2	0.075870503	0.09012984
NFE2L2	-0.07525006	0.09279889
CAV1	-0.074818039	0.09469446
TP63	-0.073856318	0.099025279
NR1D1	0.073228326	0.101937312
RPL8	0.072981768	0.103099027
HAMP	-0.072847981	0.103733778
SRC	0.072259687	0.106561736
VDR	-0.070731414	0.114193264
SESN2	-0.070601122	0.114863239
RIPK1	-0.069602418	0.120101552
PEX12	-0.069375224	0.121318854
GPT2	0.068189263	0.127830085
MTF1	-0.068095014	0.128358939
MAPK9	-0.067862554	0.129670579
ATF2	0.066720678	0.136264699
IDH2	0.066590925	0.137030045
MIR6852	-0.066020232	0.140435544
DAZAP1	0.065567663	0.143182003
GCH1	0.065450427	0.143900122
SETD1B	-0.065427224	0.144042573
FTL	-0.064421823	0.150319234
FTMT	0.064214381	0.151639785
EIF2AK4	0.06349816	0.156266893
ATG13	0.062457238	0.163181005
KEAP1	-0.062420527	0.163428984
MIR30B	-0.06220796	0.164870409
MDM2	-0.061879105	0.167119166
ACO1	0.060942741	0.173647949
PEX10	0.06047096	0.177008559

HMGB1	0.060128053	0.179481319
EGFR	0.059893787	0.181185305
MIR9-1	0.058489216	0.191653506
ATF4	0.058213104	0.193762504
PEX3	-0.057622906	0.198327421
ATG3	0.056702726	0.205600238
MAPK8	0.056544982	0.206866148
PANX2	0.055610832	0.214478535
NR5A2	-0.054991345	0.219636531
FXN	0.054633043	0.222660051
ACVR1B	-0.053583135	0.231690384
SNX4	0.052060263	0.245244669
PVT1	-0.051392293	0.251361583
NT5DC2	0.049961988	0.264814286
CTSB	0.044002986	0.326124644
MIR212	-0.043192238	0.335125973
VEGFA	0.042955533	0.337783797
NFS1	0.04268384	0.340851084
DRD5	-0.042158736	0.346829486
SQSTM1	-0.041036541	0.359827378
TNFAIP3	-0.04061109	0.364833913
HMOX1	-0.039548713	0.377523777
LRRFIP1	-0.039403699	0.379276737
MFN2	0.039393216	0.37940365
USP7	0.039163215	0.382194727
CHP1	-0.038390829	0.391659199
GPX2	0.03818926	0.394152283
CAPG	-0.037881951	0.397971559
POR	-0.037514469	0.402567752
MYB	-0.036576145	0.414446639
CP	0.036306007	0.417904446
BAP1	-0.036172704	0.419616955
SREBF2	-0.036003417	0.421797679
ATG4D	-0.035804988	0.424362217
GRIA3	0.035326121	0.430588473
PRNP	-0.035308874	0.430813702
NQO1	0.034688212	0.438964155
AIFM2	0.034247034	0.444810961
MAP1LC3B	-0.033491406	0.45492724
IREB2	0.033357704	0.456730589
PEX6	-0.032915658	0.462721239
MIR4715	-0.032883751	0.463155331
ZFAS1	0.032759064	0.464853842

ALB	-0.032203766	0.472460058
BRDT	0.031936116	0.476150453
LINC00618	-0.030866629	0.491052727
TTPA	-0.030412858	0.497450253
GABARAPL1	0.030194196	0.500548806
HDDC3	-0.030137084	0.501359788
MIR761	-0.029820893	0.505862177
AKR1C1	-0.028604173	0.523383542
PML	0.027128159	0.545047976
NOX1	0.026835428	0.549396724
BRPF1	0.026669376	0.55187113
TFAP2C	0.026517825	0.554134189
IFNG	-0.026154214	0.559582291
DRD4	-0.025263479	0.573036856
NOS2	0.024606646	0.583055336
SLC1A4	-0.024259667	0.588380366
CA9	0.020512555	0.647257944
MAPK14	-0.020119302	0.653575433
RB1	-0.019093983	0.670162748
BLOC1S5-TXNDC5	0.018983192	0.671964906
MAPK3	0.018981688	0.671989388
PRKCA	0.018887929	0.673515992
OSBPL9	-0.018100815	0.686384384
IL1B	0.017066472	0.703432927
WWTR1	-0.016259228	0.716842892
YY1AP1	-0.015694097	0.72628324
PRKAA1	-0.01567649	0.726578033
LCE2C	0.014649376	0.74384361
MIOX	-0.014394251	0.748152592
GPX4	-0.013438526	0.764363273
MAP3K11	0.013347535	0.76591214
NGB	-0.013112395	0.76991904
BRD7	0.012965589	0.772423814
DECR1	-0.012514404	0.780136525
MIR424	-0.012002205	0.788918402
SLC2A12	-0.01132938	0.800494883
MTCH1	-0.010899031	0.807922514
MIR9-3	0.010700817	0.811349472
POM121L12	0.01043749	0.815907733
AKR1C3	0.010234987	0.819417359
HIC1	-0.010150333	0.820885579
OTUB1	0.010048052	0.822660373
ATG7	-0.007598988	0.865406747

CDH1	-0.006981127	0.87625851
PROM2	-0.006473094	0.885198815
MIR522	-0.005736136	0.898193348
RHEBP1	-0.004981363	0.911530251
HCAR1	-0.004198081	0.925397187
CYP4F8	-0.00383994	0.931745383
BRD3	0.002629461	0.953231298
NOX3	-0.002467561	0.956107928
EMC2	-0.002277069	0.959493312
BECN1	-0.002196002	0.960934242
ALOX12B	-0.000166567	0.997035678
GABPB1	0.000124729	0.997780244

Table S7. The exhaustively screening of the immune cell types in the tumor infiltration process in the high- and low-risk groups by integrating eight mainstream immunoinformatic algorithms.

Immune cells	*p*	Correlation coefficient (If available)
**Wilcoxon rank-sum tested between the high- and low-risk groups**		
MHC_IPS	0.028100171	NA
EC_IPS	0.156954737	NA
SC_IPS	0.057308841	NA
CP_IPS	0.634562767	NA
AZ_IPS	0.091655219	NA
IPS_IPS	0.302686689	NA
B cell_TIMER	2.14E-14	NA
T cell CD4+_TIMER	1.49E-09	NA
T cell CD8+_TIMER	0.000793445	NA
Neutrophil_TIMER	0.023648402	NA
Macrophage_TIMER	0.010191665	NA
Myeloid dendritic cell_TIMER	1.11E-05	NA
B cell naive_CIBERSORT	0.93674187	NA
B cell memory_CIBERSORT	7.94E-08	NA
B cell plasma_CIBERSORT	0.056529259	NA
T cell CD8+_CIBERSORT	0.758100136	NA
T cell CD4+ naive_CIBERSORT	0.31828033	NA
T cell CD4+ memory resting_CIBERSORT	9.91E-06	NA
T cell CD4+ memory activated_CIBERSORT	0.000290739	NA
T cell follicular helper_CIBERSORT	0.983404528	NA
T cell regulatory (Tregs)_CIBERSORT	0.672174373	NA
T cell gamma delta_CIBERSORT	0.667631366	NA
NK cell resting_CIBERSORT	0.14759909	NA
NK cell activated_CIBERSORT	0.711048316	NA
Monocyte_CIBERSORT	0.000196192	NA
Macrophage M0_CIBERSORT	4.81E-10	NA
Macrophage M1_CIBERSORT	0.120471524	NA
Macrophage M2_CIBERSORT	0.263846287	NA
Myeloid dendritic cell resting_CIBERSORT	3.60E-05	NA
Myeloid dendritic cell activated_CIBERSORT	0.91533603	NA
Mast cell activated_CIBERSORT	4.59E-12	NA
Mast cell resting_CIBERSORT	1.29E-09	NA
Eosinophil_CIBERSORT	0.559013204	NA
Neutrophil_CIBERSORT	0.016577584	NA
B cell naive_CIBERSORT-ABS	0.12711394	NA
B cell memory_CIBERSORT-ABS	2.16E-09	NA
B cell plasma_CIBERSORT-ABS	0.620722086	NA
T cell CD8+_CIBERSORT-ABS	0.000181005	NA
T cell CD4+ naive_CIBERSORT-ABS	0.31828033	NA
T cell CD4+ memory resting_CIBERSORT-ABS	9.60E-11	NA
T cell CD4+ memory activated_CIBERSORT-ABS	0.000642965	NA

T cell follicular helper_CIBERSORT-ABS	9.78E-05	NA
T cell regulatory (Tregs)_CIBERSORT-ABS	0.030627515	NA
T cell gamma delta_CIBERSORT-ABS	0.787535241	NA
NK cell resting_CIBERSORT-ABS	0.223569919	NA
NK cell activated_CIBERSORT-ABS	0.001738691	NA
Monocyte_CIBERSORT-ABS	2.31E-07	NA
Macrophage M0_CIBERSORT-ABS	0.000119066	NA
Macrophage M1_CIBERSORT-ABS	0.222250664	NA
Macrophage M2_CIBERSORT-ABS	1.58E-08	NA
Myeloid dendritic cell resting_CIBERSORT-ABS	8.85E-07	NA
Myeloid dendritic cell activated_CIBERSORT-ABS	0.473157978	NA
Mast cell activated_CIBERSORT-ABS	4.74E-17	NA
Mast cell resting_CIBERSORT-ABS	2.36E-08	NA
Eosinophil_CIBERSORT-ABS	0.559013204	NA
Neutrophil_CIBERSORT-ABS	0.141522517	NA
B cell_QUANTISEQ	4.10E-09	NA
Macrophage M1_QUANTISEQ	0.761882871	NA
Macrophage M2_QUANTISEQ	3.07E-14	NA
Monocyte_QUANTISEQ	0.850327905	NA
Neutrophil_QUANTISEQ	0.993064168	NA
NK cell_QUANTISEQ	0.00029389	NA
T cell CD4+ (non-regulatory)_QUANTISEQ	0.872781478	NA
T cell CD8+_QUANTISEQ	0.003123933	NA
T cell regulatory (Tregs)_QUANTISEQ	2.88E-08	NA
Myeloid dendritic cell_QUANTISEQ	0.499990211	NA
uncharacterized cell_QUANTISEQ	3.58E-05	NA
T cell_MCPCOUNTER	1.18E-08	NA
T cell CD8+_MCPCOUNTER	0.002387614	NA
cytotoxicity score_MCPCOUNTER	0.206271839	NA
NK cell_MCPCOUNTER	0.043366318	NA
B cell_MCPCOUNTER	5.97E-08	NA
Monocyte_MCPCOUNTER	0.22042965	NA
Macrophage/Monocyte_MCPCOUNTER	0.22042965	NA
Myeloid dendritic cell_MCPCOUNTER	3.99E-17	NA
Neutrophil_MCPCOUNTER	8.13E-07	NA
Endothelial cell_MCPCOUNTER	1.17E-06	NA
Cancer associated fibroblast_MCPCOUNTER	0.000825896	NA
Myeloid dendritic cell activated_XCELL	2.06E-07	NA
B cell_XCELL	4.75E-07	NA
T cell CD4+ memory_XCELL	0.301894382	NA
T cell CD4+ naive_XCELL	6.72E-07	NA
T cell CD4+ (non-regulatory)_XCELL	0.254714439	NA
T cell CD4+ central memory_XCELL	2.80E-09	NA
T cell CD4+ effector memory_XCELL	5.53E-05	NA
T cell CD8+ naive_XCELL	0.995706203	NA
T cell CD8+_XCELL	7.86E-10	NA
T cell CD8+ central memory_XCELL	1.63E-05	NA
T cell CD8+ effector memory_XCELL	0.167791387	NA

Class-switched memory B cell_XCELL	6.06E-12	NA
Common lymphoid progenitor_XCELL	1.89E-06	NA
Common myeloid progenitor_XCELL	6.97E-06	NA
Myeloid dendritic cell_XCELL	2.73E-14	NA
Endothelial cell_XCELL	0.001166394	NA
Eosinophil_XCELL	1.48E-05	NA
Cancer associated fibroblast_XCELL	4.54E-06	NA
Granulocyte-monocyte progenitor_XCELL	7.56E-08	NA
Hematopoietic stem cell_XCELL	4.83E-16	NA
Macrophage_XCELL	0.006709293	NA
Macrophage M1_XCELL	0.928753277	NA
Macrophage M2_XCELL	2.02E-09	NA
Mast cell_XCELL	2.23E-10	NA
B cell memory_XCELL	2.61E-05	NA
Monocyte_XCELL	0.00036842	NA
B cell naive_XCELL	0.00025507	NA
Neutrophil_XCELL	0.851107243	NA
NK cell_XCELL	0.849974887	NA
T cell NK_XCELL	0.002045931	NA
Plasmacytoid dendritic cell_XCELL	0.976965728	NA
B cell plasma_XCELL	0.436743838	NA
T cell gamma delta_XCELL	0.569485376	NA
T cell CD4+ Th1_XCELL	2.84E-05	NA
T cell CD4+ Th2_XCELL	1.46E-15	NA
T cell regulatory (Tregs)_XCELL	0.905099357	NA
immune score_XCELL	2.58E-13	NA
stroma score_XCELL	2.55E-06	NA
microenvironment score_XCELL	5.27E-15	NA
B cell_EPIC	8.91E-07	NA
Cancer associated fibroblast_EPIC	3.66E-06	NA
T cell CD4+_EPIC	9.87E-06	NA
T cell CD8+_EPIC	3.08E-13	NA
Endothelial cell_EPIC	2.41E-05	NA
Macrophage_EPIC	0.004216259	NA
NK cell_EPIC	0.01254569	NA
uncharacterized cell_EPIC	0.090112933	NA
**Pearson correlation with the risk score**		
MHC_IPS	0.017214013	-0.106600974
EC_IPS	0.263049159	-0.050197167
SC_IPS	0.068766015	0.081541113
CP_IPS	0.84514285	0.008765516
AZ_IPS	0.119709969	-0.069745844
IPS_IPS	0.277239199	-0.048733622
B cell_TIMER	5.67E-17	-0.362840665
T cell CD4+_TIMER	3.87E-13	-0.317355559
T cell CD8+_TIMER	0.000847141	-0.148920501
Neutrophil_TIMER	0.10705766	-0.072230236
Macrophage_TIMER	0.0102384	-0.114852227

Myeloid dendritic cell_TIMER	3.22E-05	-0.184970485
B cell naive_CIBERSORT	0.277382683	0.048719087
B cell memory_CIBERSORT	3.86E-11	-0.290214203
B cell plasma_CIBERSORT	0.208744589	0.056368785
T cell CD8+_CIBERSORT	0.638204798	-0.021100076
T cell CD4+ naive_CIBERSORT	0.417729167	-0.036356201
T cell CD4+ memory resting_CIBERSORT	1.32E-06	-0.214548304
T cell CD4+ memory activated_CIBERSORT	0.000972644	0.147217124
T cell follicular helper_CIBERSORT	0.907838908	-0.005195341
T cell regulatory (Tregs)_CIBERSORT	0.417174737	-0.036399481
T cell gamma delta_CIBERSORT	0.648566187	-0.020451551
NK cell resting_CIBERSORT	0.02320549	0.101612386
NK cell activated_CIBERSORT	0.072170181	0.080562632
Monocyte_CIBERSORT	9.91E-05	-0.173376231
Macrophage M0_CIBERSORT	7.66E-13	0.313503501
Macrophage M1_CIBERSORT	0.047992974	0.088570044
Macrophage M2_CIBERSORT	0.349849669	-0.04193778
Myeloid dendritic cell resting_CIBERSORT	0.001558568	-0.141263496
Myeloid dendritic cell activated_CIBERSORT	0.019818978	0.104271329
Mast cell activated_CIBERSORT	3.71E-15	-0.342227277
Mast cell resting_CIBERSORT	5.70E-11	0.287779394
Eosinophil_CIBERSORT	0.138498816	-0.066410098
Neutrophil_CIBERSORT	0.000637167	0.152377331
B cell naive_CIBERSORT-ABS	0.01703693	-0.106770291
B cell memory_CIBERSORT-ABS	3.00E-11	-0.291780674
B cell plasma_CIBERSORT-ABS	0.020527488	-0.103684043
T cell CD8+_CIBERSORT-ABS	6.05E-06	-0.200988644
T cell CD4+ naive_CIBERSORT-ABS	0.417729167	-0.036356201
T cell CD4+ memory resting_CIBERSORT-ABS	1.02E-13	-0.32472084
T cell CD4+ memory activated_CIBERSORT-ABS	0.063637806	0.083092933
T cell follicular helper_CIBERSORT-ABS	5.58E-09	-0.257174617
T cell regulatory (Tregs)_CIBERSORT-ABS	1.22E-05	-0.194407241
T cell gamma delta_CIBERSORT-ABS	0.231695354	-0.05363642
NK cell resting_CIBERSORT-ABS	0.479858373	0.031700195
NK cell activated_CIBERSORT-ABS	0.002725824	-0.133898725
Monocyte_CIBERSORT-ABS	4.16E-07	-0.22424884
Macrophage M0_CIBERSORT-ABS	0.002599028	0.134540246
Macrophage M1_CIBERSORT-ABS	0.064533963	-0.082814515
Macrophage M2_CIBERSORT-ABS	5.59E-09	-0.257164964
Myeloid dendritic cell resting_CIBERSORT-ABS	3.68E-06	-0.20549591
Myeloid dendritic cell activated_CIBERSORT-ABS	0.82439375	0.00995822
Mast cell activated_CIBERSORT-ABS	2.42E-23	-0.425341366
Mast cell resting_CIBERSORT-ABS	5.11E-05	0.180293653
Eosinophil_CIBERSORT-ABS	0.152058426	-0.064213339
Neutrophil_CIBERSORT-ABS	0.148776988	0.064730805
B cell_QUANTISEQ	9.71E-13	-0.312154188
Macrophage M1_QUANTISEQ	0.409744825	0.036982774
Macrophage M2_QUANTISEQ	4.72E-17	-0.363707316

Monocyte_QUANTISEQ	0.986650295	-0.000750917
Neutrophil_QUANTISEQ	0.489627868	0.030999278
NK cell_QUANTISEQ	1.00E-06	-0.216883178
T cell CD4+ (non-regulatory)_QUANTISEQ	0.027111443	0.098934439
T cell CD8+_QUANTISEQ	0.002446035	-0.135353431
T cell regulatory (Tregs)_QUANTISEQ	1.07E-12	-0.311606659
Myeloid dendritic cell_QUANTISEQ	0.074045131	0.080039705
uncharacterized cell_QUANTISEQ	0.000113251	0.171952849
T cell_MCPCOUNTER	1.71E-12	-0.308913559
T cell CD8+_MCPCOUNTER	0.008777847	-0.117202966
cytotoxicity score_MCPCOUNTER	0.665995325	-0.019370103
NK cell_MCPCOUNTER	0.479174828	0.031749524
B cell_MCPCOUNTER	1.69E-09	-0.265527837
Monocyte_MCPCOUNTER	0.296285077	0.046847059
Macrophage/Monocyte_MCPCOUNTER	0.296285077	0.046847059
Myeloid dendritic cell_MCPCOUNTER	7.09E-15	-0.33889475
Neutrophil_MCPCOUNTER	7.59E-08	-0.237829192
Endothelial cell_MCPCOUNTER	1.69E-08	-0.249142701
Cancer associated fibroblast_MCPCOUNTER	9.12E-08	0.236395273
Myeloid dendritic cell activated_XCELL	9.37E-08	-0.236192276
B cell_XCELL	8.58E-12	-0.299399808
T cell CD4+ memory_XCELL	0.263769529	-0.050121583
T cell CD4+ naive_XCELL	7.01E-11	-0.286476861
T cell CD4+ (non-regulatory)_XCELL	0.132643111	-0.067410023
T cell CD4+ central memory_XCELL	5.47E-13	-0.315413771
T cell CD4+ effector memory_XCELL	3.54E-08	-0.243651957
T cell CD8+ naive_XCELL	0.817865173	0.010334912
T cell CD8+_XCELL	1.30E-09	-0.267328054
T cell CD8+ central memory_XCELL	1.16E-06	-0.215665069
T cell CD8+ effector memory_XCELL	0.011617095	-0.112892144
Class-switched memory B cell_XCELL	8.01E-18	-0.371996242
Common lymphoid progenitor_XCELL	1.09E-08	0.252371206
Common myeloid progenitor_XCELL	1.20E-07	-0.234269537
Myeloid dendritic cell_XCELL	7.74E-14	-0.326231119
Endothelial cell_XCELL	9.36E-05	-0.173981931
Eosinophil_XCELL	7.25E-09	-0.255313317
Cancer associated fibroblast_XCELL	1.14E-08	-0.252022103
Granulocyte-monocyte progenitor_XCELL	2.22E-08	-0.247151939
Hematopoietic stem cell_XCELL	3.58E-24	-0.43258508
Macrophage_XCELL	0.002713221	-0.13396126
Macrophage M1_XCELL	0.805797097	-0.011033165
Macrophage M2_XCELL	5.54E-09	-0.257226388
Mast cell_XCELL	1.36E-14	-0.335503233
B cell memory_XCELL	7.18E-09	-0.255383594
Monocyte_XCELL	8.04E-05	-0.175584498
B cell naive_XCELL	0.000106193	-0.172641487
Neutrophil_XCELL	0.540385728	0.027470891
NK cell_XCELL	0.86046538	-0.007888735

T cell NK_XCELL	8.27E-05	-0.175293804
Plasmacytoid dendritic cell_XCELL	0.242947112	-0.052366749
B cell plasma_XCELL	0.012887331	-0.11126068
T cell gamma delta_XCELL	0.485509155	-0.031293848
T cell CD4+ Th1_XCELL	8.95E-06	0.197352279
T cell CD4+ Th2_XCELL	1.44E-18	0.379789461
T cell regulatory (Tregs)_XCELL	0.502805011	-0.030065646
immune score_XCELL	9.39E-17	-0.360432624
stroma score_XCELL	7.45E-09	-0.255119122
microenvironment score_XCELL	3.57E-19	-0.385968829
B cell_EPIC	3.50E-08	-0.243738676
Cancer associated fibroblast_EPIC	3.44E-08	0.243860922
T cell CD4+_EPIC	2.77E-06	-0.208052845
T cell CD8+_EPIC	1.26E-09	-0.267533656
Endothelial cell_EPIC	1.71E-08	-0.249092178
Macrophage_EPIC	0.000713954	-0.151005105
NK cell_EPIC	0.842158025	-0.008936691
uncharacterized cell_EPIC	0.581056538	0.024762158

Table S8. Lists of drugs in CTRP and PRISM.

CTRP	PRISM
968	10-deacetylbaccatin
16-beta-bromoandrosterone	10-hydroxycamptothecin
1S,3R-RSL-3	12-O-tetradecanoylphorbol-13-acetate
3-Cl-AHPC	1-azakenpaullone
5-fluorouracil	1-naphthyl-PP1
A-804598	1-phenylbiguanide
AA-COCF3	2,3-DCPE
abiraterone	2-MeCCPA
ABT-199	2-methoxyestradiol
ABT-737	3-amino-benzamide
AC55649	3-deazaneplanocin-A
afatinib	3-fluorobenzylspiperone
AGK-2	4-(4-fluorobenzoyl)-1-(4-phenylbutyl)-piperidine
alisertib	4-chlorophenylguanidine
alvocidib	4E1RCat
AM-580	4-hydroxy-phenazone
apicidin	4-iodo-6-phenylpyrimidine
AT13387	4-methylgenistein
AT-406	4-methylhistamine
AT7867	4-pyrimidinecarbonitrile
austocystin D	5-fluorouracil
avicin D	5-hydroxymethyl-tolterodine
avrainvillamide	7-aminocephalosporanic-acid
axitinib	7-hydroxystaurosporine
AZ-3146	7-nitroindazole
azacitidine	8-bromo-cGMP
AZD1480	8-hydroxy-PIPAT
AZD4547	9-aminoacridine
AZD6482	A61603
AZD7545	A-674563
AZD7762	A-803467
AZD8055	abemaciclib
B02	abiraterone
bafilomycin A1	abiraterone-acetate
barasertib	ABT-702
bardoxolone methyl	ABT-737
Bax channel blocker	ABT-751
BCL-LZH-4	AC-264613
BEC	ACDPP
belinostat	acesulfame-potassium

bendamustine
betulinic acid
bexarotene
BI-2536
BIBR-1532
BIRB-796
birinapant
BIX-01294
blebbistatin
bleomycin A2
BMS-195614
BMS-270394
BMS-345541
BMS-536924
BMS-754807
bortezomib
bosutinib
BRD1812
BRD1835
BRD4132
BRD6340
BRD8899
BRD8958
BRD9647
BRD9876
BRD-A02303741
BRD-A05715709
BRD-A71883111
BRD-A86708339
BRD-A94377914
BRD-K01737880
BRD-K02251932
BRD-K02492147
BRD-K04800985
BRD-K09344309
BRD-K09587429
BRD-K11533227
BRD-K13999467
BRD-K14844214
BRD-K16147474
BRD-K17060750
BRD-K19103580
BRD-K24690302

acetazolamide
acetophenazine
acetylcysteine
acetylsalicylsalicylic-acid
acipimox
acivicin
ACY-1215
adapalene
adaprev
adarotene
adefovir-dipivoxil
ADL5859
AEE788
AEG3482
afatinib
afobazole
AG-14361
ajmaline
albendazole
alectinib
alexidine
alfacalcidol
alisertib
almorexant
alogliptin
alpelisib
altretamine
alvespimycin
alvocidib
AM-251
AM-404
AM-580
ambazone
amfenac
AMG-208
AMG-232
AMG458
AMG900
aminoglutethimide
aminopurvalanol-a
amisulpride
ammonium-lactate
amonafide

BRD-K26531177	amoxicillin
BRD-K27224038	amprolium
BRD-K27986637	amsacrine
BRD-K28456706	anagrelide
BRD-K29086754	anastrozole
BRD-K29313308	anguidine
BRD-K30019337	anisomycin
BRD-K30748066	AP1903
BRD-K33199242	AP26113
BRD-K33514849	apatinib
BRD-K34099515	aphidicolin
BRD-K34222889	AR-12
BRD-K34485477	AR-42
BRD-K35604418	arctigenin
BRD-K37390332	arcyriaflavin-a
BRD-K41334119	aripiprazole
BRD-K41597374	ARRY-334543
BRD-K42260513	artemisinin
BRD-K44224150	artesunate
BRD-K45681478	AS-1949490
BRD-K48334597	AS-703026
BRD-K48477130	aspirin
BRD-K49290616	AST-1306
BRD-K50799972	astemizole
BRD-K51490254	asymmetrical-dimethylarginine
BRD-K51831558	AT13387
BRD-K52037352	AT-7519
BRD-K55116708	AT7867
BRD-K61166597	AT-9283
BRD-K63431240	atenolol-(-)
BRD-K64610608	atenolol-(+/-)
BRD-K66453893	atipamezole
BRD-K66532283	atiprimod
BRD-K70511574	atorvastatin
BRD-K71781559	aurora-a-inhibitor-i
BRD-K71935468	AV-412
BRD-K75293299	avanafil
BRD-K78574327	AVL-292
BRD-K80183349	AVN-944
BRD-K84807411	axitinib
BRD-K85133207	AZ20
BRD-K86535717	AZ3146
BRD-K88742110	AZ-628

BRD-K90370028
BRD-K92856060
BRD-K94991378
BRD-K96431673
BRD-K96970199
BRD-K97651142
BRD-K99006945
BRD-M00053801
brefeldin A
brivanib
BYL-719
C6-ceramide
cabozantinib
canertinib
CAY10576
CAY10594
CAY10618
CBB-1007
CCT036477
CD-1530
CD-437
cediranib
ceranib-2
cerulenin
Ch-55
CHIR-99021
chlorambucil
CHM-1
CI-976
ciclopirox
ciclosporin
CID-5951923
CIL41
CIL55
CIL55A
CIL56
CIL70
cimetidine
clofarabine
COL-3
Compound 1541A
compound 1B
Compound 23 citrate
AZ960
azacitidine
azalomycin-b
azathioprine
AZD1480
AZD2014
AZD2461
AZD2858
AZD3463
AZD4547
AZD5363
AZD5438
AZD6482
AZD7762
AZD8055
AZD8330
AZD8931
AZD9668
azilsartan
azithromycin
azodicarbonamide
azomycin-(2-nitroimidazole)
aztreonam
bafetinib
baicalein
baicalin
balapiravir
BAM7
BAN-ORL-24
barasertib
barasertib-HQPA
bardoxolone
bardoxolone-methyl
baricitinib
batimastat
BAY-11-7082
BAY-11-7085
BAY-87-2243
BAY-K-8644-(s)-(-)
beclomethasone-dipropionate
bekanamycin
belinostat
bendamustine

Compound 7d-cis
CR-1-31B
crizotinib
cucurbitacin I
curcumin
cyanoquinoline 11
cyclophosphamide
cytarabine hydrochloride
cytochalasin B
dabrafenib
dacarbazine
darinaparsin
dasatinib
DBeQ
decitabine
dexamethasone
dinaciclib
docetaxel
doxorubicin
ELCPK
elocalcitol
entinostat
epigallocatechin-3-monogallate
erastin
erismodegib
erlotinib
etomoxir
etoposide
ETP-46464
EX-527
FGIN-1-27
fingolimod
FK-866
fluvastatin
foretinib
FQI-1
FQI-2
FSC231
fulvestrant
fumonisin B1
GANT-61
GDC-0879
GDC-0941

bendroflumethiazide
benfotiamine
bentazepam
benzethonium
benzocaine
benzydamine
benzyldimethylhexadecylammonium
bephenium-hydroxynaphthoate
berberine
beta-lapachone
betamethasone
betamethasone-dipropionate
beta-naphthol
betulinic-acid
bexarotene
BF2.649
BGT226
BI-2536
BI-78D3
BIBR-1532
BIBU-1361
BIBX-1382
bicalutamide
BI-D1870
bifemelane
BIIB021
bindarit
birinapant
bis(maltolato)oxovanadium(IV)
bisacodyl
bisindolylmaleimide-ix
bitopertin
BIX-01294
BIX-02188
blebbistatin-(-)
BMS-265246
BMS-345541
BMS-387032
BMS-599626
BMS-626529
BMS-690514
BMS-707035
BMS-754807

gefitinib
gemcitabine
GMX-1778
gossypol
GSK1059615
GSK2636771
GSK-3 inhibitor IX
GSK4112
GSK461364
GSK-J4
GW-405833
GW-843682X
HBX-41108
HC-067047
HLI 373
hyperforin
I-BET151
I-BET-762
ibrutinib
IC-87114
idelalisib
ifosfamide
imatinib
importazole
indisulam
IPR-456
isoevodiamine
isoliquiritigenin
isonicotinohydroxamic acid
ISOX
istradefylline
itraconazole
IU1
JQ-1
JW-480
JW-55
JW-74
KH-CB19
KHS101
Ki8751
Ko-143
KPT185
KU 0060648

BMS-986020
BNC105
BNTX
bortezomib
bosentan
bosutinib
brefeldin-a
brequinar
brigatinib
brilliant-green
brivaracetam
bromosporine
broxaldine
broxyquinoline
bruceantin
BTS-54505
bucladesine
bumetanide
buparlisib
buphenine
busulfan
butamben
buthionine-sulfoximine
butylscopolamine-bromide
BVD-523
BVT-948
BW-180C
BX-912
cabazitaxel
cabozantinib
caffeic-acid
caffeic-acid-phenethyl-ester
calcitriol
camptothecin
camylofine-chlorhydrate
candesartan
canertinib
canrenone
capecitabine
capsaicin
captamine
carbachol
carboplatin

KU-0063794
KU-55933
KU-60019
KW-2449
KX2-391
L-685458
lapatinib
LBH-589
LE-135
lenvatinib
leptomycin B
linifanib
linsitinib
lomeguatrib
lovastatin
LRRK2-IN-1
LY-2157299
LY-2183240
manumycin A
marinopyrrole A
masitinib
Mdivi-1
Merck60
methotrexate
methylstat
MG-132
MGCD-265
MI-1
MI-2
mitomycin-c
MK-0752
MK-1775
MK-2206
ML006
ML029
ML031
ML050
ML083
ML162
ML203
ML210
ML239
ML258
carboxyamidotriazole
carboxypyridine-disulfide
carfilzomib
cariporide
carmofur
carmustine
carvedilol
CB-10-277
CCG-50014
CCMI
CCT128930
CCT129202
CCT137690
CD-437
cebranopadol
cediranib
cefdinir
cefditoren-pivoxil
cefpiramide
CEP-32496
CEP-33779
CEP-37440
cephalomannine
cetrimonium
cetylpyridinium
CGM097
CGP-37849
CGP-52411
CGP-54626
CGS-15943
CGS-20625
CH5132799
chicago-sky-blue-6b
chidamide
CHIR-124
CHIR-98014
CHIR-99021
chlorambucil
chloramphenicol-palmitate
chlorhexidine
chlorindanol
chlormidazole
chlorogenic-acid

ML311	chloropyramine
ML312	chloroxine
ML320	chlorpropamide
ML334 diastereomer	chlorpyrifos
MLN2238	chlorquinaldol
MLN2480	cholecalciferol
momelotinib	chromocarb
MST-312	CI-844
myricetin	cibenzoline
myriocin	ciclesonide
N9-isopropylolomoucine	ciclopirox
nakiterpiosin	CID-5458317
narciclasine	cidofovir
navitoclax	cilostamide
necrostatin-1	cimetidine
necrostatin-7	cinacalcet
necrosulfonamide	cinalukast
nelarabine	cinnarazine
neopeltolide	cinromide
neratinib	ciprofibrate
neuronal differentiation inducer III	cisplatin
niclosamide	CL-218872
nilotinib	cladribine
nintedanib	clebopride
NPC-26	clenbuterol
NSC 74859	clindamycin-phosphate
NSC19630	clobetasol-propionate
NSC23766	clobutinol
NSC30930	clocortolone-pivalate
NSC48300	clofarabine
NSC632839	clofazimine
NSC95397	clofibrate
nutlin-3	clofoctol
NVP-231	clomifene
NVP-ADW742	clonazepam
NVP-BEZ235	clopamide
NVP-BSK805	clotrimazole
NVP-TAE684	CMPD-1
O-6-benzylguanine	CNX-2006
obatoclax	CNX-774
olaparib	cobicistat
oligomycin A	cobimetinib

omacetaxine mepesuccinate
OSI-027
OSI-930
ouabain
PAC-1
paclitaxel
palmostatin B
pandacostat
parbendazole
parthenolide
pazopanib
PD 153035
PD318088
PDMP
pevonedistat
PF-184
PF-3758309
PF-4800567 hydrochloride
PF-543
PF-573228
PF-750
PHA-793887
phloretin
PI-103
pifithrin-alpha
pifithrin-mu
PIK-93
piperlongumine
pitstop2
PL-DI
pluripotin
PLX-4032
PLX-4720
PRIMA-1
PRIMA-1-Met
PRL-3 inhibitor I
procarbazine
prochlorperazine
purmorphamine
PX-12
PYR-41
pyrazolanthrone
QS-11

colchicine
colforsin-daproate
colfosceril-palmitate
colistin-b-sulfate
combretastatin-A-4
costunolide
CP-673451
CP-724714
CP-945,598
CPI-1189
CPP
CR8-(R)
creatine
crenolanib
crizotinib
crizotinib-(S)
cromakalim
crystal-violet
CS-110266
CUDC-101
CUDC-907
curcumin
CX-4945
CX-5461
CYC116
cyclocytidine
cycloheximide
cyclophosphamide
cyclosporin-a
cyclosporine
cyclovalone
cyclovirobuxin-d
cyproheptadine
cyproterone-acetate
cyromazine
cyt387
CYT-997
cytarabine
cytochalasin-b
cytochlor
D-4476
D-64131
D-7193

quizartinib
QW-BI-011
R428
RAF265
regorafenib
Repligen 136
RG-108
rigosertib
RITA
RO4929097
ruxolitinib
salermide
saracatinib
SB-225002
SB-431542
SB-525334
SB-743921
SCH-529074
SCH-79797
selumetinib
semagacestat
serdemetan
SGX-523
SID 26681509
sildenafil
silmitasertib
simvastatin
sirolimus
sitagliptin
SJ-172550
skepinone-L
SKI-II
SMER-3
SN-38
SNS-032
SNX-2112
sorafenib
sotrastaurin
spautin-1
SR1001
SR8278
SR-II-138A
SRT-1720

dabrafenib
dacarbazine
dacinostat
daclatasvir
dacomitinib
dalcetrapib
danazol
danusertib
dapivirine
darapladib
darifenacin
dasatinib
daunorubicin
DCEBIO
decitabine
defactinib
deferasirox
deflazacort
deforolimus
dehydrocholate-acid
delanzomib
demecarium
deoxycorticosterone-acetate
dequalinium
desonide
desoxycortone
dexamethasone-acetate
dexrazoxane
dianhydrogalactitol
diaveridine
diazooxonorleucine
dichlorisone-acetate
dichloroacetate
dichlorvos
diclazuril
dicycloverine
dienogest
difluprednate
digitoxigenin
digitoxin
digoxigenin
digoxin
dihydroartemisinin

staurosporine
StemRegenin 1
STF-31
SU11274
sunitinib
SZ4TA2
tacedinaline
tacrolimus
tamatinib
tamoxifen
tandutinib
tanespimycin
temozolomide
temsirolimus
teniposide
TG-100-115
TG-101348
TGX-221
thalidomide
tigecycline
tipifarnib
tipifarnib-P2
tivantinib
tivozanib
topotecan
tosedostat
tozasertib
TPCA-1
trametinib
tretinoin
triazolothiadiazine
trifluoperazine
triptolide
tubastatin A
TW-37
UNC0321
UNC0638
VAF-347
valdecoxib
vandetanib
veliparib
VER-155008
vincristine
dihydroergocristine
dihydromyricetin
dimethisoquin
dinaciclib
dioscin
diphemanil
diphenyleneiodonium
dirithromycin
disulfiram
DMH1
docetaxel
dofetilide
dolastatin-10
dolutegravir
domiphen
domperidone
dorzolamide
dovitinib
doxercalciferol
doxifluridine
doxorubicin
doxycycline
DPI-201106
dronedarone
drospirenone
D-Serine
DU-728
DVD-111
E7449
ebastine
ecamsule-triethanolamine
echinomycin
efonidipine-monoethanolate
elacridar
elesclomol
eltrombopag
embelin
EMD-53998
emetine
endo-IWR-1
ENMD-2076
enocitabine
enoximone

vorapaxar
vorinostat
VU0155056
WAY-362450
WP1130
WZ4002
WZ8040
XL765
YK 4-279
YM-155
zebularine
ZSTK-474

entinostat
enzalutamide
epiandrosterone
epinastine
epinephrine
epirubicin
eplerenone
epothilone-a
epothilone-b
epothilone-d
eprinomectin
eprobemide
eprosartan
eptifibatide
EPZ-5676
equol
ER-27319
erastin
ercalcitriol
erdafitinib
erlotinib
erteberel
erythritol
estradiol
estradiol-benzoate
estradiol-cypionate
estramustine
estramustine-phosphate
estrone
ethacridine-lactate-monohydrate
ethinyl-estradiol
etofylline-clofibrate
etomoxir
etoposide
etoposide-phosphate
ETP-46464
evacetrapib
everolimus
evodiamine
EVP4593
exatecan-mesylate
exemestane
famciclovir

famotidine
favipiravir
fdcyd
felbamate
fenaclon
fenbendazole
fendiline
fenofibrate
fenoprofen
fenretinide
FERb-033
fexinidazole
FG-4592
filanesib
filgotinib
finafloxacin
FK-3311
FK-866
FK-888
fleroxacin
FLI-06
florfenicol
floxuridine
flubendazole
fludarabine
fludarabine-phosphate
fludroxycortide
flufenamic-acid
flumatinib
flumethasone
flumethasone-pivalate
fluocinolone-acetonide
fluorometholone
fluoromethylcholine
fluroxene
flutamide
fluticasone-propionate
fluvastatin
foretinib
formestane
forodesine
forskolin
fosbretabulin

foscarnet
FPH1-(BRD-6125)
FR-122047
FR-139317
frentizole
ftorafur
fudosteine
fulvestrant
G-1
galeterone
gambogic-acid
gamma-aminobutyric-acid
ganetespib
gastrodin
GDC-0068
GDC-0152
GDC-0349
GDC-0879
GDC-0941
GDC-0980
gefitinib
gemcitabine
genipin
genz-644282
gestrinone
GGTI-298
gidazepam
gilteritinib
gimeracil
ginkgolide-a
givinostat
golgicide-a
golvatinib
gossypol
GS-39783
GS-9973
GSK1070916
GSK1292263
GSK1838705A
GSK1904529A
GSK2110183
GSK2126458
GSK256066

GSK2656157
GSK2830371
GSK-3-inhibitor-IX
GSK429286A
GSK461364
GSK650394
GSK923295
GSK-J4
GTP-14564
guanidine
GW-3965
GW-405833
GW-441756
GW-501516
GW-583340
GW-788388
GW-842166
GW-843682X
GZD824
HA14-1
halcinonide
halobetasol-propionate
halofantrine
halofuginone
harringtonine
hesperadin
hexachlorophene
hexaminolevulinate
hexylresorcinol
HMN-214
homoharringtonine
homoquinolinic-acid
homosalate
hydrocortisone
hydroxytacrine-maleate-(r,s)
hydroxyurea
hyodeoxycholic-acid
hyoscyamine
hypericin
hypoestoxide
I-BET151
I-BET-762
ibrutinib

ibutilide
ICG-001
ICI-162846
icotinib
idarubicin
idasanutlin
idazoxan
idelalisib
idoxuridine
IDRA-21
idronoxil
IEM1754
ifosfamide
IKK-16
IKK-2-inhibitor-V
ilomastat
imatinib
imidapril
imiloxan
imiquimod
INC-280
indibulin
indiplon
indirubin
indisulam
indoprofen
ingenol-mebutate
INH1
inosine
iobenguane
iodipamide
iododexetimide
IOWH032
iproniazid
ipsapirone
irinotecan
isoetharine
isofloxythepin
isoflupredone-acetate
isometheptene-mucate
isopropyl-myristate
isoxsuprine
ispinesib

istradefylline
itraconazole
ivermectin
ixabepilone
ixazomib
ixazomib-citrate
JIB04
JK-184
JNJ-16259685
JNJ-26481585
JNJ-7706621
josamycin
JQ1
JTC-801
JTE-607
JZL-184
K-858
KD-023
KD025
kenpaullone
ketoconazole
ketoprofen
KF-38789
KI-16425
KI-8751
kifunensine
KP-1212
KPT-185
KPT-276
K-Ras(G12C)-inhibitor-6
k-strophanthidin
ku-0063794
KW-2449
KW-2478
KX2-391
KY02111
kynurenic-acid
L-798,106
lacitol
lafutidine
lanatoside-c
lanoconazole
lapatinib

lappaconite
lasalocid
latanoprost
latrepirdine
LB42708
LCL-161
LDN-212854
LDN-57444
LE-135
lenalidomide
lenvatinib
lerisetron
lestaurtinib
letrozole
leucovorin
levobunolol
levocarnitine
levocetirizine
levonorgestrel
LGX818
licochalcone-a
lidamidine
lidocaine
linalool
linifanib
linsitinib
litronesib
lomefloxacin
lomitapide
lonafarnib
lorazepam
lorlatinib
lovastatin
loxistatin-acid
lucitanib
lurasidone
LY2090314
LY2109761
LY2183240
LY2334737
LY2603618
LY2606368
LY2608204

LY2784544
LY2801653
LY2874455
LY3023414
LY303511
LY364947
LY456236
M-344
maprotiline
marimastat
masitinib
maxacalcitol
maytansinol-isobutyrate
mebendazole
mechlorethamine
meclizine
mefexamide
meglitinide
MEK1-2-inhibitor
MEK162
melengestrol-acetate
meloxicam
melphalan
menadione
mephenytoin
mepivacaine
mercaptopurine
mericitabine
merimepodib
mesna
mesoridazine
metaraminol
metatinib
methacycline
methiazole
methocarbamol
methotrexate
methoxyflurane
methscopolamine
methyldopa
methylphenidate
methylprednisolone
metoprolol

metoxibutropate
metronidazole
metyrapone
mevastatin
mexeneone
mexiletine
MG-132
MGCD-265
mibampator
mibefradil
midodrine
midostaurin
mifepristone
minocycline
mitomycin-c
mitoxantrone
MK-0773
MK-0812
MK-1775
MK-2206
MK-2461
MK-3207
MK-5108
MK-8245
MK-8745
ML133
MLN0128
MLN-8054
MNS-(3,4-Methylenedioxy-nitrostyrene)
mocetinostat
monensin
monobenzone
morin
motesanib
mozavaptan
MPI-0479605
MRS-1220
MTPG
mubritinib
mycophenolate-mofetil
mycophenolic-acid
N-acetylmannosamine
nadide

nadifloxacin
nafcillin
naftidrofuryl
naftifine
naloxone
naltrexone
NAN-190
nanchangmycin
napabucasin
naproxen
narasin
narlaprevir
navarixin
navitoclax
nefiracetam
nelarabine
nemonapride
nemorubicin
neratinib
nexturastat-a
NH125
niacin
niclosamide
nicorandil
nicotine
nilotinib
nilutamide
nimorazole
nintedanib
niraparib
niridazole
nisoxetine
nitarsone
nithiamide
nitisinone
nitrocaramiphen
nizatidine
nizofenone
NMS-1286937
NMS-873
NMS-E973
nobiletin
nocodazole

nomegestrol-acetate
nonoxynol-9
norepinephrine
norethindrone
norethindrone-acetate
noretynodrel
norfloxacin
norgestrel
novobiocin
NSC-23766
NSC-319726
NSC-3852
NSC-632839
NSC-663284
NSC-697923
NU6027
nutlin-3
NVP-AEW541
NVP-AUY922
NVP-BEZ235
NVP-BHG712
NVP-BVU972
NVP-TAE226
NVP-TAE684
obatoclax
obidoxime
octenidine
octopamine
odanacatib
olaparib
OLDA
oleanolic-acid
oleoylethanolamide
oligomycin-a
oltipraz
omeprazole
ONC201
oncrasin-1
ONX-0914
oprozomib
orantinib
oridonin
ornithine

orotic-acid
oseltamivir-phosphate
OSI-027
OSI-420
OSI-930
osimertinib
OTS167
OTX015
ouabain
oxaliplatin
oxazepam
oxcarbazepine
oxibendazole
oxiperomide
oxiracetam
oxonic-acid
oxprenolol
oxymatrine
oxymetazoline
oxyphencyclimine
oxyquinoline
P22077
P276-00
P5091
PAC-1
paclitaxel
pacritinib
palbociclib
paliperidone
palmatine-chloride
palmitoylethanolamide
palomid-529
panobinostat
papaverine
parachlorophenol
parbendazole
pardoprunox
paricalcitol
parthenolide
parthenolide-(-)
pazopanib
PCI-24781
PD-0325901

PD-153035
PD-168393
PD-173074
PD-184352
PD-198306
PD-318088
PD-407824
PD-98059
pelitinib
penfluridol
penicillamine-(D)
pentagastrin
pentamidine
pentostatin
pepstatin
peruvoside
PETCM
pevonedistat
PF-03758309
PF-03814735
PF-04136309
PF-04217903
PF-04457845
PF-04691502
PF-05212384
PF-3845
PF-477736
PF-4981517
PF-562271
PF-573228
PFI-1
PFK-015
PHA-665752
PHA-680632
PHA-767491
PHA-793887
PHA-848125
phenazone
phenelzine
pheniramine
phenylbutazone
phenylmercuric-acetate
phlorizin

PI-103
PI3K-IN-2
PI-828
pibenzimol
picolinic-acid
PIK-75
pilaralisib
pilocarpine
pindolol
piperacetazine
piperazine
piperine
pirenperone
piretanide
piroxicam
pitavastatin
PJ-34
PKI-179
plinabulin
PLX-4720
podophyllotoxin
polydatin
pomalidomide
ponatinib
posaconazole
poziotinib
PP-1
PP-121
PP-2
PP242
PPT
PQ-401
PR-619
practolol
pralatrexate
pranidipine
prazosin
prednisolone
prednisolone-acetate
prednisolone-hemisuccinate
prednisolone-tebutate
primaquine
proflavine-hemisulfate

propoxycaine
propranolol
proscillaridin-a
protirelin
PRT062070
PRT062607
PSB-1115
PSI-7976
PTC-209
pterostilbene
PU-H71
puromycin
pyrimethamine
pyrithione-zinc
pyritinol
pyroxamide
quinethazone
quinidine
quizartinib
R306465
R406
R547
rabeprazole
racecadotril
raclopride
radezolid
RAF265
raloxifene
raltitrexed
ramatroban
ramifenazone
ranitidine
refametinib
regorafenib
remoxipride
repaglinide
repsox
resatorvid
resiquimod
resminostat
RG108
RG2833
RGFP966

rheochrysidin
RI-1
ribavirin
ribitol
ribociclib
riboflavin
rifampin
rigosertib
riluzole
rimexolone
riociguat
RITA
ritodrine
RKI-1447
RN-1734
Ro-04-5595
Ro-10-5824
Ro-106-9920
Ro-4987655
Ro-90-7501
Ro-9187
rociletinib
romidepsin
roquinimex
RS-16566
RS-17053
RS-504393
RS-67506
RU-58841
rubitecan
rucinol
rutin
ruxolitinib
ryuvidine
S26948
salidroside
salinomycin
salvianolic-acid-B
salvinorin-a
sangivamycin
SAR131675
saracatinib
sarafloxacin

satraplatin
saxagliptin
SB-200646
SB-205384
SB-216641
SB-218078
SB-218795
SB-225002
SB-228357
SB-2343
SB-239063
SB-242235
SB-366791
SB-431542
SB-505124
SB-525334
SB-590885
SB-657510
SB-743921
SB-939
SC-12267
SC-144
SCH-58261
SCH-900776
sclareol
scriptaid
SCS
SDZ-WAG-994
secnidazole
securinine
selamectin
selinexor
selumetinib
semaxanib
se-methylselenocysteine
serdemetan
sertindole
sevelamer
sevoflurane
SGI-1027
SGI-1776
SIB-1757
SID-7969543

simvastatin
sirolimus
skepinone-l
SKF-96365
SKI-II
S-methylcysteine
SMI-4a
SN-38
SNS-314
SNX-2112
SNX-5422
sobetirome
sodium-glucoheptonate
sodium-stibogluconate
sodium-tanshinone-ii-a-sulfonate
sonidegib
sorafenib
sparfloxacin
spermidine
spermine
spiradoline
spironolactone
SR-27897
SR-33805
SR-57227A
SRT1720
STA-5326
stattic
stemregenin-1
STF-118804
streptozotocin
SU014813
SU3327
sufentanil
sulconazole
sulfabenzamide
sulfamethazine
sulfanilamide
sulfasalazine
sulmazole
sunitinib
suxibuzone
tacalcitol

tacedinaline
tacrolimus
tagatose
TAK-285
TAK-632
TAK-715
TAK-733
TAK-901
talazoparib
talmapimod
taltirelin
taltobulin
TAME
tamibarotene
tamoxifen
tanaproget
tandutinib
tanespimycin
tanshinone-i
TAS-103
taselisib
tasisulam
taurine
tazemetostat
TC1
tecastemizole
tedizolid
tedizolid-phosphate
telatinib
telotristat-ethyl
temazepam
temocapril
temoporfin
temozolomide
temsirolimus
teniposide
tenovin-6
tepoxalin
teprenone
terbutaline
terconazole
terfenadine
teriflunomide

teroxirone
tetrahydropapaverine
tetrahydrouridine
tetramethylthiuram-monosulfide
tetrandrine
tezacaftor
TG-02
TG100-115
TG-100572
TG-100713
TG-101209
TG-101348
TH-302
theophylline
thiamine
thiocolchicoside
thioguanine
thiomersal
thioproperazine
thiostrepton
thiram
thonzonium
tiagabine
tideglusib
tiletamine
tilmicosin
tiludronate
tioguanine
tiotidine
tioxolone
tipifarnib
tipiracil
tipranavir
tirapazamine
tivantinib
tivozanib
TMC-353121
TMS
tofogliflozin
topotecan
torasemide
torcetrapib
toremifene

torin-1
torin-2
tosedostat
tozasertib
TPCA-1
trametinib
tranilast
trans-4-Hydroxycrotonic-acid
trapidil
tremorine
trequinsin
triamcinolone
triamcinolone-acetonide
triamterene
triapine
trichostatin-a
triciribine
triclabendazole
trifluridine
trimebutine
triptolide
trolox
trometamol
tropisetron
trovafloxacin
tryptanthrin
TU-2100
tucatinib
TW-37
TWS-119
tyloxapol
tyrphostin-A9
tyrphostin-AG-1296
tyrphostin-AG-1478
tyrphostin-AG-494
tyrphostin-AG-99
U-0126
U-18666A
ubenimex
UH-232-(+)
UK-383367
ulipristal
UNBS-5162

UNC0631
UNC2250
uprosertib
uracil
uric-acid
uridine
usniacin-(+)
valnemulin
valrubicin
vandetanib
vanoxerine
vatalanib
VE-821
VE-822
vemurafenib
venetoclax
VER-49009
verubulin
vidarabine
vinblastine
vincamine
vincristine
vindesine
vinflunine
vinorelbine
vismodegib
VLX600
volasertib
voreloxin
vorinostat
voxtalisib
VS-4718
VTP-27999
VU0238429
VU0361737
VU0364770
VX-702
VX-765
warfarin
WAY-170523
WAY-600
WHI-P154
WP1066

WP1130
WYE-125132
WYE-354
WZ-3146
WZ-4002
WZ8040
xanomeline
XBD173
xilobam
XL388
XL-647
XL888
xylazine
Y-27632
Y-320
Y-39983
YM-155
YM-201636
YM-976
YO-01027
zaldaride
zaleplon
zardaverine
ziprasidone
ZK811752
ZK-93423
ZK-93426
ZLN005
ZM-306416
ZM-447439
zolantidine
zolmitriptan
ZSTK-474

Table S9. List of potential therapeutic agents for LUAD patients with high risk scores.

Name	Source	MOA (mechanism of action)	Target	Clinical trials for cancer	Experimental evdience for LUAD treatment	Log2FC mRNA normal and tumor	CMap Score	Correlation Coefficient	Log2FC (AUC)	Wilcox FDR
leptomycin B	CTRP	CRM1inhibitor	CRM1	Preclinical	PMID: 28942004	1.065261962	NA	-0.4835711	0.76015627	6.88E-14
paclitaxel	CTRP	Tubulin inhibitor	ABCB1, BCL2, CYP2C8, MAP2, MAP4, MAPT, NR1I2, TLR4, TUBB, TUBB1	Phase 4	PMID: 15264992	0.937278083	-71.26	-0.4543806	0.49941225	4.42E-13
parbendazole	CTRP	Tubulin inhibitor	TUBB	Preclinical	NA	1.056589072	-91.12	-0.3982977	0.19645072	1.03E-10
PHA-793887	CTRP	CDK inhibitor	CCND1, CCNE1, CDK1, CDK2, CDK4, CDK5, CDK7, CDK9	Phase 1	NA	1.12434853	-96.37	-0.3504442	0.09471431	9.14E-09
triazolothiadiazine	CTRP	Akt inhibitor	GSK3B, CTNNB1, CCND1	Preclinical	PMID: 33087032	1.012088382	NA	-0.3290963	0.13024702	7.14E-08
gemcitabine	CTRP	Ribonucleotide reductase inhibitor	CMPK1, RRM1, RRM2, TYMS	Phase 4	PMID: 8616118	1.163055429	-99.12	-0.1943795	0.09729783	0.001363921
decitabine	PRISM	DNA methyltransferase inhibitor	DNMT1	Phase 4	PMID: 23908969	1.065239869	-26.67	-0.5623162	0.1872474	7.92E-16
docetaxel	PRISM	Tubulin inhibitor	BCL2, MAP2, MAP4, MAPT, NR1I2, TUBB, TUBB1	Phase 4	PMID: 12108897	0.9605375	-71.19	-0.562295	0.2849713	3.58E-16
NVP-AUY922	PRISM	HSP inhibitor	HSP90AA1, HSP90AA2, HSP90AB1	Phase 2	PMID: 23493311	1.046180632	-88.13	-0.5525201	0.3329813	2.20E-17
ganetespib	PRISM	HSP90 inhibitor	HSP90AB1, HSP90AA, HSP90AB, HSP90B, TRAP	Phase 3	PMID: 22806877	1.072983493	NA	-0.5134653	0.2215953	9.04E-15
daunorubicin	PRISM	RNA synthesis inhibitor	TOP2A, TOP2B	Phase 4	PMID: 31239668	1.170984905	-96.68	-0.5028052	0.2402994	3.65E-14
nobiletin	PRISM	MEK inhibitor	ABCB1, MAP2K1, TYR	Preclinical	PMID: 35003309	0.974279073	-95.41	-0.2614851	0.1892516	2.66E-07
gemcitabine	PRISM	Ribonucleotide reductase inhibitor	CMPK1, RRM1, RRM2, TYMS	Phase 4	PMID: 8616118	1.163055429	-99.12	-0.2467435	0.354224	4.63E-06

Table S10. List of abbreviations.

Abbreviation	Description
LASSO	least absolute shrinkage and selection operator Cox regression model
TCGA	The Cancer Genome Atlas
ROC	receiver operating characteristic
AUC	Area under the ROC curve
tAUC	Time-dependent AUC
GSEA	Gene Set Enrichment Analysis
TICs	tumor-infiltrating immune cells
HR	hazard ratio
CI	confidence interval
FDR	false discovery rate
TIDE	Tumor Immune Dysfunction and Exclusion
PC	principal component
KEGG	Kyoto Encyclopedia of Genes and Genomes
IC50	half maximal inhibitory concentration
UMAP	Uniform Manifold Approximation and Projection
PCA	Principal components analysis
TMB	Tumor mutational burden
ACC	Adrenocortical carcinoma
BLCA	Bladder Urothelial Carcinoma
BRCA	Breast invasive carcinoma
CESC	Cervical squamous cell carcinoma and endocervical adenocarcinoma
CHOL	Cholangiocarcinoma
COAD	Colon adenocarcinoma
COADREAD	Colon adenocarcinoma/Rectum adenocarcinoma Esophageal carcinoma
DLBC	Lymphoid Neoplasm Diffuse Large B-cell Lymphoma
ESCA	Esophageal carcinoma
FPPP	FFPE Pilot Phase II
GBM	Glioblastoma multiforme
GBMLGG	Glioma
HNSC	Head and Neck squamous cell carcinoma
KICH	Kidney Chromophobe
KIPAN	Pan-kidney cohort (KICH+KIRC+KIRP)
KIRC	Kidney renal clear cell carcinoma
KIRP	Kidney renal papillary cell carcinoma
LAML	Acute Myeloid Leukemia
LGG	Brain Lower Grade Glioma
LIHC	Liver hepatocellular carcinoma
LUAD	Lung adenocarcinoma

LUSC	Lung squamous cell carcinoma
MESO	Mesothelioma
OV	Ovarian serous cystadenocarcinoma
PAAD	Pancreatic adenocarcinoma
PCPG	Pheochromocytoma and Paraganglioma
PRAD	Prostate adenocarcinoma
READ	Rectum adenocarcinoma
SARC	Sarcoma
STAD	Stomach adenocarcinoma
SKCM	Skin Cutaneous Melanoma
STES	Stomach and Esophageal carcinoma
TGCT	Testicular Germ Cell Tumors
THCA	Thyroid carcinoma
THYM	Thymoma
UCEC	Uterine Corpus Endometrial Carcinoma
UCS	Uterine Carcinosarcoma
UVM	Uveal Melanoma
OS	Osteosarcoma
ALL	Acute Lymphoblastic Leukemia
NB	Neuroblastoma
WT	High-Risk Wilms Tumor
TARGET	Therapeutically Applicable Research To Generate Effective Treatments
